# Proceedings of the 2024 Childhood Arthritis and Rheumatology Research Alliance (CARRA) Annual Scientific Meeting

**DOI:** 10.1186/s12969-024-00998-w

**Published:** 2024-09-13

## A1 Factors associated with treatment response in chronic nonbacterial osteomyelitis

### Katherine Nowicki^1^, Nathan Rogers^2^, Carson Keeter^3^, Nathan Donaldson^1^, Jennifer Soep^1^, Yongdong Zhao^4^

#### ^1^University of Colorado, Children’s Hospital Colorado; ^2^Children’s Hospital Colorado; ^3^University of Colorado; ^4^Seattle Children’s Hospital

##### **Correspondence:** Katherine Nowicki

*Pediatric Rheumatology 2024*, **22(S1):**A1

Background: Chronic Nonbacterial Osteomyelitis (CNO) is characterized by sterile inflammatory bone lesions and most commonly affects skeletally immature children. Non-steroidal anti-inflammatory drugs (NSAIDs) are the first-line treatment, but in some cases second-line treatments including methotrexate, TNF-alpha inhibitors, and bisphosphonates are required. It remains unclear which patients are most likely to respond to NSAIDs or require a second-line treatment based on their initial presentation. In this study, we sought to describe our CNO cohort and to determine which clinical variables are associated with response to NSAID monotherapy versus requiring a second-line medication.

Methods : A retrospective chart review of patients with a diagnosis of CNO made before 18 years of age who attended the CNO clinic at Children’s Hospital Colorado between 1/1/05 and 1/31/22 was performed. The standardized treatment approach involved 6 months of NSAIDs, followed by a trial of discontinuation in responders. Patients who failed the discontinuation trial were given a longer NSAID course. Patients with spinal involvement, patients with comorbidities such as psoriasis or inflammatory bowel disease, and NSAID-non-responders were treated with second-line therapies. Clinical characteristics were recorded, including which of 6 regions (head and face, neck and back, upper torso, upper extremities, lower torso, lower extremities) were affected by CNO. Patients were divided into three groups: NSAID-short (NSAID monotherapy for 3 to < 7 months), NSAID-long (NSAID monotherapy for ≥7 months), or second-line treatment. A multiple linear regression model was constructed to determine the relationship between total NSAID monotherapy days and relevant predictors. Multiple logistic regression was used to determine the odds of needing second-line treatment when considering those same predictors. Both models contained combinations of variables which minimized the Akaike Information Criteria (AIC), resulting in models with low multicollinearity and high predictive power.

Results : 164 patients fulfilled inclusion criteria and 70 patients were excluded. Cohort characteristics overall and for each of the 3 treatment groups are presented in Table 1. Comparison between the NSAID-short and NSAID-long groups showed that patients with unifocal disease at diagnosis required 47% less days of NSAID treatment than those with multifocal disease at diagnosis (Table 2). Comparison of the NSAID monotherapy groups to the second-line treatment group showed that patients with 2 or more regions affected by CNO were 1.94 times more likely to require a second-line treatment (*p* < 0.05) and that patients with symmetric bone lesions were 6.86 times more likely to require a second-line treatment (*p*< 0.0001) (Table 3).

Conclusions : Our cohort is similar to other reported CNO cohorts in terms of clinical characteristics. Patients with unifocal CNO involvement at diagnosis are more likely to require shorter NSAID treatment courses. Patients with 2 or more regions affected by CNO and those with symmetric bone lesions are more likely to require a second-line treatment. These findings may inform treatment choices for patients with CNO.

IRB Statement : This retrospective chart review study involving human participants was in accordance with the ethical standards of the institutional and national research committee and with the 1964 Helsinki Declaration and its later amendments or comparable ethical standards. The Institutional Review Board of record of records, the Colorado Multiple Institutional Review Board (COMIRB) approved this study. Informed consent and assent were obtained from all individual participants enrolled in this study after January 1, 2018 as data was collected prospectively, but consent was waived for those patients enrolled prior to January 1, 2018 given the retrospective nature of data collection for these patients.

**Table 1 (Abstract A1):** Cohort characteristics

	NSAID-short (***n***=32)	NSAID-long (***n***=62)	Second-line (***n***=70)	***P*** value	Overall (***n***=164)
**Mean age at symptom onset, years (± SD)**	8.46 (± 4.41)	8.94 (±3.05)	8.99 (±3.36)	0.757	8.87 (±3.46)
**Mean interval from symptom onset to treatment onset, days (± SD)**	124 (±210)	269 (±333)	293 (±388)	**0.0588**	251 (±343)
**Mean interval from symptom onset to diagnosis, days (± SD)**	119 (±211)	270 (±327)	324 (±403)	**0.0226**	263 (±351)
**Mean follow-up, years (± SD)**	1.21 (±1.51)	2.70 (±2.07)	3.83 (±2.34)	**<0.001**	2.89 (±2.30)
**Female sex, n (%)**	17 (53%)	37 (60%)	42 (60%)	0.775	96 (59%)
**Race and ethnicity, n (%)**				0.714	
Asian	1 (3%)	0 (0%)	1 (1%)		2 (1%)
Black	1 (3%)	1 (2%)	1 (1%)		3 (2%)
Multiracial	0 (0%)	2 (3%)	3 (4%)		5 (3%)
Native American	0 (0%)	0 (0%)	1 (3%)		1 (1%)
White	29 (91%)	56 (90%)	59 (84%)		144 (88%)
Other	0 (0%)	1 (2%)	4 (6%)		5 (3%)
Unknown	0 (0%)	1 (2%)	0 (0%)		1 (1%)
Missing	1 (3%)	1 (2%)	1 (1%)		3 (2%)
**Ethnicity, n (%)**				0.0835	
Hispanic	0 (0%)	6 (10%)	11 (16%)		17 (10%)
Non-Hispanic	31 (97%)	54 (87%)	58 (83%)		143 (87%)
Unknown	0 (0%)	1 (2%)	0 (0%)		1 (1%)
Missing	1 (3 %)	1 (2%)	1 (1%)		3 (2 %)
**Family history, n (%)**					
Inflammatory arthritis	0 (0%)	3 (5%)	5 (7%)	0.274	8 (5%)
Inflammatory Bowel Disease	0 (0%)	0 (0%)	2 (3%)	0.361	2 (1%)
Psoriasis	0 (0%)	1 (2%)	1 (1%)	1	2 (1%)
**Mean ESR at presentation, mm/hr (± SD)**	19.7 (±18.5)	19.6 (±19.6)	27.6 (±25.3)	0.093	22.8 (±20.5)
Missing ESR, n (%)	3 (9.4%)	13 (21%)	17 (24%)		33 (20%)
**Mean CRP at presentation, mg/dL (± SD)**	1.57 (±2.96)	0.950 (±2.14)	2.69 (±4.88)	0.0601	1.83 (±3.75)
Missing CRP, n (%)	3 (9.4%)	16 (25.8%)	15 (21.4%)		34 (20.7%)
**Biopsy performed, n (%)**	22 (69%)	46 (74%)	66 (94%)	**0.0025**	134 (82%)
**CNO lesion apparent on plain radiographs at presentation, n (%)**	19 (59%)	36 (58%)	31 (44%)	0.229	86 (52%)
Missing, n (%)	1 (3 %)	0 (0%)	2 (3%)		3 (2%)
**Unifocal disease suspected at diagnosis, n (%)**	23 (72%)	29 (47%)	29 (41%)	**0.015**	81 (49%)
**Total number of whole-body MRIs, n (%)**				**<0.001**	
0	28 (88%)	51 (82%)	29 (41%)		106 (66%)
1	4 (12%)	8 (13%)	16 (23%)		28 (17%)
2	0 (0%)	3 (5%)	8 (11%)		11 (7%
≥3	0 (0%)	0 (0%)	17 (24%)		17 (10%)
**Patients with affected regions, n (%)**					
Head & face	0 (0%)	0 (0%)	3 (4%)	0.233	3 (2%)
Upper torso	3 (9%)	8 (13%)	9 (13%)	0.902	20 (12%)
Upper extremity	4 (12%)	11 (18%)	22 (31%)	**0.046**	37 (23%)
Neck and back	2 (6%)	9 (15%)	18 (26%)	**0.046**	29 (18%)
Lower torso	7 (22%)	19 (31%)	33 (47%)	**0.029**	59 (36%)
Lower extremity	23 (72%)	34 (55%)	59 (84%)	**<0.001**	116 (71%)
**Mean number out of 6 regions affected (± SD)**	1.22 (± 0.608)	1.31 (± 0.561)	2.06 (± 1.03)	**<0.001**	1.61 (± 0.890)
**Symmetric involvement in the same bone, n (%)**	5 (16%)	14 (23%)	51 (73%)	**<0.001**	70 (43%)
**Mean days on NSAID monotherapy, days (± SD)**	175 (± 26.5)	725 (±512)	441 (±536)	**<0.001**	497 (±511)
**Number of NSAIDs Trialed, n (%)**				**<0.001**	
0	0 (0%)	0 (0%)	2 (3%)		2 (1%)
1	30 (94%)	28 (45%)	17 (24%)		75 (46%)
2	2 (6%)	32 (52%)	45 (64%)		79 (48%)
3 or more	0 (0%)	3 (3%)	6 (9%)		8 (5%)
**Patients with complications, n (%)**					
Vertebral height loss	0 (0%)	8 (13%)	14 (20%)	**0.0215**	22 (13%)
Amplified pain	0 (0%)	3 (5%)	10 (14%)	**0.02**	13 (8%)
**Patients in each disease activity state at study end date, n (%)**					
Active disease	0 (0%)	7 (11%)	14 (20%)	**0.024**	21 (13%)
Inactive disease on treatment	2 (6%)	11 (18%)	20 (29%)	**0.0285**	33 (20%)
Remission	30 (94%)	44 (71%)	36 (51%)	**<0.001**	110 (67%)

**Table 2 (Abstract A1):** Linear regression modeling with AIC-based variable selection comparing the NSAID-short and NSAID-long groups

Study Variable	Coefficient	***P***-value
**Unifocal disease at diagnosis**	**-0.386**	**0.029**
Number of regions affected	-0.225	0.141
Onset to treatment interval	0.000	0.162

On average, patients with unifocal disease at diagnosis require 47.11% less days of total NSAID monotherapy treatment than patients with multifocal disease at diagnosis. *P*-values here correspond to a one-way ANOVA for numeric variables and chi-squared test for categorical variables

**Table 3 (Abstract A1):** Logistic regression modeling of the odds of requiring second-line treatment with IC-based variable selection

Study Variable	OR	***P***-value
**Presence of symmetric bone lesions**	**6.862**	**<0.001**
**Number of regions affected**	**1.941**	**0.012**
Positive family history	3.770	0.113
Days from symptom onset to treatment	1.000	0.116

For each additional region affected by CNO, the odds of needing second-line therapy increased by a factor of 1.94 on average. Patients with symmetric bone lesions were, on average, 6.86 times more likely to require second-line therapy than patients without symmetric bone lesions

## A2 Predicting extension in juvenile idiopathic arthritis

### Megan Simonds^1^, Kathleen Sullivan^2^, AnneMarie Brescia^1^

#### ^1^Nemours Children’s Health, Wilmington, DE, USA; ^2^Children’s Hospital of Philadelphia, Philadelphia, PA, USA

##### **Correspondence:** Megan Simonds

*Pediatric Rheumatology 2024*, **22(S1):**A2

Background: Juvenile idiopathic arthritis (JIA) carries a risk of permanent joint damage and disability in children [1]. In adult rheumatoid arthritis (RA), early aggressive treatment can lead to remission. Treatment paradigms in JIA remain reactive rather than proactive due to lack of reliable methods to predict extension to a polyarticular course. Our objective was to validate cell subpopulations using flow cytometry to predict which patients have a high likelihood of extending.

Methods: JIA FLS cell lines from oligoarticular (oligo), extended-to-be (ETB), and polyarticular (poly) types were cultured. Flow cytometry was performed by Raybiotech, Inc. scRNA-seq was performed by Genewiz using 10x Genomics Chromium protocols. SeuratR package was used for data analysis.

Results: Our scRNA-seq data showed that fibroblast-like synoviocytes (FLS) are heterogeneous [2]. Prominent subpopulations were FLS, smooth muscle cell-like cells (SMC), and chondrocyte-like cells (CH) (Figure 1). We performed flow cytometry on normal cell lines to determine if traditional markers for FLS, SMC, and CH can distinguish these cell types from one another. These markers did not confidently isolate these cell types (Table 1). We further analyzed our scRNA-seq data for markers that could distinguish JIA subtypes via flow cytometry (CH – SMOC2, SOX9; FLS – IGFBP4, VCAN; SMC – CD309). These markers could not distinguish FLS from CH using flow. We revisited Seurat single analysis data and identified the top genes of each cell type for each subtype. Genes that contribute the most variation among cells within oligo and ETB JIA subtypes and encode secreted proteins are as follows: oligo - COL1A1, ACAN, and IFI27, ETB - SERPINE1, COL3A1, and VCAM (Figure 2).

Conclusions: While flow cytometry has proven unable to distinguish between cell subpopulations within FLS cultures, these cell subpopulations have unique genetic fingerprints with transcript expression levels that can be used to distinguish JIA subtypes.

IRB Statement : Synovial fluid and fibroblast-like synoviocyte samples were obtained from the Nemours BioMedical Institutional Review Board-approved repository (IRB#84709-32). This study was performed in accordance with the Helsinki Declaration of 1964, and its later amendments. Patients who underwent clinically indicated arthrocentesis were offered inclusion into the repository and informed consent was obtained. There is no identifying information in this publication.

Acknowledgements : This study was funded by a CARRA-Arthritis Foundation Large grant. The authors wish to acknowledge CARRA and the ongoing Arthritis Foundation financial support of CARRA.

Reference

1. Manners, P.J. and C. Bower, Worldwide prevalence of juvenile arthritis why does it vary so much? The Journal of rheumatology, 2002. 29(7): p. 1520-1530. 2. Simonds M, S.K., Rose C, Brescia A. Heterogeneity of Juvenile Idiopathic Arthritis Synovial Fibroblasts Correlates to Disease Progression and Provides Compelling Diagnostic Data [abstract]. Arthritis Rheumatol 2022 (cited 73 May 19, 2022)

**Fig. 1 (Abstract A2):**
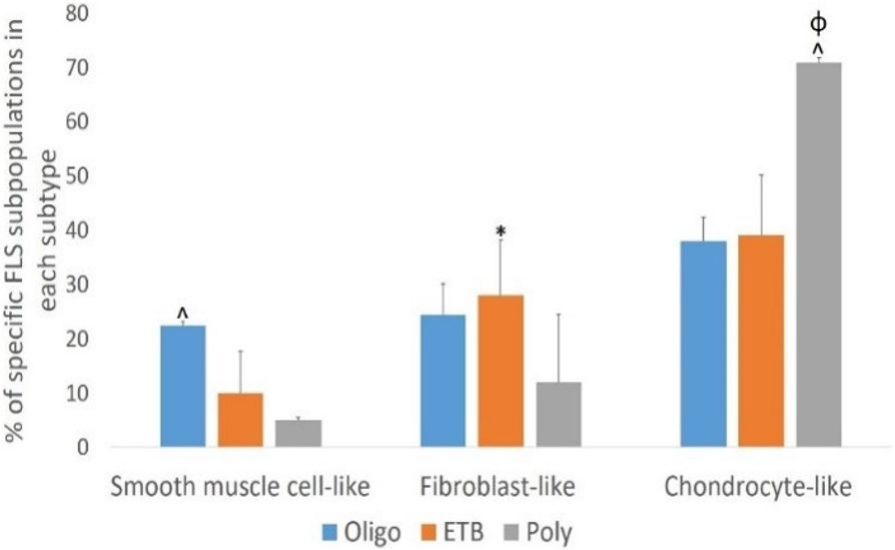
Percentage of FLS subpopulations in cultured FLS from patients with oligoarticular (Oligo), extended-to-be (ETB), and polyarticular (Poly) JIA. The percentage of smooth muscle-cell-like cells decreases in more severe subtypes of JIA; fibroblast-like cells significantly increased in ETB compared to Oligo; and chondrocyte-like cells increases in more severe subtypes of JIA. ^*p*<0.0006 Oligo vs. Poly; *p=0.03 Oligo vs. ETB; fp=0.0044 ETB vs. Poly

**Table 1 (Abstract A2):** Traditional markers from literature to distinguish cell types

A.					B.				
		FLS	CH	SMC			FLS	CH	SMC
	**CD55**	+++	++	++		**CD55**	+	+	++
	**CD271**	Negative	Negative	Negative		**CD271**	Negative	Negative	Negative
	**CD309**	Negative	Negative	+++		**CD309**	+	Negative	+
	**CD146**	Negative	+	++		**CD146**	+	+	+
	**CD45**	Negative	Negative	Negative		**CD45**	Negative	Negative	Negative
	**CD166**	+++	+++	+++		**CD166**	+++	+++	+++
	**CD44**	+++	+	+++		**CD44**	+++	+++	+++
	**CD105**	+++	+	++		**CD105**	++	++	+++
	**CD31**	Negative	Negative	+++		**CD31**	Negative	Negative	Negative
	**CD90**	+++	+++	+		**CD90**	+++	+++	++

Expected outcomes based on literature (A). Actual outcomes based on flow cytometry (B)

**Fig. 2 (Abstract A2):**
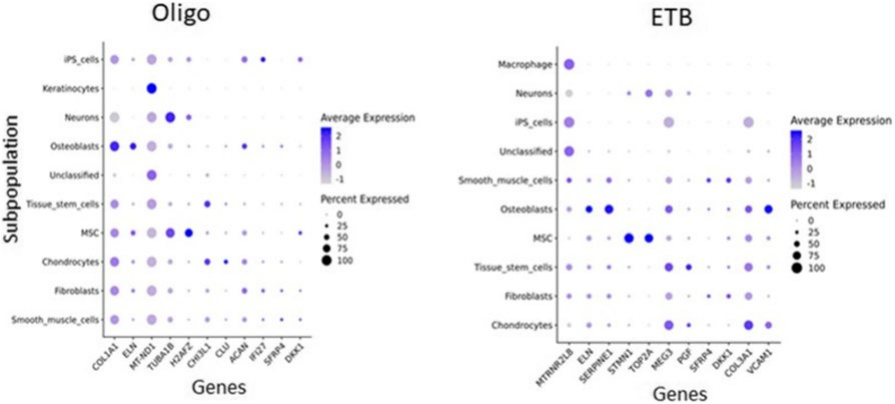
Genes that are the highest contributors to variation among subpopulations. Genes that contribute the most variation among cell subpopulations are as follows: oligo - COL1A1, MT-ND1, TUBA1B, ACAN, and IFI27, ETB - MTRNR2LB, SERPINE1, TOP2A, MEG3, COL3A1, and VCAM, poly - ACP5, SPP1, TNFAIP6, IGFBP5, AKAP12, MFAP5, and S100A4

## A3 Tubulointerstitial inflammation is associated with end-stage renal disease in pediatric lupus nephritis, single center retrospective cohort study

### Ryan Mitacek, Qiong Liu, Anthony Chang, Shireen Hashmat, Linda Wagner-Weiner

#### University of Chicago, Chicago, IL, USA

##### **Correspondence:** Ryan Mitacek

*Pediatric Rheumatology 2024*, **22(S1):**A3

Background : Lupus nephritis (LN) is associated with significant morbidity and mortality. The 2018 revised International Society of Nephrology/Renal Pathology Society (ISN/RPS) classification criteria and the NIH chronicity activity indices are used for LN classification. Some adult studies report tubulointerstitial (TI) inflammation is an independent predictor of renal outcomes in LN, however its impact in pediatric LN remains unexplored.

Methods : We conducted a retrospective, observational cohort study utilizing a subgroup analysis of patients with biopsy-confirmed LN. Inclusion criteria encompassed all patients with biopsy-confirmed lupus nephritis, where the renal biopsies were performed at the University of Chicago between January 1, 2006, and September 6, 2022. Patients were required to be ≤ 21 years of age at the time of their initial kidney biopsy. Exclusion criteria consisted of a follow-up duration of ≤ 3 months and an age ≥ 16 years at the time of initial lupus diagnosis. Non-native renal biopsies were also excluded. The primary outcome measure was end-stage renal disease (ESRD).

Results : Out of 99 identified biopsies, 42 were excluded, leaving 57 patients for analysis (see figure 1). The mean follow-up duration was 74 months (SD 26, max 210). Tubulointerstitial (TI) inflammation demonstrated a significant association with ESRD, with a hazard ratio of 6.7 (95% CI 1.8 – 24.7). Patients with TI inflammation experienced a shorter time to ESRD (mean 21.51 months, SD 20.32) compared to those without TI inflammation (mean 64.33 months, SD 37.65; see figure 2). Single variable analysis showed ESRD was also correlated with the NIH activity index (HR 1.17, 95% CI 1.06 – 1.3), the NIH chronicity index (HR 2.7, 95% CI 1.57 – 4.65), GFR (HR 6.7, 95% CI 2.7-17), and ISN class IV nephritis on biopsy (HR 6.7, 95% CI 2.15 – 21.4). However, ESRD was not correlated with race, sex, Hispanic ethnicity, or SLEDAI (see table 1). This retrospective study had a limited number of ESRD events.

Conclusions : Our findings indicate a significant association between tubulointerstitial (TI) inflammation, baseline activity / chronicity indices, initial GFR stage, ISN Class IV and the primary endpoint of ESRD in our pediatric lupus nephritis cohort. The presence of TI inflammation was also associated with a shorter time to ESRD. We are performing ongoing renal biopsy analysis to assess the impact of TI injury as measured by the NIH chronicity index to better understand the impact of TI involvement and renal outcomes.

IRB Statement : This research involved human materials and data. It was performed in accordance with the Declaration of Helsinki and has been approved by the local Institutional Review Board (ID: IRB22-1421).

**Fig. 1 (Abstract A3):**
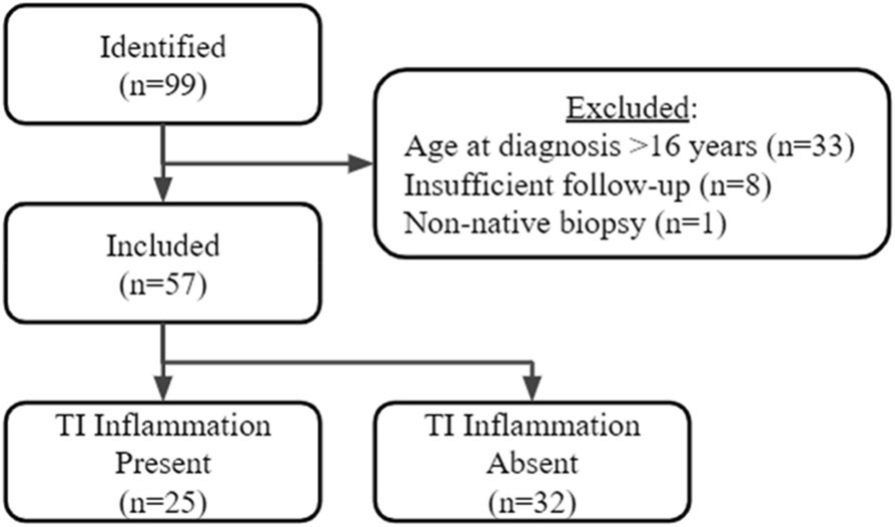
Flowchart of selected study population

**Fig. 2 (Abstract A3):**
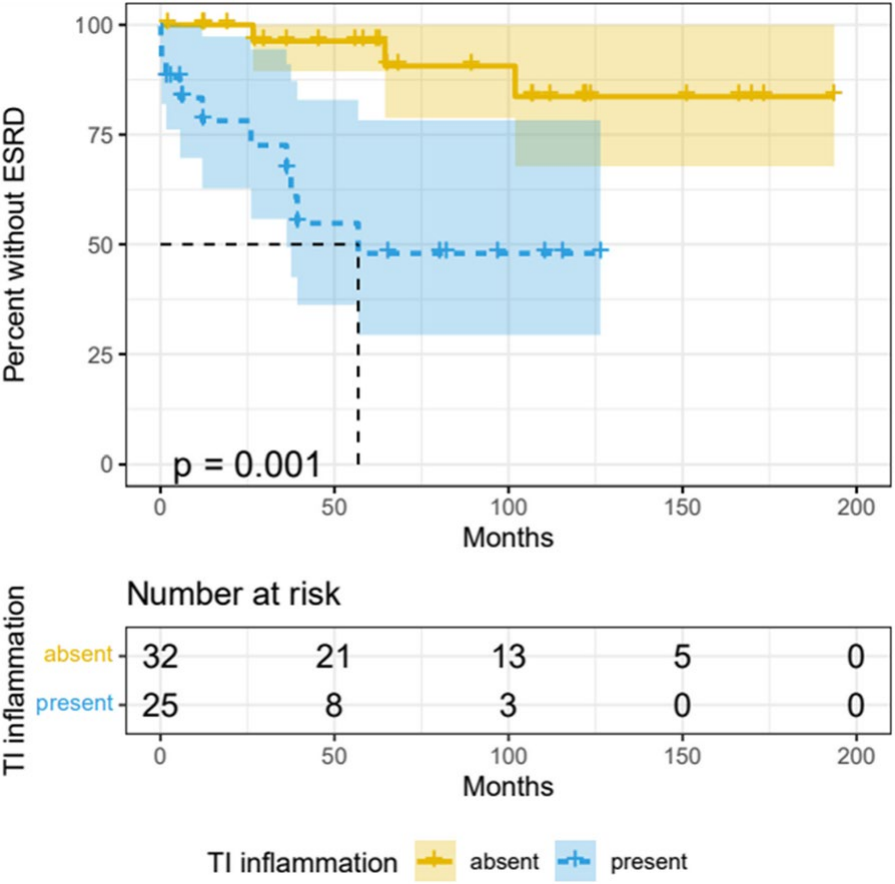
Time to End Stage Renal Disease. Univariate survival curve showing percentage of patients without ESRD or dialysis, stratified by tubulointerstitial inflammation (TI) on biopsy

**Table 1 (Abstract A3):** Demographics, baseline and biopsy characteristics, and select significant variables

n	Total	ESRD		
		No	Yes	*p*-value
	57	44	13	
**Demographic data**				
Age at SLE diagnosis, years (mean [SD])	12.86 (2.56)	12.60 (2.70)	13.67 (1.90)	0.201
Female (%)	45 (78.9)	36 (81.8)	9 (69.2)	0.555
Hispanic (%)	15 (26.3)	13 (29.5)	2 (15.4)	0.122
Race (%)				0.348
African American	39 (68.4)	28 (63.6)	11 (84.6)	
Multiracial	11 (19.3)	10(22.7)	1 (7.7)	
Caucasian	7 (12.3)	6 (13.6)	1 (7.7)	
**Index Biopsy Data**				
Age at biopsy, years (mean [SD])	57	13.90 (3.13)	15.32 (3.28)	0.185
ISN Class	57			
I		2 (3.4)	0 (0)	0.434
II		10 (17.5)	1 (4.3)	0.179
III		9 (19.1)	3 (13.6)	0.839
IV		9 (23.7)	8 (42.1)	**0.004**
V		14 (48.3)	1 (9.1)	0.072
TI Inflammation	57	15 (100)	10 (100)	**0.006**
NIH Activity Index (mean [SD])	57	3.23 (4.21)	9.08 (5.65)	**<0.001**
NIH Chronicity Index (mean [SD])	57	0.4 (0.9)	2.5 (3.4)	**0.008**
**Baseline data**				
CKD Stage				**<0.001**
Stage 1	45	26 (72.2)	2 (22.2)	
Stage 2		8 (22.2)	0 (0)	
Stage 3a		1 (2.8)	4 (26.7)	
Stage 3b		1 (2.8)	1 (8.3)	
Stage 4			0 (0)	
Stage 5		0 (0)	2 (14.3)	
Weight Category^a^	48		9	
Underweight		2 (5.1)	0 (0)	0.83
Healthy weight		21 (53.8)	6 (66.7)	
Overweight		9 (23.1)	2 (22.2)	
Obese		7 (17.9)	1 (11.1)	
Hypertension category	51			
Normal blood pressure	51	18 (46.2)	3 (37.5)	**0.029**
Elevated		4 (10.3)	2 (25)	
Hypertension stage I		12 (30.8)	1 (12.5)	
Hypertension stage II		5 (12.8)	2 (25)	
SLEDAI (mean [SD])	6.1 (9.4)	14.4 (8.8)	22.0 (9.1)	0.096
BUN (mean [SD])	47	15.4 (8.2)	34.9 (28.0)	**0.013**
Creatinine (mean [SD])	50	0.665 (0.273)	2.458 (2.915)	**0.003**
eGFR (mean [SD])	43	112.0 (37.3)	60.6 (48.5)	**0.005**

*Abbreviations*: *ESRD* End stage renal disease, *CKD* chronic kidney disease, *TI* tubulointerstitial.

^a^Weight status was determined using the CDC’s definition for obesity: Underweight indicates BMI <18.5 (<5th%ile if under 19 years old). Healthy weight indicates BMI ≥18.5 to <25 (≥5 to <85%ile if under 19 years old). Overweight indicates BMI ≥25.0 to <30 (≥85 to <95%ile if under 19 years old). Obese indicates BMI ≥30.0 (≥95%ile if under 19 years old). CKD Stage was determined using the National Kidney Foundation criteria

## A4 Clinical outcomes in down syndrome-associated arthritis compared to juvenile idiopathic arthritis

### Irene Chern^1^, Jordan Jones^2^

#### ^1^St. Christopher’s Hospital for Children, Philadelphia, PA, USA; ^2^Children’s Mercy Kansas City

##### **Correspondence:** Irene Chern

*Pediatric Rheumatology 2024*, **22(S1):**A4

Background : Down syndrome (DS) is one of the most common chromosomal conditions that affects approximately 1 in 1000 live births globally. Triplication of chromosome 21 seen in DS is associated with a four-to-six-fold higher risk of autoimmunity compared to the general population. Individuals with DS have a significantly higher prevalence of inflammatory arthritis, termed Down Syndrome-associated arthritis (DA), compared to the general population. Historically, when compared to juvenile idiopathic arthritis (JIA), the most common pediatric rheumatic disease, there has been a delay in diagnosis of DA. Additional research is needed to better elucidate the similarities and differences between DA and JIA, to improve the care of individuals with DA, and allow for earlier diagnosis and treatment. The objective of this study was to compare the clinical presentation and outcomes between DA and JIA in the Pediatric Rheumatology Care & Outcomes Improvement Network (PR-COIN) registry.

Methods : The PR-COIN registry was used to perform a retrospective case-control study that compared patients with DA matched to patients with JIA. Patients were matched on age, gender, arthritis subtype, and medication exposure. Pain rating and clinical juvenile arthritis disease activity scores ([cJADAS], which is a composite measure of active joint count, physician and patient global assessments [MD-global, Pt-global]), were compared between DA and JIA groups.

Results : Twenty patients with DA and 100 with JIA were identified. The mean days between first and last visits were 1157 for patients with JIA and 1664 for DA. Patients mostly had polyarticular arthritis subtype (70%), and those with DA had more comorbid autoimmune conditions, but less uveitis compared to the JIA group (Table 1). At the last visit those with DA had lower cJADAS scores compared to the JIA group. All measures of the cJADAS were decreased with the largest decrease in Pt-global scores when comparing between DA and JIA groups. The DA group had an average pain score that improved over time and trended with MD-global score; whereas the JIA group had a pain score that increased over time and did not trend with MD-global score (Table 2). The medication distribution and exposure were the same between groups (Table 3).

Conclusions: While a delay in diagnosis of DA is not uncommon, this study suggests that with appropriate treatment patients with DA can have similar clinical outcomes compared to those with JIA. Additionally, those with DA report less pain compared to those with JIA, and reported pain tracked with active disease for those with DA, but not JIA. This suggests that with appropriate treatment patients with DA demonstrate good resilience and improved clinical outcomes. Further, pain rating may be a poor outcome measure for active disease in patients with JIA and may represent a larger impact of central nervous system sensitization and mental health comorbidities compared to patients with DA. Here we illustrate the importance of early disease recognition and treatment, which can improve outcomes for those with DA. More research is needed to determine differences and impact of pain perception between those with JIA and DA.

IRB Statement : This study has been granted an exemption from IRB by the Children’s Mercy Research Institute IRB. The study meets criteria for Exemption Determination under Code of Federal Regulations 45 CFR 46.106 (d) category 4 (iii). Approval for exemption was effective as of 5/8/2023. CM IRB number STUDY00002736 entitled “Arthritis Outcomes for Those with Down Syndrome in the PR-COIN Registry”

Acknowledgements : We would like to acknowledge the contributions of Jade Singleton, PhD and Xing Wang, PhD, biostatisticians for the Pediatric Rheumatology Care & Outcomes Improvement Network (PR-COIN) registry. Dr. Singleton and Dr. Wang provided significant assistance in analyzing the data on this project; however, were not involved in the conceptualization of the project nor the drafting or revision of the final abstract. We will be disclosing their involvement to the learner in written form at any presentation of this study.

**Table 1 (Abstract A4):** Arthritis subtype and comorbidities compared between DA and JIA controls

	First Visit		Second Visit		Most Recent Visit	
	JIA (*n* = 100)	DA (*n*= 20)	JIA (*n* = 100)	DA (*n*= 20)	JIA (*n* = 100)	DA (*n*= 20)
Arthritis Subtype						
Enthesitis Related	5 (5.0%)	1 (5.0%)	5 (5.0%)	1 (5.0%)	4 (4.0%)	1 (5.0%)
Oligoarticular	20 (20%)	4 (20%)	19 (19%)	5 (25%)	20 (20%)	3 (15%)
Polyarticular	70 (70%)	14 (70%)	70 (70%)	13 (65%)	63 (63%)	12 (60%)
Systemic	5 (5.0%)	1 (5.0%)	5 (5.0%)	1 (5.0%)	0 (0%)	1 (5.0%)
Undifferentiated	0 (0%)	0 (0%)	0 (0%)	0 (0%)	5 (5.0%)	1 (5.0%)
Comorbidity						
Celiac disease	0	1 (5%)	0	1 (5%)	0	2 (10%)
Heart disease	0	0	0	0	0	1 (5%)
Thyroid disorder	0	2 (10%)	0	1 (5%)	0	1 (5%)
Liver disease	0	1 (5%)	0	0	0	0
Uveitis active	4 (4%)	0	1 (1%)	0	3 (3%)	0

**Table 2 (Abstract A4):** Clinical outcomes compared between DA and JIA controls

	First Visit		Second Visit		Most Recent Visit	
	JIA (*n* = 100)	DA (*n*= 20)	JIA (*n* = 100)	DA (*n*= 20)	JIA (*n* = 100)	DA (*n*= 20)
Pain Rating^a^	2.0(2.3)	2.1(2.9)	2.2(2.3)	1.6(2.6)	2.8(2.6)	1.2(1.8)
Number of Joints Affected^b^	1.6(2.8)	2.0(3.3)	1.2(2.4)	1.2(1.8)	0.9(1.9)	0.3(0.6)
Physician Global Assessment^c^	1.3(1.6)	1.2(1.8)	1.0(1.5)	0.9(1.5)	0.9(1.3)	0.4(0.9)
Patient Global Assessment^d^	2.3(2.2)	2.3(2.5)	2.2(2.3)	2.2(3.2)	2.1(2.1)	1.1(2.1)
cJADAS Score^e^	5.1(5.2)	5.4(6.9)	4.5(4.9)	4.3(5.5)	3.9(4.2)	1.8(2.5)

^a^Pain Rating (Mean (SD))- patient reported arthritis related pain over past week 0-10, higher is worse

^b^Number of Joints Affected (Mean (SD))- physician count of number of affected joints

^c^Physician Global Assessment (Mean (SD))- physician global assessment of arthritis activity 0-10, higher is worse

^d^Patient Global Assessment (Mean (SD))- patient global assessment of overall health 0-10, higher is worse

^e^cJADAS Score (Mean (SD))- composite measure of active joint count, physician global assessment, and patient global assessment, higher is worse

**Table 3 (Abstract A4):** Medication exposure in DA patients with matched JIA controls

Medication Class	JIA (*N*=100)	DA (*N*=20)
BDMARD^a^	40 (40%)	8 (40%)
BDMARD^a^+CSDMARD^b^	30 (30%)	6 (30%)
BDMARD^a^+CSDMARD^b^+SMDARD^c^	5 (5.0%)	1 (5.0%)
CSDMARD^b^	25 (25%)	5 (25%)

^a^BDMARD-Biologic Disease Modifying Antirheumatic Drug (e.g. etanercept, adalimumab, infliximab, etc.)

^b^CSDMARD-Conventional Synthetic Disease Modifying Antirheumatic Drug (e.g. methotrexate, hydroxychloroquine, sulfasalazine, etc.)

^c^SMDMARD- Small Molecule Disease Modifying Antirheumatic Drug (e.g. tofacitinib, baricitinib, etc.)

## A5 Perceptions and attitudes about physical activity in juvenile idiopathic arthritis

### Ana Leos-Leija^1^, Fernando Garcia-Rodriguez^1^, Ingris Pelaez-Ballestas^2^, Gabriela Burgos^3^, Adalberto Loyola-Sanchez^4^, Ana Villarreal-Treviño^1^, Nadina Rubio-Perez^1^

#### ^1^Hospital Universitario “Dr. Jose Eleuterio Gonzalez”, Monterrey, Nuevo Leon, Mexico; ^2^Hospital General de Mexico “Dr. Eduardo Liceaga”, Ciudad de Mexico, Mexico; ^3^Alberta Health Services, Alberta, Canada; ^4^University of Alberta, Alberta, Canada

##### **Correspondence:** Ana Leos-Leija

*Pediatric Rheumatology 2024*, **22(S1):**A5

Background : Exercise can improve general health and, in patients with juvenile idiopathic arthritis (JIA), it helps with common problems such as fatigue, weakness, and pain, increasing quality of life. However, there are barriers to exercising regularly when facing JIA, and commonly, these patients do not complete the minimum daily exercise requirements and maintain a sedentary lifestyle.

Methods : An observational, cross-sectional, prospective, descriptive study, with a mixed and sequential evaluative study design, including qualitative techniques, was carried out. The population included patients with JIA and their caregivers; pediatric rheumatologists; and sports coaches or physical education teachers. A semi-structured interview guide was created by a group of experts (3 pediatric rheumatologists, 1 physiotherapist, 1 methodologist/anthropologist, 1 patient, 1 caregiver, and 1 Physical Medicine and Rehabilitation physician), and it was evaluated in a pilot study to confirm its relevance and understanding. The interviews aimed to assess the perspectives and attitudes about physical activity (PA) and exercise, including their knowledge, adherence, motivations, preferences, barriers, and facilitators to do it.

Results : We included 20 patients with a median age of 12 (9-15), 11 (55%) were men. The most common JIA subtype was enthesitis-related arthritis (7, 35%). Nine patients (45%) had high disease activity at the time of the interview and 7 (35%) had inactive disease. Most patients (12, 60%) did not exercise regularly, the main reasons and barriers were disease activity, pain, and concerns about being injured. Of the patients who exercised regularly (8, 40%), half reported doing so for pleasure and the other half to improve disease symptoms. Twelve patients (60%) exercised before the onset of the disease, however, half stopped due to disease activity, and those who did exercise regularly reported emotional and physical benefits. Fourteen (70%) patients reported that they had received information about the importance of exercise, in most cases given by the doctors. There was observed confusion about the differences between PA and exercise in most of the participants, independently of the group.

Conclusions : We observed confusion about the differences between PA and exercise, and myths and fears about the risks of performing it while having JIA. Most of the patients do not exercise regularly, with pain and high disease activity being the main barriers. On the other hand, those who did exercise regularly reported emotional and physical benefits.

IRB Statement : The study complies with the ethical guidelines of the Declaration of Helsinki and local regulations. This is a minimal-risk study since it only involves the application of interview guides to the participants and does not require biological sampling, or invasive procedures to the participants. An institutional review board approved the protocol and other written information provided to patients and their legal representatives.

## A6 Unveiling clinical predictors of methotrexate discontinuation in juvenile idiopathic arthritis

### Ivana Stojkic, Edward Oberle, Kelly Wise, Stacy Ardoin, Alysha Taxter

#### Nationwide Children’s Hospital, Columbus, OH, USA

##### **Correspondence:** Ivana Stojkic

*Pediatric Rheumatology 2024*, **22(S1):**A6

Background : Methotrexate (MTX) is commonly utilized for treating JIA. Despite its longstanding clinical use, we are unable to predict responders in advance. Identifying clinical factors predictive of response to MTX would optimize patient care and reduce morbidity. We aimed to identify clinical characteristics predictive of MTX response in patients with JIA.

Methods : Subjects with JIA diagnosed by pediatric rheumatologists in a large quaternary care hospital and treated with MTX monotherapy from 2009 to 2022 were included. Retrospective chart review determined clinical response and reason for discontinuation. Unadjusted univariate time-varying Cox proportional hazards were used to model the association between time to MTX discontinuation with various clinical characteristics and concomitant systemic therapies. Variables with *p*< 0.2 in univariate analysis were included in multivariate model.

Results : There were 589 patients identified across 3674 encounters started on MTX within the study period. There were 401 females (68%), with a median age of 11 years [IQR 6, 14], symptom duration prior to JIA diagnosis of 16 months [8, 46], and disease duration of 63 days [0, 297] at initiation of MTX therapy. Discontinuation rate of MTX due to side effects was 36%. ANA positivity and disease duration had significantly lower hazards or discontinuing MTX therapy on univariate analysis. Symptom duration greater than 6 months, morning stiffness, poor physical function, increased pain, increased patient and provider global scores, increased active joint count, increased disease activity score, and higher baseline patient-reported outcomes had higher hazards of discontinuing MTX therapy (Table 1, figure). Rheumatoid factor negative and positive polyarthritis, enthesitis related arthritis, and psoriatic arthritis were associated with significantly higher hazards of stopping MTX in univariate analysis. In multivariate analysis, no variables were associated with discontinuation of MTX therapy (Table 2).

Conclusions : It remains challenging to model MTX response based solely on clinical factors. This highlights the need for further research looking into artificial intelligence, pharmacogenomic biomarkers, and incorporation of genomic data into predictive modeling.

IRB Statement : This study was approved by the Nationwide Childrens Hospital IRB on 3/3/23 under IRB study ID STUDY00003136.

**Table 1 (Abstract A6):** Univariate cox analysis of time to methotrexate discontinuation

	Hazard Ratio (95%CI)	*p*-value
Male sex	1.06 (0.87, 1.30)	0.55
Symptom duration more than 6 months	1.15 (0.96, 1.13)	0.13
Disease duration^a^	0.99 (0.99, 0.99)	<0.01
ANA positive	0.80 (0.65, 0.98)	0.03
NSAID therapy^a^	0.98 (0.75, 1.27)	0.85
Receipt of joint injection^a^	1.06 (0.56, 1.99)	0.85
Morning stiffness^a^	1.66 (1.24, 2.23)	<0.01
CHAQ^a^	1.87 (1.39, 2.50)	<0.01
Pain^a^	1.08 (1.03, 1.14)	<0.01
Patient global assessment^a^	1.11 (1.06, 1.17)	<0.01
Physician global assessment^a^	1.36 (1.25, 1.48)	<0.01
Active joint count^a^	1.09 (1.07, 1.11)	<0.01
JADAS^a^	1.09 (1.07, 1.12)	<0.01
Baseline CHAQ	1.56 (1.10, 2.21)	0.01
Baseline patient global	1.14 (1.06, 1.24)	<0.01
Baseline pain	1.09 (1.02, 1.17)	0.02
Baseline morning stiffness	1.66 (1.03, 2.66)	0.04
Baseline provider global	1.38 (1.20, 1.58)	<0.01
Baseline active joint count	1.06 (1.03, 1.09)	<0.01
Uveitis^a^	1.46 (0.85, 2.15)	0.16
JIA category		
Oligoarticular	Reference	NA
RF negative polyarticular	1.41 (1.11, 1.11)	<0.01
RF positive polyarticular	2.30 (1.16, 3.41)	<0.01
Enthesitis-related	1.74 (1.32, 2.32)	<0.01
Psoriatic	1.56 (1.17, 2.07)	<0.01
Undifferentiated	1.16 (0.61, 2.21)	0.65
Systemic	1.30 (0.57, 2.96)	0.53

Symptom duration >6 months prior to JIA diagnosis

*ANA* antinuclear antibody, *NSAID* nonsteroidal anti-inflammatory drug, *CHAQ* Childhood Health Assessment Questionnaire, *JADAS* juvenile arthritis disease activity score, *JIA* juvenile idiopathic arthritis

^a^Evaluated as a time-varying covariate

**Table 2 (Abstract A6):** Multivariate cox analysis of time to methotrexate discontinuation

	Hazard Ratio (95% CI)	*p*-value
Symptom duration >6 months	0.74 (0.46, 1.21)	0.24
Disease duration	0 .99 (0.99,1.00)	0.12
ANA positive	0.79 (0.47, 1.34)	0.38
Morning stiffness	1.17 (0.68, 1.99)	0.57
CHAQ	1.52 (0.94, 2.44)	0.09
Pain	0.92 (0.80, 1.06)	0.26
Patient global	0.65 (0.25, 1.72)	0.39
Physician global	0.87 (0.31, 2.42)	0.79
Active joint count	0.77 (0.31, 1.93)	0.58
JADAS	1.56 (0.60, 4.03)	0.36
Uveitis	1.54 (0.44, 5.44)	0.50
JIA Category		
Oligoarticular	Reference	NA
RF negative polyarticular	0.97 (0.54, 1.80)	0.96
RF positive polyarticular	0.89 (0.29, 2.74)	0.84
Enthesitis-related	1.56 (0.76, 3.23)	0.23
Psoriatic	1.41 (0.66, 3.01)	0.38
Systemic	1.10 (0.30, 4.03)	0.88
Undifferentiated	1.78 (0.23, 13.52)	0.58

*CHAQ* Childhood Health Assessment Questionnaire, *JADAS* juvenile arthritis disease activity score, *JIA* juvenile idiopathic arthritis

**Fig. 1 (Abstract A6):**
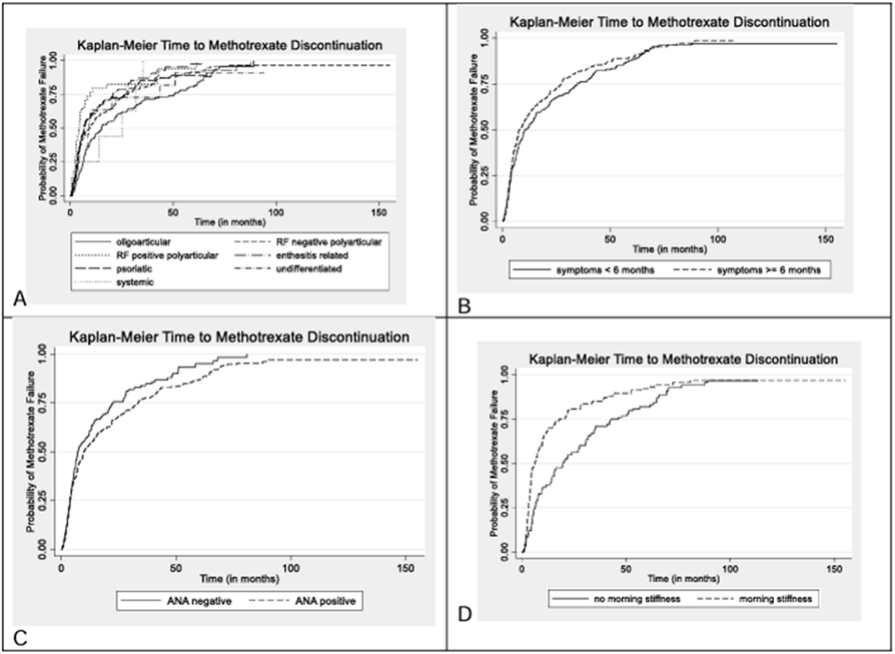
Kaplan-Meier Time to Methotrexate Discontinuation by Clinical Features. Kaplan-Meier curves for MTX discontinuation by **A** JIA subtype, **B** symptom duration more or less than 6 months, *p*=0.13, **C** ANA Status, *p*=0.03, **D** presence of morning stiffness over time, *p*<0.01

## A7 A Delphi study identifying essential areas of knowledge in pediatric rheumatology for the graduating pediatric resident

### Julia Shalen^1^, Emma Austenfeld^2^, McKenzie Vater^3^, Miriah Gillispie-Taylor^4^, For The CARRA Medical Education Working Group^5^

#### ^1^Johns Hopkins University, Baltimore, Maryland, USA; ^2^Medical College of Wisconsin, Milwaukee, WI, USA; ^3^Vanderbilt University, Nashville, TN, USA; ^4^Baylor University, Texas Children’s Hospital, Houston, TX, USA; ^5^Childhood Arthritis and Rheumatology Research Alliance (CARRA)

##### **Correspondence:** Julia Shalen

*Pediatric Rheumatology 2024*, **22(S1):**A7

Background : Childhood rheumatologic and musculoskeletal diseases (cRMDs) can cause significant morbidity and mortality, especially if not diagnosed and treated promptly. 33% of medical schools and 36% of pediatric residency programs have no affiliated pediatric rheumatologist, and many centers have a single provider covering a large catchment area. This can result in delayed patient care and inadequate clinical rheumatology education for pediatric trainees. Only 2% of the American Board of Pediatrics (ABP) content specifications pertain to cRMDs, which further decreases the likelihood that pediatric residents will allocate their studying time to these conditions. We believe that all trainees should graduate with practical knowledge about the presentation and initial management of cRMDs to optimize pediatric patient outcomes. The purpose of this study was to define essential knowledge areas in pediatric rheumatology for graduating pediatric residents.

Methods : We performed an extensive literature search of resources describing topics pertinent to pediatric rheumatology and considered potentially important for resident education. These findings were compiled into a survey that asked respondents, “How important is it for graduating pediatric residents to demonstrate competency in each of these rheumatology-related activities?” An expert focus group refined proposed items for the initial survey, and subsequent surveys were created iteratively based on the responses to prior surveys. Surveys were created in Qualtrics (Qualtrics, Provo, UT) and sent to invited participants between April 2023 and July 2023. Voting panel members were invited to participate in a modified Delphi process via email. Data was analyzed in Excel (Microsoft, Redmond, WA).

Results : Panelists were recruited as stakeholders in rheumatology-related pediatric resident education; the group represented diverse geographic and specialty backgrounds with a broad range of clinical experience. A total of 21 panelists completed two rounds of surveys with a 100% completion rate. Our analysis yielded a total of 107 items: those rated by >70% of respondents as “Very Important” were categorized as Essential (*n* = 52, 49.6%), and those rated by >70% of respondents as either “Very Important” or “Somewhat Important” categorized as Recommended (*n* = 55, 51.4%). The final list of items was organized into groups based on specific disease relevance and general principles of communication and patient management.

Conclusions : The current ABP content specifications are insufficient to prepare graduating pediatric residents to contend with cRMDs commonly encountered in the pediatric population. As we face a pediatric rheumatology workface shortage, having access to an expanded, standardized list of important rheumatology-related competencies might better prepare graduating pediatric residents to both serve patients with cRMDs and avoid unnecessary specialty referrals. The creation and distribution of these Delphi survey results with our shared opinion on the most appropriate pediatric rheumatology competencies will enhance critical education for the pediatric resident.

IRB Statement : This study was approved by the Johns Hopkins School of Medicine Institutional Review Board IRB00323040

Acknowledgements : On behalf of the CARRA Medical Education Workgroup. The authors wish to acknowledge CARRA and the ongoing Arthritis Foundation financial support of CARRA.

## A8 Temporomandibular joint involvement in pediatric patients with juvenile idiopathic arthritis: a single center retrospective study

### Paola Sparagana, Lynnette Walters, Heather Benham, Tracey Wright, Elizabeth Sloan, Julie Fuller

#### Texas Scottish Rite Hospital, Dallas, TX USA

##### **Correspondence:** Paola Sparagana

*Pediatric Rheumatology 2024*, **22(S1):**A8

Background : Involvement of the TMJ in JIA is a risk factor for poor prognosis due to its potential impact on mandibular growth, functional impairment, and associated comorbidities. Early diagnosis and treatment of TMJ arthritis is critical in preventing long-term complications. This study aims to evaluate the characteristics and therapeutic interventions of JIA patients with confirmed TMJ involvement.

Methods : In this retrospective cross-sectional study, data was abstracted from the electronic medical records of JIA patients with TMJ involvement confirmed by MRI from 2013 and 2023. Descriptive statistics were used to summarize patient characteristics, clinical features, radiographic findings, and therapeutic treatments.

Results : A total of 54 patients were included (see Table 1). The majority were female (87%), White (89%), and non-Hispanic (80%). The average age of JIA diagnosis was 7 years (SD ± 5.2), and the mean age at TMJ arthritis diagnosis was 12 years (SD ± 3.7). The most frequent JIA subtype was polyarticular (54%). ANA was positive in 65%, HLA-B27 in 9%, RF in 6%, and CCP in 6%. Thirty-six (67%) of the patients reported TMJ-related symptoms. Four of the symptomatic patients had an unremarkable clinical exam and were diagnosed based on MRI findings. Eighty percent experienced bilateral TMJ arthritis. On clinical exam, asymmetric mouth opening was the most common feature (54%), followed by reduced maximal mouth opening (43%), and mandibular growth disturbance (37%). Of the 54 patients, 41% had otherwise normal joint exams with no other active or limited joints indicating they had isolated TMJ arthritis. In initial MRI findings, 83% displayed active arthritis, 89% displayed chronic arthritis, and 72% displayed either active or inactive erosions. Recognition of active TMJ arthritis led to significant changes in management. Prior to TMJ involvement, most patients were on NSAIDs (63%), but less than half were treated with DMARDs (41%) or TNF inhibitors (39%). Only one patient was receiving oral steroids. Prior to TMJ involvement, most patients were on NSAIDs (63%), but less than half were treated with DMARDs (41%) or TNF inhibitors (39%). Only one patient was receiving oral steroids. Notably, there was significant increase in the use of NSAIDs (*p*=0.0019), steroids (*p*=0.0055), and TNF inhibitors (*p*< 0.0001) after TMJ involvement was recognized. Prior to their TMJ involvement, only half of polyarticular JIA patients were on TNF inhibition. This increased to 96% after diagnosis. Due to failed conservative treatment and advanced joint degeneration, 3 patients required bilateral TMJ replacement.

Conclusions : We observed TMJ arthritis in several subtypes of JIA with the majority of patients having bilateral involvement. Importantly, the findings demonstrate a significant increase in the utilization of NSAIDs, steroids, and TNF inhibition after the diagnosis of TMJ arthritis. At the time of TMJ arthritis diagnosis, the majority had evidence of chronic disease and erosions, suggesting a potential opportunity for earlier recognition of TMJ involvement. These results underscore the importance of timely recognition and appropriate management of TMJ involvement in JIA patients, aiming to optimize patient outcomes and quality of life.

IRB Statement : This study has been granted an exemption from requiring ethics approval by UT Southwestern IRB because this was a retrospective chart review of existing medical records. All data was recorded in an anonymous manner such that subjects cannot be identified directly or through identifiers linked to the subject.

**Table 1 (Abstract A8):** Demographic data and characteristics

	Total Cohort (***n*** = 54)	Systemic (***n***=2)	Oligoarticular (***n***=19)	Polyarticular (***n***=23)	Psoriatic (***n***=6)	Unclassified (***n***=4)
**Gender** n(%)						
Male	7 (13)	0 (0)	1 (5)	4 (18)	2 (33)	0 (0)
Female	47 (87)	2 (100)	18 (95)	19 (82)	4 (67)	4 (100)
**Race** n(%)						
African American or Black	1 (2)	0 (0)	1 (5)	0 (0)	0 (0)	0 (0)
Asian	3 (5)	0 (0)	1 (5)	0 (0)	1 (17)	1 (25)
White	48 (89)	2 (100)	17 (90)	21 (91)	5 (83)	3 (75)
Other	2 (4)	0 (0)	0 (0)	2 (9)	0 (0)	0 (0)
**Ethnicity** n(%)						
Hispanic or Latino	11 (20)	1 (50)	3 (16)	5 (22)	1 (17)	1 (25)
Non-Hispanic or Latino	43 (80)	1 (50)	16 (84)	18 (78)	5 (83)	3 (75)
**Age in years (mean ± SD)**						
At JIA diagnosis	7 ± 5.2	8 ± 7.1	5.8 ± 4.4	7.6 ± 5.4	4.2 ± 5.0	13.8 ± 1.3
At TMJ diagnosis	11.9 ± 3.8	14.5 ± 2.1	11.1 ± 3.3	12.0 ± 4.4	11 ± 3.1	15.3 ± 1.9
**ANA** n(%)						
Positive	35 (65)	0 (0)	14 (74)	15 (65)	3 (50)	3 (75)
Negative	17 (32)	1 (50)	5 (26)	7 (31)	3 (50)	1 (25)
Unknown	2 (4)	1 (50)	0 (0)	1 (4)	0 (0)	0 (0)
**Rheumatoid Factor** n(%)						
Positive	3 (6)	0 (0)	0 (0)	2 (9)	0 (0)	1 (25)
Negative	45 (83)	1 (50)	15 (79)	21 (91)	5 (83)	3 (75)
Unknown	6 (11)	1 (50)	4 (21)	0 (0)	1 (17)	0 (0)
**CCP** n(%)						
Positive	3 (6)	0 (0)	0 (0)	3 (14)	0 (0)	0 (0)
Negative	28 (52)	0 (0)	11 (58)	10 (43)	3 (50)	4 (100)
Unknown	23 (43)	2 (100)	8 (42)	10 (43)	3 (50)	0 (0)
**HLA-B27** n(%)						
Positive	5 (9)	0 (0)	2 (11)	1 (4)	1 (17)	1 (25)
Negative	33 (61)	0 (0)	10 (53)	15 (65)	5 (83)	3 (75)
Unknown	16 (30)	2 (100)	7 (37)	7 (30	0 (0)	0 (0)
**TMJ Laterality** n(%)						
Right	8 (15)	1 (50)	5 (26)	2 (9)	0 (0)	0 (0)
Left	3 (6)	0 (0)	1 (5)	1 (4)	1 (17)	0 (0)
Bilateral	43 (80)	1 (50)	13 (69)	20 (87)	5 (83)	4 (100)
**Positive MRI Findings** n(%)						
Active Arthritis^a^	45 (83)	2 (100)	17 (89)	18 (78)	5 (83)	3 (75)
Chronic Arthritis^b^	48 (89)	2 (100)	17 (89)	21 (91)	4 (67)	4 (100)
Erosions	39 (72)	1 (50)	15 (79)	16 (69)	5 (83)	2 (50)
**Positive Clinical Findings** n(%)						
Reduced MMO^c^	23 (43)	0 (0)	5 (26)	13 (57)	2 (33)	3 (75)
Mandibular Growth Disturbances^d^	20 (38)	0 (0)	7 (37)	9 (39)	2 (33)	2 (50)
Asymmetrical Mouth Opening	29 (54)	1 (50)	15 (79)	8 (35)	3 (50)	2 (50)

No ERA patients presented with TMJ arthritis in this cohort

*JIA* juvenile idiopathic arthritis, *TMJ* temporomandibular joint, *ANA* antinuclear antibody, *CCP* anti-citrillunated peptide, *MMO* maximal mouth opening

^a^Active Arthritis: synovial enhancement, fluid or thickening; pannus, bone marrow edema

^b^Chronic Arthritis: condylar flattening, disc abnormalities, heterotrophic bone formation

^c^Reduced maximal mouth opening (MMO) was defined as < 3 fingers able to fit in mouth

^d^Mandibular growth disturbances was defined as: mandibular asymmetry, micrognathia

## A9 Real-world treatment patterns and outcomes in patients with rheumatologic disease–associated hemophagocytic lymphohistiocytosis treated with emapalumab in the United States

### Carl Allen^1^, Edward Behrens^2^, Shanmuganathan Chandrakasan^3^, Michael Jordan^4^, Jennifer Leiding^5^, Abiola Oladapo^6^, Priti Pednekar^7^, Mona Riskalla^8^, Kelly Walkovich^9^, John Yee^6^

#### ^1^Division of Pediatric Hematology and Oncology, Baylor College of Medicine, Houston, Texas, USA; ^2^Children’s Hospital of Philadelphia, Philadelphia, Pennsylvania, USA; ^3^Division of Bone and Marrow Transplant Research, Aflac Cancer and Blood Disorders Center, Children’s Healthcare of Atlanta, Emory University, Atlanta, Georgia, USA; ^4^Division of Bone Marrow Transplantation and Immune Deficiency, Cincinnati Children’s Hospital Medical Center, Cincinnati, Ohio, USA; Department of Pediatrics, University of Cincinnati College of Medicine, Cincinnati, Ohio, USA; ^5^Division of Allergy and Immunology, Department of Pediatrics, Johns Hopkins University, Baltimore, Maryland, USA; Bluebird Bio, Cambridge, Massachusetts, USA; ^6^Sobi, Inc. Waltham, Massachusetts, USA; ^7^PRECISIONheor, Bethesda, Maryland, USA; ^8^University of Minnesota Medical Center, Minneapolis, Minnesota, USA; ^9^Division of Pediatric Hematology Oncology, Department of Pediatrics, University of Michigan Medical School, Ann Arbor, Michigan, USA

##### **Correspondence:** Edward Behrens

*Pediatric Rheumatology 2024*, **22(S1):**A9

Background : Secondary hemophagocytic lymphohistiocytosis (sHLH), a rare, life-threatening, hyperinflammatory syndrome, occurs in the context of an underlying disease (rheumatologic, malignancy, etc) and/or infectious trigger. sHLH is caused by the overproduction of proinflammatory cytokines, eg, interferon gamma (IFNγ). The REAL-HLH study assessed clinical and demographic characteristics and real-world treatment patterns and outcomes in patients with hemophagocytic lymphohistiocytosis (HLH) treated with emapalumab, a fully human anti-IFNγ monoclonal antibody indicated for adults and children with primary HLH with refractory, recurrent, or progressive disease, or intolerance to conventional HLH therapy.

Methods : A retrospective medical chart review across 33 US hospitals identified patients treated with ≥1 dose of emapalumab between November 20, 2018, and October 31, 2021. Data extracted on the subset of patients with rheumatologic disease-associated HLH (sHLH/macrophage activation syndrome [MAS]) from time of emapalumab initiation to end of data availability, death, or study end (December 31, 2021) are presented.

Results : Fifteen of 105 (14.3%) study patients had HLH associated with an underlying rheumatologic disease; most (10/15; 66.7%) had Still’s disease (systemic juvenile idiopathic arthritis [sJIA] = 9; adult-onset Still’s disease [AOSD] = 1). Median (range) age at HLH diagnosis was 5 (0.9–39) years, and most patients (9/15; 60.0%) initiated emapalumab treatment in an intensive care unit (Table 1). Emapalumab was most frequently initiated for the treatment of refractory (5/15; 33.3%), recurrent (5/15; 33.3%), and progressive (4/15; 26.7%) disease. Most patients received HLH-related therapies prior to (10/15; 66.7%) and concurrently (15/15; 100.0%) with emapalumab, mostly corticosteroids (15/15; 100%) and anakinra (9/15; 60.0%). Median (range) time from HLH diagnosis to emapalumab initiation and overall treatment duration was 21.0 (1–988) and 63 (3–397) days, respectively. Median (range) emapalumab starting and cumulative treatment doses were 3.1 (0.8–5.9) and 29.7 (4.0–223.6) mg/kg, respectively (Figure 1). Following treatment with emapalumab-containing regimens, key laboratory parameters were normalized in most patients (Table 2). Overall survival and 12-month survival probability from emapalumab initiation was 86.7%. There were 2 deaths; 1 patient had rheumatoid arthritis and was deemed unresponsive to treatment, whereas the other with sJIA died due to uncontrolled disseminated adenoviremia, unrelated to the clinical condition for which emapalumab was used (investigator-determined).

Conclusions : This study reported real-world treatment patterns and outcomes with emapalumab across a diverse patient population with rheumatologic disease–associated HLH. Following treatment with emapalumab-containing regimens, most laboratory parameters were normalized, and the overall survival rate was 86.7%. A pivotal clinical trial of emapalumab in patients with sHLH and underlying rheumatologic disease is ongoing (NCT05001737).

IRB Statement : The protocol and data collection forms were reviewed by the Western Institutional Review-Copernicus Group Institutional Review Board (WCG IRB) and local IRBs, where required. There was no direct patient involvement in the study and anonymized data collection was conducted in compliance with US Health Insurance Portability and Accountability Act (HIPAA) policies.

Acknowledgements : The authors thank Sajjad Raza and Alice Pressman (PRECISIONheor) and Corey Best (Sobi, Inc.) for their support. Editorial and medical writing support was provided by Aparna Nori, PhD, CMPP, of rareLife solutions, Westport, Connecticut, USA, and funded by Sobi, Inc.

**Table 1 (Abstract A9):** Demographics and clinical characteristics of patients with rheumatologic disease–associated HLH (*N* = 15)^a^ and the subset of patients with Still’s disease (*n* = 10)

Parameter	Patients with rheumatologic disease–associated HLH (*N* = 15)	
	All patients (*N* = 15)	Still’s disease^b^ (*N* = 10)^c^
Age at HLH diagnosis, median (range), years	5.0 (0.9–39)	2.5 (1.0–22.0)^d^
Female, n/N (%)	11/15 (73.3)	8/10 (80.0)
Race (White), n/N (%)	7/15 (46.7)	6/10 (60.0)
Infection at diagnosis, n/N (%)	5/15 (33.3)	3/10 (30.0)
Virus	4/5 (80.0)	3/3 (100.0)
Bacteria	1/5 (20.)	0/10 (0.0)
Fungi	0/15 (0.0)	0/10 (0.0)
CNS involvement at diagnosis, n/N (%)	1/15 (6.7)	0/10 (0.0)
Age at initiation of emapalumab, median (range), years	5.0 (0.9–39)	2.5 (1.0–22.0)
Initiated emapalumab in an ICU, n/N (%)	9/15 (60.0)	6/10 (60.0)
Received supportive care at initiation of emapalumab, n/N (%)	7/15 (46.7)	6/10 (60.0)
Ventilator	3/7 (42.9)	3/6 (50.0)
Dialysis	1/7 (14.3)	1/6 (16.7)
Pressors	0/7 (0.0)	0/6 (0.0)
Extracorporeal membrane oxygenation	0/7 (0.0)	0/6 (0.0)
Other^e^	4/7 (57.1)	3/6 (50.0)

*CNS* central nervous system, *HLH* hemophagocytic lymphohistiocytosis, *ICU* intensive care unit, *n* number of patients with event of interest, *N* number of patients with available data

^a^Still’s disease (*n* = 10), rheumatoid arthritis (*n* = 1), systemic lupus erythematous (*n* = 1), juvenile idiopathic arthritis (*n* = 1), diffuse myositis (*n* = 1), *NLRC4* mutation (*n* = 1)

^b^Adult-onset Still’s disease (AOSD) and systemic juvenile idiopathic arthritis (sJIA)

^c^Patients with Still’s disease was a subset of the group of patients with rheumatologic disease–associated HLH

^d^The patient with AOSD was 22 years of age at diagnosis

^e^High-flow nasal cannula; tunneled percutaneous catheter; nasogastric/orogastric tube insertion; bilevel positive airway pressure (BiPAP); continuous positive airway pressure (CPAP)

**Fig. 1 (Abstract A9):**
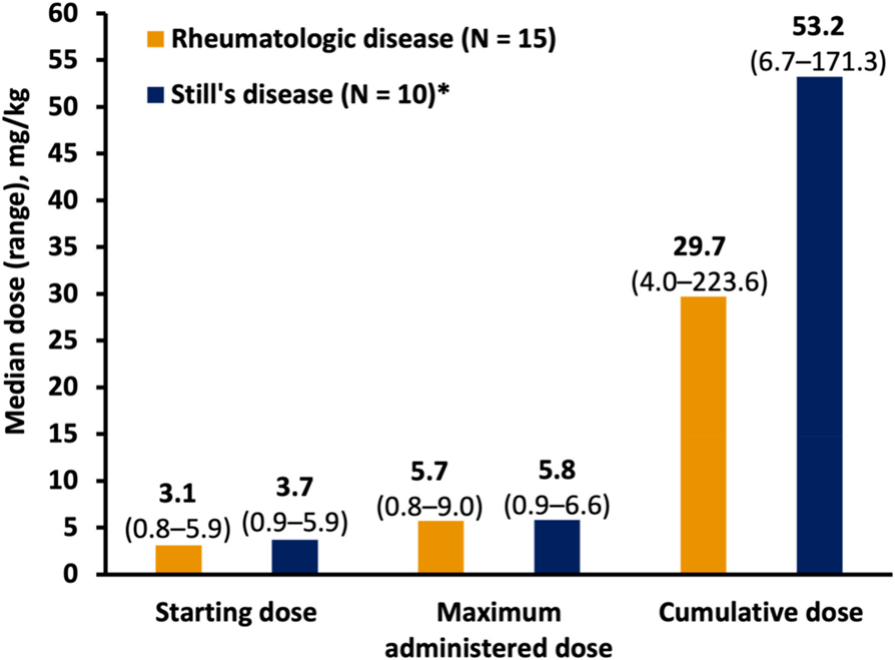
Emapalumab dosing in patients with rheumatologic disease–associated HLH (*N* = 15) and the subset of patients with Still’s disease (*N* = 10)

**Table 2 (Abstract A9):** Physician-assessed normalization of laboratory values and median time to first normalization in patients with rheumatologic disease–associated HLH (*N* = 15) and the subset of patients with Still’s disease (*N* = 10)

Parameter	Patients with rheumatologic disease–associated HLH (***N*** = 15)			
	All patients (***N*** = 15)		Patients with Still’s disease (***N*** = 10)^**c**^	
	Proportion of patients, n/N (%)^a,b^	Median (range) time to normalization, days^a^	Proportion of patients, n/N (%)^a,b^	Median (range) time to normalization, days^a^
Absolute neutrophil count	13/14 (92.9)	7.0 (1.0–40.0)	9/10 (90.0)	7.0 (3.0–40).0)
Absolute lymphocyte count	13/14 (92.9)	7.5 (1.0–28.0)	9/10 (90.0)	7.0 (3.0–28.0)
CXCL9	9/11 (81.8)	32.0 (5.0–129.0)	6/8 (75.0)	32.0 (5.0–39.0)
Alanine transaminase	11/14 (78.6)	14.0 (4.0–108.0)	8/10 (80.0)	14.0 (4.0–108.0)
Platelet count	11/14 (78.6)	14.0 (1.0–34.0)	8/10 (80.0)	10.5 (1.0–26.0)
Fibrinogen	9/13 (69.2)	14.0 (1.0–26.0)	6/10 (60.0)	15.5 (1.0–26.0)
sCD25	6/9 (66.7)	30.5 (3.0–153.0)	4/6 (66.7)	51 (29.0–153.0)
Ferritin	6/14 (42.9)	38.5 (15.0–106.0)	5/10 (50.0)	46.0 (15.0–106.0)

*CXCL9* chemokine ligand 9, *HLH* hemophagocytic lymphohistiocytosis, *n* number of patients who achieved normalization on each laboratory parameter, *N* number of patients with available data on each laboratory parameter, *sCD25* soluble interleukin-2 receptor α

^a^Based on physician’s report

^b^Proportion of patients who achieved normal values for each laboratory parameter at any time during emapalumab treatment

^c^Patients with Still’s disease was a subset of the group of patients with rheumatologic disease–associated HLH

## A10 Patient and family perceptions of real time access to electronic health information: a social media survey

### Caitlan Pinotti^1^, Rajdeep Pooni^2^, Vincent Del Gaizo^3^, Melanie Kohlheim^3^, Emily Schildt^4^, Alysha Taxter^5^, Tova Ronis^6^, for the CARRA Clinical Informatics Workgroup^3^

#### ^1^Duke University, Durham, NC USA; ^2^Stanford Children’s Health, Stanford University School of Medicine; ^3^Childhood Arthritis and Rheumatology Research Alliance (CARRA); ^4^Seattle Children’s Hospital, Seattle, WA, USA; ^5^Nationwide Children’s Hospital, Columbus, OH, USA; ^6^Children’s National Hospital, George Washington University

##### **Correspondence:** Caitlan Pinotti

*Pediatric Rheumatology 2024*, **22(S1):**A10

Background : As of April 2021, the 21st Century Cures Act requires that patients have immediate access to their electronic health information (EHI) (https://www.healthit.gov/topic/oncs-cures-act-final-rule). This study seeks to understand pediatric rheumatology patients’ and caregivers’ perceptions of the increased data accessibility.

Methods : An anonymous, 23-question Qualtrics survey was distributed via social media to patients and families with pediatric rheumatic diseases by patient partners. The survey link and QR code were posted on disease-specific and personal social media accounts, primarily Facebook. The survey was available for 10 days and included participant attestation of a confirmed diagnosis by a pediatric rheumatologist. Descriptive statistics and a two-tailed t-test were used in analysis, including for those questions where a 5-point Likert scale was used (1 = strongly disagree to 5 = strongly agree). A *p*-value < 0.05 was considered significant.

Results : The survey received 325 initial responses; 23 responses were incomplete, 34 listed countries of residence outside the United States, 14 did not or were unsure if they had EHI access, and 1 participant did not confirm a diagnosis by a pediatric rheumatologist. Table 1 contains demographics and diagnoses of the remaining 253 eligible responses. Nearly 48% of participants reported accessing their electronic patient portal > 12 times in the last year and only 8% reported accessing it 1-2 times. Forty-five percent reported accessing their portal the same day as their appointment, 36% only when they get an alert for new results, and 0.8% only when a healthcare provider messages. Ninety-eight percent use the patient portal to access lab results, 80% healthcare provider messages, 64% imaging results, 53% appointments, and 28% medications. Free-text questions solicited respondent likes/dislikes of increased access to EHI, including “It’s data about ME; it is only fitting that I should know it.” Table 2 and Figure 1 contain additional summary data for survey questions using 4 or 5-point Likert scales. There were no significant differences in responses by diagnosis (JIA versus systemic connective tissue disease), however, respondents diagnosed ≤ 5 years ago were more likely to prefer immediate access to EHI, rather than waiting for a provider.

Conclusions : Respondents reported overwhelmingly positive feedback regarding immediate access to EHI through the patient portal. Though some did report that electronic access to labs increases their worry, families largely expect access to this data. This study is limited by the fact that it targeted an already engaged, English-speaking population with digital literacy, access to their patient portal, and patient engagement in disease-specific social media sites.

IRB Statement : This study was granted exemption by Duke’s Internal Review Board (Pro00113906).

Acknowledgements : The authors would like to thank the patients and families who participated in this study and completed the survey. They also wish to acknowledge CARRA and the ongoing Arthritis Foundation financial support of CARRA.

**Table 1 (Abstract A10):** Demographics and diagnoses of eligible respondents with completed surveys. *N* = 253

	Count (%)
Respondent	
*Patient*	12 (5)
*Parent/Guardian*	241 (95)
Patient age 12 years+	117 (46)
Primary language	
*English*	252 (99.6)
*Spanish*	1 (0.4)
Race	
*Asian*	3 (1.2)
*Black, African American, African, or Afro-Caribbean*	2 (0.8)
*Hispanic, Latino, or Spanish Origin*	6 (2)
*Native American, American Indian, or Alaskan Native*	1 (0.4)
*White*	204 (81)
*Mixed race*	33 (13)
*Prefer not to answer*	4 (1.6)
Diagnosis	
*Juvenile idiopathic arthritis (JIA), including those with hypermobility or amplified musculoskeletal pain*	94 (37)
*Systemic connective tissue disease*^a^	159 (63)
Years since diagnosis, median [IQR]	4 [2,8]

^a^includes systemic lupus erythematosus, mixed connective tissue disease, Sjogren syndrome, inflammatory myositis, vasculitis, scleroderma, autoinflammatory syndromes (including periodic fever syndromes), and chronic noninfectious osteomyelitis

**Table 2 (Abstract A10):** Survey responses analyzed by number of years since diagnosis. Agreement level measured on a 5-point Likert scale where strongly disagree = 1 and strongly agree = 5. Mean (standard deviation). *p-value < 0.05

Question	Years Since Diagnosis ≤ 5 (*n* = 153)	Years Since Diagnosis > 5 (*n*=83)	*p*-value
I like having electronic access to my child’s results as soon as they are available, before discussing with my child’s healthcare provider	4.80 (0.54)	4.81 (0.65)	0.90
I would prefer that my child’s results only get released after my child’s healthcare provider has discussed them with me.	1.59 (1.03)	1.89 (1.15)	0.04*
I have felt worried about how to understand my child’s test results when accessing them electronically.	2.62 (1.37)	2.39 (1.26)	0.21

**Fig. 1 (Abstract A10):**
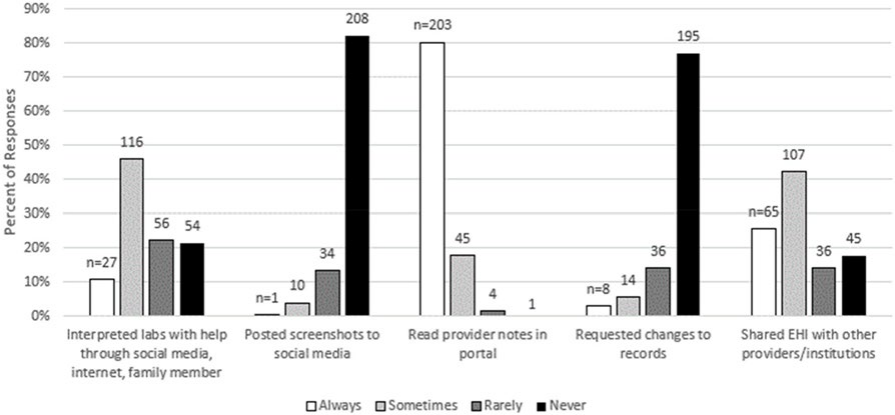
Percentage rates of response on a 4-point Likert scale (always, sometimes, rarely, never) regarding respondent uses of the patient portal and electronic health information

## A11 FACE-Q outcomes in craniofacial scleroderma: a pilot study on the functional and psychosocial impact of disease in children and young adults

### Tyler Nguyen^1^, Alex Cappitelli^1^, Laura Nuzzi^1^, Lisa Nussbaum^1^, Katharina Shaw^2^, Olivia Langa^1^, Fatma Dedeoglu^1^, Ruth Ann Vleugels^3^, Ingrid Ganske^1^

#### ^1^Boston Children’s Hospital, Boston, MA USA; ^2^Children’s Hospital of Philadelphia, Philadelphia, PA USA; ^3^Brigham and Women’s Hospital, Boston, MA USA

##### **Correspondence:** Ingrid Ganske

*Pediatric Rheumatology 2024*, **22(S1):**A11

Background : Craniofacial Scleroderma (CS) is a rare autoimmune disorder characterized by progressive atrophy of the skin and underlying soft tissue. Changes in facial asymmetry as a result of disease activity have aesthetic and functional ramifications that can affect quality of life. This study assesses the functional and psychosocial impacts of CS in children and adolescents through Patient Reported Outcomes (PROs).

Methods : This is a prospective study of PROs completed by children and adolescents with clinically stable CS during intake and follow-up visits between 2019-2023. Patients completed 10 functional and psychosocial domains of the validated FACE-Q Craniofacial Module (1) along with the capture of 3-dimensional (3D) photographs to document disease status. FACE-Q scores were analyzed to 1) calculate median scores for each domain, 2) evaluate differences between younger (7-14 years) and older (15+ years) age groups, 3) compare our cohort to the FACE-Q validation cohort (1), 4) document frequency/types of adverse effects, 5) measure variation in domain scores for individuals completing multiple surveys, 6) perform regression analyses between psychosocial domains, and 7) compare PROs based on severity of disease.

Results : FACE-Q surveys from 24 patients (aged 7-23 years; 18 females) were collected. Median scores were highest in Jaw Appearance (96.0) and lowest in Appearance Distress (50.0). Younger patients (*n*=12) reported significantly lower median scores in four psychosocial domains (*p*< 0.05, all). Significant differences in five domains (*p*< 0.05, all) were identified between our cohort and the validation group. Adverse effects were reported in 21 patients (87.5%), most commonly identified as firmness of the face (*n*=16). Patients completing surveys at 3+ time points (*n*=9) showed slight improvement in scores across all domains over time. Regression analyses found Facial Appearance domain scores predictive of scores in the Psychological domain (R2 =0.623, *p*< 0.001) but not in the Appearance Distress domain (R2=0.0711, p=0.208). On average, region-specific scores were similar for Minor and Moderate atrophy, but worse in severe disease which had the lowest average scores across all domains.

Conclusions : FACE-Q outcomes in patients with CS indicate a complex interaction of facial appearance satisfaction, scholastic and social quality of life, supporting the need for psychosocial support. Further studies may be useful in assessing satisfaction following treatment, including reconstructive procedures. 1. Klassen AF, Rae C, Wong Riff KW, et al. FACE-Q Craniofacial Module: Part 1 validation of CLEFT-Q scales for use in children and young adults with facial conditions. J Plast Reconstr Aesthetic Surg JPRAS. 2021;74(9):2319-2329. doi:10.1016/j.bjps.2021.05.040

IRB Statement : This study was approved by the Institutional Review Board at Boston Children’s Hospital (IRB-P00029401), and all research activities were conducted in accordance with the Declaration of Helsinki.

## A12 Type I interferon signatures can be accurately measured by serum LAMP3 expression in juvenile-onset SLE and are associated with cardiovascular risk

### Junjie Peng, Saara Atif, Elizabeth Jury, Coziana Ciurtin, George Robinson

#### University College London

##### **Correspondence:** George Robinson

*Pediatric Rheumatology 2024*, **22(S1):**A12

Background : Patients with juvenile-onset systemic lupus erythematosus (JSLE, onset < 18 years) have more severe disease and relatively higher cardiovascular disease (CVD) and mortality risk compared to adult-onset patients. This could be associated with more predominant type I interferon (IFN) signalling. We investigated a more efficient method to assess the heterogeneity of type I IFN signatures in JSLE patients and their relationship with CVD using multi-omics.

Methods : RNA-sequencing (UCL-Genomics) was used to assess differentially expressed genes (DEGs) in peripheral blood mononuclear cells between JSLE patients with inactive disease (*n*=29, mean age=19) and healthy controls (HCs, *n*=8, mean age=18). Data was analysed by gene ontology pathway enrichment, weighted gene co-expression network analysis (WGCNA), hierarchical clustering, and receiver operating characteristic (ROC) analysis. BST2 expression was quantified by spectral flow cytometry on peripheral blood mononuclear cell (PBMC) subsets. Proteomics (Olink) and Metabolomics (Nightingale) assessed serum proteins (detection validated by ELISA) and metabolites associated with inflammation and CVD in matched patients. Patient clinical data was analysed by trajectory analysis with mean follow up of 4.9 years and 17 clinical encounters.

Results : JSLE patients had significantly enriched type I IFN signalling pathways compared to HCs (*p*< 0.0001), despite inactive disease, validated by unbiased WGCNA, where type I IFN signalling was the most significantly upregulated network module in JSLE patients, followed by T-cell activation and cytotoxicity modules. Despite the pathway dominance of type I IFN signalling, patients clustered heterogeneously into a high (H-IFN, 66%, ROC: *p*< 0.0001, AUC=1.00 vs HCs) and low (L-IFN, 34%, ROC: *p*=0.53, AUC=0.59 vs HCs) transcriptomic type I IFN signature group using normalised gene counts, validated by IFN-scores (*p*< 0.0001). 281 DEGs were upregulated in the H-IFN (vs L-IFN) group, with notable enrichment of cell cycle pathways. Serum protein levels of LAMP3 correlated most significantly (*p*=0.0018, *r*=0.67) with transcriptomic IFN-scores in patients (ROC: *p*=0.0069, AUC=0.95 for H-IFN vs L-IFN) from a panel of >280 proteins, highlighting a novel biomarker of H-IFN with translational potential for patient stratification; this was supported by fast and cost-effective biomarker quantification/validation by ELISA. This biomarker accuracy was not replicated by the IFN-induced cell surface marker, BST2, supporting serum protein quantification. No difference in clinical serology or disease activity trajectory was observed between type I IFN groups. However, transcriptomic IFN-scores correlated positively with proteomic (including ICAM1/VCAM1) and metabolic (including glycoprotein acetyls and ApoB:ApoA1 ratios) biomarkers known to reflect CVD-risk.

Conclusions : JSLE patients can be stratified by type I IFN signatures associated with CVD-risk and upregulated cell cycle pathways even in low disease activity states. Serum LAMP3 measurement could be used as a cost-effective method to stratify patients for therapeutic intervention to improve outcomes.

IRB Statement : This study was approved by the London-Harrow Research Ethics Committee, reference 11/LO/0330. Written informed consent was acquired from patients and healthy controls.

## A13 Development and usability testing of web-based standardized scoring tool for magnetic resonance images from children with chronic nonbacterial osteomyelitis (CNO)

### Farzana Nuruzzaman^1^, T. Shawn Sato^2^, Andrew Carbert^3^, Joel Paschke^3^, Lauren Potts^4^, Meinrad Beer^5^, Mingqian Huang^6^, Ramesh Iyer^7^, Johanna Monsalve^8^, Anh-Vo Ngo^7^, Jennifer Stimec^9^, Mahesh Thapa^7^, Xiaoyue Zhang^10^, Walter P. Maksymowych^3^, Polly Ferguson^2^, Yongdong Zhao^11^ for the CARRA CRMO/CNO Workgroup^12^

#### ^1^Stony Brook Children’s Hospital, Stony Brook, NY, USA; ^2^University of Iowa; ^3^CARE Arthritis; ^4^Long Beach, CA; ^5^University Hospital, Ulm Germany; ^6^Mount Sinai Hospital; ^7^University of Washington; ^8^SUNY-Stony Brook Hospital; ^9^Sick Kids Children’s Hospital; ^10^Biostatistical Consulting Core - Renaissance School of Medicine at Stony Brook University; ^11^Seattle Children’s Hospital; ^12^Childhood Arthritis and Rheumatology Research Alliance (CARRA)

##### **Correspondence:** Farzana Nuruzzaman

*Pediatric Rheumatology 2024*, **22(S1):**A13

Background : The ChRonic nonbacterial Osteomyelitis Magnetic Resonance Imaging Scoring (CROMRIS) tool was developed to assess specific characteristics of bone and soft tissue inflammation in MRI of patients with CNO, but was labor intensive to utilize. Primary objectives of this study are: 1) to adapt the CROMRIS tool to a web-based platform and 2) assess the usability of this web-based CROMRIS system among radiologists.

Methods : A prototype web-based CROMRIS tool limited to the arms and legs was developed by CARRA’s CRMO Workgroup and CARE-Arthritis in 2019. Monthly meetings between software developers, rheumatologists, radiologists and an illustrator led to a beta version that included the whole body. A purposive sample of radiologists (*n*=7) provided feedback on the beta version in a demo session on 4/11/22 via semi-structured surveys (Stony Brook University #IRB2021-00033). Usability was assessed in 5/2022 and in 12/2022 using the System Usability Scale (SUS), a Likert scale in which respondents indicate their level of agreement or disagreement on a scale of 1 to 5 for 10 statements on ease of use, effectiveness, and satisfaction in content of use. Feedback was reported with descriptive content analysis, continuous variables as means and categorical variables as percentages.

Results : A clickable-schematic-based CROMRIS was developed to include all body regions: head (skull/mandible), spine, torso (clavicle, sternum, ribs), pelvis, hands, feet, arms and legs [Figure 1]. Notable features are the ability to immediately highlight a schematic region upon selection to directly input scores. Suggested changes included labeling of individual spine and rib segments, insertion of scoring legend on each tab for reference and creation of a summary page with a composite diagram where one can visualize the location and size of lesions by color as well as a numerical CROMRIS activity index [Figure 2]. The general scoring on the portal is indicated by clicking on a bone unit to indicate the presence of bone marrow hyperintensity consistent with an active CNO lesion, which is qualified by other lesion parameters [Figure 3]. A video tutorial and MRI Atlas is on the platform for training (https://www.carearthritis.com/mriportal/crmo/index/). Visual factors and anatomical diagrams were among the features “liked best” by the survey respondents. Mean SUS scores increased from 64.5 (below average) to 75 (above average). All respondents agreed that the final version of the web-based CROMRIS was “easy to learn” and found that the “various functions of the web-based CROMRIS were well integrated, and “would like to use the web-based CROMRIS in future clinical trials.”

Conclusions : The web-based CROMRIS portal shows good usability amongst radiologists. Studies of interrater reliability among experienced pediatric radiologists are underway. Once validated, this tool can be used as a semi-quantitative MRI scoring tool to allow for standardization of reporting output of radiological interpretations of MRI in CNO.

IRB Statement : IRB approval Stony Brook University #IRB2021-00033).

Acknowledgements : This project is supported by a CARRA-Arthritis Foundation Small Grant. The authors wish to acknowledge the ongoing Arthritis Foundation financial support of CARRA.

**Fig. 1 (Abstract A13):**
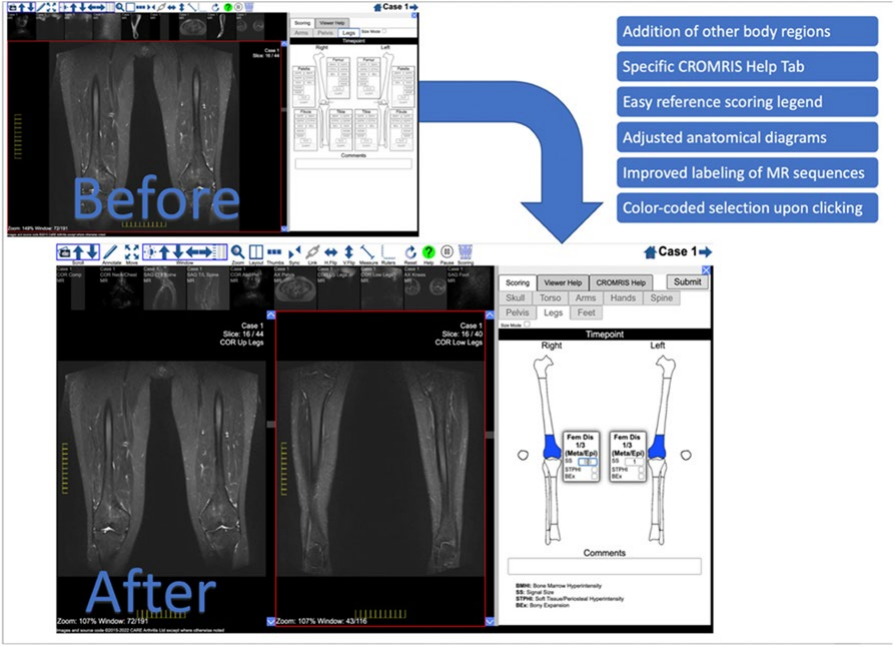
Screenshot of Web-based CROMRIS Portal on Tab for Long Bones of Lower Extremity Before and After Feedback Sessions/Usability Testing

**Fig. 2 (Abstract A13):**
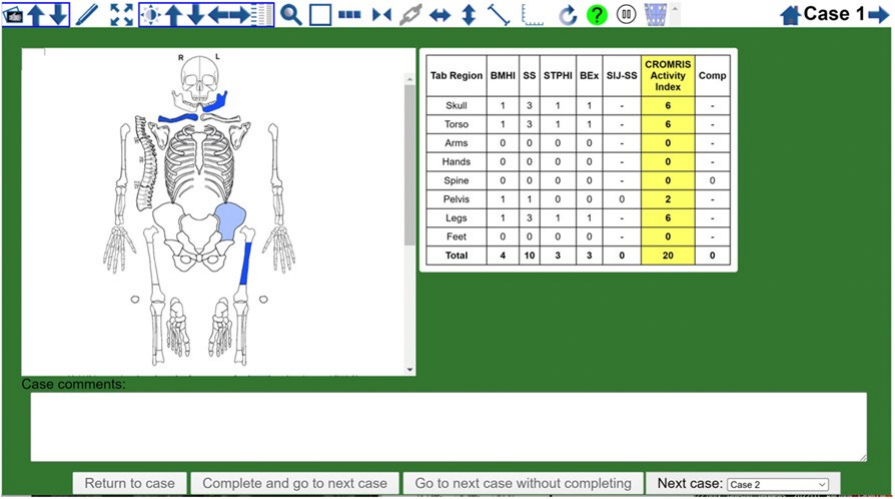
Screenshot of Summary Page for Final Web-based CROMRIS Portal

**Fig. 3 (Abstract A13):**
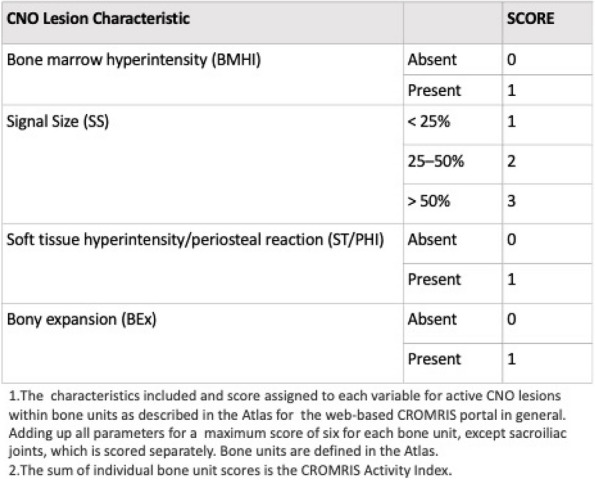
Scoring of Active CNO Lesions in General Bone Units in the Web-Based CROMRIS

## A14 Prospective value of brain structural MRI metrics to aid the diagnosis and clinical evaluation of systemic lupus erythematosus in children

### Diana Valdes Cabrera^1^, Santiago Arciniegas^1^, Matthias Wagner^2^, Birgit Ertl-Wagner^1^, Tala El Tal^1^, Asha Jeyanathan^1^, Lawrence Ng^1^, Deborah Levy^1^, Linda Hiraki^1^, Andrea Knight^3^

#### ^1^The Hospital for Sick Children, Toronto, Ontario Canada; ^2^The Hospital for Sick Children, Toronto, Ontario, Canada; University Hospital Augsburg, Germany; ^3^The Hospital for Sick Children and SickKids Research Institute, Toronto, Ontario, Canada

##### **Correspondence:** Diana Valdes Cabrera

*Pediatric Rheumatology 2024*, **22(S1):**A14

Background : Brain tissue atrophy and ventricular enlargement are characteristic imaging biomarkers of systemic lupus erythematosus (SLE) that have been observed in children (cSLE) with and without a neuropsychiatric (NPSLE) diagnosis. However, these brain features are not commonly quantified in conventional Magnetic Resonance Imaging (MRI) evaluations of cSLE. Brain volumes obtained from clinical control data can facilitate baseline and longitudinal evaluations of cSLE populations; yet, neuroimaging work is limited by small cSLE cohorts. This study aimed to leverage the value of structural MRI data obtained in clinical settings over a decade and to investigate the impact of brain volumes on the diagnosis and evaluation of cSLE.

Methods : T1-weighted brain MRI data were acquired from retrospective clinical samples of 133 patients with cSLE (4-18 years) and 132 controls that were scanned from 2010 to 2021 on 1.5T or 3T scanners (Table 1). Structural metrics included total grey matter (GM) cortex, subcortical GM, white matter (WM), and lateral ventricles volumes, mean cortical thickness and WM surface area (Figure 1A). Group differences between NPSLE and non-NPSLE subgroups and controls were examined with analysis of covariance (ANCOVA), including age, sex, and total intracranial volume as covariates. Age associations and age and group (cSLE versus controls) interaction effects were explored with linear regression in brain metrics that displayed group differences. Cortical GM volume/thickness and lateral ventricles volumes were tested as predictors of group classification in cSLE patients and healthy controls with logistic regression and receiver operating characteristic analyses.

Results : ANCOVA group analyses revealed lower cortical volume (F=32.9), thinner cortex (F=27.4), and greater lateral ventricles volumes (F=21.0) in both NPSLE and non-NPSLE groups compared to healthy controls (*p*< 0.001), but no differences within cSLE subgroups or between NPSLE subgroup and controls for WM surface area (Figure 1B-E). Regression analyses showed an interaction effect between age and group with GM thickness that was driven by the cSLE group (t=-3.04, *p*=0.003; Figure 1D). Together, lower cortical tissue volume and higher lateral ventricle volume predicted cSLE diagnosis with a sensitivity of 75.9% and specificity of 73.5% when compared to healthy controls (Figure 2).

Conclusions : Cortical atrophy and thinning, and enlarged lateral ventricles were frequently observed in patients with cSLE. Cortical thinning at a faster rate in cSLE than in healthy controls indicated changes in GM morphology that are specific to this pathology. These structural MRI metrics may aid in assessing early brain atrophy and in evaluating the magnitude of these deviations with age in cSLE when compared to neuroimaging brain data from age and sex comparable healthy populations. Additionally, evaluations of cSLE against healthy brain MRI data might increase diagnostic precision in cSLE, even when retrospectively acquired.

IRB Statement : I certify that this study has received the appropriate IRB approval by an appropriate ethics committee (The Research Ethics Board established by the SickKids Board of Directors). The IRB number is 1000079127 and the official title is “Identifying Neuroimaging Biomarkers of Neuropsychiatric Childhood-Onset Systemic Lupus Erythematosus Artificial Intelligence and Machine Learning”.

**Table 1 (Abstract A14):** Demographic and clinical characteristics of non-NPSLE, NPSLE, and healthy control groups

*Group (% SLE)*	*Age in Years (Mean ± SD)*	*Sex (% Female)*	*Time since Diagnosis (Months)*	*SLEDAI* ^a^ *(Activity)*	*Prednisone Exposure (Current/Past)*	*Current Prednisone Dose (mg)*
Non-NPSLE (82/133, 62%)	14.40 ± 2.7	68/82 (83%)	14.7 ± 32.3	6.5 ± 6.8	41/82 (50%)	14.4 ± 21.2
NPSLE (51/133, 38%)	14.10 ± 2.8	41/51 (80%)	10.9 ± 21.1	11.5 ± 9.0^*^	34/51 (67%)	24.5 ± 25.6^*^
Controls (HC)	14.48 ± 2.7	108/132 (82%)	-	-	-	-

^*^p<0.05, independent samples T-tests

^a^SLE Disease Activity Index

**Fig. 1 (Abstract A14):**
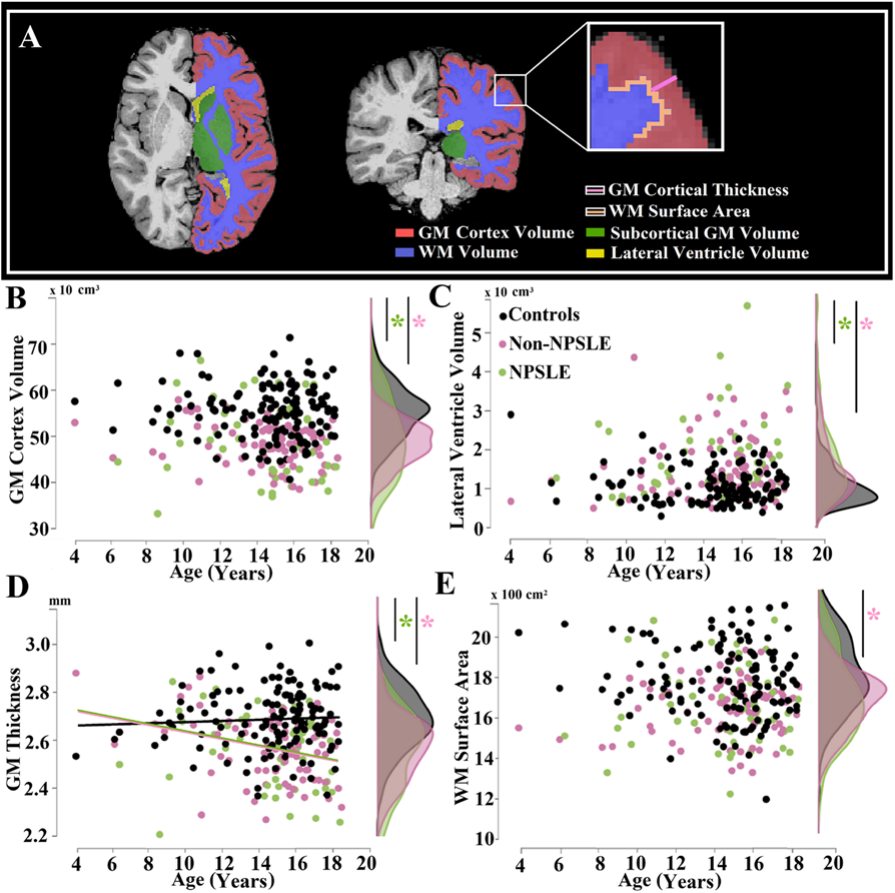
Brain structural MRI metrics (**A**) that showed significant abnormalities in NPSLE and non-NPSLE subgroups compared to a healthy control cohort (**B**-**E** *p<0.001)

**Fig. 2 (Abstract A14):**
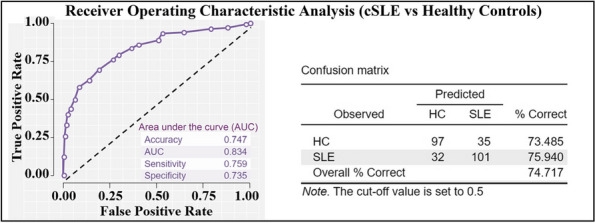
Receiver operating characteristic analysis revealed that cortical and lateral ventricles volumes can aid with cSLE classification with an overall accuracy of 74.75%

## A15 Validation of an electronic health record algorithm to identify children with chronic rheumatic disease

### Alysha Taxter^1^, Matthew Basiaga^2^, Rajdeep Pooni^3^, Caitlan Pinotti^4^, Lisa Buckley^5^

#### ^1^Nationwide Children’s Hospital, Columbus, OH, USA; ^2^Mayo Clinic; ^3^Stanford Children’s Health, Stanford University School of Medicine; ^4^Duke University, Durham, NC USA; ^5^Monroe Carell Jr. Children’s Hospital at Vanderbilt, Nashville, TN, USA

##### **Correspondence:** Alysha Taxter

*Pediatric Rheumatology 2024*, **22(S1):**A15

Background : Many study teams spend hours reviewing the electronic health record (EHR) to identify possible research subjects. The EHR is a rich data source, but manual review can be time consuming and burdensome. This study aims to validate a predictive electronic algorithm to identify children with rheumatic disease using on-demand self-service EHR reporting functionality.

Methods : Four diverse study sites using the same EHR (Epic Systems Corporation) created SlicerDicer reports. Queries included search terms for diagnosis (juvenile arthritis (ICD10-CM M08.*, M40.5), lupus and associated conditions (M32.*, L93.*, H01.12, M35.*), and dermatomyositis (M33.*)), department (pediatric rheumatology), and encounter type (office visit). Clinic schedules were manually reviewed both prospectively and retrospectively over a 2 week period to determine the diagnosis. SlicerDicer queries were run prospectively and retrospectively over the same two week period. Clinic schedules were compared to the SlicerDicer reports. Descriptive statistics, and logistic regression with clustering by site are reported.

Results : Of the total 674 patient visits evaluated throughout the study at four clinic sites, there were 589 (88%) patient visits prospectively evaluated, and 517 (77%) patient visits retrospectively evaluated. In total, there were 433 (64%) of patient visits included in both the prospective and retrospective queries. There were 85 visits added onto clinic schedules after prospective queries were completed, and 194 (29%) visits either cancelled or no-showed. Four patients who were added on after prospective queries were completed did not show to their appointments. Prospective queries had a sensitivity of 86%, specificity of 96%, PPV of 92%, and NPV of 92%, with an AUC of 0.92 (Figure 1A). Retrospective queries had a sensitivity of 80%, specificity of 97%, PPV of 95%, and NPV of 87%, with an AUC of 0.91 (Figure 1B). Age and return visit type were statistically associated with having a diagnosis and being present on both the prospective and retrospective queries in univariate analysis (Table 1). Of the 433 patients included in both the prospective and retrospective queries, 33 (8%) changed status between queries. Change of diagnosis status was not associated with age, sex, or visit type in univariate analysis (Table 2).

Conclusions : We were able to develop a successful query to identify subjects both prospectively and retrospectively with rheumatic conditions. Further integration of predictive algorithms into standard research screening workflows is a potential way to improve subject identification and recruitment.

IRB Statement : The study was approved by Nationwide Children’s Internal Review Board, STUDY00002524, and other study sites as necessary.

Acknowledgements : This work was funded by a CARRA-Arthritis Foundation Small Grant. The authors wish to acknowledge CARRA and the ongoing Arthritis Foundation financial support of CARRA.

**Fig. 1 (Abstract A15):**
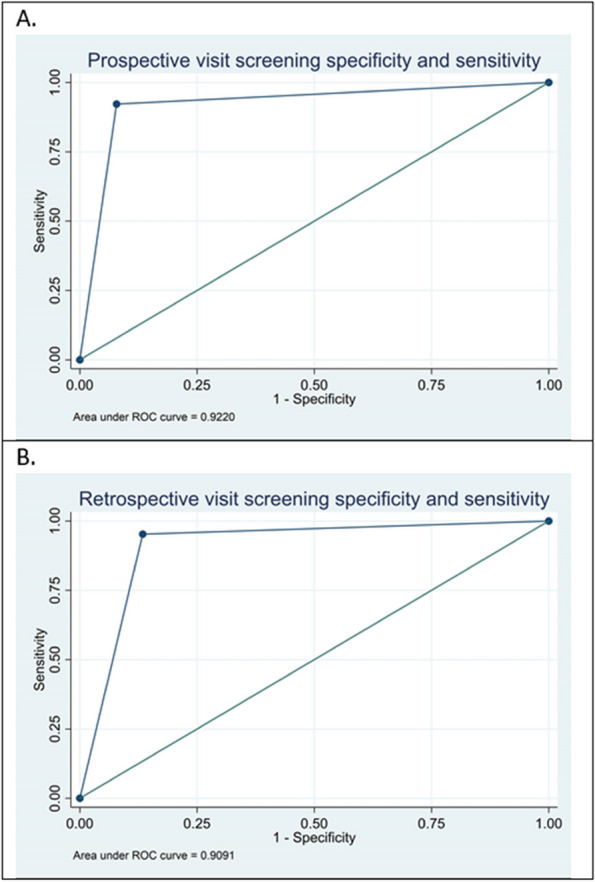
Receiver Operator Curve of Prospective and Retrospective Visit Screening Reports. **A** Receiver Operator Curve of Prospective Visit Screening with SlicerDicer queries containing search terms for diagnosis, department, and encounter type. **B** Receiver Operator Curve of Retrospective Visit Screening with SlicerDicer queries containing search terms for diagnosis, department, and encounter type

**Table 1 (Abstract A15):** Univariate predictors if patient has diagnosis and is included on query

	Prospective Query		Retrospective Query	
	B-coefficient (95% CI)	*p*-value	B-coefficient (95% CI)	*p*-value
Age	0.06 (0.02, 0.10)	<0.01	0.05 (0.08, 0.08)	0.02
Return visit type	4.39 (2.41, 6.36)	<0.01	2.56 (1.77, 3.35)	<0.01
Female sex	-0.06 (-0.43, 0.31)	0.75	0.20 (-0.19, 0.60)	0.31

**Table 2 (Abstract A15):** Univariate regression of change of diagnosis status

	B-coefficient (95% CI)	*p*-value
Age	0.04 (-0.01, 0.08)	0.08
Return visit type	-0.39 (-1.21, 0.43)	0.35
Female sex	0.17 (-0.63, 0.97)	0.68

## A16 Feasibility and acceptability of yoga for adolescents with juvenile idiopathic arthritis

### Adina Dawoud^1^, Jill Blitz^1^, Steffany Moonaz^2^

#### ^1^Children’s Hospital Los Angeles, Los Angeles, CA USA; ^2^Southern California University of Health Sciences

##### **Correspondence:** Steffany Moonaz

*Pediatric Rheumatology 2024*, **22(S1):**A16

Background : Yoga shows effectiveness in young adults with arthritis but has not been studied in adolescents with juvenile idiopathic arthritis (JIA). Unique challenges arise for this population, including decreased activity level and different psychosocial challenges than healthy peers. However, exercise and stress management are beneficial for JIA management. Yoga combines adaptable movement with tools for self-regulation. This project aimed to determine the feasibility and acceptability of an 8-week group yoga intervention for adolescents with JIA.

Methods : Of the 79 rheumatology clinic patients screened, 25 consented to participate in 2 cohorts (16 and 9). Participants were ages 14-18 years with a JIA diagnosis, spoke and understood English, and could get on and off the floor independently. All participants were cleared by a rheumatologist. Each 75-minute group session included breathing techniques, relaxation, mindfulness, and modified classical yoga postures, using various props and a yoga rope wall. An online yoga video was available for home practice. Outcomes measured at baseline and 8 weeks included physician global assessment with joint count, visual assessment with a joint damage assessment index, the Pediatric Quality of Life Arthritis Module 3.0 (Peds QL) and the Visual Analog Scale for pain. An anonymous satisfaction survey was administered after week 8 (5-point Likert scale). Descriptive statistics were used to summarize patient characteristics and outcomes. Wilcoxon signed-rank tests were used to assess difference in outcome measures before and after the intervention. Spearman correlation was used to assess correlations of global assessment and joint count with sessions attended.

Results : Of the 25 participants who consented, 13 attended at least one class with mean attendance of 5.7±2.2 classes. Common reasons for non-enrollment of eligible patients included distance, schedule conflicts, and lack of interest. Reasons for non-participation by enrolled participants is unavailable. Average distance to classes was 29.0±41.7 miles (range 5-202 mi.). For those who attended, there was a trend toward improvement for the Pain and Hurt domain of the Peds QL assessment (*p*=.16), but no other outcomes approached significance. Satisfaction data was available for 8 participants and was high in all areas, including pain improvement, program enjoyment, and likelihood of continuing yoga.

Conclusions : Adolescents with JIA who attended an 8-week yoga program reported enjoyment, pain reduction, and interest in continued practice. No adverse events were reported. This suggests yoga may be safe and acceptable for this population. A trend toward reduction in Pain and Hurt suggests possible effectiveness for improving these symptoms. These findings must be interpreted with great caution due to the very low enrollment and attendance, indicating barriers to feasibility such as scheduling and distance. Future studies should consider stakeholder engagement to understand and reduce barriers to participation, along with larger sample sizes to test intervention effectiveness.

IRB Statement : This project was approved by the Institutional Review Board of Children’s Hospital of Los Angeles, #CHLA-17-00037-CR001.

## A17 Creating a predictive algorithm for diagnosis of chronic inflammatory arthritis based on initial consultation data: a retrospective review at a quaternary pediatric rheumatology center

### Kendra Lauer, Kyla Driest, Alysha Taxter

#### Nationwide Children’s Hospital, Columbus, OH, USA

##### **Correspondence:** Kendra Lauer

*Pediatric Rheumatology 2024*, **22(S1):**A17

Background : Juvenile idiopathic arthritis (JIA) is the most common chronic rheumatic disease in children. With the current workforce shortage, wait times for initial consultation with pediatric rheumatology is lengthy. Providers are overwhelmed with referrals for non-specific joint complaints that infrequently result in an arthritis diagnosis. This study aims to develop a predictive algorithm for diagnosis of arthritis based on initial consultation visits using patient-reported, historical, and referral data.

Methods : All new patients seen in our quaternary pediatric rheumatology clinic between 2021 to 2023 were included. Reason for visit is recoded by the registration team at scheduling. Patients complete electronic patient reported outcomes (PROs) during routine clinical care, including a 56-point review-of-systems (ROS), pain, physical function, and morning stiffness. Diagnosis of chronic inflammatory arthritis was defined as ICD-10 codes of L40.5 (psoriatic arthritis), M05 (rheumatoid arthritis), M06 (other rheumatoid arthritis), M08 (JIA), or M45 (ankylosing spondylitis) documented as a current or future encounter diagnosis, problem list, or past medical history. Logistic regression evaluated the association of an arthritis diagnosis with patient and referral data; sample was randomly split into 80% derivation and 20% test set. Variables with *p*< 0.2 in univariate were included in multivariate, and a final multivariate model was evaluated on the test set. Complete data are reported.

Results : There were 2054 subjects; 1360 (66%) female, with a median age of 13 [IQR 8,16], and 197 (10%) were diagnosed with arthritis. Common reasons for referrals include pain (33%), swelling (12%), antinuclear antibody testing (11%), and fever (6%) (Table 1). Common patient-reported symptoms include joint complaints (71%), psychological symptoms (57%), muscle complaints (56%), gastrointestinal symptoms (52%), and neurological symptoms (52%). The PROs of patient global, pain, and physical function, as well as joint pain and morning stiffness, were higher or more commonly present, respectively, in children with arthritis (all *p*< 0.05). Positive constitutional, cardiac, and neurologic ROS domains were negatively associated with arthritis diagnosis whereas joint symptoms were positively associated with an arthritis diagnosis (all *p*< 0.05) (Table 2). A final predictive model had an area under the curve (AUC), sensitivity, and specificity of 0.83, 23%, and 99% (Table 3).

Conclusions : We were able to successfully build a predictive algorithm for diagnosis of arthritis by leveraging data potentially available before a consultative visit. Our data suggests that such models could be integrated into clinical referral pathways to assist with expedited access to pediatric rheumatology.

IRB Statement : The study was approved by Nationwide Children’s Hospital Institutional Review Board, approval number STUDY00003317.

Acknowledgements : None.

**Table 1 (Abstract A17):** Summary of referral and patient-reported data by diagnosis of inflammatory arthritis

	Total (*N*=2054)	No diagnosis of inflammatory arthritis (*N*=1857)	Diagnosis of inflammatory arthritis (*N*=197)	*p*-value
Refer for positive ANA	224 (11%)	219 (12%)	5 (3%)	<0.01
Refer for pain	673 (33%)	620 (34%)	53 (27%)	0.06
Refer for swelling	238 (12%)	167 (9%)	71 (36%)	<0.01
Refer for fever	122 (6%)	120 (6%)	2 (1%)	<0.01
Refer for rash	87 (4%)	85 (5%)	2 (1%)	0.02
Refer for fatigue	60 (3%)	58 (3%)	2 (1%)	0.10
Patient global, median [IQR]	4 [1, 6]	4 [2, 5]	5 [1, 9]	<0.01
Patient pain, median [IQR]	4 [1, 7]	4 [1, 6]	5 [3, 7]	<0.01
CHAQ, median [IQR]	0.25 [0, 0.88]	0.25 [0, 0.75]	0.5 [0.13, 1]	<0.01
Morning stiffness	1260 (62%)	1100 (60%)	160 (81%)	<0.01

Variables are reported as N (%) unless otherwise specified. CHAQ: childhood health assessment questionnaire

*IQR* interquartile range

**Table 2 (Abstract A17):** Review-of-system domains associated with diagnosis of inflammatory arthritis

	Total (*N*=2054)	No diagnosis of inflammatory arthritis (*N*=1857)	Diagnosis of inflammatory arthritis (*N*=197)	*p*-value
Constitutional	1470 (72%)	1341 (72%)	129 (65%)	0.05
Eyes	732 (36%)	660 (36%)	72 (37%)	0.78
Ears	432 (21%)	397 (21%)	35 (18%)	0.24
Nose	368 (18%)	339 (18%)	29 (15%)	0.22
Mouth/Throat	889 (43%)	810 (44%)	79 (40%)	0.34
Cardiac	815 (39%)	763 (41%)	52 (26%)	<0.01
Respiratory	614 (30%)	552 (30%)	61 (32%)	0.61
Gastrointestinal	1059 (52%)	968 (52%)	91 (46%)	0.11
Urinary	257 (12%)	234 (13%)	23 (12%)	0.71
Reproductive	246 (12%)	227 (12%)	19 (10%)	0.29
Muscle	1158 (56%)	1051 (56%)	107 (54%)	0.53
Joint	1459 (71%)	1285 (69%)	174 (88%)	<0.01
Dermatologic	837 (41%)	760 (41%)	77 (39%)	0.62
Hematologic	643 (31%)	581 (31%)	62 (31%)	0.96
Neurologic	1069 (52%)	994 (54%)	75 (38%)	<0.01
Psychiatric	1187 (57%)	1084 (58%)	103 (52%)	0.10

**Table 3 (Abstract A17):** Multivariate analysis factors associated with inflammatory arthritis diagnosis in test set

	Odds Ratio (95% CI)	*p*-value
Patient global	1.33 (1.09, 1.62)	0.01
Patient-reported pain	0.96 (0.79, 1.17)	0.68
Patient-reported morning stiffness	2.79 (0.95, 8.21)	0.06
CHAQ	0.61 (0.24, 1.52)	0.29
Refer for positive ANA	0.44 (0.05, 3.62)	0.44
Refer for pain	0.53 (0.22, 1.26)	0.15
Refer for swelling	7.22 (3.00, 17.35)	<0.01
Refer for fever	1.33 (0.15, 11.91)	0.80
Refer for rash	--	--
Refer for fatigue	1.13 (0.11, 11.99)	0.92
Positive mouth/throat ROS	1.22 (0.53, 2.84)	0.64
Positive cardiac ROS	1.03 (0.10, 2.61)	0.96
Positive joint ROS	1.18 (0.36, 3.85)	0.79
Positive neurologic ROS	0.21 (0.08, 0.57)	<0.01
Positive psychiatric ROS	0.90 (0.38, 2.13)	0.81

*ROS* Review-of Systems, *CHAQ* childhood health assessment questionnaire

## A18 Elucidating the factors that influence the use of minor salivary gland biopsy for the evaluation of childhood Sjögren’s disease

### Hemalatha Srinivasalu^1^, Brian Dizon^2^, Matthew Basiaga^3^, Sara Stern^4^, Akaluck Thatayatikom^5^, Seunghee Cha^6^, Scott Lieberman^7^, for the CARRA Sjögren’s Workgroup^8^

#### ^1^GW School of Medicine, Washington, D.C. USA; ^2^NIAMS, NIH; ^3^Mayo Clinic; ^4^University of Utah, Salt Lake City, UT, USA; ^5^AdventHealth for Children; ^6^University of Florida, Gainsville, Gainsville, FL USA; ^7^University of Iowa, Iowa City, IA, USA; ^8^Childhood Arthritis and Rheumatology Research Alliance (CARRA)

##### **Correspondence:** Hemalatha Srinivasalu

*Pediatric Rheumatology 2024*, **22(S1):**A18

Background : Childhood Sjögren’s disease (cSjD) is a rare condition, and its diagnosis is challenging due to a lack of pediatric-specific diagnostic criteria. In this project, we aimed to understand the current clinical trend for obtaining MSG biopsy in Childhood Sjogren’s followed by determining the characteristic pathology features and focus score cut-off for Childhood Sjogren’s Disease.

Methods : For aim 1, we analyzed data from the retrospective international cohort of 300 patients collected by the Childhood Sjögren’s Disease Workgroup. We compared the use of MSG biopsy in the evaluation of cSjD in the retrospective cohort according to the clinicians who submitted the cases, demographics, clinical manifestations, disease features, and other diagnostic testing. For aims 2 & 3, we have selected 50 age and sex-matched MSG biopsy from patients with Childhood Sjogren’s and controls. Pathologists are queried on the presence of small cellular aggregates, glandular fibrosis, acinar atrophy, fatty infiltration, granulomas in the biopsies along with determining focus scores. Digitalization and pathology review are currently underway.

Results : In the retrospective cohort, minor salivary gland (MSG) biopsy was performed as part of the evaluation for cSjD in 44% of patients. Among 26 clinicians who contributed cases of cSjD, no patterns in the use of MSG biopsy were observed. SSA and SSB were performed in 97% of patients, while ocular stain (18%), Schirmer test (45%), unstimulated salivary flow (15%), parotid sialography (12%), and salivary ultrasound (40%), were performed less frequently. Patients who had undergone MSG biopsy had significantly greater odds of having additional testing performed. Age less than 9 years (*p*=0.0003, OR 2.9), male biologic sex (*p*=0.005, OR 2.4), parotitis (*p*< 0.001, OR 2.8), and dry mouth (*p*=0.01, OR 1.8) were associated with a higher odds of MSG biopsy. Hypergammaglobulinemia (*p*=0.0001, OR 0.36) and serologies positive for SSA (*p* < 0.0001, OR 0.14), SSB (*p*< 0.0001, OR 0.30), RF (*p*=0.04, OR 0.59), or ANA (*p*=0.002, OR 0.33) were associated with a lower odds of MSG biopsy.

Conclusions : Use of MSG biopsy in the evaluation of cSjD was heterogeneous. While SSA and SSB were routinely performed in the evaluation of cSjD, additional tests, including MSG biopsy, appeared to be reserved for patients with atypical features such as young age, male biologic sex, and negative serologies. These observations suggest a framework for standardizing the evaluation of cSjD to improve diagnosis.

IRB Statement : This study was considered Exempt Certified by IRB at Children’s National Hospital. IRB # Pro00012976.

Acknowledgements : CARRA-Arthritis Foundation Small Grant. The authors wish to acknowledge CARRA and the ongoing Arthritis Foundation financial support of CARRA Childhood Arthritis and Rheumatology Research Alliance and the International Childhood Sjögren Syndrome Workgroup NIAMS, NIH

## A19 Clinical disease manifestations associated with TNF inhibitor non-response in juvenile spondyloarthritis

### Melissa Oliver^1^, Kelly Mosesso^1^, Pamela Weiss^2^, Robert Colbert^3^, Matthew Stoll^4^, Hemalatha Srinivasalu^5^, for the CARRA Registry Investigators^6^

#### ^1^Indiana University School of Medicine, Indianapolis, IN, USA; ^2^UPENN/CHOP; ^3^National Institute of Arthritis and Musculoskeletal and Skin Diseases, NIH; ^4^University of Alabama at Birmingham; ^5^GW School of Medicine; ^6^Childhood Arthritis and Rheumatology Research Alliance (CARRA)

##### **Correspondence:** Melissa Oliver

*Pediatric Rheumatology 2024*, **22(S1):**A19

Background : Tumor necrosis factor inhibitors (TNFi) are effective in children with juvenile spondyloarthritis (JSpA) and generally represent the first-line choice for biologic therapy. However, not all JSpA patients respond well to TNFi initially (primary non-response) or respond well but lose efficacy over time (secondary non-response). Children who do not respond to initial TNF inhibition are left with limited options. We aimed to identify the clinical characteristics associated with TNFi nonresponse in JSpA patients.

Methods : Retrospective analysis of JSpA patients and their response to first TNFi agent enrolled in the Childhood Arthritis and Rheumatology Research Alliance (CARRA) Registry between July 2015 - January 2019. JSpA population included enthesitis-related arthritis, psoriatic arthritis, and undifferentiated spondyloarthritis subtypes. Primary non-response defined as discontinuation of TNFi within the first 3 months of starting due to ineffectiveness and absence of other causes. Secondary non-response defined as 1) stopping TNFi AND 2) switching to another biologic or addition of a disease-modifying anti-rheumatic drug (DMARD) after 3 months OR for specific reason other than ineffectiveness if less than 3 month. Responders were defined as those continuing the 1st TNFi for duration of study. Demographic and disease characteristics were compared at 1) baseline visits prior to TNFi start and 2) time of initial TNFi non-response or visit at least 4 months following TNFi start for the responders.​

Results : Total of 286 patients met inclusion criteria; 168 TNFi responders and 118 TNFi non-responders. The median duration prior to initial TNFi non-response for the non-responders was 268 days (IQR:159.5, 426.5). Demographics are shown in Table 1. Mean age at TNFi start was 12.1y for non-responders (SD 4.0y) and 12.1y for responders (SD 4.1y). Non-responders were more likely to be female compared to responders, 60.5% vs 48.5% (*p*=0.04). BMI was higher in non-responders compared to responders, 22.5 vs 21 (*p*=0.01). Non-responders had more arthritis, enthesitis and sacroiliitis at their baseline visit prior to TNFi start and a post-TNFi start visit compared to responders shown in Table 2. The majority of non-responders were secondary non-responders (*N*=113/118; 96%) shown in Figure 1. Following initial TNFi therapy for all the non-responders, most patients switched to 2nd TNFi (60.2%), followed by DMARD (35.2%) then another biologic (4.6%).

Conclusions : JSpA patients who did not respond to initial TNFi had more active disease and more axial involvement at baseline prior to starting therapy. The majority of patients who failed TNFi were due to secondary non-response. Additionally, JSpA patients with a higher BMI at baseline may be a risk factor for TNFi non-response

IRB Statement : The study was conducted in accordance with the Declaration of Helsinki, and the protocol was approved by the Ethics Committee of Indiana University (protocol #: 1812741336).

Acknowledgements : The authors wish to acknowledge the support from Arthritis Foundation and CARRA. This project is funded by a CARRA-Arthritis Foundation small grant. This work could not have been accomplished without the aid of the following organizations: The NIH’s National Institute of Arthritis and Musculoskeletal and Skin Diseases (NIAMS) & the Arthritis Foundation. We would also like to thank all participants and hospital sites that recruited patients for the CARRA Registry. The authors thank the following CARRA Registry site principal investigators: K. Abulaban, C. Aguiar Lapsia, S. Ardoin, L. Barillas-Arias, M. Basiaga, K. Baszis, H. Brunner, H. Bukulmez, E. Chalom, J. Chang, D. Co, K. Cook, A. Cooper, C. Correll, T. Davis, F. Dedeoglu, M. DeGuzman, A. Dhanrajani, K. Ede, B. Edelheit, B. Feldman, I. Ferguson, D. Glaser, D. Goldsmith, B. Gottlieb, T. Graham, T. Griffin, T. Hahn, L. Harel, O. Harry, M. Hollander, S. Hong, M. Horwitz, J. Hsu, A. Huber, L. Imundo, C. Inman, P. Kahn, S. Kim, D. Kingsbury, M. Klein-Gitelman, L. Lim, M. Mannion, D. McCurdy, D. Milojevic, S. Mohan, T. Moore, K. Moore, L. Moorthy, S. Nativ, M. Natter, K. Onel, J. Patel, S. Prahalad, C. Rabinovich, A. Robinson, T. Ronis, M. Rosenkranz, N. Ruth, S. Sabbagh, K. Schikler, C. Schutt, E. Sloan, J. Spitznagle, Y. Sterba Rakovchik, K. Stewart, G. Syverson, S. Tarvin, M. Tesher, D. Toib, M. Toth, M. Twilt, H. Van Mater, D. Wahezi, P. Weiss, J. Weiss, L. Woolnough, E. Wu, A. Yalcindag, Y. Zhao

**Table 1 (Abstract A19):** Demographics by TNFi response status

Characteristic	Non-responder (***N***=118)	Responder (***N***=168)	*p* value
**Sex, Male (n, %)**	45 (38.1%)	87 (51.8%)	**0.023**
**Age, y**			0.910
Mean (SD)	12.1 (4.0)	12.1 (4.1)	
**Race (n, %)**			0.369
White	96 (86.5%)	124 (79.5%)	
Black	6 (4.8%)	10 (6.1%)	
Other race	14 (11.3%)	23 (14.1%)	
**Not Hispanic or Latino (n, %)**	108 (91.5%)	147 (87.5%)	0.281
**Disease duration, y**	1.2 (1.8)	1.3 (2.1)	0.295
**Positive HLA-B27** ^**a**^ **(n, %)**	35 (37.2%)	54 (41.5%)	0.516
**BMI** kg/m2	22.5 (6.6)	21.1 (6.3)	**0.023**

^a^20.3% & 22.6% HLA-B27 Not done/Unknown for non-responder & responder group, respectively

**Table 2 (Abstract A19):** Clinical characteristics at baseline and post-TNFi start visits

	Baseline visit			Post-TNFi start visit		
	Non-responder (***N***=118)	Responder (***N***=168)	*p* value	Non-responder (***N***=118)	Responder (***N***=168)	*p* value
**Active Joint Count** Mean (SD)	5.0 (6.5)	5.0 (6.0)	0.115	4.0 (6.1)	0.7 (1.6)	**<0.001**
**Tender Entheses** (n, %)	49 (45.0%)	53 (34.4%)	0.084	45 (41.7%)	22 (14.1%)	**<0.001**
**Clinically Active Sacroiliitis** (n, %)	36 (34.6%)	29 (18.8%)	**0.004**	25 (23.6%)	11 (7.4%)	**<0.001**
**Uveitis** (n, %)	6 (5.1%)	3 (1.9%)	0.177	7 (6.0%)	0 (0)	**0.002**
**PhGA** Mean (SD)	3.1 (2.1)	3.4 (2.4)	0.580	2.3 (1.9)	0.8 (1.1)	**<0.001**
**PtGA** Mean (SD)	4.6 (2.5)	3.5 (2.5)	**0.007**	4.3 (2.3)	2.1 (2.4)	**<0.001**
**CHAQ Disability Index** Mean (SD)	0.7 (0.6)	0.6 (0.6)	0.107	0.6 (0.6)	0.3 (0.5)	**<0.001**

Uveitis: ever had uveitis at baseline visit or had new onset, or previously reported uveitis at post-TNFi start visit, *PhGA* Physician Global Assessment (0-10), *PtGA* Patient Global Assessment (0-10), *CHAQ* Childhood Health Assessment Questionnaire (0-3)

**Fig. 1 (Abstract A19):**
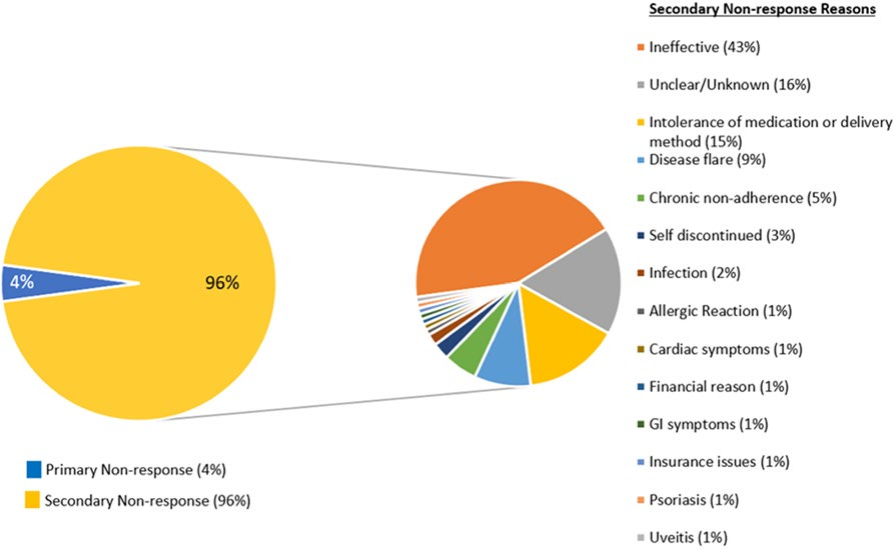
Reasons for TNFi Non-response

## A20 Defining and endotyping syndrome of undifferentiated recurrent fevers

### Marci Macaraeg^1^, Grant Schulert^1^, Nadine Saad^2^, Fatma Dedeoglu^3^, Tiphanie Vogel^4^

#### ^1^Cincinnati Children’s Hospital Medical Center, Cincinnati, OH, USA; ^2^University of Michigan, Ann Arbor, MI, USA; ^3^Boston Children’s Hospital, Boston, MA USA; ^4^Baylor College of Medicine/Texas Children’s Hospital

##### **Correspondence:** Marci Macaraeg

*Pediatric Rheumatology 2024*, **22(S1):**A20

Background : Syndrome of Undifferentiated Recurrent Fevers (SURF) is an understudied and poorly defined disease entity. It causes significant financial and emotional burden to patients and their families and is difficult to diagnose and manage. Our preliminary data suggest that at least two distinct groups exist within SURF with unique cytokine profiles. The goal of this study is to group patients diagnosed with SURF by endotype, identify candidate driving forces behind each group, and determine best treatment practices for SURF patients. Our hypothesis is that distinct groups exist within SURF, and that each group has unique clinical and cytokine characteristics, as well as different responses to treatment. The first specific aim of this study is to group SURF patients according to endotype using their clinical symptoms and cytokine findings. The second specific aim is to determine the characteristics and variables of SURF patients likely to benefit from early biologic initiation. Secondary outcomes from this aim will be to identify characteristics and variables of patients who experience disease control with on-demand steroids, colchicine, or cimetidine alone.

Methods : Patients diagnosed with SURF after March 2018 are being enrolled into this study at Cincinnati Children’s, Texas Children’s, Boston Children’s, and University of Michigan. Inclusion criteria for SURF includes: 1) age between 0-18, 2) history of recurrent fevers, defined as at least 3 episodes in the past that cannot be attributed to infection or other disease, 3) fever episodes are stereotypical, 4) not consistent with PFAPA or a hereditary fever syndrome based on the 2019 Eurofever/PRINTO criteria, and 4) negative genetic testing if symptoms are consistent with an HRF. Demographics, clinical course, and treatment responses will be recorded in REDCap, and some patients will provide biosamples for multiplex cytokine analysis, which will be performed at CCHMC.

Results : Preliminary results from the CCHMC cohort show that SURF is heterogeneous in presentation and treatment response. Hierarchical cluster analysis of peripheral cytokine analysis shows that a subset of SURF patients cluster together with higher levels of IL-12p70, IL-23, and IFNγ compared to the rest of SURF and all PFAPA patients. Lastly, the CCHMC cohort also showed that many SURF patients had variants of unknown clinical significance in genes implicated in B cell development, immunodeficiencies, granulocyte/monocyte development, and inflammatory bowel disease risk. The heterogeneity, clustering, and genetic findings led to the hypothesis of the existence of endotypes within SURF. This study is a step to identify these endotypes. This multi-institutional study is ongoing, with data currently still being collected.

Conclusions : Preliminary data from CCHMC suggest that different endotypes exist within SURF. This multi-institutional study will further evaluate whether there exist distinct SURF endotypes, what drives the disorder and each of its endotypes, and how physicians can better predict which treatment will be most successful for each patient.

IRB Statement : This study was approved by the Institutional Review Board of Cincinnati Children’s Hospital Medical Center (CCHMC IRB), and written informed consent was obtained from all patients and/or their legal guardians.

Acknowledgements : The authors wish to acknowledge CARRA and the ongoing Arthritis Foundation financial support of CARRA. The authors would like to thank the CARRA-Arthritis Foundation grant for funding this project, the Lellen Family Foundation for supporting the Pediatric Autoinflammatory Disease registry at CCHMC, the CARRA Autoinflammatory and Rare disease workgroup for all of their support, and the patients and families who participate in this study.

## A21 Proposed pathways involved in the pathogenesis of juvenile dermatomyositis

### Samantha Coss^1^, Rabheh Abdul Aziz^1^, Danlei Zhou^2^, Katherine Miller^1^, Stacy Ardoin^1^, Edward Oberle^1^, Kyla Driest^1^, Ohoud Al Ahmed^1^, Anjali Patwardhan^1^, Shoghik Akoghlanian^1^, Vidya Sivaraman^1^, Joanne Drew^1^, Charles Spencer^1^, Chack-Yung Yu^1^

#### ^1^Nationwide Children’s Hospital, Columbus, OH, USA; ^2^The Abigail Wexner Research Institute at Nationwide Children’s Hospital

##### **Correspondence:** Samantha Coss

*Pediatric Rheumatology 2024*, **22(S1):**A21

Background : Juvenile dermatomyositis (JDM) is an autoimmune disease that causes skin and muscle inflammation. Why patients develop JDM is not well understood. We sought to investigate the relationship between genetic risk factors, including C4 GCN, autoantibodies, and clinical features in JDM.

Methods : Subjects were recruited from 2 pediatric institutions (*n*=61). C4 GCN was determined by Southern blot. NovaSeq6000 S2 PE150bp whole exome sequencing (WES) provided 100x median coverage (*n*=45). Comparative analyses were performed via the student’s t test (parametric) and via the Mann Whitney U test (non-parametric). Correlation was assessed via linear regression. Contingency analyses were performed via Fisher’s exact test.

Results : C4 GCN was associated with muscle pathology and extra-muscular disease at diagnosis. Lower C4A and C4L GCN but higher C4B and C4S GCN were associated with a higher number of abnormal muscle enzymes (*p*=0.034, *p*=0.020, 0.026, and *p*=0.010, respectively). Higher C4S GCN also correlated with lower muscle strength (*p*=0.039) and lower C4L with abnormal MRI (*p*= 0.039). C4S GCN is one of the strongest factors in the development of disease complications. Higher GCN of C4S was a risk factor for arthritis (*p*=0.032), systemic symptoms (*p*=0.020), and dysphagia at diagnosis (*p*=0.0078). C4 GCN was associated with myositis-related antibodies. Patients with homozygous C4A deficiency were more likely to test positive for anti-NXP2 (OR 20.00 [2.71-261.1], *p*=0.011), and A higher C4B GCN correlated with development of myositis-associated antibodies (*p*=0.043). Patients with JDM showed increased complement activation. Levels of circulating C3a, C4a, and C5a were higher in patients with JDM (*p*< 0.0001, *p*=0.028, and *p*< 0.0001). Patients with JDM also showed increased deposition of complement split product C4d on RBC as measured by flow cytometry (*p*< 0.0001); this was most notable in patients with C4A insufficiency and higher C4B GCN (*p*=0.0003 and *p*=0.003). We performed WES and ingenuity pathway analysis to identify disease drivers. We identified genes involved in endothelial integrity (laminin 5, collagen 6A6), skin and muscle homeostasis (tyrosinase, TYR, involved in melanin biosynthesis; titin; and Fer-1 like family member 5, which may be involved in myocyte membrane repair), mitochondrial function (solute carrier protein 25, SLC25), and transcriptional regulation (zinc fingered protein 48). Many of these genes were mutated in large portions of our patients: 30% carry titin mutations and 22% SLC25.

Conclusions : Both genetic and environmental factors contribute to the development of JDM. Yet the initial trigger for JDM remains unclear. Here, we show that low GCN of C4A and C4L and high GCN of C4B and C4S correlate with muscle disease, extra-muscular pathology at diagnosis, and autoantibody production. In addition, we have identified candidate gene pathways that may be involved in disease pathogenesis. These data suggest that C4 plays a key role in the pathogenesis of JDM and that mutations in proteins involved in mucosal, skin, and muscle homeostasis may act as triggers for aberrant autoreactive immune responses that then further perpetuate autoimmunity in JDM.

IRB Statement : All studies were approved by the Nationwide Children’s Hospital and Children’s Hospital of Buffalo’s respective IRBs. Informed consent was obtained prior to each subject’s participation.

## A22 Social determinants of health in children with rheumatic disease: a single center cohort

### Kristina Ciaglia^1^, Elizabeth Sloan^2^, Tracey Wright^2^

#### ^1^UT Southwestern Medical Center and Scottish Rite Hospital for Children; ^2^Texas Scottish Rite Hospital, Dallas, TX USA

##### **Correspondence:** Kristina Ciaglia

*Pediatric Rheumatology 2024*, **22(S1):**A22

Background : Rheumatic disease disproportionately impacts certain socioeconomic, racial, and ethnic groups frequently resulting in health care inequities. Social determinants of health (SDOH) are conditions in the environment where people exist that encompass a wide range of systems, and influence quality of life, outcomes, and risks. Aligning with health equity literature, children from disadvantaged socioeconomic backgrounds experience worse outcomes and delays in care.1 In adult SLE, the negative impacts of SDOH are well described with unfavorable determinants leading to more severe disease and increased mortality.2 We aimed to identify SDOH in a cohort of children with rheumatic disease followed at a single center to begin to explore risk factors for disparities in our patient population.

Methods : Patient caregivers completed a SDOH survey prior to the start of the clinic visit. Survey results from February to May 2023 were extracted from the EMR. Patient demographics, diagnosis, and survey responses were analyzed using standard descriptive statistics.

Results : We analyzed SDOH surveys (*n*=861) completed during the initial survey roll out. Most patients surveyed were female (70%), and the average age was 11.7 years. Seventy percent reported White race, and 38% identified their ethnicity as Hispanic/Latino. Eleven percent of respondents had no high school degree, and 7.6% reported they need help with reading materials. Approximately 4% of patients reported needing help with cost of care, utilities, food, housing, or transportation. Twenty-one percent reported being often or sometimes worried about food security (Table 1). Of the patients surveyed, 66 had a diagnosis of SLE, 50 had JDM, and 371 had JIA (Table 2). In comparison with JIA and JDM families, SLE caregivers disclosed a significant higher rate of needing help with reading materials (*p*=0.0136) and lack of high school degree (*p*=0.0355).

Conclusions : Patients seen in our center come from diverse socioeconomic, racial, and ethnic backgrounds. The SDOH survey identified critical barriers to patient care including health literacy, food security, and living costs such as transportation, housing, and utilities. Health literacy may be an essential limiting factor for our families with children who have SLE. Further study is needed to understand the impact of SDOH on outcomes for children with rheumatic disease and how it varies by diagnosis. Future efforts to improve the management and outcomes of children with rheumatic disease should focus on specific SDOH that could influence health inequities. 1. Akinsete AM, Woo JMP, Rubinstein TB. Disparities in Pediatric Rheumatic Diseases. Rheum Dis Clin North Am. Feb 2022;48(1):183-198. doi:10.1016/j.rdc.2021.09.014 2. Williams JN, Drenkard C, Lim SS. The impact of social determinants of health on the presentation, management and outcomes of systemic lupus erythematosus. Rheumatology (Oxford). Mar 29 2023;62(Suppl 1):i10-i14. doi:10.1093/rheumatology/keac613

IRB Statement : This is a restrospective chart review that is under an IRB for our rheumatology department.

**Table 1 (Abstract A22):** SDOH survey responses from patients seen in rheumatology clinic

Social Determinant of Health Survey Questions	Responses: Number of patients (%)
In the last 12 months, was there a time when you were not able to pay the mortgage or rent on time?	Yes: 109 (12.7%) No: 569 (66.1%) Refused to answer/blank: 183 (21.2%)
In the last 12 months, how many places have you lived?	1: 606 (70.4%) 2: 58 (6.7%) 3+: 6 (0.7%) Refused to answer/blank: 191 (22.2%)
In the last 12 months, was there a time when you did not have a steady place to sleep or slept in a shelter (including now)?	Yes: 19 (2.2%) No: 674 (78.3%) Refused to answer/blank: 168 (19.5%)
In the past 12 months, has lack of transportation kept you from medical appointments or from getting medications?	Yes: 39 (4.5%) No: 621 (72.2%) Refused to answer/blank: 201 (23.3%)
In the past 12 months, has lack of transportation kept you from meetings, work, or from getting things needed for daily living?	Yes: 35 (4.1%) No: 629 (73%) Refused to answer/blank: 197 (22.9%)
Within the past 12 months, you worried that your food would run out before you got the money to buy more.	Never true: 508 (59%) Sometimes true: 121 (14.1%) Often true: 19 (2.2%) Refused to answer/blank: 213 (24.7%)
Within the past 12 months, the food you bought just didn’t last and you didn’t have money to get more.	Never true: 542 (63%) Sometimes true: 92 (10.6%) Often true: 11 (1.3%) Refused to answer/blank: 216 (25.1%)

**Table 2 (Abstract A22):** SDOH demographics and responses in patients with pediatric rheumatic disease by diagnosis

	Overall Cohort (***n*** = 861)	SLE Cohort (***n*** = 66)	JDM Cohort (***n*** = 50)	JIA Cohort (***n*** = 371)
**Mean Age (SD)**	11.7 (4.4)	14.9 (3.1)	11.4 (4.9)	11.7 (4.3)
**Race/Ethnicity** Number (%)				
Non-Hispanic White	311 (36.1%)	8 (12%)	26 (52%)	173 (46.6%)
Black/African American	65 (7.5%)	11 (16.7%)	5 (10%)	32 (8.6%)
Asian	36 (4.2)	4 (6%)	2 (4%)	13 (3.5%)
American Indian	7 (0.8%)	1 (1.5%)	1 (2%)	4 (1.1%)
Other Race	129 (15%)	6 (9.1%)	2 (4%)	33 (8.9%)
Hispanic/Latino	310 (36%)	38 (57.6%)	24 (48%)	117 (31.5%)
**Sex** Number (%)				
Male	259 (30%)	7 (10.6%)	16 (32%)	106 (28.9%)
Female	602 (70%)	59 (89.4%)	34 (68%)	265 (71.4%)
**Insurance Status** Number (%)				
Government/None	369 (42.9)	73 (65.2%)	25 (50%)	148 (39.9%)
Commercial	492 (57.1%)	23 (34.8%)	25 (50%)	223 (60.1%)
**SDOH Responses** Number (%)				
“I need help with reading materials”				
Yes	66 (7.6%)	10 (15.2%)	6 (12%)	18 (4.9%)
No	632 (73.4%)	35 (53%)	24 (48%)	240 (64.7%)
Refused to answer/blank	163 (18.9%)	21 (31.8%)	20 (40%)	113 (30.4%)
“Do you have a High School degree?”				
Yes	592 (68.8%)	32 (48.5%)	24 (48%)	228 (61.4%)
No	101 (11.7%)	11 (16.7%)	6 (12%)	31 (8.4%)
Refused to answer/blank	168 (19.5%)	23 (34.9%)	20 (40%)	112 (30.2%)

## A23 Resilience & mental health in childhood-onset systemic lupus erythematosus: a cross-sectional study

### Isabella Zaffino^1^, Ashley Danguecan^1^, Joanna Law^1^, Kiah Reid^1^, Angela Cortes^1^, Eugene Cortes^1^, Sandra Williams-Reid^1^, Adrienne Davis^1^, Asha Jeyanathan^1^, Sona Sandhu^1^, Lawrence Ng^1^, Paris Moaf^1^, Deborah Levy^1^, Linda Hiraki^1^, Andrea Knight^2^

#### ^1^The Hospital for Sick Children, Toronto, Canada; ^2^The Hospital for Sick Children and SickKids Research Institute, Toronto, Canada

##### **Correspondence:** Isabella Zaffino

*Pediatric Rheumatology 2024*, **22(S1):**A23

Background : Childhood-onset systemic lupus erythematosus (cSLE) presents enduring physical and mental health struggles, with up to 60% and 37% of patients experiencing depressive and anxiety symptoms, respectively. Despite its potential to influence mental health outcomes, resilience, defined as the ability to adapt following experiences of adversity, remains understudied in cSLE. We aimed to 1) describe individual psychological and socio-ecological resilience within a cSLE cohort, and 2) examine the relationship between resilience levels with mental health outcomes and disease characteristics (Figure 1).

Methods : We conducted a prospective cross-sectional study of youth ages 11 to 18 years, meeting ACR/SLICC classification criteria for cSLE. Participants were recruited from The Hospital for Sick Children Lupus Clinic between October 2021 and October 2023. Youth completed the Connor-Davidson Resilience Scale 10-item (CD-RISC 10) and the Child and Youth Resilience Measure Revised (CYRM-R), measuring individual psychological resilience (i.e., personal ability to recover from adversity) and socio-ecological resilience (i.e., feeling a sense of belonging within society), respectively. In both measures, higher scores indicate more resilience. Mental health outcomes included symptoms of depression (Beck Depression Inventory- Second Edition (BDI-II)) and anxiety (Screen for Child Anxiety Related Emotional Disorders (SCARED)). In both measures, higher scores indicate more severe symptoms. Disease-related outcomes were disease activity (Systemic Lupus Erythematosus Disease Activity Index (SLEDAI)) and duration. We examined associations between CD-RISC and CYRM-R scores, and depression, anxiety, disease activity and disease duration using Pearson correlations.

Results : Thirty-six youth with cSLE were recruited (Table 1); 83.3% were female and 80.6% were non-white, with mean age of 15.3 years (SD=1.9). Mean resilience scores were CD-RISC of 26.4 (SD=6.7) and CYRM-R of 74.0 (SD=7.1). Mean scores were BDI-II of 13.3 (SD=10.0) and SCARED of 24.6 (SD=13.9), for depressive and anxiety symptoms. The mean SLEDAI score was 8.8 (SD=7.7) and disease duration 2.6 years (SD=2.4). A statistically significant association was found in which lower depressive symptoms were associated with higher resilience scores on both the CD-RISC 10 (r= -0.46, *p*=0.02), and CYRM-R (r= -0.42, *p*=0.03). No statistically significant associations were found between CD-RISC 10 or CYRM-R with the outcomes of anxiety symptoms, disease activity, or disease duration (Table 2).

Conclusions : In this cSLE cohort, higher individual and socio-ecological resilience were associated with lower levels of depressive symptoms in youth with cSLE, suggesting the potential for interventions that promote resilience as a means to decrease depression symptoms. Further exploration of patient and disease-related factors contributing to resilience in cSLE is needed.

IRB Statement : This project has been approved by the Research Ethics Board at The Hospital for Sick Children (REB #1000078857).

Acknowledgements : This project is supported by funding from the Lupus Research Alliance, US Department of Defense, SickKids Garry Hurvitz Centre for Brain and Mental Health Outcomes, and a Canada Research Chair in Mental Health and Chronic Disease of Childhood (to Dr. Knight).

**Fig. 1 (Abstract A23):**
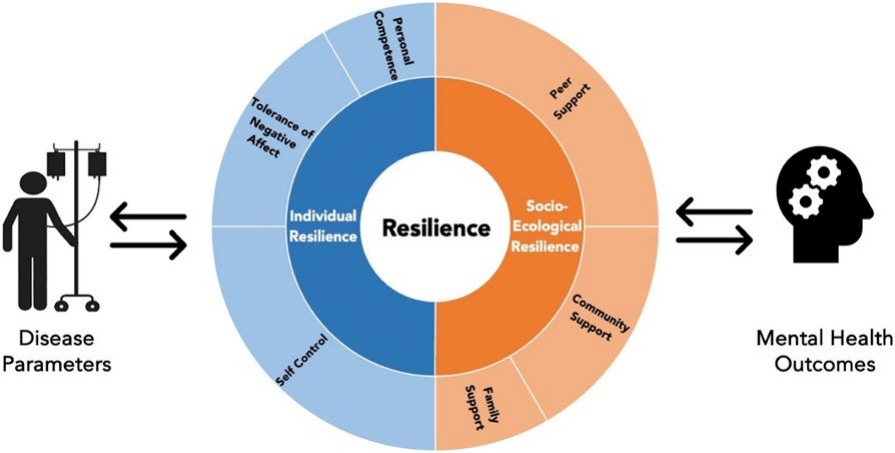
Conceptual Model for Relationship Among Resilience, Disease Parameters and Mental Health Outcomes According to the Disability-Stress Coping Model, CD-RISC 10 and CYRM-R

**Table 1 (Abstract A23):** cSLE patient descriptive statistics

Demographic Characteristics	Total Cohort (***n***=36)^**a**^
Age at study visit in years, mean (SD)	15.3 (1.9)
Sex/gender, n (%)	
Female	30 (83.3)
Male	4 (11.1)
Other	2 (5.6)
Race/ethnicity, n (%)	
Asian	21 (58.3)
Black or African American	4 (11.1)
White	7 (19.4)
Other	4 (11.1)
Household income, n (%)	
Above poverty line	22 (61.1)
Below poverty line	11 (30.6)
**Disease Characteristics**	
Age at diagnosis in years, mean (SD)	11.9 (5.1)
Disease duration in years, mean (SD)	2.6 (2.4)
Disease activity (SLEDAI), mean (SD)	8.8 (7.7)
Disease damage (SDI) (Score >0), n (%)	0 (0)
**Resilience Characteristics**	
CD-RISC 10, mean (SD)	26.4 (6.7)
CYRM-R, mean (SD)	74.0 (7.1)
**Mental Health Characteristics**	
Depressive symptom BDI-II total score, mean (SD)	13.3 (10.0)
Clinically elevated depressive symptoms, n (%)	
Minimal	10 (27.8)
Mild	11 (30.6)
Moderate	2 (5.6)
Severe	4 (11.1)
Anxiety symptom SCARED total score, mean (SD)	24.6 (13.9)
Clinically elevated anxiety symptoms, n (%)	13 (36.1)
Panic disorder/somatic symptoms	8 (22.2)
Generalized anxiety disorder	11 (30.6)
Separation anxiety disorder	10 (27.8)
School anxiety disorder	9 (25.0)
School avoidance	10 (27.8)

^a^Missing Data: Income (*n*=3), Depression (*n*=9), Anxiety (*n*=9), Age at diagnosis in years (*n*=4), Disease duration in years (*n*=4), Disease activity (*n*=6), Disease damage (*n*=5)

**Table 2 (Abstract A23):** Pearson correlations of resilience, mental health, and disease outcomes

	Resilience	
Mental Health Outcomes	CYRM-R *r* (*p* value)	CD-RISC 10 *r (p* value)
Depressive symptoms (BDI-II)	-0.42 (0.03)*	-0.46 (0.02)^*^
Anxiety symptoms (SCARED)	-0.05 (0.80)	-0.12 (0.55)
**Disease Outcomes**		
Disease activity (SLEDAI)	-0.01 (0.97)	-0.001 (0.10)
Disease duration	-0.29 (0.11)	-0.02 (0.89)

^*^Indicates statistically significant results (*p* < 0.05)

## A24 Chronic nonbacterial osteomyelitis (CNO): continued delays in diagnosis and treatment lead to damage

### Karen Onel^1^, Johanna Gandelsman^2^

#### ^1^Hospital for Special Surgery, New York, NY, USA; ^2^Rush Medical College

##### **Correspondence:** Karen Onel

*Pediatric Rheumatology 2024*, **22(S1):**A24

Background : CNO is a rare autoinflammatory bone disease characterized by sterile uni- or multifocal bone lesions that often begins in childhood. It is currently approached as a diagnosis of exclusion. Symptomatic patients undergo painful testing, often for years, to rule out disease mimickers leading to delays in treatment.

Methods : We analyzed the diagnostic course and barriers to treatment of consecutive CRMO patients seen in a Pediatric Rheumatology clinic at a tertiary hospital with a focus on bone and joints at least twice over a ten-year span between August 2011 and August 2021, including clinical signs, labs, and imaging studies. Data presented in Table 1.

Results : Of 59 patients eventually diagnosed with CRMO, the mean time from symptom onset to diagnosis was 2.1 years. Bone pain was the most common complaint, followed by bone swelling and morning stiffness. A family history of autoimmunity was common, and many children presented with or developed a related rheumatic disease including psoriasis, inflammatory bowel disease and spondylarthritis. Whole body imaging was done on all and most children had multifocal disease. Bone biopsies were commonly performed. All 18 children with unifocal lesions underwent biopsy except for one that surgeons felt would be difficult and potentially dangerous. 3 patients underwent three biopsies and several others had two. 4 patients were referred directly from pediatrics to pediatric rheumatology. More than half of the cohort saw at least two physicians in addition to their pediatrician prior to referral. Most commonly children were seen by combinations of orthopedics, sports medicine, oncology, and infectious disease specialists. Courses of antibiotics were common with several receiving 3 courses. Despite imaging and biopsy results consistent with CRMO, as well as persistent symptoms, many children (23%) received no DMARD treatments for at least one year after biopsy. Most common treatments besides NSAIDs were cDMARDs, bisphosphonates,and bDMARDs. Permanent damage including vertebral fractures secondary to delay in treatment was common. Several patients required surgery including one patient who required a limb salvage procedure. Ten of these patients have been followed for more than 5 years, 7 of whom are still receiving treatment at the time of analysis.

Conclusions : The lack of awareness about CRMO leads to enormous emotional, physical, and financial burdens on patients and their families. Persistent delays in diagnosis and treatment can lead to permanent damage. These delays are likely conservative as compared to those experienced by children living far from specialized pediatric care. Increasing disease awareness amongst general pediatricians, pediatric subspecialists, orthopedists and radiologists is critically needed.

IRB Statement : Protocol submitted to HSS IRB. As information is de-identified and data is batched, waiver of submission was granted.

**Table 1 (Abstract A24):** Demographic, clinical and laboratory characteristics of patients with CRMO

	Total (*n*=59)
**Demographics**	
Female/male	39:20
Age at onset, years, mean (range)	mean (5-13.5)
Age at diagnosis, years, mean (range)	11 (6-16)
Time from first symptom to diagnosis, years, mean (range)	2.1 (0.25-16)
Follow-up, months, median (range)	24 (6-60)
**Clinical Features n (%)**	
Presenting symptoms	
Bone pain	56/59 (95%)
Bone swelling	22/59 (37%)
Morning stiffness	21/59 (36%)
Family History of Inflammatory Disease	37/59 (63%)
Personal History of 2nd Inflammatory Disease	35/59 (59%)
Unifocal:Multifocal	18:41
Bone Biopsy	43/59 (72%)
**Treatments**	
Antibiotics	12/59
NSAIDs	56/59
Biologics	33/59
Conventional DMARDS	15/59
Glucocorticoids	4/59
Bisphosphonates	9/59

## A25 Analysis of SLCO1B1 variants as predictors of methotrexate tolerance in mexican children with juvenile idiopathic arthritis

### Jimena Garcia-Silva^1^, Beatriz Silva-Ramirez^2^, Ana Leos-Leija^3^, Viviana Leticia Mata-Tijerina^2^, Maria de Lourdes Aldana-Galvan^4^, Ana Villarreal-Treviño^3^, Nadina Rubio-Perez^5^, Fernando Garcia-Rodriguez^5^

#### ^1^Hospital Universitario “Dr. Jose Eleuterio Gonzalez” Universidad Autónoma de Nuevo León; Monterrey, México; ^2^Centro de Investigación Biomédica del Noreste, Instituto Mexicano del Seguro Social; ^3^Hospital Universitario “Dr. Jose Eleuterio Gonzalez”, Monterrey, Nuevo Leon, Mexico; ^4^Hospital Universitario “Dr. Jose Eleuterio González”, Universidad Autonoma de Nuevo León; ^5^Hospital Universitario “Dr. Jose Eleuterio Gonzalez”, Universidad Autonoma de Nuevo León

##### **Correspondence:** Jimena Garcia-Silva

*Pediatric Rheumatology 2024*, **22(S1):**A25

Background : Methotrexate (MTX) is the disease-modifying drug of choice for most clinical forms of juvenile idiopathic arthritis (JIA). The most frequent adverse events (AEs) of MTX are gastrointestinal alterations and hepatotoxicity, which can affects adherence to treatment, leading to the activation of the disease. The SLCO1B1 gene codes for a liver protein that is responsible for drug transportation. Genetic variation within the SLCO1B1 gene locus impacts drug transport, which can lead to altered pharmacokinetic profiles. Our aim was to determine the association between Single Nucleotide Polymorphisms (SNPs) in the SLCO1B1 gene (rs4149056, rs2306283) with the presence of AEs in patients with JIA being treated with MTX.

Methods : Observational study, where polymorphisms of the SLCO1B1 gene were analyzed in 23 patients with JIA and were related to the adverse events reported in the clinical record during therapy with MTX. The SNPs were determined with Fast Real Time-PCR using TaqMan®SNP Assays. The haplotypes were conformed of the two SNPs at *1A, *1B, *5, and *15. Allele frequencies, genotypes and haplotypes were compared using the following programs: https://www.snpstats.net/ and Epi Info V7 https://www.cdc.gov/epiinfo/esp/es_index.html.

Results : Twenty-three children diagnosed with JIA were included: 17 girls (73.91%), with a mean age of 11.26 (3-17) years. Fourteen patients (60.86%) presented some type of adverse effect, with gastrointestinal alterations being the most frequent (13, 92.85%). The distribution of genotypes was found in Hardy-Weinberg equilibrium. The SLCO1B1*1B was associated with the presence of more AEs in the response to MTX in patients with JIA (OR=4.22, 95%CI=1.19 -18.89, *p*=0.03).

Conclusions : SLCO1B1 genotyping is a promising way to identify patients at higher risk of getting AE during treatment with MTX allowing individualization of the treatment. Due to the sample size, the results should be interpreted as preliminary and further confirmatory studies in a larger cohort are needed.

IRB Statement : The study complies with the ethical guidelines of the Declaration of Helsinki and local regulations. This is a minimal-risk study, all the procedures were revised and approved by the ethics committee of our institution (Facultad de Medicina y Hospital Universitario, Universidad Autónoma de Nuevo León).

## A26 Depression and transition readiness in adolescents and young adults with childhood onset rheumatic disease: is there a correlation?

### Rebecca Overbury, Devin Eddington, Katherine Sward, Aimee Hersh

#### University of Utah, Salt Lake City, UT, USA

##### **Correspondence:** Rebecca Overbury

*Pediatric Rheumatology 2024*, **22(S1):**A26

Background : Patients with childhood onset rheumatic disease (CORD) transfer from a pediatric rheumatologist to an adult rheumatologist. Health care transition (HCT) prepares these patients for transfer. At the University of Utah, a rheumatology transition clinic serves patients who need to prepare for transfer. In transition clinic we address transition readiness. However, comorbidity with mental health disease is common in this patient population. There is a gap in understanding how depression might affect transition readiness. In this research we ask whether depression is correlated with transition readiness or transition readiness over time in our transition clinic patient population.

Methods : Patients in our transition clinic can participate in the associated research registry (*n* = 175). We collect the Transition Readiness Assessment (TRA) survey, from GotTransition electronically at two timepoints. First, upon enrollment into the registry (Module 2), and second just prior to transferring to an adult clinic (Module 5). We similarly collect a Patient Health Questionnaire (PHQ9). This is requested between the first and second TRA. Results from TRA and PHQ9 surveys were compared for correlation. Pearson’s product-moment correlation coefficient was used for calculating correlation between TRA and PHQ9. A linear regression model was fitted for correlation between demographic data (sex, race, ethnicity, and age at diagnosis) and TRA and PHQ9. For TRA, a higher score correlates to great transition readiness (0 - 23). For PHQ9, a higher score correlates with greater symptoms of depression (0 - 27).

Results : The mean TRA score for Module 2 was 16.9 (SD 4.6), range (4 - 23). The mean TRA score for Module 5 was 18.9 (SD 4.3), range (7 - 23). The mean difference in TRA from Module 2 to Module 5 was 2.2 (SD 3.6), range (-5 - 16). The mean PHQ9 score was 7.2 (SD 6.7), range (0 - 27) (Table 1). Between Module 2 TRA and PHQ9 the correlation coefficient was -0.0975 (95% CI: -0.2856 to 0.0979), with a *p*-value of 0.3272 (Figure 1). This suggests a weak, non-significant negative correlation. Between Module 5 TRA and PHQ9 the correlation coefficient was -0.0872 (95% CI: -0.3811 to 0.2226) with a *p*-value of 0.5828 (Figure 2). This implies a weak, non-significant negative correlation. The correlation between changes in TRA and PHQ9 was -0.0493 (95% CI: -0.3481 to 0.2585) with a *p*-value of 0.7563. This indicates a weak, non-significant negative correlation. There was no statistically significant relationship between demographic values and Module 2 TRA (*p* = 0.6199) or PHQ9 (*p* = 0.5277). This analysis indicates weak and non-significant correlations between TRA, PHQ9, and demographic factors.

Conclusions : This is a negative study. No statistically significant correlations were identified. However, our analysis shows consistent negative correlations between higher TRA scores (greater transition readiness) and lower PHQ9 scores (less symptoms of depression). Additionally, the mean PHQ9 score for all participants was 7.2, interpreted as a provisional diagnosis of mild depression. While not causative, this data suggests that addressing depression simultaneously to transition readiness may be a critical component to HCT in adolescent and young adult patients with CORD.

IRB Statement : This research involved human participants and was performed in accordance with the Declaration of Helsinki. It was reviewed and approved by the University of Utah Institutional Review Board (IRB).

Acknowledgements : This investigation was supported by the University of Utah Study Design and Biostatistics Center, with funding in part from the National Center for Research Resources and the National Center for Advancing Translational Sciences, National Institutes of Health, through Grant UL1TR002538 (formerly 5UL1TR001067-05, 8UL1TR000105 and UL1RR025764).

**Table 1 (Abstract A26):** TRA scores, PHQ scores, and demographics

Variable	Levels	Summary (***N***=173)
TRA Score Mod2	Mean (SD)	16.9 (4.6)
	Median (IQR)	18.0 (14.0, 21.0)
	Range	(4.0, 23.0)
TRA Score Mod5	Mean (SD)	18.9 (4.3)
	Median (IQR)	20.0 (17.0, 23.0)
	Range	(7.0, 23.0)
Module Score Difference	Mean (SD)	2.2 (3.6)
	Median (IQR)	2.0 (0.0, 4.0)
	Range	(-5.0, 16.0)
PHQ9 Score	Mean (SD)	7.2 (6.7)
	Median (IQR)	5.0 (2.0, 10.0)
	Range	(0.0, 27.0)
Ethnicity	Hispanic or Latino	30 (17.6%)
	NOT Hispanic or Latino	132 (77.6%)
	Not Reported	4 (2.4%)
	Unknown	4 (2.4%)
Race	American Indian or Alaskan Native	1 (0.6%)
	Asian	5 (2.9%)
	Black or African American	5 (2.9%)
	Multiracial	4 (2.3%)
	Native Hawaiian or Pacific Islander	3 (1.7%)
	Unknown	16 (9.2%)
	White	139 (80.3%)
Sex	Female	132 (76.3%)
	Male	41 (23.7%)
Gender Identity	Cis-female	64 (70.3%)
	Cis-male	17 (18.7%)
	Non-binary	4 (4.4%)
	other	5 (5.5%)
	Transgender male	1 (1.1%)
Primary diagnosis	ankylosing spondylitis	4 (2.5%)
	AoSD	3 (1.9%)
	Bechets	3 (1.9%)
	Crystal Arthropathy	2 (1.3%)
	Enthesitis Related JIA	20 (12.6%)
	inflammatory enthesitis	1 (0.6%)
	MCTD	3 (1.9%)
	Oligo extended JIA	6 (3.8%)
	Oligo persistent JIA	13 (8.2%)
	Other	7 (4.4%)
	Other Autoinflammatory	3 (1.9%)
	Polyarticular RF negative JIA	28 (17.6%)
	Polyarticular RF+ JIA	10 (6.3%)
	Psoriatic Arthritis	3 (1.9%)
	Psoriatic JIA	3 (1.9%)
	Raynaud’s	1 (0.6%)
	Rheumatoid Arthritis	21 (13.2%)
	Sjogren	2 (1.3%)
	spondyloarthritis	2 (1.3%)
	Systemic JIA	3 (1.9%)
	Systemic Lupus Erythematosus	20 (12.6%)
	Undifferentiated Connective tissue Disease	1 (0.6%)
Age at diagnosis	Mean (SD)	13.0 (4.5)
	Median (IQR)	15.0 (11.0, 16.0)
	Range	(1.0, 19.0)

**Fig. 1 (Abstract A26):**
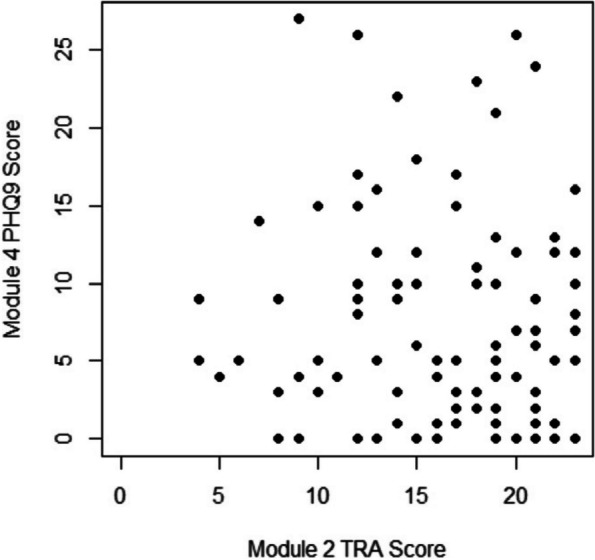
Correlation between TRA scores (Module 2) and PHQ9 scores

**Fig. 2 (Abstract A26):**
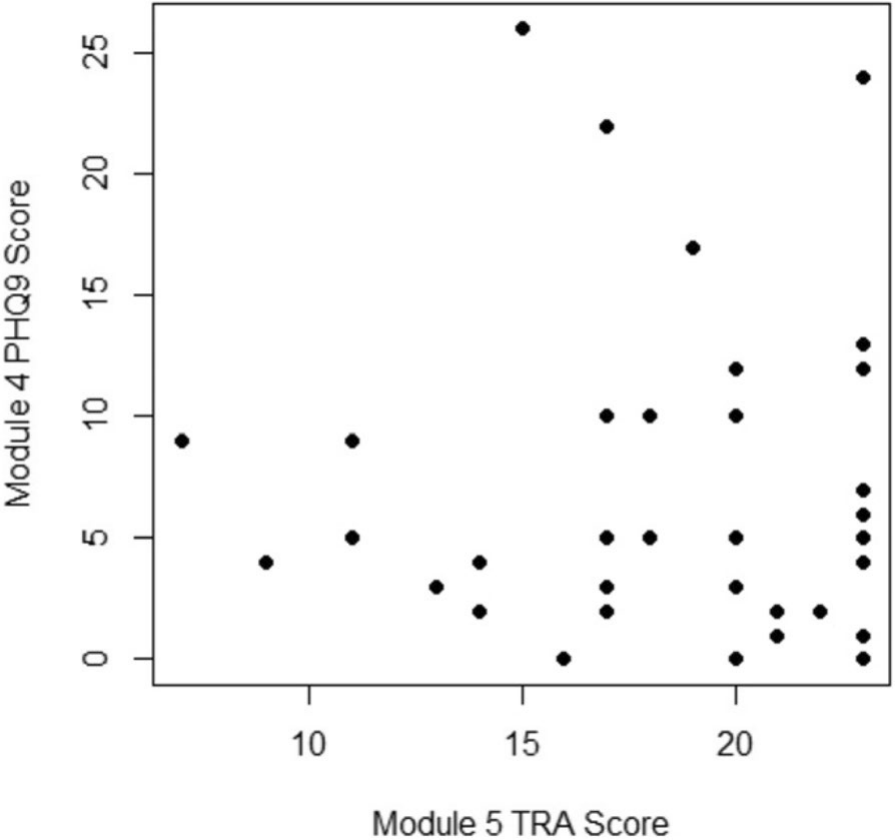
Correlation between TRA scores (Module 5) and PHQ9 scores

## A27 CARRA+PReS alignment of registry parameters to further collaborative research on childhood-onset systemic lupus erythematosus

### Rebecca Sadun^1^, Jennifer Cooper^2^, Alenxandre Belot^3^, Eve Smith^4^, Laura Lewandowski^5^

#### ^1^Duke University School of Medicine, Durham, NC, USA; ^2^University of Colorado, Aurora, CO, USA; ^3^Centre Hospitalier Universitaire de Lyon, Lyon, France; ^4^Alder Hey Children’s Hospital, Liverpool, UK; ^5^National Institute of Arthritis and Musculoskeletal and Skin Diseases, NIH, Bethesda, MD, USA

##### **Correspondence:** Rebecca Sadun

*Pediatric Rheumatology 2024*, **22(S1):**A27

Background : Childhood-onset systemic lupus erythematosus (cSLE) occurs in approximately 20% of all SLE cases. cSLE has a worse prognosis than adult-onset SLE, often requiring more aggressive therapy. CARRA coordinates a collaborative cSLE registry for patients in the US and Canada, while additional registries capture cSLE data in the United Kingdom (UK JSLE Registry) and across Europe (JIR Registry). To date, differences in data fields and collection methodology have been barriers to collaborative inter-registry research, which could enhance patient numbers and improve research quality. Through collaborative efforts between CARRA and the Paediatric Rheumatology European Society (PReS), key “Core” and “Expanded” datasets have been developed through consensus with a focus on feasibility to conduct SLE research.

Methods : SLE experts (15 pediatric rheumatologists, 4 combined pediatric and adult rheumatologists, 2 pediatric nephrologists) with international representation (10 countries; 5 continents) participated in multiple rounds of Delphi questionnaires, topic-specific taskforce meetings, and 3 virtual consensus meetings. Four patients/caregivers also attended each taskforce and consensus meeting, with votes given equal weight. Final data elements for the core and expanded datasets were determined using adapted nominal group technique and voting, with consensus requiring ≥ 80% agreement.

Results : The group achieved consensus for Core (Table 1) and Expanded (Table 2) Datasets, inclusive of the following: demographics, SLE history, co-morbidities, family history, SLE classification criteria, visit data, laboratory testing, kidney biopsy data, disease activity measurements, damage, medications, and serious adverse events. In addition, consensus was achieved regarding patient reported outcomes (PROMs) for the Expanded Dataset (Table 3) to capture fatigue, depression, anxiety, physical functioning (all using PROMIS measures) and discrimination (using the Everyday Discrimination Scale). Finally, consensus was achieved regarding frequency of data collection for each data item (baseline, 3 months + flare visits and annual timepoints).

Conclusions : Standardized Core and Expanded Datasets for registry-based international cSLE research were defined by global expert consensus. These datasets consider unique aspects of children with SLE in a range of geographic and resourced settings. The core and expanded datasets will facilitate international collaborative research for children and adolescents with SLE worldwide, inclusive of patient-oriented outcomes.

IRB Statement : This study was granted an exemption by the Duke University IRB.

Acknowledgements : The authors wish to acknowledge the CARRA-PReS Collaborative Research Award, which funded this project, as well as the ongoing support of the CARRA and PReS organizations. In addition, we wish to acknowledge the many physicians, researchers, patients, parents, and registry database managers who helped make this work possible.

**Table 1 (Abstract A27):** Core dataset

**Demographics**
Date of birth
Country of residence
Biologic sex
Gender identity
Ancestry
**SLE history**
Date of SLE diagnosis
Date of SLE symptom onset
**Family history**
SLE in first degree relatives
**SLE Classification **(collect disaggregated elements)
2012 SLICC classification criteria
2019 EULAR/ACR classification criteria
**General visit data **
Blood pressure, height, weight
For females: menarche (date) & current menses
**Disease activity**
SLEDAI-2K
Physician global assessment of disease activity
Patient/parent global assessment
**SLE-related damage**
SLICC Damage Index
**Kidney biopsy data**
Date of biopsy
ISN/RPS classification
**Laboratory Tests**
White blood cell, lymphocyte, neutrophil counts
Hemoglobin concentration
Platelet count
Serum creatinine, albumin
Erythrocyte sedimentation rate, C-reactive protein
ANA and ENA results (positive/negative)
dsDNA (titer)
Antiphospholipid antibodies (positive/negative)
IgG
Urine microscopy (RBCs, WBCs, casts)
Urine protein to creatinine ratio
**Medications**
Corticosteroids (oral and intravenous)
Immunomodulators
Antihypertensives
Anticoagulants
Lipid lowering agents
Vitamin D
Antidepressants
Contraceptives
**Serious adverse events (dates)**
Hospitalization (reason)
Death (cause)
End-stage kidney disease, dialysis, transplant
Pregnancy (date, outcome)
Malignancy (type)

**Table 2 (Abstract A27):** Additional data elements for expanded dataset

**Family history **
Primary Sjogren syndrome
Rheumatoid Arthritis
Antiphospholipid syndrome
Other rheumatic disease
**Disease activity**
BILAG-2004
**SLE-related damage**
Parental height (to identify decreased final height)
**Kidney biopsy data**
Modified NIH disease activity & chronicity scores
Presence of thrombotic microangiopathy
Percent crescents
Percent sclerosis
Presence of interstitial fibrosis & tubular atrophy
**Genetic data**
Confirmed or suspected monogenic lupus
DNA collected/sequenced: yes/no
Known or suspected mutation/variant
**Laboratory tests**
Ferritin
Direct coombs, LDH, haptoglobin
CH50
Anti-C1q antibodies
TSH
Hgb A1c
Total cholesterol, triglycerides, LDL, HDL
25-OH vitamin D
**Adverse events **
HCQ maculopathy
**Comorbidities**
Sjogren syndrome
Autoimmune thyroid disease
Other autoimmune disease (options + free-text)
Primary immunodeficiency
Tuberculosis (latent, active)
HIV
Depression

**Table 3 (Abstract A27):** Patient-Reported Outcome Measures (PROMs) for expanded cSLE dataset

Domain	Measure	Number of questions	Notes
Disease Activity	Patient/Parent Global Assessment of Disease Activity	1	Also included in core dataset
Experiences of Discrimination	Everyday Discrimination Scale SF	5	Ages 14+ years
Fatigue	PROMIS Pediatric Fatigue SF	10	Ages 8-18 years
Global Health	PROMIS Pediatric Global Health	7	Ages 8-18 years
Anxiety	PROMIS Pediatric Anxiety SF^a^	8	Ages 8-18 years
Depression	PROMIS Pediatric Depression SF^a^	8	Ages 8-18 years

*PROMIS* Patient Reported Outcomes Measurement Information System, *SF* Short Form

^a^Recommend use of anxiety and depression PROMs in settings where real-time routine mental health screening is integrated into clinical care. Anxiety and depression PROMs *should not* replace standard mental health screening tools

## A28 Facilitating collaborations in pediatric localized scleroderma research: international validation of outcome measures

### Christina Zigler^1^, Clare Pain^2^, Debra Henke^1^, Hanna Lythgoe^3^, Kaveh Ardalan^1^, Kathryn Torok^4^, Suzanne Li^5^

#### ^1^Duke University School of Medicine, Durham, NC USA; ^2^Alder Hey Children’s NHS Foundation Trust, Liverpool, England; ^3^Royal Manchester Children’s Hospital, Manchester, UK; ^4^University of Pittsburgh Medical Center, Children’s Hospital of Pittsburgh, Pittsburgh, PA, USA; ^5^Joseph M Sanzari Children’s Hospital, Hackensack Meridian School of Medicine, Hackensack, NJ, USA

##### **Correspondence:** Christina Zigler

*Pediatric Rheumatology 2024*, **22(S1):**A28

Background : Juvenile localized scleroderma (jLS) is a rare condition that impairs health-related quality of life (HRQoL) through inflammation and fibrosis of the skin and underlying tissues as well as extracutaneous sites. Recent studies demonstrate extracutaneous manifestations (ECMs) occur in over half of jLS patients and are associated with poorer HRQoL, but current measures fail to capture this complexity, limiting our understanding of the full impact on individuals with jLS. Thus, there is a critical need for coordinated, international partnerships to effectively validate and implement these novel measures.

Methods : Our overall objective is to advance global collaborations between the Childhood Rheumatology Research Alliance (CARRA), Paediatric Rheumatology European Society (PReS), and Paediatric Rheumatology INternational Trials Organisation (PRINTO) to improve jLS outcomes via 3 aims. AIM 1: Internationally validate the Localized Scleroderma Quality of Life Instrument (LoSQI), a jLS-specific patient-reported HRQoL measure. AIM 2: Refine weighting of items in two of the Localized scleroderma Total Severity Score (LoTSS) modules with an international group of jLS experts. AIM 3: Generate the first prospective global jLS dataset in order to evaluate the psychometric properties of outcome measures for use in jlS (LoSQI, LoTSS, and the Patient Reported Outcomes Measurement Information System (PROMIS®)).

Results : An update on all 3 aims can be found in Table 1. We anticipate the project will be completed by the end of the project period, March 1, 2024.

Conclusions : Upon completion of the project, the expected outcomes will be: 1) validity evidence supporting the use of multilingual versions of the LoSQI and PROMIS® measures for assessing disease-specific and generic HRQoL in individuals with jLS; 2) refined weighting of two LoTSS module items allowing for improved standardized scoring of ECMs; and 3) an integrated international collaborative research network with the first combined dataset spanning CARRA, PReS, and PRINTO. These efforts will support future studies that assess the effectiveness of treatments on the full spectrum of disease impact, in keeping with CARRA’s mission and vision to conduct collaborative research and work towards a world free of limitations from pediatric rheumatic diseases.

IRB Statement : All study procedures were performed in accordance of the Declaration of Helsinki; aims 1 and 3 were determined exempt, while study procedures in Aim 2 were approved by the Duke University IRB (Pro00108843).

Acknowledgements : The authors wish to acknowledge CARRA and the ongoing Arthritis Foundation financial support of CARRA. This work is supported by a CARRA-Arthritis Foundation Large Grant (project period 03/01/2021 – 02/28/2023). We would like to thank PReS/PRINTO for their collaboration and input into this work and the investigators who volunteered to participate in the Delphi process (Aim 2).

**Table 1 (Abstract A28):** Update on the project for 2023

Aim	Status
AIM 1: Internationally validate the Localized Scleroderma Quality of Life Instrument (LoSQI), a jLS-specific patient-reported HRQoL measure	The study team has reviewed and provided feedback on the back-translations for 21 languages, and reviewed summaries of cognitive interview data for 3 languages. 5 countries are still enrolling for the probe phase.
AIM 2: Refine initial weighting of items in two of the modules of the Localized scleroderma Total Severity Score (LoTSS) with an international group of jLS experts.	Three rounds of the Delphi process have been completed. Refined weighting of the LoTSS Craniofacial/Neurologic and Other Organ modules have been defined. We will utilize the quantitative data described in Aim 3 to analyze psychometric properties of the new weighting scheme.
AIM 3: Generate the first prospective global jLS dataset in order to evaluate the psychometric properties of novel jLS outcome measures (LoSQI, PROMIS, TMS).	International data is currently being collected by Associate Prof Clare Pain via PReS/PRINTO. 68 participants have currently been enrolled across 11 sites. SCORE data is collected and includes data from upwards of 50 pediatric patients. A data use agreement is in progress.

## A29 Evaluation of a tool to enhance training of the physical examination of the temporomandibular joint (TM Joint) in juvenile idiopathic arthritis (JIA)

### Tova Ronis^1^, Nancy Pan^2^, Rebecca Sadun^3^, Melissa Lerman^4^, Cory Resnick^5^, James Bost^6^, Peter Stoustrup^7^, Marinka Twilt^8^, for the CARRA Registry Investigators^9^

#### ^1^Children’s National Hospital, George Washington University, Washington, DC, USA; ^2^Weill Cornell Medical College, Hospital for Special Surgery, New York, NY; ^3^Duke University School of Medicine, Durham, NC, USA; ^4^University of Pennsylvania, Children’s Hospital of Philadelphia, Philadelphia, PA, USA; ^5^Harvard Medical School, Boston, MA, USA; ^6^Children’s National Hospital, Washington, DC, USA; ^7^Aarhus University, Aarhus, Denmark; ^8^University of Calgary, Alberta Children’s Hospital, Calgary, AB, Canada; ^9^Childhood Arthritis and Rheumatology Research Alliance (CARRA)

##### **Correspondence:** Tova Ronis

*Pediatric Rheumatology 2024*, **22(S1):**A29

Background : Arthritis of the TM joint is a frequent finding in patients with JIA.2 Orofacial examination enables early detection of TM joint involvement. The TM Joint Juvenile Arthritis Work Group published a standardized physical exam (article) to assess for JIA-induced orofacial manifestations.2 The aim of the study was to demonstrate the effectiveness of a novel e-learning module in teaching the physical exam of the TM joint in JIA.

Methods : A 20-minute e-learning module consisting of videos and interactive questions was created. Pediatric rheumatology fellows were randomized, and fellows were stratified by post-graduate year. One group received the article while the second group received both the article and access to the e-learning module. All participants completed a written pre-test before the learning intervention and then underwent both: an in-person objective structured clinical examination (OSCE) during the Childhood Arthritis and Rheumatology Research Alliance Scientific Meeting in March 2023; and a written post-test. The maximum OSCE score was 18. Crosstab tables and Chi-squared tests were used to assess categorical variables across groups. Wilcoxon Rank Sum tests were used for continuous measures due to the small sample size.

Results : Twenty-two pediatric rheumatology fellows enrolled, with 11 in each group. Both reported an equal amount of time spent preparing for the OSCE. Written test: The two groups performed equally. There was a trend toward significance in defining maximal incisal opening (MIO) on the written test in the module group. Both groups had a trend towards improvement in recognition of patient profiles and facial asymmetry. OSCE: The mean OSCE score was 11.1 (SD 3.3) in the article group and 13.5 (SD 1.9) in the module group, with a trend towards significance (*p*=0.059). Significant differences were seen on the OSCE in learning domains related to measuring MIO, calculating maximal unassisted mouth opening (MUMO), and assessment of facial symmetry. There were no differences in other domains (see Table 1). Confidence in TM joint exam skills was increased with no difference between groups (Figure 1). Enjoyment scores in the module group were higher than in the article group (mean 7.7/10 vs 5.9/10, *p*=0.017) (Figure 2). Follow up: Three months later, the TM joint exam was performed “often” or “most of the time” in 27% in the article only group and 45.5% in the module group, although this difference was not statistically significant. All participants in the module group were moderately or very satisfied with the learning intervention compared with 73% in the article group.

Conclusions : This study demonstrated effectiveness of a novel e-learning module in teaching the physical exam of the TM joint in JIA. Learners who viewed the module were more adept at obtaining quantitative TM joint measurements than those who read the article. Both groups showed improvement in overall skill and confidence level, although the module group enjoyed the learning experience more.

IRB Statement : Exemption was obtained from the Children’s National Hospital IRB.

Acknowledgements : This project is supported by both a George Washington University SMHS Education Research Grant as well as a CARRA-Arthritis Foundation small grant. The authors wish to acknowledge the Arthritis Foundation for their ongoing financial support of CARRA.

**Table 1 (Abstract A29):** OSCE scores by learning group. *OSCE* objective structured clinical examination, *MUMO* maximal unassisted mouth opening

	Module Mean (SD)	Article Mean (SD)	*P* value (Wilcoxon Rank Sum Test)
OSCE total score	13.54 (1.91)	11.09 (3.27)	0.059
History taking	3.09 (0.94)	2.82 (1.08)	0.581
Palpation	2 (0.63)	2.27 (0.90)	0.214
Mandibular deviation	2.54 (1.13)	2.09 (1.37)	0.418
Measurements	4.09 (1.38)	2.36 (1.21)	**0.005**
MUMO calculation	1.45 (0.93)	0.36 (0.81)	**0.012**
Symmetry	1 (0)	0.63 (0.50)	**0.030**
Profile	0.81 (0.40)	0.91 (0.30)	0.543

**Fig. 1 (Abstract A29):**
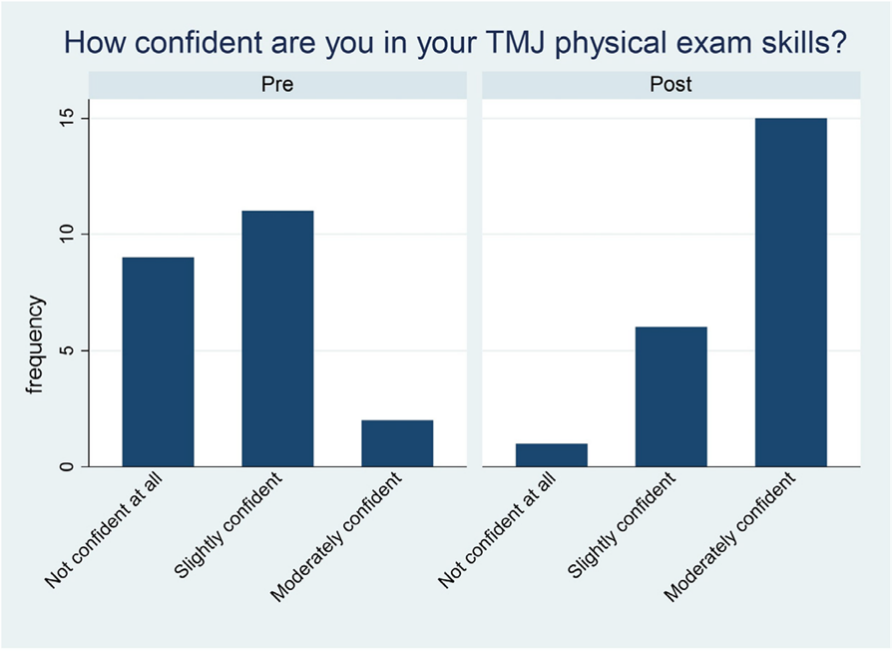
Self-reported confidence in TM Joint physical exam skills before and after the learning intervention for all participants. No difference was noted between groups

**Fig. 2 (Abstract A29):**
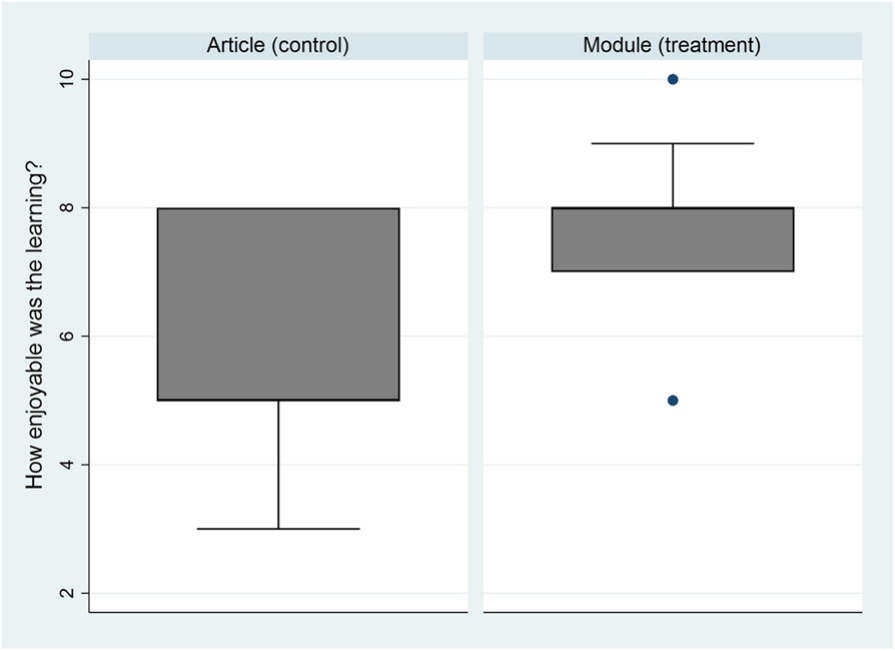
Participant response to “How enjoyable was the learning?”

## A30 Pathogenesis of muscle weakness in 3D muscle model: investigating theories for juvenile dermatomyositis

### Lauren Covert^1^, Anna Demelo^2^, George Truskey^2^

#### ^1^Duke University School of Medicine; ^2^Duke University Pratt School of Engineering

##### **Correspondence:** Lauren Covert

*Pediatric Rheumatology 2024*, **22(S1):**A30

Background : A 3D skeletal muscle model is useful to investigate pathogenic theories for juvenile dermatomyositis (JDM), a condition associated with upregulated type I interferon [1, 2] and mitochondrial dysfunction [3]. Using human-derived pediatric bioengineered muscle (myobundles), we examined the effects of the following compounds on muscle function: antimycin A (AMA), mitotempo (mitoT), and polyinosinic:polycytidylic acid (poly I:C). AMA is an electron transport chain complex III inhibitor that favors reactive oxygen species production, whereas mitoT is a mitochondrially targeted antioxidant that scavenges superoxide. Poly I:C is a TLR3 ligand, synthetic double-stranded RNA that mimics viral infection and driver of type I interferon. Hypothesis: AMA and poly I:C have a detrimental effect on myobundle contractile force, yet mitoT ameliorates AMA effects.

Methods : Myogenic cells isolated from three healthy pediatric donors were cultured and used to create myobundles based on established protocols [4] (Fig. 1). After maturation, myobundles were exposed to 0 (control condition), 5 nM AMA, 10 uM MitoT, 5 nM AMA plus 10 uM MitoT, or 10 ug/mL poly I:C in differentiation media every other day for 7 days. Contractile force and kinetics of myobundles were measured after electrical stimulation (Twitch: 1 stimulation at 1 Hz for 10 ms; Tetanus: 20 stimulations at 20 Hz for 1 s; Fatigue: 600 stimulations at 20 Hz for 30 s). Data across treatment groups was analyzed using one-way ANOVA with multiple post hoc comparisons.

Results : AMA and mitotempo alone decreased twitch and tetanus force. MitoT alone also decreased twitch time to half-relax. Combination AMA and mitoT enhanced the effects of each compound alone, decreasing twitch and tetanus force further and decreasing time to half-relax. Combination treatment also increased fatigue. Poly I:C decreased twitch and tetanus force in myobundles in 2 out of 3 donors and decreased fatigue across all donors. (Fig. 2)

Conclusions : AMA and mitotempo independently decrease contractile muscle force, indicating the importance of cellular respiration and ROS in muscle function, respectively. When in combination, AMA and mitotempo have an additive detrimental effect on muscle contractile force and fatigue. Poly I:C leads to donor-dependent skeletal muscle response, yet may be associated with decreased force production and increased muscle fatigue. Bioengineered pediatric muscle can elucidate pathways of muscle weakness in JDM.

IRB Statement : Sample collection occurred under Duke University IRB approved protocols (00009025).

References

1. Moneta, G.M., et al., Muscle Expression of Type I and Type II Interferons Is Increased in Juvenile Dermatomyositis and Related to Clinical and Histologic Features. Arthritis Rheumatol, 2019. 71(6): p. 1011-1021
2. Baechler, E.C., H. Bilgic, and A.M. Reed, Type I interferon pathway in adult and juvenile dermatomyositis. Arthritis Res Ther, 2011. 13(6): p. 249
3. Gonzalez-Chapa, J.A., M.B. Macêdo, and C. Lood, The Emerging Role of Mitochondrial Dysfunction in the Pathogenesis of Idiopathic Inflammatory Myopathies. Rambam Maimonides Med J, 2023. 14(2)
4. Covert, L.T., et al., Effect of type I interferon on engineered pediatric skeletal muscle: A promising model for juvenile dermatomyositis. Rheumatology, 2023.

**Fig. 1 (Abstract A30):**
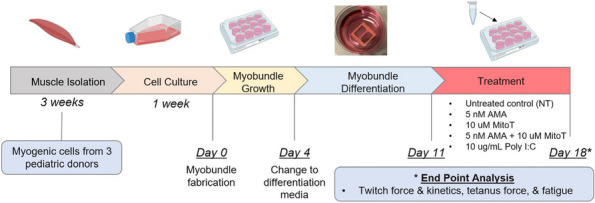
Experimental schema

**Fig. 2 (Abstract A30):**
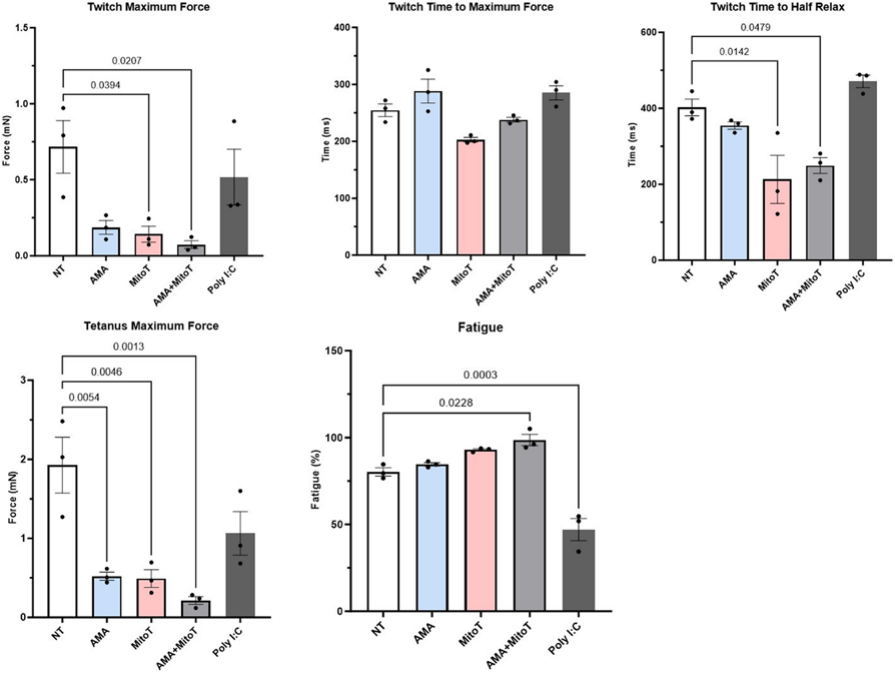
Contractile force and twitch kinetics data shown for *N*=3 donors, 5-6 myobundles were averaged per donor. NT = non-treated, AMA = 5 nM antimycin A, MitoT = 10 μM mitotempo, Poly I:C = 10 μg/mL Poly I:C

## A31 Treatment barriers in patients with juvenile idiopathic arthritis

### Julia Harris, Leslie Favier, Michael Holland, Emily Fox, Jordan Jones, Cara Hoffart, Ashley Cooper

#### Children’s Mercy Kansas City

##### **Correspondence:** Julia Harris

*Pediatric Rheumatology 2024*, **22(S1):**A31

Background : Most patients with juvenile idiopathic arthritis (JIA) require scheduled anti-inflammatory and immunosuppressive medications to treat their disease. Patient-reported barriers have been shown to directly correlate with treatment adherence rates. Identification of treatment barriers offers a valuable opportunity for providers to implement patient-specific self-management support.

Methods : Our site routinely offers a treatment barriers assessment form as part of our self-management support. Initially, patients/parents completed a paper form at their clinic visit, but in August 2023, an electronic version was implemented. A chart review of follow-up patients with JIA was performed in August and September 2023 to identify use of the barriers assessment form, detect which form was utilized, determine percent of visits with a treatment barrier present, and identify which treatment barriers were selected.

Results : There were 265 charts reviewed over the 2-month period, and 81.5% (*n*=216) of patients with JIA were on scheduled anti-inflammatory and/or immunosuppressive medication (Figure 1). The barriers assessment form was completed in 47.7% (*n*=103) of the visits; of those visits, 64.1% (*n*=66) of the forms were completed electronically. Of those who completed a barriers assessment form, 40.8% (*n*=42) identified at least 1 barrier with a total of 121 barriers. Of the visits with barriers identified, most (73.8%) reported barriers to injectable medication, 47.6% had barriers to oral medication, and none (0.0%) reported barriers to infusion medication. Table 1 highlights the individual treatment barriers noted. The most common treatment barriers included treatment is painful (*n*=17), dislike side effects (*n*=14), and forget to do treatment (*n*=14).

Conclusions : Assessing for treatment barriers is an important first step in providing tailored self-management support for patients with JIA. Treatment barriers are common in patients with JIA on injectable and oral anti-inflammatory and/or immunosuppressive medications. There was a wide variety of reported barriers. Our next steps include a quality improvement project to improve consistent use of the treatment barriers assessment form and standardization of the implementation of adherence solution tools.

IRB Statement : Per hospital policy, quality improvement projects do not require review by the institutional review board as they are not considered human subjects research.

**Table 1 (Abstract A31):** Treatment barriers identified in follow-up JIA visits

Barrier	Visit with barrier identified (***n***=42)^**a**^
Treatment is painful	17 (40.5%
Dislike side effects	14 (33.3%)
Forget to do treatment	14 (33.3%)
Makes me uncomfortable or upset	12 (28.6%)
Dislike the taste	10 (23.8%)
Run out of medication	9 (21.4%)
Pills hard to swallow	8 (19%)
Worry about future side effects	7 (16.7%)
Feel treatment is not needed	6 (14.3%)
Refuse to do treatment	5 (11.9%)
Do not want others to know	5 (11.9%)
Treatment is inconvenient	4 (9.5%)
May impact ability to have children in the future	4 (9.5%)
Gets in the way of other activities	3 (7.1%)
Treatment does not work	2 (4.8%)
Treatment is too expensive	1 (2.4%)
Instructions hard to follow	0 (0%)

^a^Visits can have ≥1 barrier identified

**Fig. 1 (Abstract A31):**
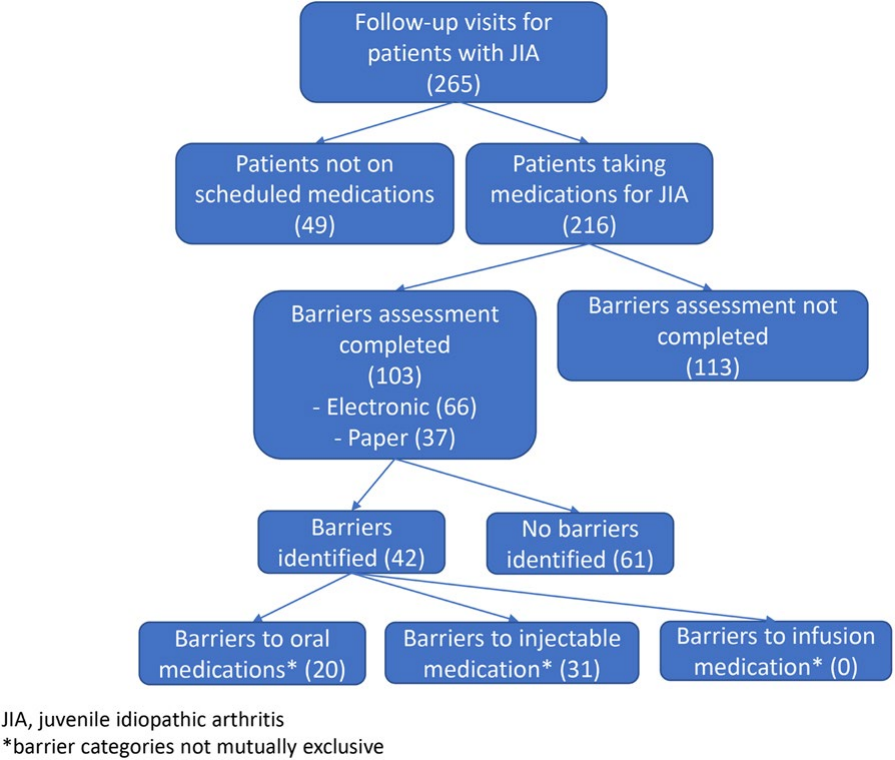
See text for description.

## A32 Patient- and center-level risk factors for research lost to follow-up using the childhood arthritis and rheumatology research alliance (CARRA) registry

### Monica Aswani^1^, Livie Huie^1^, Kristine Hearld^1^, Melissa Mannion^1^, Emily Smitherman^1^, for the CARRA Registry Investigators^2^

#### ^1^University of Alabama at Birmingham, Birmingham, AL, USA; ^2^Chilhood Arthritis and Rheumatology Research Alliance (CARRA)

##### **Correspondence:** Monica Aswani

*Pediatric Rheumatology 2024*, **22(S1):**A32

Background : Clinical registries are typically envisioned to be representative of a target patient population and reflective of health care delivery practices for said population. Variation in clinical practices by different sites and concerns related to recruitment/retention can have profound implications for the validity of data collected and extent to which it may be impacted by selection bias. Thus, the objective of this study is to assess the role of patient- and site-level factors associated with research attrition in the Childhood Arthritis and Rheumatology Research Alliance (CARRA) Registry.

Methods : Patients enrolled in the CARRA Registry from 2015 to 2019 were eligible. The effects of patient (level 1) and site (level 2) factors on lost to follow-up were assessed using a 2-level logistic model, followed by empirical Bayes estimation to investigate cross-center variation. Sites classify patients as inactive if they are referred elsewhere, move to a non-CARRA site, transition to adult rheumatology, achieve remission, or are lost to follow-up. We defined our outcome of interest as patients lost to follow-up (= 1) versus all other patients (=0), who may be active or inactive due to other reasons. The empirical Bayes estimates are used to graphically illustrate variation in lost to follow-up rates between unadjusted and adjusted models (Figure 1).

Results : The final analytic sample contained 9,730 patients nested within 69 sites (Table 1). Most of the sample was female (*n*=6,481, 72%), White (*n*=6,403, 64%), and privately insured (*n*=5,559, 62%). Overall, 728 (7.5%) patients in the cohort were lost to follow-up. The intra-class correlation coefficient (ICC) from null model signified that 20.3% of the variance in lost to follow-up is at the registry site level. Percent of missed Registry visits (OR 1.09, 95% CI: 1.086, 1.12), non-Medicaid state insurance (OR 1.95, 95% CI: 1.15, 3.12), and an income between $25,000 and $49,999 (OR 1.52, 95% CI: 1.003, 2.31) were significantly associated with higher odds of lost to follow-up (Figure 2).

Conclusions : Significant variation in research participant lost to follow-up exists at the site level. Moreover, individual-level characteristics such as visit history, insurance status, and household socioeconomic status were associated with the likelihood of being lost to follow-up. This non-random attrition may also highlight concerns related to patient retention in clinical practice.

IRB Statement : This study was approved by the University of Alabama at Birmingham Institutional Review Board (IRB-170112004).

Acknowledgements : This study was supported by a CARRA-Arthritis Foundation Health Equity Research Grant. This work could not have been accomplished without the aid of the following organizations: The NIH’s National Institute of Arthritis and Musculoskeletal and Skin Diseases (NIAMS) & the Arthritis Foundation. We would also like to thank all participants and hospital sites that recruited patients for the CARRA Registry. The authors thank the following CARRA Registry site principal investigators: K. Abulaban, C. Aguiar Lapsia, S. Ardoin, L. Barillas-Arias, M. Basiaga, K. Baszis, H. Brunner, H. Bukulmez, E. Chalom, J. Chang, D. Co, K. Cook, A. Cooper, C. Correll, T. Davis, F. Dedeoglu, M. DeGuzman, A. Dhanrajani, K. Ede, B. Edelheit, B. Feldman, I. Ferguson, D. Glaser, D. Goldsmith, B. Gottlieb, T. Graham, T. Griffin, T. Hahn, L. Harel, O. Harry, M. Hollander, S. Hong, M. Horwitz, J. Hsu, A. Huber, L. Imundo, C. Inman, P. Kahn, S. Kim, D. Kingsbury, M. Klein-Gitelman, L. Lim, M. Mannion, D. McCurdy, D. Milojevic, S. Mohan, T. Moore, K. Moore, L. Moorthy, S. Nativ, M. Natter, K. Onel, J. Patel, S. Prahalad, C. Rabinovich, A. Robinson, T. Ronis, M. Rosenkranz, N. Ruth, S. Sabbagh, K. Schikler, C. Schutt, E. Sloan, J. Spitznagle, Y. Sterba Rakovchik, K. Stewart, G. Syverson, S. Tarvin, M. Tesher, D. Toib, M. Toth, M. Twilt, H. Van Mater, D. Wahezi, P. Weiss, J. Weiss, L. Woolnough, E. Wu, A. Yalcindag, Y. Zhao

**Fig. 1 (Abstract A32):**
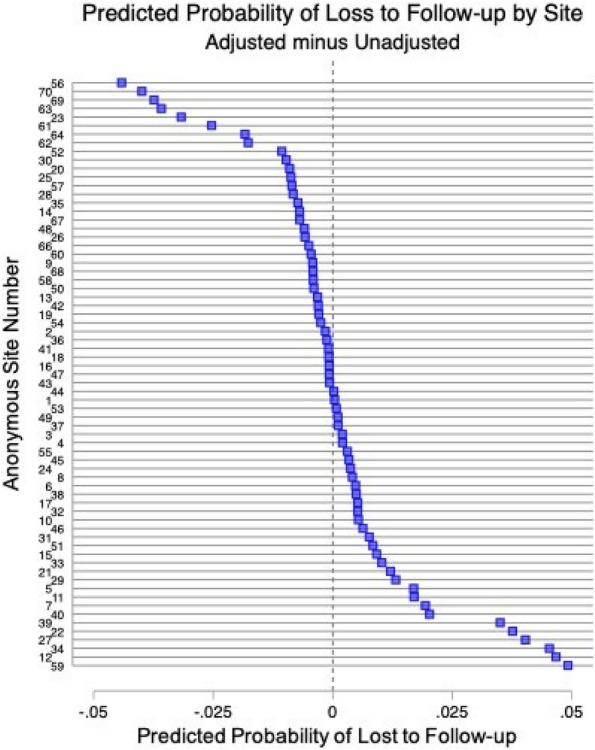
Site-level Variation. Sites with a negative difference (i.e., higher unadjusted predicted probability, to the left of zero) means controlling for patient- and site-level factors made their lost to follow-up look more favorable. Sites with a positive difference (i.e., higher adjusted predicted probability, to the right of zero) means controlling for patient- and site-level factors made their lost to follow-up look worse off

**Fig. 2 (Abstract A32):**
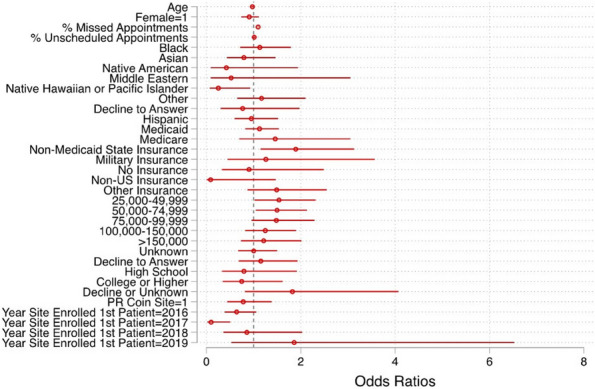
Multilevel Logistic Regression. Reference categories include: Male, White race, private insurance, less than high school, non-PR-COIN site, and site started CARRA enrollment in 2015

**Table 1 (Abstract A32):** Descriptive statistics (*N* = 9730)

	Not Lost to Follow-up		Lost to Follow-up	
	Mean/Prop.	SD/Freq.	Mean/Prop.	SD/Freq.
Lost to Follow-up	.000	9002	1.000	728
Age	11.540	4.795	12.341	4.837
Female	.720	6483	.668	486
% Missed Appointments	6.964	14.410	47.449	18.756
% Unscheduled Appointments	1.674	6.407	.755	3.699
*Race/Ethnicity*				
White	.711	6403	.720	524
Black	.064	575	.088	64
Asian	.046	410	.027	20
Native American	.013	115	.008	6
Middle Eastern	.006	56	.004	3
Native Hawaiian or Pacific Islander	.006	54	.003	2
Other	.016	140	.023	17
Decline to Answer	.019	175	.011	8
Hispanic	.119	1074	.115	84
*Insurance*				
Private Insurance	.618	5559	.635	462
Medicaid	.195	1755	.232	169
Medicare	.021	186	.021	15
Non-Medicaid State Insurance	.025	229	.029	21
Military Insurance	.016	146	.014	10
Indian Health Insurance	.012	108	.016	12
No Insurance	.048	435	.003	2
Non-US Insurance	.065	584	.051	37
Other Insurance				
*Income*				
<25,000	.079	715	.092	67
25,000-49,999	.117	1054	.143	104
50,000-74,999	.117	1056	.137	100
75,000-99,999	.106	957	.117	85
100,000-150,000	.167	1499	.120	87
>150,000	.151	1363	.106	77
Unknown	.134	1209	.183	133
Decline to Answer	.128	1149	.103	75
*Education*				
Less than High School	.037	332	.018	13
High School	.133	1193	.076	55
College or Higher	.611	5503	.266	194
Decline or Unknown	.219	1974	.640	466
PR Coin Site	.414		.492	
*Year Site Enrolled 1st Patient*				
2015	.493	4438	.613	446
2016	.462	4157	.368	268
2017	.019	170	.003	2
2018	.019	170	.015	11
2019	.007	67	.001	1

## A33 Health literacy in childhood-onset systemic lupus erythematosus

### McKenzie Vater, Alaina Davis, Sarah Jaser

#### Vanderbilt University Medical Center, Nashville, TN USA

##### **Correspondence:** McKenzie Vater

*Pediatric Rheumatology 2024*, **22(S1):**A33

Background : The degree of healthy literacy in childhood-onset systemic lupus erythematosus (cSLE) patients is poorly understood. Additionally, how health literacy relates to medication adherence and psychosocial outcomes in this high-risk population has not been well described. The objective of this study was to evaluate health literacy in relation to medication adherence among this patient population.

Methods : Adolescents aged 10-24 with cSLE (*n*=48) completed questionnaires that included health literacy screening using the Brief Healthy Literacy Screen (BHLS) and the Newest Vital Sign (NVS), as well as medication adherence using the Medication Adherence Self-Report Inventory (MASRI).

Results : Inadequate health literacy, defined as a BHLS score less than or equal to 9 or NVS score < 4, was common in this population. In our sample, 67% of adolescents reported inadequate health literacy on the BHLS, while 42% reported inadequate health literacy on the NVS. Medication non-adherence was reported by 26% of adolescents. In bivariate correlations, higher medication adherence was associated with a higher BHLS score (*r*=.36, *p*=0.017) but not with the NVS score (*r*=.06, *p* = .724). We observed no significant differences in BHLS scores related to gender, age, race/ethnicity, insurance type, patient education level, parent education level or household income. However, there was a notable difference in the NVS as it related to race/ethnicity. In African Americans, the NVS mean score was 3.2 compared to 4.2 in White/Other race and ethnicity, t(1, 41) = -2.36, *p* = .023). Additionally, there was a notable difference in health literacy related to income; adolescents with an annual household income >$100,000 reported significantly higher health literacy (mean NVS = 4.8) than those from lower-income households (mean NVS = 3.4), t(1, 29) = 2.28, *p* = .030).

Conclusions : Among the patients studied in our cohort, inadequate health literacy was prevalent among adolescents and young adults with cSLE. Adequate health literacy is associated with higher medication adherence. These findings suggest that screening for low health literacy and providing additional education and support may improve medication management and outcomes in this patient population. It is well known that patients in minority groups with cSLE experience higher disease burden and disproportionately have more significant morbidity and mortality. If there is higher likelihood of having inadequate health literacy in this minority group, and those with lower SES, more attention could be focused on these patient populations to continue to combat pre-existing health inequities.

IRB Statement : The institution review board approved this study. IRB #230076.

## A34 Assessment of ophthalmology education in pediatric rheumatology: a CARRA survey of pediatric rheumatology fellows

### Jully Padam^1^, Tzielan Lee^1^, Rajdeep Pooni^2^, Dana Gerstbacher^1^, Quan Dong Nguyen^1^, Sheila T. Angeles-Han^3^, for the CARRA Investigators^4^

#### ^1^Stanford University, Palo Alto, CA, USA; ^2^Stanford Children’s Health, Stanford University School of Medicine; ^3^Cincinnati University; ^4^Childhood Arthritis and Rheumatology Research Alliance (CARRA)

##### **Correspondence:** Jully Padam

*Pediatric Rheumatology 2024*, **22(S1):**A34

Background : There has not been much literature around ophthalmology training in non-ophthalmology specialties. Chan et al. found that while medical school training meets the International Council of Ophthalmology Task Force recommendations family medicine residents were uncomfortable in handling sight-threatening ocular conditions. (PMID: 21468339).No studies assessing ophthalmology education in rheumatology trainees were found. This cross-sectional survey assessed the current level of education in CARRA affiliated pediatric rheumatology fellows in basic eye examination and understanding eyesight threatening diseases common in rheumatology. Interdisciplinary work with ophthalmologists as well as identifying potential educational gaps and adequacy of further ophthalmology teaching and development of a pilot educational curriculum. We hypothesize that improving fellows’ skills and knowledge could reflect in better visual outcomes for patients.

Methods : We sent a self-developed electronic survey to 84 pediatric rheumatology fellows affiliated with CARRA. The Qualtrics survey was approved by Stanford University School of Medicine Institutional Review Board (IRB). The survey was divided in 4 sections (1)level of ophthalmology training,(2) assessment level of confidence performing eye examination excluding slit lamp and confidence screening for eye manifestations of rheumatologic diseases,(3) communication with ophthalmology and (4)gauging level of interest for further training in this area. Qualitative data analysis was done with chi square statistics.

Results : In total, 46 of 84 pediatric rheumatology fellows (55%) responded to the survey and 2% declined to participate in the study. Only 50% of the fellows reported receiving ophthalmology education in medical school (Figure 1). No more than 35% reported receiving training as a resident or as a fellow trainee with one-time lecture. 65% of participants reported that their current training programs do not have an educational curriculum and 81% do not provide a standardized approach for teaching the eye examination in their clinics (Figure 2). The percentage of participants that feel confident to perform a basic eye examination in JIA patients are only 6% (Figure 4). 51% of the participants reported to be somewhat prepared but would prefer someone available to consult regarding clinical eye findings (Figure 3). 78% of participants agree with the importance of developing their clinical ophthalmology evaluation skills and would participate in further educational opportunities available for training.

Conclusions : The majority of Ophthalmology training is received in medical school. Fewer opportunities were available in residency and fellowship programs (Figure 2). One third of trainees received their education as a one-time lecture. This result correlates with the low level of confidence in pediatric rheumatology fellows performing eye examination and recognizing eye findings seen in rheumatologic diseases. Developing an Ophthalmology educational curriculum for pediatric rheumatology trainees is a necessity. Our survey identified that current Pediatric rheumatology fellows would participate and have time available for such a training.

IRB Statement : STANFORD UNIVERSITY Stanford, CA 94305 [Mail Code 5579] David D Oakes, M.D. CHAIR, PANEL ON MEDICAL HUMAN SUBJECTS (650) 723-4550 (650) 725-8013 Date: May 22, 2023 To: Jully Munoz Padam, MD, Dean’s Office Operations - Dean Other From: David D Oakes, M.D., Administrative Panel on Human Subjects in Medical Research Tzielan Chang Lee MD, Dana Gerstbacher MD, Rajdeep Pooni MD eProtocol eProtocol #: IRB Pediatric Rheumatology Eye Survey 68214 61) The IRB reviewed your research protocol on May 22, 2023 and determined that the only involvement of human subjects in the research activities will be in one or more of the categories that are exempt from the regulations at 45 CFR 46 or 21 CFR 56. If this protocol is used in conjunction with any other human use it must be re-reviewed. The IRB requests prompt notification of any complications or incidents of noncompliance which may occur during any human use procedure. David D Oakes, M.D., Chair Review Type: EXEMPT - NEW Funding: None Assurance #: FWA00000935 (SU) Please remember that all data, including all signed consent form documents, must be retained for a minimum of three years past the completion of this research. Additional requirements may be imposed by your funding agency, your department, HIPAA, or other entities. (See Policy 1.9 on Retention of and Access to Research at http://doresearch.stanford.edu/policies/research-policy-handbook) Notice Of Exempt Review Exempt Under Category: 2

Acknowledgements : Stanford University Pediatric Rheumatology - LPCH. Stanford University Uveitis and Ocular Inflammation Byers Eye Institute. The authors wish to acknowledge CARRA and the ongoing Arthritis Foundation financial support of CARRA.

**Fig. 1 (Abstract A34):**
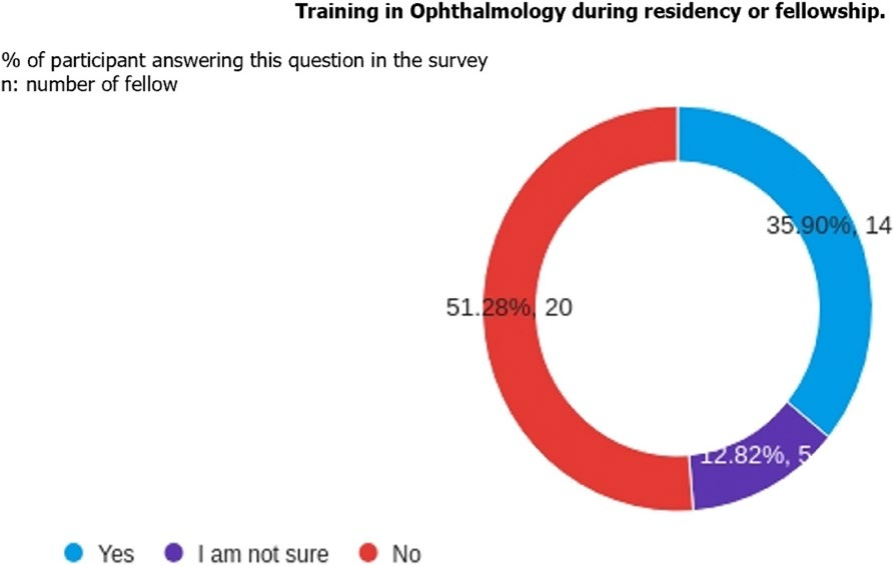
See text for description.

**Fig. 2 (Abstract A34):**
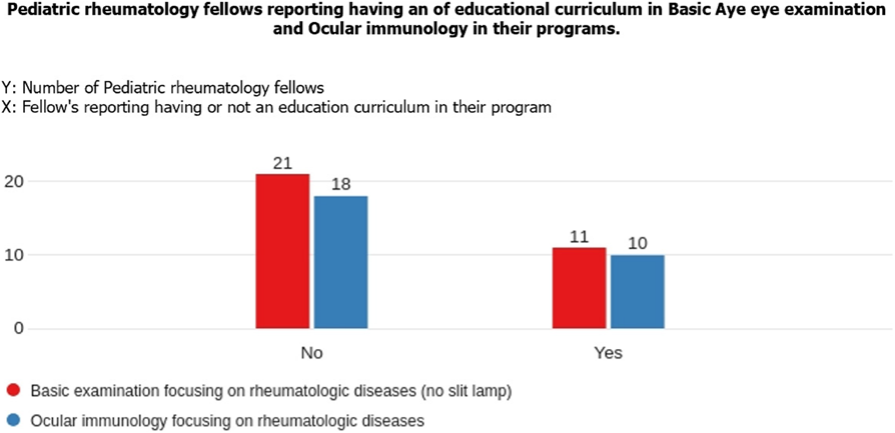
See text for description.

**Fig. 3 (Abstract A34):**
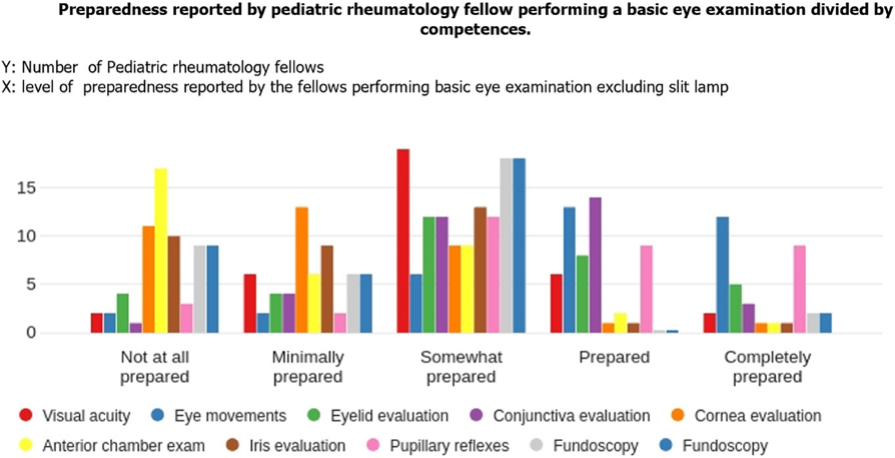
See text for description.

**Fig. 4 (Abstract A34):**
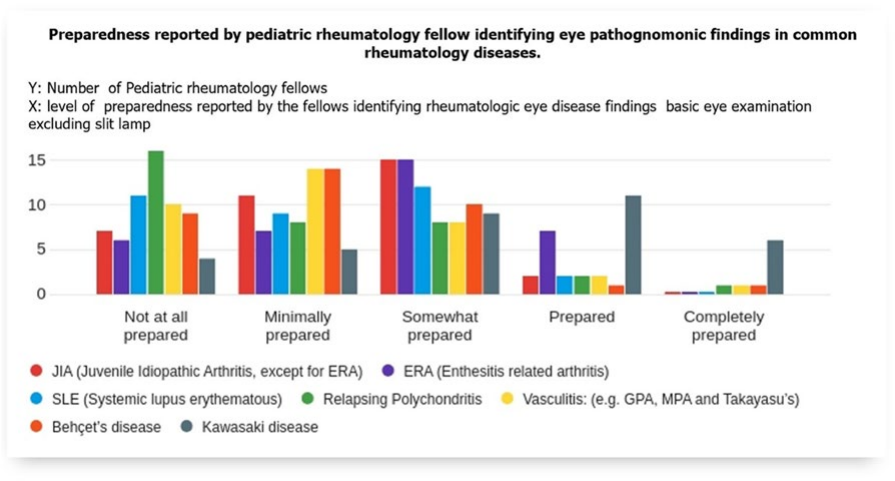
See text for description.

## A35 Disease-specific DNA methylation profiles in CD4+T cells separate psoriatic JIA from ERA-JIA patients promising potential as biomarkers

### Ana Carvalho^1^, Amandine Charras^1^, Emil Carlsson^1^, Liza McCann^2^, Clare Pain^2^, Polly Ferguson^3^, Christian Hedrich^4^

#### ^1^Institute of Life Course and Medical Sciences, University of Liverpool, Liverpool, UK; ^2^Alder Hey Children’s NHS Foundation Trust, Liverpool, England; ^3^University of Iowa; ^4^Department of Women’s and Children’s Health, University of Liverpool

##### **Correspondence:** Christian Hedrich

*Pediatric Rheumatology 2024*, **22(S1):**A35

Background : Juvenile idiopathic arthritis (JIA) covers seven subtypes, including enthesitis-related (ERA-) and psoriatic (ps)JIA. Correct classification can be difficult because of overlapping clinical presentations, variable disease courses and complex criteria. Diagnostic delay and poor response to first-line treatments contributes to prolonged disease activity and damage. To improve diagnosis and develop target-directed individualised treatments, identification of disease-specific pathomechanisms is essential. Because CD4+ T cells contribute to inflammation and damage in JIA, we investigated DNA methylation patterns, an epigenetic mark regulating gene expression, in CD4+ T cells to identify disease-associated molecular signatures that may be used as disease biomarkers.

Methods : Peripheral blood mononuclear cells were collected from 10 psJIA, 5 ERA-JIA patients, and 8 healthy controls (HC). Non-regulatory (CD25-) CD4+ T-cells were separated through FACS sorting, and T cell subset distribution was analysed between groups (FlowJo). DNA methylation profiling was performed using Illumina EPIC850K arrays, followed by bioinformatic analysis with R packages minfi and ChAMP.

Results : Overall proportions of CD4+ T and CD8+ cells were comparable between groups. When compared to HC, psJIA patients exhibited a larger proportion of circulating effector memory and reduced naïve CD4+ T cells. DNA methylation analysis revealed 1157 differentially methylated positions (DMPs) associated with 671 genes between psJIA and HC, 1484 DMPs affecting 922 genes between psJIA and ERA-JIA patients, and 1380 DMPs affecting 760 genes between ERA-JIA and HC. Principal component (PCA) and partial least-squares discriminant analysis (PLS-DA) of DNA separated psJIA from HC and ERA-JIA. Gene ontology (GO) analysis in psJIA comparing with HC involved in “Modulation by symbiont of entry into host” and “Regulation of Viral Life Cycle”. In both psJIA versus HC and psJIA versus ERA-JIA, GO analysis involved terms suggested overlapping pathways between JIA subforms, including “Synapse Assembly”, “Neurotransmitter Secretion” and “Neuron Cell-Cell Adhesion”. Several genes included ≥3 hypomethylated DMPs in their promoter region suggesting increased potential for gene expression. These included the interferon-alpha antiviral effector gene IQ motif-containing GTPase-activating protein 2 (IQGAP2), the neuronal pro-survival factor ninjurin 2 (NINJ2) that has previously been linked with Inflammatory Bowel Disease and Peripheral Autonomic Neuropathy, Fibroblast growth factor receptor 2 (FGFR2) that plays a role in bone growth during embryonic development, and Homeobox protein Hox-A5 (HOXA5) that is part of a developmental regulatory system providing cells with positional identities.

Conclusions : Differential DNA methylation signatures promise potential as future biomarkers to differentiate between psJIA and ERA-JIA. Permissive DNA methylation patterns affecting several genes may explain molecular and clinical features associated with psJIA.

IRB Statement : The study was approved by the South Central - Oxford A Research Ethics Committee. In accordance with the Declaration of Helsinki, written informed consent was obtained from all participants and/or their legal guardians.

Acknowledgments : The authors wish to acknowledge CARRA and the ongoing Arthritis Foundation financial support of CARRA.

## A36 Gaps in care in adolescents and young adults with childhood-onset systemic lupus

### Tamar Rubinstein^1^, Lisbel Guzman^2^, MaryAnn Ramos^3^, Carmen Rodriguez^2^, Syeda Samar Sohail^1^, Chaim Putterman^4^, Vilma Gabbay^5^

#### ^1^Albert Einstein College of Medicine, Bronx, NY; Children’s Hospital at Montefiore, Bronx, NY, USA; ^2^Albert Einstein College of Medicine, Bronx, NY; ^3^Children’s Hospital at Montefiore, Bronx, NY; ^4^Azrieli Faculty of Medicine, Bar-Ilan University, Zefat, Israel; Albert Einstein College of Medicine, Bronx, NY; ^5^University of Miami/ Nathan Kline Institute for Psychiatric Research

##### **Correspondence:** Tamar Rubinstein

*Pediatric Rheumatology 2024*, **22(S1):**A36

Background : Childhood-onset systemic lupus (cSLE) has especially high morbidity and mortality with suboptimal care; the adolescent and young adult (AYA) age is characterized by challenges in health behaviors. Our study aims to investigate psychosocial contributors to disengagement in care among AYA with cSLE from the Bronx, NY, a vulnerable population at high risk for poor outcomes. In preparation, we set out to describe the prevalence of gaps in lupus care (defined as no lupus visits for ≥6 months, as a primary example of disengagement) and related factors among the eligible cohort of patients. Our intention is to monitor the consented study sample for bias and consider targeted recruitment efforts to ensure that we do not unintentionally select out the most affected patients.

Methods : We extracted data from the electronic medical record (EMR) of all patients 15-24 years old who had a diagnostic codes related to lupus associated with a visit in a pediatric rheumatology clinic (treating through age 25) during a 12-month retrospective observation period (Jan 2021-Dec 2022). Through EMR chart review we excluded those without a diagnosis of SLE (by SLICC or EULAR/ACR Criteria) and those without diagnosis or documented symptoms ≤18 years old. Demographics, and screens for social needs and depression were collected from visit data as close to the index date as available. We used Mann Whitney U tests, Chi-square tests, and Fisher’s exact tests, to identify factors associated with gaps in care (≥180 days between lupus visits).

Results : Among 91 AYA with cSLE, 40 (44%) had gaps in care during the observation period. Associations between demographics/ patient factors and gaps in care are shown in Table 1. Among 65 patients with social needs screens completed, 15 (23%) had at least one unmet need. The most common of which were food insecurity and transportation needs endorsed each by 5 (8%). Having any unmet social needs was significantly associated with having a history of gaps in care (*p*=0.05), as was older age (*p*=0.01). Spanish speaking vs English speaking was a significant protective factor (*p*=0.04).

Conclusions : Gaps in care are common among AYA with cSLE from the Bronx, NY. To ensure health equity when developing interventions to address disengagement in care, it is imperative that the most affected patients are not left out of research. In our further work to investigate specific psychosocial factors influencing disengagement, we will periodically assess the generalizability of our consented study cohort. Our goal is to identify targets for intervention to improve engagement in care in AYA with cSLE in this high-risk population.

IRB Statement : This study was approved by the Einstein/Montefiore Institutional Review Board #14314.

Acknowledgements : Research reported in this publication was supported by the CARRA/Arthritis Foundation (AF) Career Development Award and the National Institute of Arthritis and Musculoskeletal and Skin Diseases and the Office of the Director of the National Institutes of Health under Award Number K23AR080803. The content is solely the responsibility of the authors and does not necessarily represent the official views of the National Institutes of Health or CARRA/Arthritis Foundation.

**Table 1 (Abstract A36):** Demographic and psychosocial factors associated with disengagement in care adolescents and young adults (AYA) from a clinical cohort in the Bronx, NY Disengaged in care was defined as ≥6 months without a lupus clinic visit in a 12-month observation period. *P* values represent Kruskal-Wallis tests for continuous variables; chi-square tests for (1) gender, (2) unmet social needs, and (3) Depression screening; Fischer exact tests for (1) race/ethnicity and (2) preferred language

	Total cohort	AYA with gaps in care*	AYA without gaps in care	*p* value
Age (median, IQR)	20 (18, 21)	21 (19, 22)	19 (17, 21)	0.01
Gender				0.1
Female (n, %)	77 (85%)	31 (78%)	46 (90%)	
Male (n, %)	14 (15%)	9 (23%)	5 (10%)	
Non-binary (n, %)	0	0	0	
Race/ ethnicity				0.8
Black	35 (38%)	16 (46%)	19 (37%)	
Hispanic/ Latino, non-Black	42 (46%)	17 (43%)	25 (49%)	
Other	13 (14%)	6 (15%)	7 (14%)	
Missing	1 (1%)	1 (3%)	0	
Median household income by zipcode (median, IQR)	48,000 (37,000-61,000)	56,000 (39,000-63,000)	39,000 (37,000-59,000)	0.1
Preferred language				0.01
English	79 (87%)	39 (97.5%)	40 (78%)	
Spanish	12 (13%)	1 (2.5%)	11 (22%)	
Unmet social needs				0.04
None	50 (55%)	16 (40%)	34 (68%)	
≥1	15 (16%)	9 (23%)	6 (12%)	
Missing	26 (29%)	15 (38%)	11 (22%)	
Depression screening (PHQ9)				0.4
Clinical symptoms	24 (26%)	13 (33%)	11 (22%)	
None	60 (67%)	25 (63%)	35 (69%)	
Missing	7 (8%)	2 (5%)	5 (10%)	

## A37 Belimumab use in childhood-onset lupus: outcomes from the childhood arthritis and rheumatology research alliance registry

### Jordan Roberts^1^, Kristen Carlin^2^, Kristen Hayward^1^, Rebecca Sadun^3^, Emily Smitherman^4^, Scott Wenderfer^5^, for the CARRA Registry Investigators^6^

#### ^1^Seattle Children’s Hospital, University of Washington School of Medicine, Seattle, WA, USA; ^2^Seattle Children’s Hospital; ^3^Duke University School of Medicine, Durham, NC, USA; ^4^University of Alabama; ^5^BC Children’s Hospital; ^6^Childhood Arthritis and Rheumatology Research Alliance (CARRA)

##### **Correspondence:** Jordan Roberts

*Pediatric Rheumatology 2024*, **22(S1):**A37

Background : Belimumab is a biologic therapy targeting B-cell activating factor (BAFF) which was recently approved for childhood-onset systemic lupus erythematosus (cSLE). However, use in the pediatric population remains less common than among adults with SLE, and data on belimumab effectiveness in cSLE outside of the clinical trial setting is limited. We aimed to describe characteristics of patients enrolled in the Childhood Arthritis and Rheumatology Research Alliance (CARRA) Registry who received belimumab for cSLE, and to assess changes in oral glucocorticoid doses, disease activity scores and patient-reported outcomes in the year following belimumab initiation.

Methods : We included youth with cSLE enrolled in the CARRA Registry. We compared demographic and disease characteristics of those who were exposed to ≥ 1 dose of belimumab with all cSLE patients enrolled in the CARRA Registry. Summary statistics were performed to describe medications: 1) co-administered within 30 days of belimumab initiation, and 2) used at any time prior to belimumab administration. Among Registry participants who received belimumab, linear mixed effects models were used to assess trajectories of SLEDAI-2K scores, prednisone-equivalent daily oral glucocorticoid doses, and PROMIS (Patient-Reported Outcomes Measurement Information System) measures, including pain, global health and mobility scores, over the 12 months following belimumab initiation. This study was reviewed and approved by the Seattle Children’s Hospital IRB, Protocol #00003967.

Results : Out of 994 patients with a baseline visit for cSLE, we identified 63 patients who received ≥ 1 dose of belimumab. Patient and disease characteristics of belimumab users and the entire CARRA Registry pSLE cohort are presented in Table 1. The mean SLEDAI-2K score at time of belimumab initiation was 9.4. Forty-four percent of belimumab users had a history of lupus nephritis. At time of belimumab initiation, 83% of patients were prescribed an antimalarial, 70% were on oral glucocorticoids, and 59% were receiving mycophenolate mofetil concurrently (Table 2). Twenty-four percent had previously received rituximab, and 16% had a history of cyclophosphamide exposure prior to first belimumab dose. The mean prednisone dose at time of belimumab initiation was 13 mg/day. SLEDAI-2K scores and daily oral prednisone dose decreased significantly over the 12 months following belimumab initiation (Table 3). PROMIS global health and mobility scores significantly improved. PROMIS pain scores showed no statistically significant change.

Conclusions : Youth with cSLE in the CARRA Registry who were prescribed belimumab were similar to the entire CARRA cSLE cohort. Most recipients had longstanding disease at time of belimumab initiation. Among this multicenter cohort of cSLE patients, disease activity scores and daily oral prednisone equivalent doses significantly decreased during the first year after starting belimumab. Patient-reported mobility and global health scores improved. Further research is needed to determine whether changes after starting belimumab represent improved outcomes compared to alternative therapeutic strategies.

IRB Statement : This study was reviewed and approved by the Seattle Children’s Hospital IRB, Protocol #00003967.

Acknowledgements : We would like to acknowledge the support of CARRA via a Data Analysis Support Grant, and the contributions of the CARRA Lupus Nephritis Workgroup members. This work could not have been accomplished without the aid of the following organizations: The NIH’s National Institute of Arthritis and Musculoskeletal and Skin Diseases (NIAMS) & the Arthritis Foundation. We would also like to thank all participants and hospital sites that recruited patients for the CARRA Registry. The authors thank the following CARRA Registry site principal investigators: K. Abulaban, C. Aguiar Lapsia, S. Ardoin, L. Barillas-Arias, M. Basiaga, K. Baszis, H. Brunner, H. Bukulmez, E. Chalom, J. Chang, D. Co, K. Cook, A. Cooper, C. Correll, T. Davis, F. Dedeoglu, M. DeGuzman, A. Dhanrajani, K. Ede, B. Edelheit, B. Feldman, I. Ferguson, D. Glaser, D. Goldsmith, B. Gottlieb, T. Graham, T. Griffin, T. Hahn, L. Harel, O. Harry, M. Hollander, S. Hong, M. Horwitz, J. Hsu, A. Huber, L. Imundo, C. Inman, P. Kahn, S. Kim, D. Kingsbury, M. Klein-Gitelman, L. Lim, M. Mannion, D. McCurdy, D. Milojevic, S. Mohan, T. Moore, K. Moore, L. Moorthy, S. Nativ, M. Natter, K. Onel, J. Patel, S. Prahalad, C. Rabinovich, A. Robinson, T. Ronis, M. Rosenkranz, N. Ruth, S. Sabbagh, K. Schikler, C. Schutt, E. Sloan, J. Spitznagle, Y. Sterba Rakovchik, K. Stewart, G. Syverson, S. Tarvin, M. Tesher, D. Toib, M. Toth, M. Twilt, H. Van Mater, D. Wahezi, P. Weiss, J. Weiss, L. Woolnough, E. Wu, A. Yalcindag, Y. Zhao

**Table 1 (Abstract A37):** CARRA pSLE cohort and belimumab user patient characteristics

	Belimumab Users at Time of Belimumab Initiation (***n***=63)	Cohort with pSLE Diagnosis at Baseline Visit (***n***=994)
Female	58 (92.1%)	856 (86.1%)
Age at SLE Diagnosis, Years, Mean (Standard Deviation)	13.5 (3.3)	13.9 (2.9)
Age at Belimumab Initiation, Years, Mean (SD)	15.9 (2.9)	---
Disease Duration at Belimumab Initiation, Months, Median (IQR)	18 (30.0)	---
SLEDAI-2K Score, Mean (SD)	9.4 (6.6)	8.6 (7.6)
Race		
White	15 (30.6%)	260 (34.8%)
Black	25 (51.0%)	283 (37.8%)
Asian	5 (10.2%)	112 (16.3%)
More than 1 Race	3 (6.1%)	43 (5.7%)
Other	1 (2.0%)	40 (5.3%)
Hispanic Ethnicity	19 (30.2%)	267 (26.9%)
Insurance		
Public	33 (52.4%)	412 (41.5%)
Private	21 (33.3%)	427 (43.0%)
Uninsured	0 (0.0%)	21 (2.1%)
Military	4 (6.3%)	22 (2.2%)
Non-US	2 (3.2%)	55 (5.5%)
Other	3 (4.8%)	42 (4.2%)
More than 1 Insurance Type	0 (0.0%)	13 (1.3%)
Household Income		
<$25,000	5 (12.2%)	124 (19.3%)
$25-49,999	14 (34.1%)	163 (25.4%)
$50-74,999	13 (31.7%)	98 (15.3%)
$75-99,999	4 (9.8%)	77 (12.0%)
$100-150,000	3 (7.3%)	91 (14.2%)
Above $150,000	2 (4.9%)	89 (13.9%)
Parental Education		
Less than high school	6 (12.5%)	98 (12.0%)
High school graduate	15 (31.3%)	203 (24.8%)
College	23 (47.9%)	358 (43.7%)
Graduate School	4 (8.3%)	161 (19.6%)
History of Lupus Nephritis	28 (44.4%)	419 (43.8%)

**Table 2 (Abstract A37):** Co-administered and previously used medications for pSLE among belimumab initiators

Medication	Any Prior Use n (%)	Use at Time of Belimumab Initiation n (%)
Hydroxychloroquine/chloroquine	54 (85.7)	52 (82.5)
Oral glucocorticoids	54 (85.7)	44 (69.8)
Mycophenolate mofetil	43 (68.3)	37 (58.7)
Intermittent pulse dose IV glucocorticoids	30 (47.6)	16 (25.4)
Methotrexate	18 (28.6)	8 (12.7)
Azathioprine	15 (23.8)	4 (6.3)
Rituximab	15 (23.8)	5 (7.9)
Cyclophosphamide	10 (15.9)	3 (4.8)
Tacrolimus	4 (6.3)	4 (6.3)
TNFi	2 (3.2)	0 (0.0)
Anakinra	1 (1.6)	0 (0.0)

**Table 3 (Abstract A37):** Changes in disease activity, prednisone dose and patient reported outcomes during first year following belimumab initiation

	SLEDAI-2K, β (95% CI)	Daily Oral Prednisone Equivalent Dose, β (95% CI)	PROMIS Pain, β (95% CI)	PROMIS Global Health, β (95% CI)	PROMIS Mobility, β (95% CI)
**Intercept** (estimated mean score or dose, at time of belimumab initiation)	9.5 (8.1, 10.9)	13.2 (10.0, 16.4)	53.9 (50.9, 56.8)	36.9 (35.1, 38.7)	46.2 (43.5, 49.0)
**Time** (estimated change per month)	-0.29 (-0.46, -0.13)^*^	-0.49 (-0.87, -0.10)^*^	-0.00 (-0.01, 0.01)	0.01 (0.00, 0.02)^*^	0.01 (0.00, 0.02)^*^

^*^ *p*<0.05

## A38 Peripheral blood immunophenotype of juvenile scleromyositis overlap patients using bulk RNA sequencing: are overlap patients molecularly distinct?

### Amanda Robinson^1^, Gabrielle Morgan^2^, Giffin Werner^3^, Anwesha Sanyal^3^, Haley Havrilla^3^, Srilakshmi Chaparala^4^, Lauren Pachman^5^, Kathryn Torok^3^

#### ^1^University of Pittsburgh Medical Center, Pittsburgh, PA, USA; University of Utah, Salt Lake City, UT, USA; ^2^Ann & Robert H. Lurie Children’s Hospital of Chicago, Chicago, IL, USA; ^3^University of Pittsburgh Medical Center, Children’s Hospital of Pittsburgh, Pittsburgh, PA, USA; ^4^Molecular Biology Information Service, Health Science Library System, University of Pittsburgh, Pittsburgh, PA, USA; ^5^Northwestern University Feinberg School of Medicine, Ann & Robert H. Lurie Children’s Hospital of Chicago, Chicago, IL, USA

##### **Correspondence:** Amanda Robinson

*Pediatric Rheumatology 2024*, **22(S1):**A38

Background : Juvenile systemic sclerosis (jSSc) and juvenile dermatomyositis (JDM) are rare systemic autoimmune diseases that share a similar age of onset, heavy cutaneous disease burden, and frequent internal organ involvement. A proportion of jSSc and JDM patients have features of both diseases, scleromyositis overlap disease (jOverlap). There are no universally accepted classification criteria for this subset of patients. Previous analysis of a single center cohort of jSSc demonstrated clinical differences between patients with and without overlap features. jOverlap was common, associated with more frequent musculoskeletal involvement including myositis, and was enriched for U1RNP and PM-Scl autoantibody positivity. This study utilizes bulk RNA sequencing (RNA-Seq) with differentially expressed gene (DEG) analyses to evaluate the peripheral blood immunophenotype of jOverlap patients compared to healthy controls, jSSc, and JDM.

Methods : Peripheral blood bulk RNA-Seq was performed on children with jSSc (*n*=25), JDM (*n*=25), jOverlap (*n*=26), and age/sex matched healthy controls (HCs) (*n*=21). Samples were collected at two tertiary care referral centers with dedicated jSSc and JDM clinics, each contributing jOverlap samples. Disease category was determined by the treating physician. Demographics, clinical disease manifestations, treatment status, and autoantibody profile were extracted. RNA was isolated from peripheral blood and sequenced using the Illumina HTS TruSeq RNA Access library preparation kit and Illumina NextSeq 500 platform. Data processing and analyses were performed using Partek Flow software and the DESeq2 platform. Significant DEGs were identified based on a log2 fold cutoff value of ≤1.5 or ≥1.5 and a false discovery rate (FDR) step up of < 0.1. Following principal component analysis on normalized data, t-distributed stochastic neighbor embedding (t-SNE) was performed to determine clusters based on gene expression similarity.

Results : jOverlap patients were predominantly female and White. jOverlap exhibited a unique autoantibody distribution, enriched for PM-Scl, U1RNP, and U3RNP autoantibodies (Table 1). Comparison of jOverlap to HCs demonstrated 295 DEGs, 209 of which were up-regulated. The top protein coding genes of significance are summarized in Table 2 and include those involved with multiple immune functions including type 1 INF signaling, an immune signature well documented in both adult and juvenile dermatomyositis. Down-regulated genes included those associated with the immunoglobulin chain. Clustering analyses using t-SNE are shown in Figure 1. jOverlap was found in all 4 unique clusters.

Conclusions : Scleromyositis overlap patients demonstrate a unique immunophenotype compared to HCs, jSSc, and JDM. Though HCs, jSSc, and JDM clustered well together, jOverlap was found in all clusters identified, suggesting disease category is not the only modifier responsible for differential gene expression. Additional analyses of the relationship between DEGs with age, autoantibody positivity, clinical disease manifestations, and disease activity are needed to further characterize this unique patient population.

IRB Statement : This study was approved by the University of Pittsburgh IRB (PRO11060222).

Acknowledgements : The authors wish to acknowledge CARRA and the ongoing Arthritis Foundation financial support of CARRA. This work was generously funded by a CARRA-Arthritis Foundation Small Grant.

**Table 1 (Abstract A38):** Demographic, treatment status, and autoantibody profile for patients with juvenile systemic sclerosis, juvenile dermatomyositis, and juvenile scleromyositis overlap disease

	Juvenile Systemic Sclerosis (*n*=25)	Juvenile Dermatomyositis (*n*=25)	Juvenile Scleromyositis Overlap Disease (*n*=26)
Demographics			
Age at Disease Onset (y) (mean (IQR))	9.3 (5.9-12.5)	6.4 (3.2-8.8)	10.0 (8.0-12.5)
Age at Diagnosis (y) (mean (IQR))	11.7 (8.3-15.3)	7.1 (4.0-10.5)	11.1 (8.7-14.3)
Age at Sample (y) (mean (IQR))	13.2 (10.3-16.8)	9.5 (6.0-13.9)	14.5 (12.7-17.1)
Female (%)	92	68	65
Race (%)			
White	84	100	65
Black	16	0	15
Asian	0	0	12
Other/Mixed	0	0	8
Hispanic (%)	12	28	23
Treatment Status			
Duration of Untreated Disease (m) (mean (IQR))	28.5 (11.6-36.2)	8.4 (2.4-11.0)	14.7 (2.8-15.4)
Duration Sample Treatment (m) (mean (IQR))	16.6 (2.9-25.5)	27.5 (3.0-31.2)	38.8 (7.8-56.2)
Primary Autoantibody (%)			
ANA only	24	0	0
Scl-70	52	0	3.8
Centromere	12	0	0
RuvBL1/2	4	0	0
p155/140	0	40	11.5
Mi-2	0	12	0
MDA5	0	8	3.8
Jo-1	0	4	0
MJ	0	4	7.7
Th/To	0	4	0
PM-Scl	0	4	23.1
U1RNP	4	4	23.1
U3RNP	0	0	15.4
Multiple	0	4	3.8
Negative	0	16	3.8
Unknown	4	0	3.8

**Table 2 (Abstract A38):** Top up-regulated and down-regulated DEGs and associated pathways observed in children with scleromyositis overlap disease compared to healthy controls

DEG	Pathway	p	FDR step up	Fold change
Up-Regulated				
IFI27	type 1 INF signaling, apoptosis, innate immune response	5.21E-16	9.90E-12	49.4
SIGLEC1	cell-cell adhesion	1.01E-07	4.82E-04	5.95
IFI44L	type 1 INF signaling	2.46E-07	7.80E-04	5.50
GPRC5B	NFkB signaling, macrophage cytokine production	4.67E-08	2.96E-04	4.47
NOTCH2NLR	calcium ion binding	3.63E-05	8.43E-03	4.23
USP18	negative regulation of type I INF signaling, innate immune response	1.20E-06	1.52E-03	3.91
RSAD2	CD4+ alpha-beta T cell differentiation and activation, defense response to virus, innate immune response, TH2 cell cytokine production, TLR 7/9 signaling	1.46E-04	1.98E-02	3.52
CXCL10	cellular response to IL-17, regulation of T cell chemotaxis, endothelial cell activation, negative regulation of angiogenesis, negative regulation of myoblast differentiation	7.39E-05	1.33E-02	3.31
IFITM3	type I INF-mediated signaling pathway, response to type II INF	8.26E-06	3.57E-03	3.27
Down-Regulated				
IGHV1-69D	B cell receptor signaling, complement activation	4.57E-06	2.59E-03	-5.36
IGHA2	B cell receptor signaling, complement activation	1.83E-04	2.20E-02	-4.04
KLRC2	positive regulation of NK cell mediated cytotoxicity	2.21E-05	6.46E-03	-2.93
EGR3	angiogenesis, endothelial cell chemotaxis, muscle organ development, gamma-delta T cell differentiation	4.21E-04	3.66E-02	-2.49
FOXH1	cellular response to cytokine stimulus	1.26E-03	6.51E-02	-2.28

**Fig. 1 (Abstract A38):**
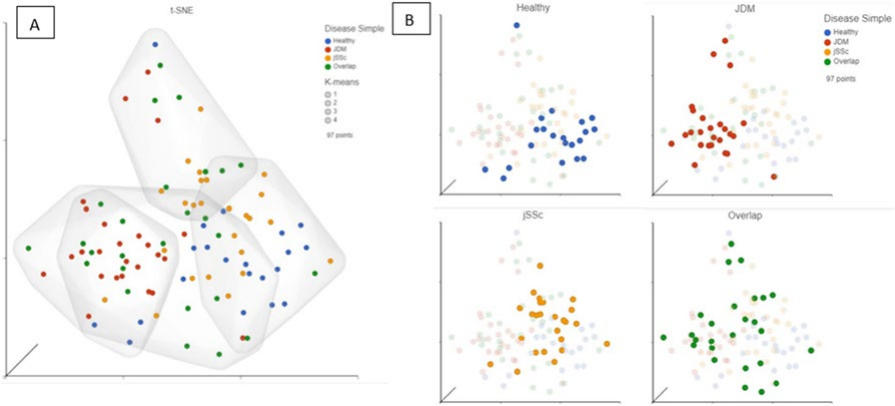
t-SNE plot of bulk RNA-seq data for jOverlap, jSSc, JDM, and HC (**A**), split by disease category (**B**)

## A39 A mental health workshop for pediatric rheumatology providers

### Aviya Lanis^1^, Kaveh Ardalan^2^, Suzanne Edison^3^, Alana Goldstein-Leever^4^, Andrea Knight^5^, Sarah Mohammed^6^, Tamar Rubinstein^7^, Rebecca Sadun^2^, For the CARRA Mental Health Workgroup^8^

#### ^1^Seattle Children’s Hospital, Seattle, WA, USA; ^2^Duke University School of Medicine, Durham, NC, USA; ^3^Cure JM; ^4^Nationwide Children’s Hospital, Columbus, OH, USA; ^5^The Hospital for Sick Children and SickKids Research Institute, Toronto, Canada; ^6^American College of Rheumatology; ^7^Albert Einstein College of Medicine, Bronx, NY; Children’s Hospital at Montefiore, Bronx, NY, USA; ^8^ Childhood Arthritis and Rheumatology Research Alliance (CARRA)

##### **Correspondence:** Aviya Lanis

*Pediatric Rheumatology 2024*, **22(S1):**A39

Background : Patients with rheumatic diseases have a high mental health burden with estimates of anxiety and depression rates as high as 65% [1]. Patients and caregivers have identified pediatric rheumatologists as the preferred source of mental health care referral and resources, but pediatric rheumatology centers have limited resources for identifying or treating patients’ mental health challenges [2, 3]. In an effort to address the gap in support and empower pediatric rheumatologists to facilitate mental health conversations, we developed a workshop for pediatric rheumatologists on integrating mental health screening and discussions into rheumatology clinic encounters. The current study aimed to assess the impact of this workshop on the provider confidence and the likelihood that pediatric rheumatology providers would start screening for and addressing mental health concerns in clinic.

Methods : A two-hour in-person workshop was held at the 2023 American College of Rheumatology Convergence. The workshop included a review of the importance of addressing mental health, how to start conversations around mental health, screening tools, assessing suicidality, and overcoming common barriers for screening. Participants consolidated their skills by engaging in a 30-minute break-out activity during the workshop, inclusive of two role-play scenarios. Participant confidence in addressing mental health care was assessed using a retrospective pre/post survey with a 5-point Likert scale (very low to very high confidence) for eight domains. Descriptive statistics and a paired two-tailed t-tests were used in data analysis.

Results : Sixty-six participants attended some or all of the workshop, with a survey response rate of 18%. Survey respondents included two pediatric rheumatology fellows in their first and second years of fellowship and 10 pediatric rheumatology attendings with a median of 13.5 years (range 0.2-47) in their attending roles. Table 1 demonstrates that a statistically significant increase in confidence took place for 6 of the 8 domains assessed. Ten of the twelve survey respondents reported that they were somewhat or extremely likely to share workshop resources with patients. Most respondents reported infrequent use of mental health screening measures (Figure 1), but after workshop completion most respondents reported being somewhat or very likely to use mental health screeners in the future (Figure 2).

Conclusions : In-person workshops can improve pediatric rheumatologist confidence in assessing and addressing the mental and emotional health needs of their patients. Based on participant feedback, additional trainings are needed to educate pediatric rheumatologists in pharmacologic and non-pharmacologic strategies to manage childhood mental health problems.

IRB Statement : This study was granted an exemption by the Duke University Institutional Review Board.

Acknowledgements : The Childhood Arthritis and Rheumatology Research Alliance’s support for the Mental Health Workgroup and the American College of Rheumatology (ACR) Annual Meeting Planning Committee (AMPC) support group were essential for completion of this project. Thank you to Dr. Susan Shenoi for her additional support as the ACR AMPC Pediatric representative who helped make this workshop a reality. The authors wish to acknowledge CARRA and the ongoing Arthritis Foundation financial support of CARRA.

References

1. Goldstein-Leever A et al. Increasing access to psychological services within pediatric rheumatology care. Ped Rheumatol 2023;21:51.
2. Knight AM et al. Barriers and facilitators for mental healthcare in pediatric lupus and mixed connective tissue disease: A qualitative study of youth and parent perspectives. Ped Rheum Online J 2015;13:52.
3. Fawole OA et al. Engaging patients and parents to improve mental health intervention for youth with rheumatological disease. Ped Rheum Online J 2021;19:19.

**Table 1 (Abstract A39):** Confidence level of participants in assessing and addressing mental healthcare-related domains before and after workshop participation. Confidence level measured on a 5-point Likert scale using a retrospective pre/post survey

Confidence level Domain	Pre-Workshop Mean	Post-Workshop Mean	***P***-Value
Initiate discussions with patients about emotional health	3.58	4.33	0.0015
Initiate discussions with caregivers about emotional health	3.58	4.25	0.0007
Assess patients with chronic medical conditions for comorbid depression	3.58	4.00	0.0538
Assess patients with chronic medical conditions for comorbid anxiety	3.50	4.08	0.0116
Assess patients with chronic medical conditions for suicidal ideation risk	3.25	3.92	0.1201
Use the Patient Health Questionnaire (PHQ)-8 or PHQ-9	3.50	4.00	0.0261
Use the Generalized Anxiety Disorder-7	3.25	3.83	0.0116
Use the Ask Suicide-Screening Questions (ASQ) or Columbia Suicide-Severity Rating Scale (C-SSRS)	2.58	3.42	0.002

**Fig. 1 (Abstract A39):**
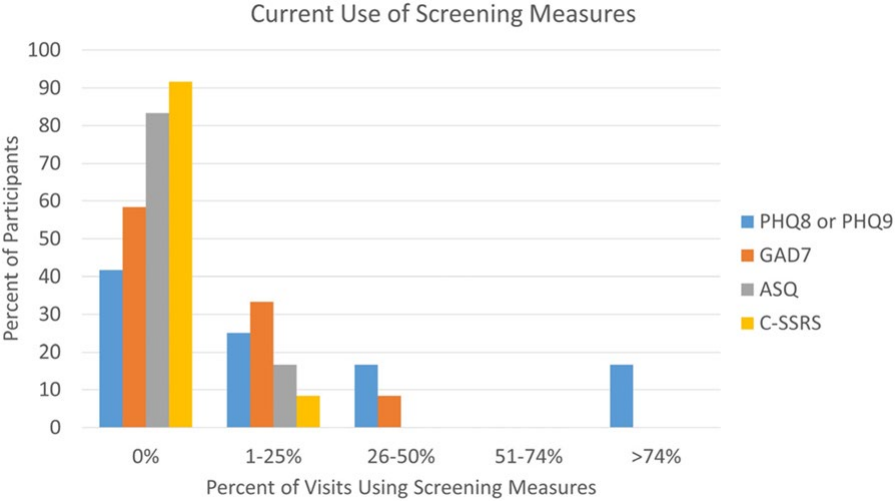
Reported frequency of screening measure use prior to participation in the workshop. PHQ=Patient Health Questionnaire; GAD7=Generalized Anxiety Disorder-7; ASQ=Ask Suicide-Screening Questions; C-SSRS=Columbia Suicide-Severity Rating Scale

**Fig. 2 (Abstract A39):**
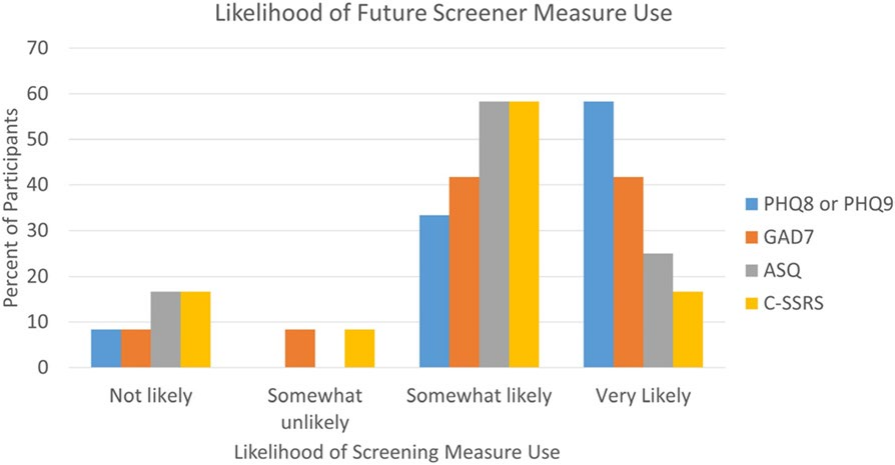
Report of likelihood to use screening measures following participation in the workshop. PHQ=Patient Health Questionnaire; GAD7=Generalized Anxiety Disorder-7; ASQ=Ask Suicide-Screening Questions; C-SSRS=Columbia Suicide-Severity Rating Scale

## A40 Improving the utilization and completeness of the pediatric rheumatology provider handoff for patients diagnosed with juvenile idiopathic arthritis

### Sarah Baluta, Christina Schutt, Anne LePard, Amy Vallee, Chrisana Pokorny, Homaira Rahimi, Bethany Marston, Barbra Murante

#### University of Rochester Medical Center

##### **Correspondence:** Sarah Baluta

*Pediatric Rheumatology 2024*, **22(S1):**A40

Background : A provider handoff is a means for transferring information between healthcare providers. Inadequate or inaccurate handoff communication among providers pose a safety risk to patients and can lead to adverse outcomes1-2. Studies that have examined the effectiveness of various handoff strategies show that the most effective intervention involves the use of a standardized handoff protocol3. The aim of this study was to increase the utilization of a standardized provider handoff for patients diagnosed with Juvenile Idiopathic Arthritis (JIA) among healthcare providers within the Pediatric Rheumatology Division at the University of Rochester Medical Center from 29% to 90% over a four-month period.

Methods : Data was collected through chart review of the electronic medical record (EMR) of patients diagnosed with juvenile idiopathic arthritis subtypes. Healthcare providers within the division of pediatric rheumatology were provided with an educational intervention about how to properly fill out the standardized provider handoff, and how to transfer pertinent patient information from within the EMR to the provider handoff (Figure 1). The study team record whether a provider had ever filled out the standardized provider handoff, and whether the information entered for the characteristic section into the handoff was accurate and complete on a monthly basis from August to November 2023. Patient data was de-identified and stored within a secured document that is password protected with only access to the healthcare providers within the pediatric division at the University of Rochester Medical Center.

Results : A baseline review of the utilization of the provider handoff showed that 89% of providers utilized the handoff in May 2023, but only 29% of the utilized handoffs had a completed and accurate characteristic section (disease subtype, clinical features, and relevant antibodies). After an educational intervention in July 2023 and changes to the standardized provider handoff in September 2023, there were improvements in provider utilization and completeness of the characteristic section within the provider handoff to 94.7% and 71.9% respectively (Figures 2 and 3).

Conclusions : Provider handoffs can improve coordination and continuity of care among different providers within an organization or practice2. Using a standardized provider handoff can improve utilization, accuracy, and completeness of provider handoffs for patients with JIA.

IRB Statement : The University of Rochester Policy 1001 under Quality Improvement; “Activities generally NOT human subject research does not require RSRB review. Quality Assuarance and Quality Improvement (QA/QI) Pokections do not require RSRB Review.”

References

1. Manser T, Foster S, Gisin S, Jaeckel D, Ummenhofer W. Assessing the quality of patient handoffs at care transitions. Qual Saf Health Care. 2010 Dec;19(6):e44. doi: 10.1136/qshc.2009.038430. PMID: 21127094
2. Jewell, Jennifer A. et al. “Standardization of Inpatient Handoff Communication.” Pediatrics (Evanston) 138.5 (2016): e1–. Web
3. Raeisi A, Rarani MA, Soltani F. Challenges of patient handover process in healthcare services: A systematic review. J Educ Health Promot. 2019 Sep 30;8:173. doi: 10.4103/jehp.jehp_460_18. PMID: 31867358; PMCID: PMC6796291.

**Fig. 1 (Abstract A40):**
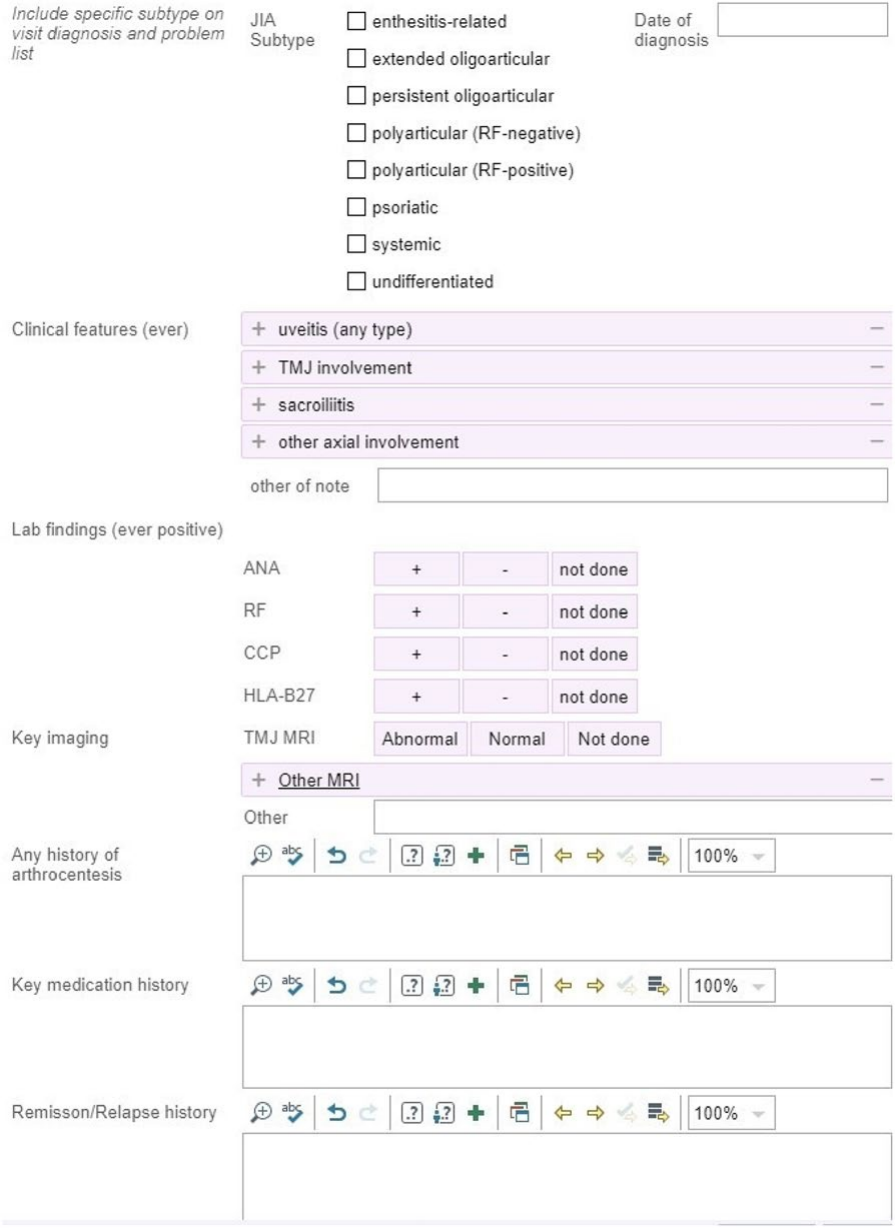
An example of the standardized provider handoff for a patient with JIA

**Fig. 2 (Abstract A40):**
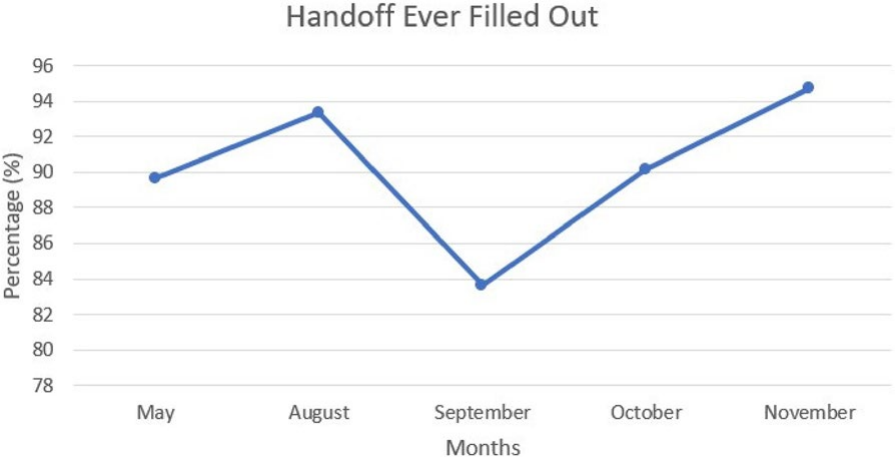
Percentage of provider handoff utilization following an educational intervention in July 2023 and improvements to the provider handoff in September 2023

**Fig. 3 (Abstract A40):**
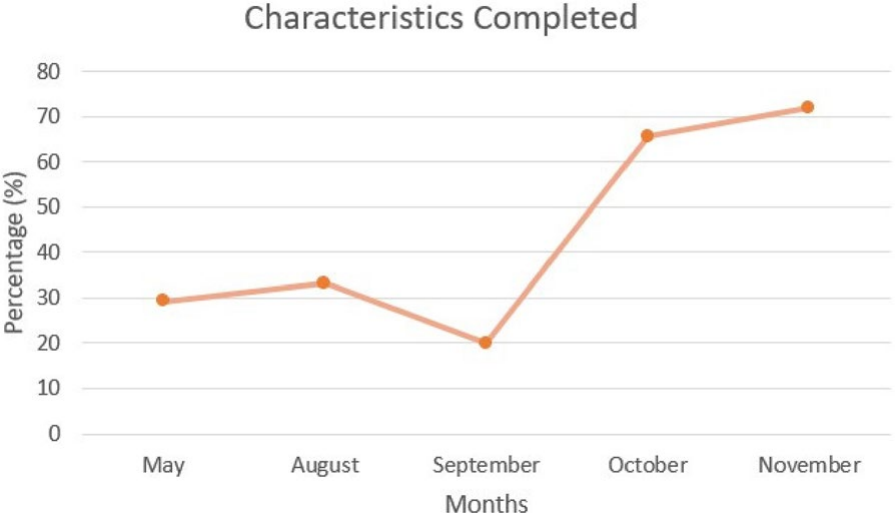
Percentage of provider handoff completeness of the characteristic section of the provider handoff following an educational intervention in July 2023 and improvements to the provider handoff in September 2023

## A41 IL10 inhibits toll-like receptor-9-induced T cell receptor-mediated T cell activation in macrophage activation syndrome

### Matthew Eremita^1^, Omar Geier^2^, Matthew Taylor^1^, Joyce Hui-Yuen^1^

#### ^1^Cohen Children’s Medical Center, Queens, NY, USA; ^2^Feinstein Institutes for Medical Research

##### **Correspondence:** Matthew Eremita

*Pediatric Rheumatology 2024*, **22(S1):**A41

Background : Macrophage activation syndrome (MAS) is a potentially fatal complication of systemic juvenile idiopathic arthritis (sJIA). Gene expression pathway analyses have identified altered Toll-like receptor (TLR) signaling in sJIA and MAS. Repeated administration of CpG, a TLR-9 agonist, induces an MAS-like phenotype in mice; fulminant disease is induced with co-administration of intereukin-10 receptor blocking antibody (αIL10R). IL10 is an anti-inflammatory cytokine that induces T cell immune tolerance. Deficient production of IL10 by antigen presenting cells drives pathology in sJIA patients and animal models of the disease. Polymorphisms in the IL10 gene family confer susceptibility to sJIA. We hypothesized that IL10 inhibits T cell activation (TCA) in TLR-9-induced inflammation, and that TCA contributes to hypercytokinemia in TLR-9-induced inflammation.

Methods : Five experimental groups of C57Bl6 mice were studied: 1) untreated, negative controls; 2) MAS: treated with CpG 50 μg intraperitoneally (ip) every other day for 5 doses days -8 – 0; 3) CpGHi: treated with CpG 500 μg ip on day 0; 4) CpGHi+αIL10R: treated with CpG 500 μg plus αIL10R 1000 μg (both ip) on day 0; 5) αCD3ε (positive controls): treated intravenously with 50 μg of anti-CD3ε stimulating antibody on day 0. On day 1 following completion of treatment animals were sacrificed. Spleens were harvested and digested, and leukocytes were isolated. Cells were cultured in brefeldin A to entrap expressed products intracellularly or were stained directly post-isolation. Stained cells were analyzed with flow cytometry. Non-specific TCA was quantified by CD69 staining on flow cytometry. T cell receptor-mediated- (TCR-) TCA was quantified by Nur77 staining on flow cytometry. Data were analyzed using one-way Analysis of Variance with Šídák post-hoc tests.

Results : MAS exposure did not affect non-specific TCA. CpGHi-exposure increased non-specific TCA in CD4+ T cells compared to MAS-exposed mice and untreated controls. CpGHi+αIL10R exposure increased non-specific TCA compared to MAS- and CpGHi-exposed mice, and untreated controls (Fig. 1, *p* < 0.05). MAS exposure did not affect TCR-TCA. CpGHi+αIL10R exposure increased TCR-TCA compared to MAS- and CpGHi-exposed mice, and untreated controls (Fig. 2, *p* < 0.05). CpGHi+αIL10R exposure increased the proportion of CD8+ T cells expressing TNFα compared to untreated controls and CpGHi-exposed mice (Fig. 3B, *p* < 0.05), but not MAS-exposed mice. CpGHi+αIL10R exposure increased serum concentrations of TNFα, IL6, and IL12 compared to MAS- and CpGHi-exposed mice, and untreated controls (Fig. 3C-E, *p* < 0.05). CpGHi+αIL10R exposure increased serum concentrations of IFNγ, CXC-motif chemokine ligand 9 (CXCL9), and CXCL10 compared to MAS-exposed mice and untreated controls (Fig. 3F-H, *p* < 0.05).

Conclusions : IL10 prevents TLR-9-induced TCA. TCA contributes to TLR-9-induced hypercytokinemia. The established, non-infectious model of MAS with repeated administration of low-dose CpG does not adequately capture the contribution of T cells to the development of fulminant disease.

IRB Statement : IACUC protocol 2022-021 is approved effective 1/4/23. Protocols are approved for a maximum 3-year period.

Acknowledgements : This study was funded by the 2023 CARRA-Arthritis Foundation Fellow Grant. The authors wish to acknowledge CARRA and the ongoing Arthritis Foundation financial support of CARRA.

**Fig. 1 (Abstract A41):**
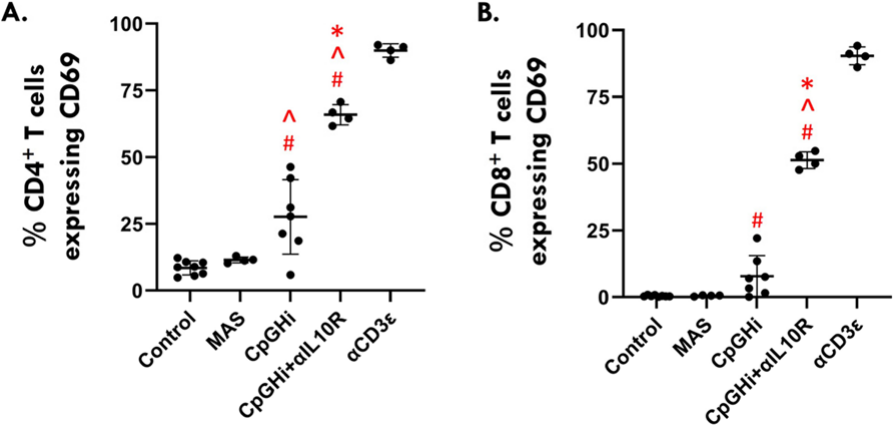
IL10 inhibits TLR9-induced non-specific T cell activation. Flow cytometric data showing the relative proportions of CD69+ splenic (**A**) CD4+ and (**B**) CD8+ T cells. Data are pooled from three experiments of 12, 7 and 8 10-12-week-old mice. Gates defining T cell populations were unique to individual experiments. αCD3ε-exposed mice served as positive controls for individual experiments (%CD69+ CD4+ and CD8+ T cells set at 90%). Data were analyzed using one-way Analysis of Variance with Šídák post-hoc tests. # vs Control; ^ vs MAS; * vs CpGHi; *p* < 0.05

**Fig. 2 (Abstract A41):**
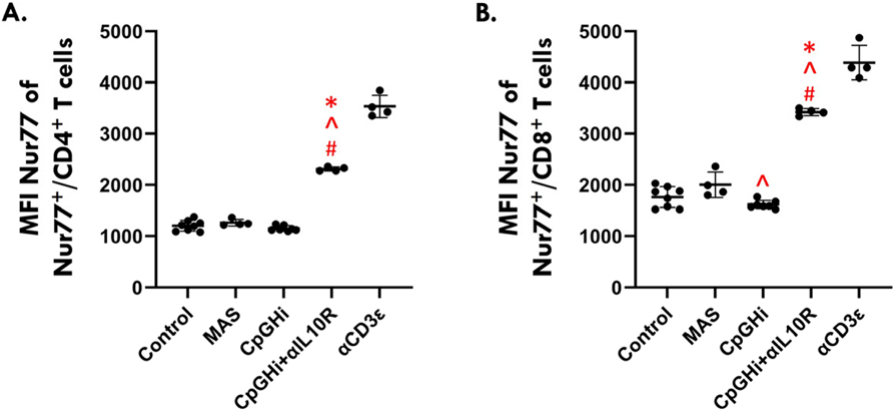
IL10 inhibits TLR9-induced T cell receptor-mediated T cell activation. Flow cytometric data showing the median fluorescence intensities (MFIs) of Nur77+ splenic (**A**) CD4+ and (**B**) CD8+ T cells. Data are pooled from three experiments of 12, 7 and 8 10-12-week-old mice. Gates defining T cell populations were unique to individual experiments. αCD3ε-exposed mice served as positive controls for individual experiments (%Nur77+ CD4+ and CD8+ T cells set at 90%). Data were analyzed using one-way Analysis of Variance with Šídák post-hoc tests. # vs Control; ^ vs MAS; * vs CpGHi; *p* < 0.05

**Fig. 3 (Abstract A41):**
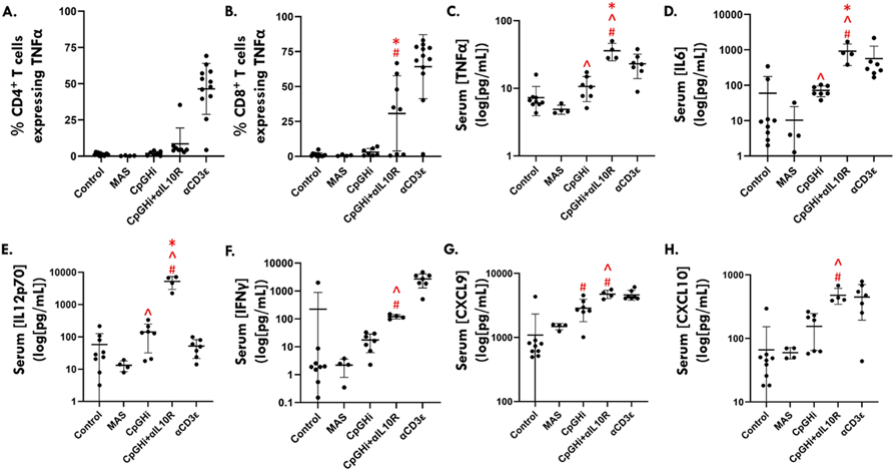
**A**-**H** TLR9-induced T cell receptor-mediated T cell activation correlates with severe hypercytokinemia. Flow cytometric data showing the relative proportions of TNFα+ splenic (**A**) CD4+ and (**B**) CD8+ T cells. Data are pooled from four experiments of 12, 11, 8 and 12 10-12-week-old mice. **C**-**H** Serum cytokine levels obtained via submandibular cheek bleed. Data are pooled from three experiments of 12, 11, and 8 10-12-week-old mice. Statistical analyses were performed following log transformation of the data owing to failure of tests for normality. Data were analyzed using one-way Analysis of Variance with Šídák post-hoc tests. # vs Control; ^ vs MAS; * vs CpGHi; *p* < 0.05

## A42 Deriving research priorities in neuropsychiatric systemic lupus erythematosus in children

### Ekemini Ogbu^1^, Martha Rodriguez^2^, Alexandra Theisen^3^, Simone Appenzeller^4^, Emily Beil^5^, Onengiya Harry^6^, Ryan Kammeyer^7^, Natoshia Cunningham^8^, Kristen Fisher^5^, Marisa Klein-Gitelman^9^, Eyal Muscal^5^, Nadine Schwartz^10^, Sefi Kronenberg^11^, Paige Seegan^12^, Lawrence Ng^13^, Marietta DeGuzman^5^, Monica Banks^14^, Angela Killinger^14^, Mckenna Bowes^15^, Ellie Rose Killinger^14^, Sarah Wong^14^, Kiana Johnson^14^, Y. Ingrid Goh^16^, Dhriti Sharma^17^, Mekibib Altaye^18^, Susanne Benseler^19^, Hermine Brunner^18^, Andrea Knight^16^, for the CARRA NPSLE Workgroup^20^

#### ^1^Cincinnati Children’s Hospital Medical Center, Cincinnati, OH. University of Cincinnati College of Medicine, Cincinnati, OH. Johns Hopkins University, Baltimore, MD, USA; ^2^Indiana University School of Medicine, Riley Hospital for Children, Indianapolis, Indiana, USA; ^3^Saint Louis University School of Medicine, SSM Health Cardinal Glennon Children’s Hospital, USA; ^4^University of Campinas, Sao Paulo, Brazil; ^5^Baylor College of Medicine, Texas Children’s Hospital, Houston, Texas, USA; ^6^Atrium Health Wake Forest Baptist, Brenner Children’s Hospital, North Carolina, USA; ^7^University of Colorado School of Medicine, Children’s Hospital Colorado, USA; ^8^Michigan State University, Grand Rapids, Michigan, USA; ^9^Ann & Robert H. Lurie Children’s Hospital of Chicago, Illinois, USA; ^10^Nationwide Children’s Hospital, Ohio, USA; ^11^The Hospital for Sick Children and University of Toronto, Canada; ^12^Johns Hopkins University, Baltimore, MD, USA; ^13^The Hospital for Sick Children; ^14^Family Advisory Council, Neuropsychiatric Workgroup, Childhood Arthritis and Rheumatology Research Alliance; ^15^Family Advisory Council, Neuropsychiatric Workgroup, Childhood Arthritis and Rheumatology Research Alliance. University of Pittsburgh, Pittsburgh, Pennsylvania, USA; ^16^The Hospital for Sick Children and SickKids Research Institute, Toronto, Canada; ^17^Cincinnati Children’s Hospital Medical Center, Cincinnati, OH, USA; ^18^Cincinnati Children’s Hospital Medical Center, Cincinnati, OH, USA. University of Cincinnati College of Medicine, Cincinnati, OH, USA; ^19^University of Calgary, Calgary, Canada; ^20^Childhood Arthritis and Rheumatology Research Alliance (CARRA)

##### **Correspondence:** Ekemini Ogbu

*Pediatric Rheumatology 2024*, **22(S1):**A42

Background : Neuropsychiatric systemic lupus erythematosus (NPSLE) is associated with significant morbidity and mortality in childhood-onset systemic lupus erythematosus (cSLE). Despite the high frequency and burden of NPSLE in cSLE, it is arguably the least understood manifestation of this disease. High-quality, collaborative studies are still needed to address important gaps in knowledge and care of NPSLE in cSLE, guide management and improve outcomes for affected children, adolescents, and young adults. An important first step in achieving this is developing a prioritized and patient-centered research agenda that broadly incorporates perspectives from researchers, clinicians, patients, and caregivers affected by NPSLE. Thus, our objective was to derive this research agenda which would determine the: (1) high priority research areas for understanding the biology of NPSLE and improving care; (2) best research approaches to addressing the identified high priority areas, and (3) top barriers and facilitators of advancing NPSLE research and care.

Methods : The US Agency of Healthcare Research and Quality Prioritization Criteria Methods were used for this project. A multidisciplinary, international NPSLE taskforce was convened in October 2022. The NPSLE taskforce was comprised of pediatric rheumatologists, pediatric neurologists, psychiatrists, psychologists, research coordinators, and a family advisory council of patients/caregivers from Childhood Arthritis and Rheumatology Research Alliance (CARRA) sites (Table 1). Overarching research domains were initially identified by discussion with project leads and CARRA NPSLE workgroup members. The taskforce conducted a scoping review of published literature on NPSLE over a 22-year period (2000 – 2022), expanded the research domains based on identified knowledge and research gaps, and developed a list of research topics. These overarching research domains and topics were then refined and revised at the CARRA 2023 Annual Meeting. Consensus on the domains and topics will be achieved by the NPSLE taskforce and then distributed via an online survey to CARRA members, and pediatric neurology, psychiatry, and psychology national societies with interest in NPSLE. The research topics will be ranked in order of importance, feasibility and actionability.

Results : The 4 initial research domains identified were: diagnosis, treatment, clinical course, and outcomes. Following review of 163 publications on NPSLE, a total of 12 research domains were derived based on identified knowledge gaps (Table 2). Each research domain had 4 - 13 research topics.

Conclusions : This prioritized, and patient-centered internationally endorsed research agenda will inform strategic research that would improve outcomes for NPSLE in cSLE.

IRB Statement : This study was deemed exempt by the Cincinnati Children’s Hospital Medical Center Institutional Review Board.

Acknowledgements : This publication is based on research supported by the Lupus and Allied Diseases Association, Inc. and the Childhood Arthritis and Rheumatology Research Alliance. The authors wish to acknowledge CARRA and the ongoing Arthritis Foundation financial support of CARRA.

**Table 1 (Abstract A42):** Composition of the 26-member pediatric neuropsychiatric lupus taskforce

	N
Pediatric Rheumatologists	12
Pediatric Neuroimmunologists	2
Psychiatrists	2
Psychologists	2
Research Coordinators	2
Family Advisory Council	6

**Table 2 (Abstract A42):** Taskforce identified domains for addressing research gaps in pediatric neuropsychiatric lupus

Initial Research Domains	Final Research Domains
Diagnosis	Economic impact
Treatment	Psychosocial impact
Clinical course	Epidemiology
Outcomes	Predictors and Risk factors
	Pathoetiology
	Biomarkers
	Classification
	Diagnosis
	Treatment
	Clinical course
	Outcomes
	Quality of research studies

## A43 CD169 expression on monocytes is a useful marker for assessing type I interferon status in pediatric inflammatory diseases

### Takuya Mimura^1^, Tadafumi Yokoyama^2^, Naoto Sakumura^2^, Masaaki Usami^2^, Yusuke Matsuda^2^, Taizo Wada^2^

#### ^1^Kanazawa University, Kanazawa, Ishikawa, Japan; ^2^Kanazawa University

##### **Correspondence:** Takuya Mimura

*Pediatric Rheumatology 2024*, **22(S1):**A43

Background : Type I interferons (IFNs) had an important role in various inflammatory or autoimmune diseases. However, evaluation of type I IFNs is challenging because of their rapid clearance in peripheral blood. Type I IFN gene signature has recently been used to evaluate the IFN status, but this is often a labor-intensive and time-consuming method. Therefore, we assessed feasibility of measuring expression of an CD169 (Siglec-1), an IFN-inducible protein, on monocytes as alternative markers for type I IFN status in various pediatric inflammatory diseases.

Methods : After stimulating peripheral blood mononuclear cells (PBMCs) from healthy donors with human IFN-α or lipopolysaccharide (LPS) for 12 hours, PBMCs were analyzed for CD169 expression on monocytes. We also examined CD169 expression on monocytes by varying stimulating IFN-α concentration. We collected data from flow cytometric analysis of surface CD169 on monocytes and an ELISA of IFN-α and soluble CD169 in peripheral blood, and compared these data in various pediatric inflammatory diseases including viral infection, bacterial infection , systemic lupus erythematosus (SLE), Kikuchi-Fujimoto disease (KFD), Kawasaki disease (KD) and inflammatory bowel disease (IBD). We also conducted a time-series analysis of CD169 expression on monocytes in patients with KFD, anti-melanoma differentiation-associated gene-5 positive juvenile dermatomyositis (MDA5+JDM), Sjögren’s syndrome (SjS) and SLE. In SLE patients, the correlation between various clinical data and CD169 expression on monocytes was also analyzed.

Results : After stimulating PBMCs with IFN-α, CD169 expression was increased in CD14+ monocytes but unchanged in CD14dim CD16+ monocytes. LPS stimulation alone did not increase CD169 expression on CD14+ monocytes. In experiments conducted with varying concentrations of IFN-α, higher IFN-α resulted in stronger expression of CD169 in CD14+ monocytes. In analysis of comparison of the expression level of CD169 on monocytes in various inflammatory diseases, the expression of CD169 was significantly increased in patients with viral infection, SLE and KFD. On the contrary, CD169 expression on monocytes did not increase in patient with bacterial infection, KD and IBD. Circulating IFN-α levels were higher than HCs in viral infection and KFD, but not in SLE. Soluble CD169 levels was elevated only in viral infection compared to HCs. The time-series analysis revealed that CD169 expression on monocytes decreased after the acute phase in KFD and MDA5+JDM patients but was observed even 2 years after onset in SjS and SLE patients. Furthermore, CD169 on monocytes in SLE patients was found to have a negative correlation with white blood cell count, lymphocyte count and C4 levels.

Conclusions : CD169 expression on monocytes increases in patients with type I interferon-associated inflammatory diseases, making it a useful diagnostic marker. Furthermore, in diseases with a monophasic course, CD169 expression on monocytes may also serve as a disease activity marker.

IRB Statement : The study protocol was reviewed and approved by the Ethics Committees of Kanazawa University, School of Medicine (protocol number: 113822-1).

## A44 Relationship between brain injury markers and executive function in children with systemic lupus erythematosus and healthy controls

### Oscar Mwizerwa^1^, Justine Ledochowski^1^, Tala El Tal^1^, Sarah Mossad^1^, Victoria Lishak^1^, Joanna Law^1^, Lawrence Ng^1^, Paris Moaf^1^, Asha Jeyanathan^1^, Adrienne Davis^1^, Linda Hiraki^1^, Deborah Levy^1^, Joan Wither^2^, Zahi Touma^2^, Ashley Danguecan^1^, Andrea Knight^3^

#### ^1^The Hospital for Sick Children, Toronto, Canada; ^2^UHN; ^3^The Hospital for Sick Children and SickKids Research Institute, Toronto, Canada

##### **Correspondence:** Oscar Mwizerwa

*Pediatric Rheumatology 2024*, **22(S1):**A44

Background : Patients with childhood-onset systemic lupus erythematosus (cSLE) commonly experience impaired executive function (EF), and attribution to neuropsychiatric lupus (NPSLE) is challenging. Serum markers of brain injury may be useful potential biomarkers for EF in NPSLE. We investigated the relationship between serum brain injury markers and EF in cSLE.

Methods : We utilized prospectively-collected cross-sectional data from children with SLE ages 12-17 years recruited from the SickKids Lupus Clinic from January 2020–December 2022, and age, sex-matched healthy controls. Serum brain injury marker levels for glial fibrillary acidic protein (GFAP), serum neurofilament light chain (sNFL), and Tau were quantified using the Simoa Human Neurology 4–Plex B assay (Quanterix, Billerca, MA, USA). EF was assessed using the Delis Kaplan Executive Function System (DKEFS) Color Word Interference Test. Scaled scores were derived for the Inhibition and Inhibition/Switching conditions (normative mean= 10, standard deviation= 3; lower scores indicate more difficulties), which measure cognitive inhibition and mental flexibility, respectively. Descriptive data included disease duration, activity (SLEDAI-2K), damage (SLICC damage index, SDI>0) and clinical manifestations. We compared brain injury marker levels between the cSLE and control groups using the Wilcoxon rank-sum test and examined associations between the markers and EF scores (Inhibition and Switching) using Spearman correlations.

Results : Participants included 31 children with cSLE (mean age=15.6 years ± SD 1.5, 87% female) and 30 healthy controls (mean age=15.5 years ± 1.6, 83% female). For cSLE, the median disease duration was 27.7 months (IQR 20.9-50.7), median disease activity was 2 (IQR 2-4), 13% had disease damage, and one had a NPSLE diagnosis. There were no statistically significant differences between median levels of brain injury markers in the cSLE group versus controls for GFAP (77.6, 52.7-122 vs 69.1, 54-91.4), sNFL (4.9, 3.5-6.3 vs 5.4, 4-8) or Tau (2.6, 1.8-3.9 vs 2.5, 1.9-3.3). Scores for EF measures in the cSLE group versus controls were similar on the inhibition (median 9, 8-12 vs 12, 10-13) and inhibition/switch trials (10, 8-12 vs 11, 9-13). There was no correlation between brain injury markers and EF scores across the whole cohort. In the cSLE group, worse performance on DKEFS Inhibition correlated with higher sNFL (*r*=-0.29, *p*=0.11) and GFAP (*r*=-0.28, *p*=0.13), though not statistically significant.

Conclusions : Our study results indicate similar levels of brain injury markers and EF in group-wise comparisons of cSLE and controls, and a possible relationship between EF and the markers in the cSLE group. Further analysis will investigate the relationship between these markers and other disease features.

IRB Statement : These data are part of a larger project titled “A Prospective study of Multi-level Biomarkers for Pediatric Neuropsychiatric Lupus” with REB approval number (REB#: 1000063027)

Acknowledgements : This project received sponsorship from the Lupus Research Alliance

## A45 Successful implementation of a mental health screening program for youth with juvenile myositis

### Y. Ingrid Goh^1^, Kayla Baker^2^, Audrey Bell-Peter^2^, Vanessa Carbone^2^, Brian Feldman^2^, Luana Flores Pereira^2^, Jayne MacMahon^2^, Valerio Maniscalco^2^, Jo-Anne Marcuz^2^, Greta Mastrangelo^2^, Tanya Slater^2^, Kristi Whitney^2^, Andrea Knight^1^

#### ^1^The Hospital for Sick Children and SickKids Research Institute, Toronto, Canada; ^2^The Hospital for Sick Children, Toronto, Canada

##### **Correspondence:** Y. Ingrid Goh

*Pediatric Rheumatology 2024*, **22(S1):**A45

Background : High levels of emotional distress have been reported in children with juvenile myositis (JM). Inadequate recognition of mental health concerns by healthcare providers (HCPs) can contribute to poor disease outcomes. A multicenter study recently confirmed the feasibility and acceptability of mental health screening (MHS) in patients with JM. The global aim of this project was to implement MHS with referrals to an integrated social worker as standard of care for patients ≥5 years of age seen in the SickKids JM clinic. The specific aim was to increase the rate of MHS from 0 to ≥50% in a 90-day period and ensure that all patients with moderate/severe screening results received referrals for social work assessment.

Methods : A multidisciplinary stakeholder team iteratively developed screening and referral workflows and created educational resources for HCPs and families. All patients attending JM clinic were screened [12 to 18 years old: Generalized Anxiety Disorder-7 (GAD-7) and Patient Health Questionnaire (PHQ-9); caregivers of 5 to < 12 years old: Pediatric Symptom Checklist (PSC-17)]. All patients were to receive a mental health resource handout and patients with positive screens were to be referred to the clinic’s social worker. HCPs were to document MHS in patients’ After Visit Summary (AVS) and chart. Feedback from HCPs and patients/caregivers who agreed to participate in surveys informed successive plan, do, see, and act (PDSA) cycles.

Results : Workflows were iteratively developed to ensure that all HCPs agreed on their roles. Educational materials were created to educate HCPs and families. Reminders were placed in examination rooms and SmartPhrases were created to facilitate documentation. All thirty-two patients attending clinic were screened during three PDSA cycles from March 7-May 30, 2023. They all received the mental health resource handout and all positive screens were referred to the integrated social worker except for one, where the patient was being seen as a secondary consult. On average, documentation was completed 58% in the patient’s AVS and 90% in the patient’s chart. All patient surveys had positive feedback about the MHS process.

Conclusions : The JM clinic has successfully implemented MHS for all of its patients within 90-days. Additional PDSA cycles are needed to monitor its sustainability as well as monitor the impact of workflow changes when the hospital implements an institution-wide MHS program. We plan to expand MHS to incorporate immediate family members in the future.

IRB Statement : This quality improvement project has been approved by the SickKids Quality Management Office.

Acknowledgements : The authors would like to acknowledge CureJM for supporting this project.

## A46 Increased incidence of adverse events and events of special interest with treatment intensification in non-systemic JIA

### Paivi Miettunen^1^, Luca Carlini^2^, Angela Pistorio^2^, Violeta Panaviene^3^, Jordi Anton Lopez^4^, Sylvia Kamphuis^5^, Troels Herlin^6^, Pavla Dolezalova^7^, Marco Cattalini^8^, Helga Sanner^9^, Gordana Susic^10^, Maria Maggio^11^, Soad Hashad^12^, Reem Abdwani^13^, Donato Rigante^14^, Ana Rodriquez Lozano^15^, Chiara Pallotti^16^, Joost Swart^16^, Nicolino Ruperto^17^

#### ^1^IRCCS Istituto Giannina Gaslini, Pediatric and Rheumatology Clinic, Genoa, Italy; Alberta Children’s Hospital and University of Calgary; ^2^IRCCS Istituto Giannina Gaslini, Pediatric and Rheumatology Clinic, Genoa, Italy; ^3^Children’s Hospital, Affiliate of Vilnius University Hospital Santaros Klinikos, Vilnius, Lithuania; ^4^Institut de Recerca Sant Joan de Déu - SJD Barcelona Children’s Hospital, Barcelona, Spain; ^5^Polikliniek Kinderimmunologie en -reumatologie, Sophia Kinderziekenhuis, Erasmus MC - Erasmus Medisch Centrum, Rotterdam, Neatherlands; ^6^Department of Paediatrics and Adolescent Medicine, Aarhus University Hospital, Aarhus, Denmark; ^7^Charles University in Prague, Prague, Chech Republic; ^8^Università degli Studi di Brescia, Brescia (UNIBS), Italy; ^9^Department of Rheumatology, Oslo University Hospital, Oslo, Norway; ^10^Institute of Rheumatology Belgrade, Serbia; ^11^University Department PROMISE “G. D’Alessandro”, University of Palermo, Palermo, Italy; ^12^Tripoli Children’s Hospital, Tripoli, Libya; ^13^Sultan Qaboos University, Muscat, Dubai; ^14^Department of Life Sciences and Public Health, Fondazione Policlinico Universitario A. Gemelli IRCCS, Rome, Italy and Università Cattolica Sacro Cuore, Rome, Italy; ^15^Immunology Department, Instituto Nacional de Pediatría, Mexico City, Mexico; ^16^CS Istituto Giannina Gaslini, UOSID Centro Trial, PRINTO, Genoa, Italy; ^17^Istituto Giannina Gaslini,UOC Servizio di Sperimentazioni Cliniche Pediatriche, PRINTO, Genoa, Italy

##### **Correspondence:** Paivi Miettunen

*Pediatric Rheumatology 2024*, **22(S1):**A46

Background : It is not known if patients’ adverse event (AE) profile worsens, as their treatment is intensified. Our goal was to report AEs of at least moderate intensity, serious AE and events of special interest (ESI) in non-systemic-JIA patients as they progressed from less intensive treatment with non-steroidal anti-inflammatory drugs (NSAIDs) to treatment with conventional synthetic and biologic DMARDs (csDMARDs/bDMARDs) with data from Pharmachild registry.

Methods : Inclusion criteria were children with non-systemic-JIA as per ILAR criteria with whole drug exposure from onset to last observation. Data was available from 1987 until December 2021. Non-systemic-JIA patients were classified according to their treatment (chosen by the treating physician) into either a “Control group” (CG) (NSAIDs +/- intra-articular (IA) glucocorticoids only) or a “STEP-up group” (starting with NSAIDS +/- IA glucocorticoids but subsequently requiring DMARDS as required for disease control): STEP-1 (NSAIDs +/- intra-articular glucocorticoids); STEP-2 (csDMARDs +/- oral glucocorticoids), and STEP-3 (bDMARDs +/- other medications). AEs were classified by the latest version of MedDRA dictionary (Version 23.1). Statistical analysis included descriptive statistics and Cox multivariate regression model.

Results : A total of 8052 non-systemic JIA patients (69.9% female) were included: 719 (8.95%) in CG; 7333 (91.1%) in STEP-1; 6856 (85.1%) in STEP-2 and 5052 (62.7%) in STEP-3. The most frequent JIA category was oligoarthritis (76 % in CG versus 39.1% in STEP-1). The median (IQR) duration of each treatment period was 5.54 (2.88-9.18) years: CG 2.65 (1.04-6.38), STEP-1 0.62 (0.25-1.79), STEP-2 1.59 (0.57-3.85) and STEP-3 3.13 (1.48-5.58) years. Methotrexate (94%) and Etanercept (70%) were the most frequent conventional synthetic and biologic DMARDs, respectively. AEs were seen in all groups, least frequently in STEP-1 (1.8%) and most frequently in STEP-3 (24.4%). SAEs were most common in STEP-3 (492 (8%) patients), and rare in STEP-1 (62 (0.8%) patients) and CG (6 ( 0.8%) patients). Infections were the most frequent AEs in all groups (Incidence rates 0.33-4.14/100 patient years), with bDMARD group having the highest rate. Gastrointestinal disorders were most frequent in STEP-2 (N, IR, 95% CI: 330, 1.81 (1.62- 2.01), all other AEs in STEP-3. Patients treated with bDMARDs had the highest risk for development of first episodes of AE, SAE, infection and serious infection, with highest hazard ratio for serious infection (HR, 95% CI) at 19.17 (9.74- 37.74).

Conclusions : This is the first attempt to present a dynamic AE profile in a very large sample of non-systemic-JIA patients from an international pharmacovigilance registry applying a novel method of using patients as their “own controls”. Risk of AEs increased and AE severity worsened with treatment intensification, with bDMARDs associated with a higher prevalence of AE, SAE and ESI when compared to treatment with csDMARDS or NSAIDs. The most frequent ESIs were infections.

IRB Statement : Ethics approval: Pharmachild registries obtained approval from their respective ethics committees and were conducted in accordance with the Declaration of Helsinki. All subjects provided written informed consent/assent based on existing national regulations. The Pharmachild registry is registered at Clinicaltrials.gov (NCT01399281) and at the European Network of Centres for Pharmacoepidemiology and Pharmacovigilance (ENCePP) (http:// www.encepp.eu/encepp/viewResource.htm?id=19362).

## A47 Disparities in disease course of children with lupus by neighborhood-level child opportunity

### Joyce Chang^1^, Gabrielle Alonzi^1^, Emily Smitherman^2^, Pooja Patel^3^, Gabrielle Morgan^3^, Livie Huie^2^, Karen Costenbader^4^, Mary Beth Son^1^

#### ^1^Boston Children’s Hospital, Boston, MA, USA; ^2^University of Alabama at Birmingham, Birmingham, AL, USA; ^3^Ann & Robert H. Lurie Children’s Hospital of Chicago, Chicago, IL, USA; ^4^Mass General Brigham, Boston, MA, USA

##### **Correspondence:** Joyce Chang

*Pediatric Rheumatology 2024*, **22(S1):**A47

Background : Structural racism segregates children belonging to minoritized groups into neighborhoods with lower childhood opportunity, defined as resources and conditions that promote healthy childhood development. We determined whether lower neighborhood-level opportunity associates with more severe disease presentation or higher disease activity over time among children with systemic lupus erythematosus (SLE).

Methods : We linked medical records to census tract-level data for children with SLE at 3 tertiary centers (2016-2022): *N*=148 Boston Children’s Hospital (BCH); *N*=270 Lurie Children’s Hospital of Chicago (LCH); *N*=70 Children’s Hospital of Alabama (CHA). The index visit was the first visit with a physician diagnosis of SLE. The primary outcome was severe initial disease presentation (composite of Systemic Lupus Erythematosus Disease Activity Index (SLEDAI-2K) ≥10, intensive care admission, or dialysis). For patients diagnosed after 2016, SLEDAI-2K scores over time were used as a secondary outcome. Geocoded addresses were linked to census tract-level Child Opportunity Index (COI) 2.0 (29 indicators across education, socioeconomic status, physical environment) and Black or Hispanic Location Quotients (residential segregation relative to larger metropolitan area). Associations between COI and outcomes were estimated using logistic regression or linear mixed effects models. Covariates included age at SLE onset, sex, race, ethnicity, non-English primary language, insurance status, nephritis and neurologic involvement.

Results : We present results from *N*=148 patients in the BCH SLE cohort. Most (57%) were privately insured, 36% presented with nephritis, 8% required intensive care at presentation, and median index SLEDAI-2K was 10 [IQR 6,18]. Nationally ranked COI was skewed towards very high (36%) and high (19%) average opportunity. 75 (51%) patients met criteria for severe presentation. Living in areas with low vs. very high state-ranked opportunity areas associated with 4-fold higher adjusted odds of severe presentation (Table 1). For 86 patients diagnosed with SLE after 2016, there were 644 calculable SLEDAI-2K scores (median of 7/patient [2-11]). The majority of Black (13/18, 72%) and Hispanic children (16/22, 73%) lived in low/very low state-ranked COI areas, whereas 62% of non-Hispanic White and 73% of Asian children lived in high/very high COI areas. Living in areas with moderate, low or very low state-ranked COI (vs. very high) associated with significantly higher SLEDAI-2K over time, both with or without adjustment for time, insurance status, race and ethnicity, and major organ involvement (Table 2). There was a significant test of trend (*p*=0.001). Results were similar after adjusting for SLEDAI-2K at presentation (Figure 1).

Conclusions : For children with SLE in a U.S. region with high average opportunity, lower relative neighborhood-level child opportunity associated with more severe initial disease presentation and higher disease activity during follow-up. Area-level conditions, among other factors in lower resourced areas, may drive inequitable SLE outcomes at numerous points, including initial access to and after establishing subspecialty care.

IRB Statement : This study was granted an exemption and waiver of consent by the Boston Children’s Hospital Institutional Review Board for secondary use of existing data.

Acknowledgements : This work was funded by the Childhood Arthritis and Rheumatology Research Alliance (CARRA). The authors wish to acknowledge CARRA and the ongoing Arthritis Foundation financial support of CARRA.

**Table 1 (Abstract A47):** Individual and area-level factors associated with higher SLE disease severity at initial presentation to Boston Children’s Hospital

		Unadjusted			Adj. demographics + COI category			Adj. demographics + Location Quotient		
	*N*=148	OR	95% CI	*p*	aOR	95% CI	*p*	aOR	95% CI	*p*
Age at diagnosis (yr), mean (SD)	14 (3)	**0.9**	**[0.8 - 1.0]**	^*^	**0.9**	**[0.8 - 1.0]**	^*^	**0.9**	**[0.8 - 1.0]**	^*^
Female, n (%)	125 (85)	0.6	[0.3 - 1.5]		0.5	[0.2 - 1.3]		0.5	[0.2 - 1.5]	
Race and ethnicity										
Asian, non-Hispanic^a^	24 (16)	1.4	[0.5 - 3.9]		1.5	[0.5 - 4.8]		1.1	[0.4 - 3.4]	
Black	31 (21)	1.4	[0.5 - 3.6]		1.0	[0.3 - 3.2]		0.7	[0.2 - 2.3]	
Hispanic Non-White/Non-Black	27 (18)	1.3	[0.5 - 3.3]		1.0	[0.3 - 3.7]		0.8	[0.2 - 2.8]	
Hispanic White	8 (5)	0.1	[0.0 - 1.3]		**0.1**	**[0.0 - 1.0]**	^*^	**0.1**	**[0.0 - 1.0]**	^*^
Other, Non-Hispanic^b^	7 (5)	0.4	[0.1 - 2.3]		0.3	[0.1 - 1.9]		0.3	[0.1 - 1.8]	
Unknown	9 (6)	0.8	[0.2 - 3.4]		0.8	[0.2 - 4.3]		0.6	[0.1 - 3.2]	
White, Non-Hispanic	42 (28)	-	[ref]							
Non-English primary language	18 (12)	1.0	[0.4 - 2.6]		0.6	[0.2 - 2.1]		0.6	[0.2 - 2.1]	
Private vs. Public Insurance	85 (57)	0.7	[0.4 - 1.4]		0.7	[0.3 - 1.7]		0.7	[0.3 - 1.7]	
COI state ranked										
Very Low	33 (22)	1.5	[0.5 - 4.3]		1.6	[0.5 - 5.3]		-		
Low	36 (24)	**2.8**	**[1.0 - 8.1]**	^*^	**4.1**	**[1.2 - 13.4]**	^*^			
Moderate	23 (16)	1.5	[0.5 - 4.6]		3.5	[0.9 - 13.2]				
High	30 (20)	1.6	[0.6 - 4.7]		2.1	[0.7 - 6.7]				
Very High	26 (18)	-	[ref]							
Black Location Quotient ≥1.0^c^	48 (32)	**2.3**	**[1.1 - 4.8]**	^*^	-			**2.5**	**[1.1 - 5.9]**	^*^
Hispanic Location Quotient ≥1.0^c^	53 (36)	1.6	[0.8 - 3.2]		-			-		

Unadjusted and multivariable adjusted logistic regression models of high disease severity (SLEDAI-2K ≥10, intensive care admission, or dialysis) at initial presentation to Boston Children’s Hospital for *N*=148 SLE patients still followed in rheumatology clinic between 2016-2022

*COI* Child Opportunity Index version 2.0

^*^ *p*-value <0.05

^a^ncluding one patient self-reported as Native Hawaiian

^b^including one patient self-reported as American Indian and one as Middle Eastern

^c^Highly Black/White segregated or Hispanic/non-Hispanic segregated census tract relative to metropolitan area

**Table 2 (Abstract A47):** Association between relative childhood opportunity within state and disease activity over time among incident SLE cases at Boston Children’s Hospital, 2016-2022

	Univariable^a^			Multivariable		
	β	95% CI	*p*	β	95% CI	*p*
Child Opportunity Index						
Very High	*(reference)*					
High	1.5	[-0.7, 3.8]		1.4	[-0.5, 3.3]	
Moderate	**3.0**	**[0.6, 5.5]**	^*^	**2.7**	**[0.7, 4.7]**	^**^
Low	**3.9**	**[1.8, 6.0]**	^***^	**3.7**	**[2.0, 5.4]**	^***^
Very Low	**2.7**	**[0.7, 4.7]**	^**^	**2.9**	**[1.3, 4.6]**	^**^
Insurance status						
Public	1.2	[-0.4, 2.8]		1.4	[-0.1, 3.0]	
Private	*(reference)*					
Other/Uninsured	**2.1**	**[1.0, 3.2]**	^***^	1.6	[0.0, 3.2]	
Race						
Asian	2.3	[-0.4, 5.1]		1.1	[-0.7, 2.9]	
Black	1.8	[-0.2, 3.8]		-1.2	[-3.4, 1.1]	
Hispanic	1.0	[-0.9, 3.0]		-1.7	[-3.5, 0.1]	
Other, non-Hispanic	-0.8	[-3.0, 1.4]		-0.4	[-3.7, 2.9]	
Unknown	1.7	[-2.2, 5.6]		-1.0	[-3.8, 1.9]	
White, non-Hispanic	*(reference)*					
Non-English language	0.7	[-1.5, 2.9]				
Age at SLE onset	-0.2	[-0.4, 0.1]				
Male sex	0.5	[-1.0, 2.1]				
Nephritis at presentation	**3.8**	**[2.4, 5.2]**	^***^	**3.1**	**[1.7, 4.5]**	^***^
Neurologic involvement at presentation	**4.2**	**[1.8, 6.6]**	^***^	**3.4**	**[1.6, 5.2]**	^***^
Initial SLEDAI-2K at presentation	**0.1**	**[0.0, 0.2]**	^**^			
Time (month)	**-0.3**	**[-0.4, -0.2]**	^***^	**-0.3**	**[-0.4, -0.2]**	^***^
Time-squared	**0.004**	**[0.002, 0.006]**	^***^	**0.004**	**[0.003, 0.006]**	^***^

Linear mixed effects models with random slope and random intercept, robust variance estimators for *N*=86 patients with 644 evaluable visits

^*^ *p*<0.05

^**^ *p*<0.01

^***^ *p*<0.001

^a^All models adjusted for follow-up time and time-by-time interaction

**Fig. 1 (Abstract A47):**
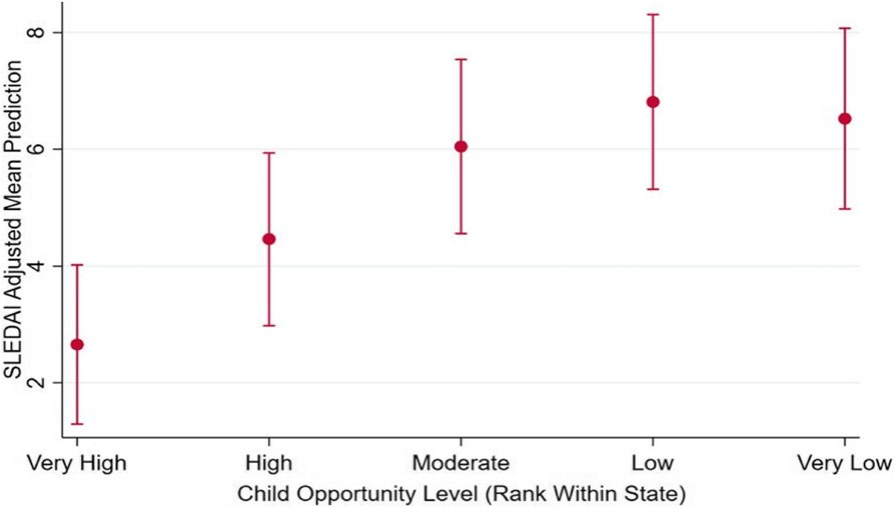
Disease activity during follow-up increases with decreasing neighborhood opportunity, adjusted for initial disease activity. Marginal mean predicted SLEDAI-2K scores at rheumatology follow-up visits by state-ranked Child Opportunity Index level, adjusted for follow-up time, insurance status, race and ethnicity, nephritis at presentation, neurologic involvement at presentation, and initial SLEDAI-2K score at the index visit

## A48 Speaking the same language: international cross-validation of emerging biomarkers for juvenile idiopathic arthritis

### Grant Schulert^1^, Rebecca Marsh^1^, Carine Wouters^2^, Patrick Matthys^3^, Dirk Foell^4^, Scott Canna^5^, Giusi Prencipe^6^, Claudia Bracaglia^6^, Fabrizio De Benedetti^6^, Dilan Dissanayake^7^, Ronald Laxer^7^, Sebastiaan Vastert^8^, Kelly Brown^9^, David Cabral^9^, Christoph Kessel^5^, For the CARRA FROST Investigators^10^

#### ^1^Cincinnati Children’s Hospital Medical Center, Cincinnati, OH, USA; ^2^UZ Leuven, Belgium; ^3^KU Leuven, Belgium; ^4^University of Muenster, Germany; ^5^Children’s Hospital of Philadelphia, Philadelphia, PA, USA; ^6^Bambino Gesù Children’s Hospital, Rome, Italy; ^7^Hospital for Sick Children, Toronto, Canada; ^8^UMC Utrecht, Netherlands; ^9^British Columbia Children’s Hospital, Vancouver, Canada; ^10^Childhood Arthritis and Rheumatology Research Alliance (CARRA)

##### **Correspondence:** Grant Schulert

*Pediatric Rheumatology 2024*, **22(S1):**A48

Background : To date, several studies have validated the use of key biomarkers such as IL-18, CXCL9 and S100 proteins in diagnosis and monitor treatment response of systemic juvenile idiopathic arthritis (sJIA). Despite the promise of these biomarkers, their clinical utility is limited by their overall lack of standardization. The objective of this study is to cross-validate emerging systemic JIA biomarkers across different measurement platforms and different international centers to facilitate their wider introduction into routine clinical care.

Methods : Healthy donor serum samples spiked with defined concentrations of recombinant S100 proteins, CXCL9, CXCL10, IL-18 and sCD25 were distributed in blinded manner among all participating centers (CARRA member sites: Cincinnati, Philadelphia, Toronto, Vancouver; PReS centers: Leuven, Muenster, Rome, Utrecht). Individual spiked protein levels were determined using locally established platforms including commercial ELISA, commercial/custom luminex, Ella and Mesoscale. Data were analyzed for variances across different platforms and agreement across identical platforms in different labs, and used to generate conversion factors for standardization. In the second step, baseline and follow-up serum and plasma samples from the FROST study will be distributed for analysis of each biomarker on at least two platforms. Results will be adjusted using the proposed conversion factors.

Results : We observed extremely tight correlation of spiked biomarker levels with the amounts quantified by the employed measurement platforms (IL-18 (Figure 1) R2=0.744-0.999, *P*< 0.0001; CXCL9 (Figure 2) R2=0.924-0.999, *P*< 0.0001; S100A8/A9 R2=0.926-0.963, *P*< 0.0001; S100A12 R2=0.955-0.969, *P*< 0.0001; CXCL10 0.926-0.963, *P*< 0.0001). However, the actual spike recovery of the individual assays varied substantially. While some assays met 90-100% spike recovery over almost the entire tested concentration range (1pg-500ng/mL), others consistently yielded high (approx. 500%) or low (i.e. 15-70%) spike recovery. Interestingly, CXCL9 levels (Figure 2) were determined by Ella at three centers in three different countries and yielded nearly identical spike recovery. Further, our data determined the lower level of detection for each. In general, Luminex and Ella assays performed better at very low (sub-physiological) concentrations. FROST samples have been distributed to Cincinnati, Muenster, and Rome, are currently being assayed for IL-18, CXCL9, S100A8/A9, and S100A12.

Conclusions : Our spike recovery approach demonstrates high correlation of individual assay results but widely divergent absolute concentrations measured. Importantly, the same platforms appear to yield highly comparable results across countries. From our data we can now clearly identify assays with almost perfect spike recovery and calculate conversion factors for those that over- or underperform in their concentration output. Altogether, the results from our study will enable wide interpretation and translation of respective biomarker data and pave the way towards their wider use in routine clinical practice and international collaborative studies.

IRB Statement : The CARRA Registry and Biosample consent have been IRB approved, and all patients who provided biosamples provided informed consent.

Acknowledgements : The authors wish to acknowledge CARRA and the ongoing Arthritis Foundation financial support of CARRA. This work is funded by a CARRA-PReS Collaborative Research Grant. FROST: This work could not have been accomplished without the aid of the following organizations: The NIH’s National Institute of Arthritis and Musculoskeletal and Skin Diseases (NIAMS) & the Arthritis Foundation. We would also like to thank all participants and hospital sites that recruited patients for the CARRA Registry. The authors thank the following CARRA Registry site principal investigators, sub- investigators and research coordinators: R. Agbayani, S. Akoghlanian, E. Allenspach, E. Anderson, S. Ardoin, S. Armendariz, I. Balboni, L. Ballenger, S. Ballinger, F. Barbar-Smiley, K. Baszis, H. Bell-Brunson, H. Benham, W. Bernal, T. Bigley, B. Binstadt, M. Blakley, J. Bohnsack, A. Brown, M. Buckley, D. Bullock, B. Cameron, S. Canna, E. Cassidy, J. Chang, V. Chauhan, T. Chinn, P. Chira, A. Cooper, J. Cooper, C. Correll, L. Curiel-Duran, M. Curry, A. Dalrymple, D. De Ranieri, F. Dedeoglu, M. DeGuzman, N. Delnay, V. Dempsey, J. Dowling, J. Drew, K. Driest, Q. Du, D. Durkee, M. Eckert, C. Edens, M. Elder, S. Fadrhonc, L. Favier, B. Feldman, I. Ferguson, B. Ferreira, L. Fogel, E. Fox, R. Fuhlbrigge, J. Fuller, N. George, D. Gerstbacher, M. Gillispie-Taylor, I. Goh, D. Goldsmith, S. Grevich, T. Griffin, M. Guevara, P. Guittar, M. Hager, T. Hahn, O. Halyabar, M. Hance, S. Haro, J. Harris, J. Hausmann, K. Hayward, L. Henderson, A. Hersh, S. Hillyer, L. Hiraki, M. Hiskey, P. Hobday, C. Hoffart, M. Holland, M. Hollander, M. Horwitz, J. Hsu, A. Huber, M. Ibarra, C. Inman, S. Jackson, K. James, G. Janow, S. Jones, K. Jones, J. Jones, C. Justice, U. Khalsa, B. Kienzle, S. Kim, Y. Kimura, M. Kitcharoensakkul, T. Klausmeier, K. Klein, M. Klein-Gitelman, S. Kramer, J. Lai, B. Lang, S. Lapidus, E. Lawson, R. Laxer, P. Lee, T. Lee, M. Lerman, D. Levy, S. Li, C. Lin, N. Ling, M. Lo, S. Lvovich, J. Maller, A. Martyniuk, K. McConnell, I. McHale, E. Meidan, E. Mellins, M. Miller, R. Modica, K. Moore, T. Moussa, V. Mruk, E. Muscal, K. Nanda, L. Nassi, J. Neely, L. Newhall, P. Nigrovic, B. Nolan, E. Oberle, O. Okeke, M. Oliver, K. O’Neil, R. Oz, A. Paller, J. Patel, P. Pepmueller, K. Phillippi, R. Pooni, S. Protopapas, B. Puplava, S. Radhakrishna, S. Ramsey, H. Reid, S. Ringold, M. Riordan, M. Riskalla, M. Ritter, M. Rodriquez, K. Rojas, M. Rosenkranz, T. Rubinstein, C. Sandborg, L. Scalzi, K. Schikler, K. Schmidt, E. Schmitt, R. Schneider, C. Seper, J. Shalen, R. Sheets, S. Shenoi, J. Shirley, E. Silverman, V. Sivaraman, C. Smith, J. Soep, M. Son, L. Spiegel, H. Stapp, S. Stern, A. Stevens, B. Stevens, K. Stewart, E. Stringer, R. Sundel, M. Sutter, R. Syed, R. Syed, T. Tanner, G. Tarshish, S. Tarvin, M. Tesher, A. Thatayatikom, B. Thomas, D. Toib, K. Torok, C. Toruner, S. Tse, T. Valcarcel, N. Vasquez, R. Vehe, J. Velez, E. von Scheven, S. Vora, L. Wagner-Weiner, D. Wahezi, M. Waterfield, P. Weiss, J. Weiss, A. White, L. Woolnough, T. Wright, M. Yee, R. Yeung, K. Yomogida, Y. Zhao, A. Zhu.

**Fig. 1 (Abstract A48):**
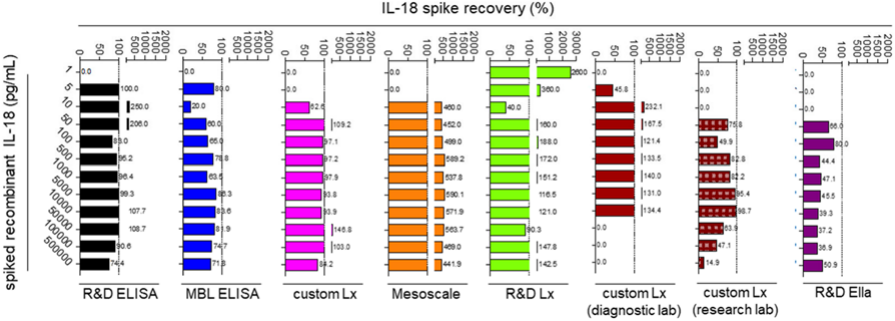
See text for description.

**Fig. 2 (Abstract A48):**
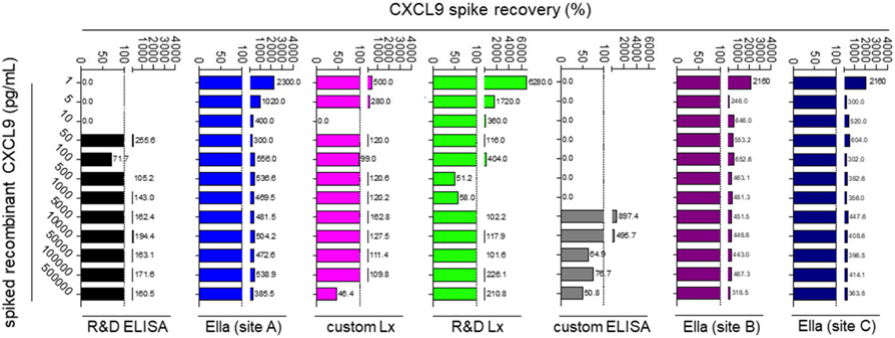
See text for description.

## A49 Delay in the diagnosis of juvenile idiopathic arthritis in Mexico

### Jimena Garcia-Silva, Alondra Correa-Galvan, Ana Leos-Leija, Maria de Lourdes Aldana-Galvan, Ana Villarreal-Treviño, Fernando Garcia-Rodriguez, Nadina Rubio-Perez

#### Hospital Universitario “Dr. Jose Eleuterio Gonzalez” Universidad Autónoma de Nuevo León; Monterrey, México

##### **Correspondence:** Jimena Garcia-Silva

*Pediatric Rheumatology 2024*, **22(S1):**A49

Background : Juvenile Idiopathic Arthritis (JIA) is a rare disease, with a global prevalence of 32.6 per 100,000 caucasian children, not well-known by pediatricians and general practitioners, which leads to delayed diagnosis and treatment. Timely referral to a pediatric rheumatology center and effective care are critical to changing the natural history of the disease and improving patients’ long-term prognosis. The journey of patients with JIA to reach a pediatric rheumatologist is usually complex and frequently involves visits to multiple medical specialists, taking around 3 months in countries such as France and Germany. We aimed to evaluate the time between the onset of symptoms and diagnosis in patients with JIA and the demographic factors related to the delay.

Methods : Ambispective observational study of patients with JIA diagnosed at a pediatric rheumatology clinic in a period of 15 years (2008-2023). Descriptive statistics were carried out and an univariate analysis of demographic factors that affected delay in diagnosis was performed using SPSS v.27.

Results : A total of 133 patients with a diagnosis of JIA were included, 92 girls (69.2%), with an age at diagnosis of 9.8 (IQR 6.1-11.6) years and positive rheumatoid factor polyarticular JIA as the most frequent subtype (47, 35.3%). The median time between the onset of symptoms and diagnosis was 143 (IQR 63-295) days and between the onset of symptoms and the patients’ first visit to our center was 119 (IQR 54-273) days. There was no significant difference in the delay related to JIA subtypes or sex. The median time to diagnosis after the patients’ first visit to our center was 2 (IQR 0-22) days, but in the oligoarticular and enthesitis-related arthritis subtypes this time increased to 15 (IQR 0-39) and 22 (IQR 7-34) days, respectively (*p*=0.003).

Conclusions : Our study have showed the important delay to diagnosis that exists in our population. This highlights the importance of education about pediatric rheumatic diseases, in addition to the need for qualitative studies to explore factors related to the delay of referral and diagnosis to be able to address this problem in our country and improve the outcomes.

IRB Statement : This study complies with the ethical guidelines of the Declaration of Helsinki and local regulations. This is a minimal-risk study, all the procedures were revised and approved by the ethics committee of our institution (Facultad de Medicina y Hospital Universitario, Universidad Autónoma de Nuevo León).

## A50 Early biologic use improves disease trajectories in STOP-JIA patients through 3 years

### Mei Sing Ong^1^, Sarah Ringold^2^, Marc Natter^3^, George Tomlinson^4^, Laura Schanberg^5^, Brian Feldman^6^, Vincent Del Gaizo^7^, Yukiko Kimura^8^, for the CARRA STOP-JIA Investigators^7^

#### ^1^Harvard Medical School & Harvard Pilgrim Health Care Institute, Boston, MA, USA; ^2^Seattle Children’s Hospital, Seattle, WA, USA; ^3^Boston Children’s Hospital, Boston, MA, USA; ^4^University of Toronto, Toronto, Ontario, Canada; ^5^Duke University School of Medicine, Durham, NC USA; ^6^The Hospital for Sick Children, Toronto, Ontario, Canada; ^7^Childhood Arthritis and Rheumatology Research Alliance (CARRA); ^8^Hackensack Meridian Health, Hackensack, NJ, USA

##### **Correspondence:** Mei Sing Ong

*Pediatric Rheumatology 2024*, **22(S1):**A50

Background : Although conventional synthetic disease-modifying antirheumatic drugs (csDMARDs) and biologic DMARDs (bDMARDs) have vastly improved polyarticular juvenile idiopathic arthritis (pJIA) outcomes, questions remain regarding the ideal timing of bDMARD initiation. In a previous analysis of the Start Time Optimization of Biologics in Polyarticular JIA (STOP-JIA) study, we showed that early initiation of a bDMARD (within 3 months of study enrollment) was associated with rapid improvement of disease activity over a one-year period. Here, we extended the analysis to evaluate disease trajectories through 3 years following study enrollment.

Methods : We applied latent class trajectory modeling (LCTM) to evaluate the trajectories of STOP-JIA patients through 3 years following study enrollment, as defined by clinical Juvenile Arthritis Disease Activity Score (cJADAS10). Study cohort was confined to those with cJADAS10 measurements at baseline and at ≥ 1 follow-up visit in both years 2 and 3. Multiple imputation was used for missing data.

Results : Of the 400 patients enrolled in STOP-JIA, 259 were included. LCTM identified 2 distinct trajectories (Figure 1): (1) Latent Class 1 (*n*=54; 21%), characterized by slow improvement of disease activity and failure to achieve inactive disease (cJADAS10≤2.5) through 3 years; and (2) Latent Class 2 (*n*=205; 79%), characterized by rapid improvement of disease activity, achievement of inactive disease within the first year, and maintenance of inactive disease at 3 years. The odds of experiencing the more favorable trajectory (Class 2) were greatest among patients treated with bDMARDs within the first month (OR 3.02; 95% 1.47 – 6.67), and diminished with increasing delay in initiating bDMARD (Figure 2). Furthermore, there was a dose-response relationship between total days of bDMARD use within the first 3 months and the likelihood of being in the more favorable trajectory (OR 1.26; 95% 1.04 – 1.53).

Conclusions : Our findings provide further evidence that early bDMARD use has a favorable impact on the disease trajectories of pJIA patients through a 3-year timeframe.

IRB Statement : This study was approved by the Harvard Pilgrim Health Care Institute Institutional Review Board (#2004526-3).

Acknowledgements : This study was funded by CARRA. The authors wish to acknowledge CARRA and the ongoing Arthritis Foundation financial support of CARRA. Post-hoc analysis of STOP-JIA data was funded by a CARRA Data Analysis Support Grant. This work could not have been accomplished without the aid of the following organizations: The NIH’s National Institute of Arthritis and Musculoskeletal and Skin Diseases (NIAMS) & the Arthritis Foundation. We would also like to thank all participants and hospital sites that recruited patients for the CARRA Registry. The authors thank the following CARRA Registry site principal investigators, sub- investigators and research coordinators: R. Agbayani, S. Akoghlanian, E. Anderson, K. Baszis, H. Bell-Brunson, H. Benham, S. Benseler, H. Brunner, E. Chalom, J. Chang, V. Chauhan, T. Chinn, A. Cooper, L. Curiel-Duran, T. Davis, J. Dean, F. Dedeoglu, V. Dempsey, J. Dowling, J. Drew, M. Eckert, B. Feldman, K. Fields, C. Fleming, I. Goh, B. Gottlieb, T. Graham, T. Griffin, M. Guevara, M. Hance, M. Hollander, S. Hong, J. Hsu, A. Huber, A. Huttenlocher, C. Inman, H. Jackson, J. Jaquith, S. Jared, S. Jones, S. Kim, D. Kingsbury, K. Klein, M. Klein-Gitelman, S. Kramer, B. Malla, M. Mannion, A. Martyniuk, T. Mason, K. Mcallister, K. McConnell, I. McHale, K. Moore, E. Muscal, S. Nativ, K. O’Neil, K. Onel, J. Patel, S. Protopapas, B. Puplava, J. Quach, C. Rabinovich, S. Ringold, M. Riordan, M. Ritter, K. Rojas, M. Rosenkranz, K. Schikler, T. Seay, C. Smith, H. Stapp, K. Stewart, J. Sumner, R. Syed, M. Tesher, A. Thatayatikom, R. Vehe, N. Volpe, D. Wahezi, A. Watts, J. Weiss, P. Weiss, A. Zhu.

**Fig. 1 (Abstract A50):**
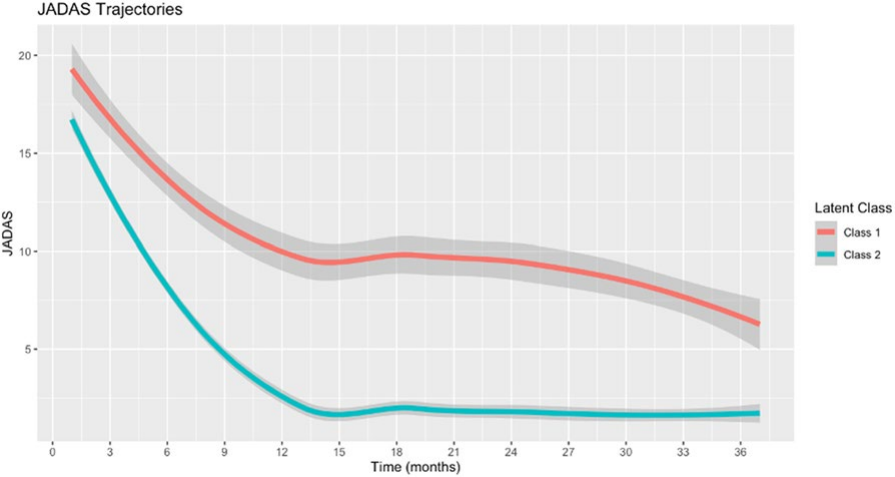
LCTM analysis of disease trajectories

**Fig. 2 (Abstract A50):**
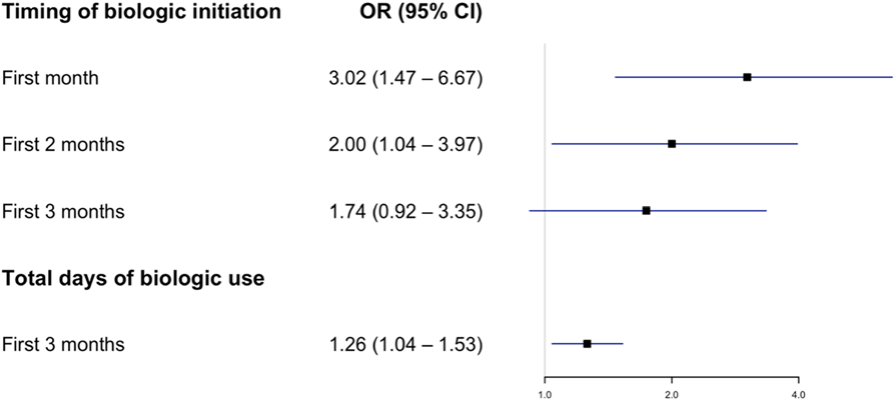
The association of biologic use with trajectory class 2

## A51 Physician perspectives on multidisciplinary lupus nephritis clinics

### Anita Dhanrajani^1^, Rebecca Sadun^2^, for the CARRA Investigators^3^

#### ^1^Children’s Hospital of New Orleans, New Orleans, LA, USA; ^2^Duke University School of Medicine, Durham, NC, USA; ^3^Childhood Arthritis and Rheumatology Research Alliance (CARRA)

##### **Correspondence:** Anita Dhanrajani

*Pediatric Rheumatology 2024*, **22(S1):**A51

Background : Patients with lupus nephritis (LN) have a 26 times higher risk of mortality compared to those with lupus but no renal involvement. Studies on patients with adult-onset lupus have found that multidisciplinary models are effective in delivering better quality of care. A single-center, cross-sectional study from Rush University in Chicago found that lupus patients seen in a dedicated lupus clinic performed better with disease specific quality measures, compared to those seen in a general rheumatology clinic. Another study exploring subspecialty multidisciplinary clinics in adults with LN demonstrated that wait times to diagnose LN decreased by 40%. There are, however, no studies specific to multidisciplinary clinics in childhood LN. Nevertheless, a 2019 multidisciplinary prioritization exercise concluded that there is a strong need for multidisciplinary collaboration in the management of childhood lupus. Research in other chronic diseases such as cancer and cystic fibrosis has shown that multi-disciplinary models of care improve disease related and patient reported outcomes.

Methods : This was a mixed methods prospective study involving members of the CARRA LN workgroup (focus group) and across CARRA sites (survey). The focus group sessions used semi-structured interviews, during which data was gathered from CARRA physician members on clinic structure and on barriers and challenges for multidisciplinary LN clinics. The themes arising from the focus group guided the development of a 25-item survey that was disseminated electronically to all CARRA provider members. Participation was requested from one member per site to avoid duplication. The survey collected data around structure of multidisciplinary clinics such as types of specialists involved, presence and functions of patient care navigator, billing models, presence of trainees, outcome measures collected during clinic, and free-response questions about potential challenges.

Results : After eliminating duplicate sites and incomplete responses, 29 final responses were included in the data analysis. Sixteen (55%) respondents indicated that they have an existing multidisciplinary lupus nephritis clinic, 9 (31%) said they had neither an existing clinic nor any plans to start one in the future, 3 (10%) said that plans were underway to start a clinic within the next one year, and 1 indicated they tried to start a clinic in the past (Table 1). All 16 sites that had a multidisciplinary clinic have both a rheumatologist and a nephrologist collaborating in the clinic. Table 1 shows details of clinical staffing at all centers. The majority of sites (11/17, 64.7%) revealed that their clinic caters specifically to patients with lupus nephritis, whereas a few sites include all patients with lupus (*n*=2), complex lupus nephritis only (*n*=3), or only incipient lupus (*n*=1).

Conclusions : Several models of multidisciplinary lupus clinics exist in North America. Further qualitative data is needed to elucidate the benefits and drawbacks to these clinics, as well as barriers and facilitators to their development and smooth function. Future work will also focus on multidisciplinary lupus clinic outcomes, including patient reported outcomes (PROs).

IRB Statement : This research was in accordance with the Declaration of Helinski and approved by the University of Mississippi medical center Institutional review board ethics committee. (UMMC-IRB-2022-462)

Acknowledgements : The authors wish to acknowledge CARRA and the ongoing Arthritis Foundation financial support of CARRA.

**Table 1 (Abstract A51):** Clinic staffing

**Pediatric Specialists**					
**Rheumatologist**	**Nephrologist**	**Adolescent medicine**	**Pulmonologist**	**Dermatologist**	**Other**
16	16	2	1	1	1 (Pediatric & adolescent gynecologist)
**Allied Health Professionals**					
**Psychologist**	**Physical Therapist**	**Occupational Therapist**	**Pharmacist**	**Dietitian**	**Social worker**
1	2	1	2	3	8
**Trainees/Advance Practice Providers**					
**Rheumatology fellow**	**Nephrology fellow**	**Pediatric resident**	**Physician assistant**	**Nurse practitioner**	**Other**
11	10	9	1	1	1 (Gynecology fellow)

Out of 16 respondents, the numbers above indicate how many clinics involved each of the various physician specialists, allied health professionals, trainees, and advanced practice providers

**Fig. 1 (Abstract A51):**
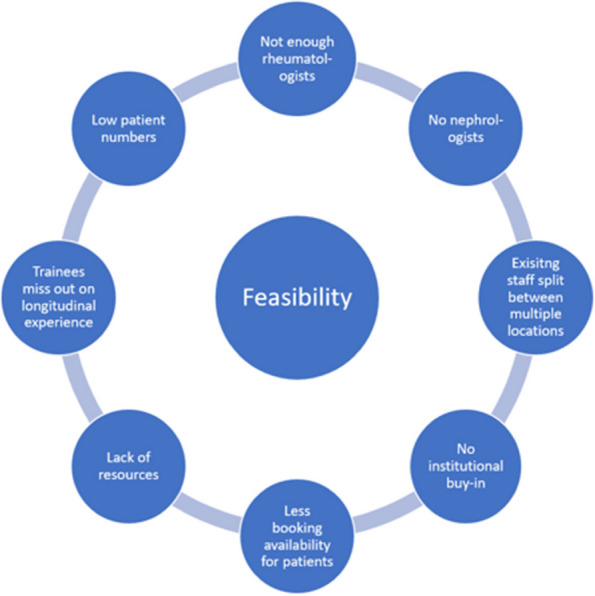
Barriers and challenges

## A52 Baseline clinical features and biomarker profiles of the childhood arthritis and rheumatology research alliance (CARRA) systemic juvenile idiopathic arthritis-associated lung disease (SJIA-LD) Cohort

### Esraa Eloseily^1^, Autumn Clark^2^, Min-Lee Chang^3^, MaryEllen Riordan^4^, Alan Russell^5^, Marc Natter^3^, Sherry Thornton^6^, Yukiko Kimura^4^, Grant Schulert^7^, for the CARRA Registry SJIA-LD Cohort Investigators^8^

#### ^1^Cincinnati Children’s Hospital, Cincinnati, OH, USA; ^2^Cincinnati Children’s Hospital Medical Center Burnet Campus: Cincinnati Children’s Hospital Medical Center; ^3^Boston Children’s Hospital, Boston, MA USA; ^4^Hackensack Meridian Health, Hackensack, NJ, USA; ^5^Duke Clinical Research Institute, Durham, NC USA; ^6^University of Cincinnati, Cincinnati, OH, USA; ^7^Cincinnati Children’s Hospital Medical Center, Cincinnati, OH, USA; ^8^Childhood Arthritis and Rheumatology Research Alliance (CARRA)

##### **Correspondence:** Esraa Eloseily

*Pediatric Rheumatology 2024*, **22(S1):**A52

Background : Systemic juvenile idiopathic arthritis (SJIA) associated lung disease (SJIA-LD) is an emerging and life-threatening clinical problem with urgent unmet needs including prevalence, pathogenesis, disease biomarkers, influence of biologics, and outcomes.

Methods : Existing or newly enrolled CARRA Registry patients with SJIA and suspected, probable, or definite SJIA-LD were included in the cohort. In addition to standard Registry data, lung disease specific data was obtained using a standardized case report form through REDCap Cloud, and biosamples collected when available. Biomarker profiles were determined from plasma using a custom Luminex panel. This study was approved by the DCRI Reliant IRB and/or IRB of all Registry sites.

Results : 37 patients were enrolled in the SJIA-LD cohort, from 16 CARRA Registry sites in the US. 46% had definite (biopsy-proven), 36% probable, and 18% suspected SJIA-LD. Demographic and clinical features are shown in Table 1. Of those who underwent lung biopsy, all had pulmonary alveolar proteinosis (PAP) and interstitial inflammation, and 40% had collagenous fibrosis. 77% had at least one definite episode of macrophage activation syndrome (MAS) (including 64% that met the 2016-SJIA-MAS criteria), 73% had more than one MAS episode, and 32% had subclinical MAS. MAS occurred prior to SJIA-LD diagnosis in 68% and coincided with it in 18%. Across all patient samples, SJIA-LD patients showed significantly increased plasma levels of IL-6, IL-12, IL-18, CXCL9, CD25, CCL11, CCL17, MCP-1, and MCP-3 compared to healthy pediatric controls. Cluster analysis defined 3 distinct groups of SJIA-LD patients. Group 1 (*n*=7) showed high levels of TNF, IL-6, IL-17, MCP-1 and 3, CCL11, and CCL17; group 2 (*n*=8) showed high IL-10, IL-12, IL-18, CXCL9, CXCL10, CD25, and CD163; and group 3 (*n*=10) showed high CCL15 and CCL25 (Figure 1).

Conclusions : Patients in the CARRA SJIA-LD cohort exhibit a broad spectrum of clinical and radiographic features, disease activity, and treatment approaches. Recurrent MAS was common. Patients with SJIA-LD showed multiple distinct plasma biomarker patterns. As an ongoing prospective cohort study of this emerging disease, we will be able to assess clinical features, longitudinal disease progression and trajectories, as well as associated immune biomarkers and cellular populations.

IRB Statement : This study was approved by the DCRI Reliant IRB and/or IRB of all Registry sites.

Acknowledgements : This project is supported by a CARRA-Arthritis Foundation Large Grant. The authors wish to acknowledge the Arthritis Foundation for their ongoing support of CARRA. Baylor/TCH: Marietta DeGuzman, Tiphanie Vogel; Boston Children’s: Fatma Dedeoglu, Lauren Henderson, Pui Lee; CCHMC: Alexi Grom, Grant Schulert, Tracy Ting; Children’s Mercy: Ashley Cooper; CHOP: Edward Behrens, Scott Canna, Pamela Weiss; Hackensack: Yukiko Kimura, Jennifer Weiss; Lurie Children’s Hospital: Marisa Klein-Gitelman; Minnesota: Colleen Correll, Patricia Hobday, Mona Riskalla; Nationwide: Stacy Ardoin; NIH: Michael Ombrello, Marianna Marquez; Phoenix Children’s: Kaleo Edo; Stanford: Joyce Hsu, Betsy Mellins, Vivian Saper; Seattle Children’s: Dan Zhao; UAB: Melissa Mannion; UCSF: Susan Kim, UoF: Leandra Woolnough; UIowa: Sandy Hong, Scott Lieberman; UPMC: Margalit Rosenkranz; Utah: CJ Inman; Vanderbilt: Brent Graham; Washington University: Kevin Baszis; Wisconsin: Dominic Cokra

**Table 1 (Abstract A52):** Demographic and clinical features of patients in the SJIA-LD cohort (*N*=37)

**Clinical feature** **(*N*=37)**	**Summary or median (IQR)**
Sex, n,%	23, 62% F
Age at SJIA onset, median	1 years (0.5-5)
Age at LD onset, median	4 years (2-8)
LD duration	23 months (18-28)
SJIA duration	74 months (42-107)
LD duration at enrollment, median (IQR)	28 months (7-46)
SJIA duration at LD diagnosis, median (IQR)	21.5 months (10.25-35.75)
SJIA disease duration at enrollment, median (IQR)	55 months (24-83)
LD Category	52% definite LD 33% probable LD 11% suspected LD
Clinical features at LD diagnosis	53% cough 47% clubbing 47% dyspnea on exertion 34% tachypnea 29% hypoxia 21% digital erythema
Baseline chest CT findings (*n*=35)	54.3% ground glass opacities 40% septal thickening 37% peribronchovascularthickening 22.9% peripheral consolidation 20% hilar adenopathy 20% pleural thickening
Pulmonary function tests (*n*=19)	54.4% abnormal DL 41.2% abnormal spirometry 6.5% abnormal pulse oximetry
Patient assessment of disease activity, median (IQR)	1 (3.8)
PGA, median (IQR)	0.8 (1.5)
PGALD, median (IQR)	3 (3)
Health Related quality of life	3 (13%) Excellent 9 (39%) Very good 4 (17.4%) Good 7 (30.4%) Fair
**Select Labs at baseline visit**	**Median (IQR)**
White blood cell count	8.8x109/L (6.8-13.4)
Hemoglobin	11.6 g/dL (10.6-12.7)
Platelet count	316.5 109/L (256.8-416)
ESR	9 mm/hr(5-23)
CRP	0.5 mg/dL (0.25-1.5)
Ferritin	56 ng/ml (29-121)
IL-18	24,336 pg/mL (4,147-49,275)
**Medication used**	**Summary**
csDMARDsever used	Methotrexate 45.7% Cyclosporin A 45.7%
bDMARDsand tsDMARDsever used	Anakinra 82.9% Canakinumab 57% Tofacitinib 57% Tocilizumab 46%
Taking oral steroids at baseline visit, Median dose (IQR)	42.9%, 6 mg/day (3-10)

**Fig. 1 (Abstract A52):**
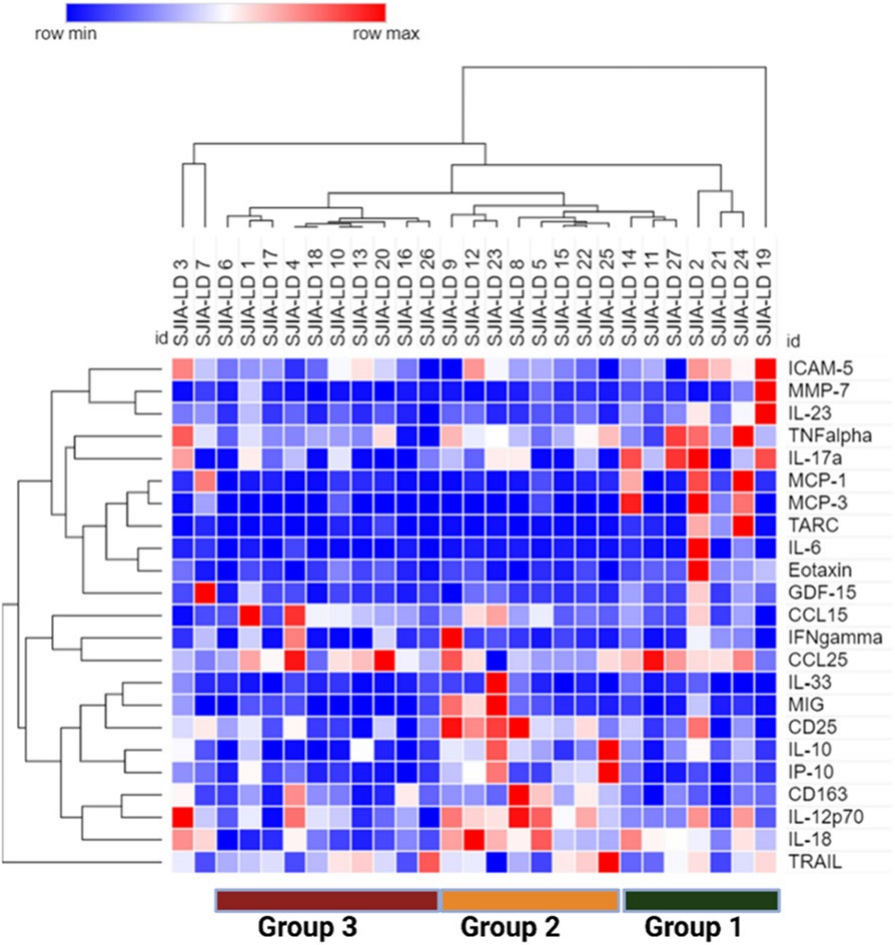
Heatmap showing plasma biomarker levels in SJIA-LD patients

## A53 Implementation of pneumococcal vaccination in pediatric patients with systemic lupus erythematosus: quality improvement initiative

### Julia Klauss^1^, Lauren Robinson^2^, Leanne Mansfield^3^, Karen Onel^1^, Nancy Pan^2^

#### ^1^Hospital for Special Surgery, New York, NY, USA; ^2^Weill Cornell Medical College, Hospital for Special Surgery, New York, NY; ^3^Division of Allergy, Immunology, and Rheumatology, University of Wisconsin School of Medicine and Public Health, Madison, WI

##### **Correspondence:** Julia Klauss

*Pediatric Rheumatology 2024*, **22(S1):**A53

Background : Patients with Systemic Lupus Erythematosus (SLE) are at increased risk for invasive pneumococcal infection. Additional pneumococcal vaccination is indicated for immunosuppressed patients with rheumatic disease per established guidelines (CDC, ACIP). Despite the established safety and immunogenicity of pneumococcal vaccinations in the childhood onset SLE population, vaccine uptake remains suboptimal. Pre-visit planning has been shown to improve rates of pneumococcal vaccination among patients with childhood onset SLE at a single center (Sivaraman V, et al, 2020). The specific aim of this quality improvement initiative was to increase the percentage of patients with childhood onset SLE at our center who were up to date with pneumococcal vaccination from a baseline of 6% to at least 30% by 6 months. Additionally, ongoing data was collected to assess the sustainability of these interventions over time.

Methods : Baseline pneumococcal vaccination rates were obtained via retrospective chart review of all SLE patient encounters for 3 months prior to the project start date. A patient was considered “up to date” with pneumococcal vaccination if they were not eligible for any additional pneumococcal vaccine doses by the end of the encounter. Plan-Do-Study-Act (PDSA) methodology was used. Interventions included establishing in-office vaccine supply, educational sessions with the multidisciplinary team, and pre-visit planning. Pre-visit planning consisted of reviewing the vaccine record and annotating the electronic medical record appointment notes with the recommended vaccination. Percentage of patients up to date with vaccination was recorded and reviewed by the team monthly.

Results : Our interventions led to a 6 point upward trend in the percentage of patients with SLE who were up to date with pneumococcal vaccination to a new baseline of 67% by 6 months (Figure 1). This improved vaccination rate was sustained for over one year with ongoing pre-visit planning but without additional follow-up interventions.

Conclusions : Our pneumococcal vaccination initiative involving subspecialty office vaccine administration and pre-visit planning led to a significant improvement in pneumococcal vaccination rates among patients with childhood SLE. We have demonstrated the reproducibility of these simple interventions to lead to sustained improvement in vaccination rates for children with SLE. Ongoing barriers to continued improvement may be persistent vaccine hesitancy and timing of vaccination in relation to B cell depleting therapy. Important future directions include updating these interventions to reflect the recent approval of PCV20 in children as well as measuring and intervening on vaccine hesitancy.

IRB Statement : Exemption was granted by Hospital for Special Surgery Institutional Review Board on 11/28/2023 under EXEMPT 45 CFR 46.104((d) Category #(4). Reference number: 2023-2396

Acknowledgements : We would like to acknowledge the contributions of the pediatric rheumatology department as well as the nursing staff.

**Fig. 1 (Abstract A53):**
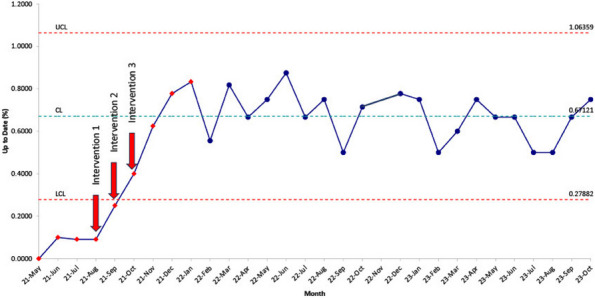
Pneumococcal Vaccination in Patients with SLE. Control chart showing percentage of patients up to date with pneumococcal vaccination. Intervention 1: establishment of in-office vaccine supply. Intervention 2: multidisciplinary education sessions. Intervention 3: pre-visit planning

## A54 Update on the child/adolescent lupus: understanding etiology (CLUE) study

### Linda Hiraki^1^, Susan Thompson^2^, Laura Lewandowski^3^, for the CARRA Registry Investigators^4^

#### ^1^The Hospital for Sick Children, Toronto, Ontario, Canada; ^2^Cincinnati Children’s Hospital Medical Center, Cincinnati, OH, USA; ^3^National Institute of Arthritis and Musculoskeletal and Skin Diseases, NIH, Bethesda, MD, USA; ^4^Childhood Arthritis and Rheumatology Research Alliance (CARRA)

##### **Correspondence:** Linda Hiraki

*Pediatric Rheumatology 2024*, **22(S1):**A54

Background : Genetics plays a critical role in determining who will get SLE. Genome-wide association studies (GWAS) of adults with SLE have identified >100 common variants for SLE. Prior studies have established a greater burden of these common SLE risk variants in children compared to adults with SLE. With increasing availability of whole genome sequencing (WGS), there is recognition of monogenic forms of SLE in some childhood-onset SLE (cSLE) patients. To date, there have been no comprehensive multi-center studies of genetic risk of cSLE. Defining the genetics of this disease is a first essential step towards understanding the biologic mechanisms leading to disease. To address this gap in knowledge, the CARRA Childhood Lupus Translational research Collaboration Hub (CLUTCH) launched the Child/adolescent Lupus study: “Understanding Etiology (CLUE)” study to complete whole genome sequencing of cSLE patients enrolled in the CARRA Lupus Registry.

Methods : From June 1, 2021 to June 30, 2023, we recruited prevalent and incident cSLE patients enrolled in the CARRA Lupus Registry and consented for the CARRA biorepository, who had not yet contributed a biosample to the CARRA biorepository. Detailed demographic and clinical information on all cSLE patients is available from the CARRA registry. We collected whole blood for DNA (EDTA) and RNA (Tempus tubes) samples from patients at any registry visit. We will also study the genetics of cSLE patients enrolled in the completed Atherosclerosis Prevention in Paediatric Lupus Erythematosus (APPLE) trial using DNA collected prospectively during the study. We will complete paired end whole genome sequencing (WGS) using and Illumina HiSeq X platform.

Results : We enrolled *n*=220 participants in CLUE, 9% of the Lupus Registry population. CLUE participants enrolled to date appear to be representative of the Lupus Registry population with a higher proportion of CLUE participants having lupus nephritis compared with the total Registry population (62% vs. 51%). WGS is complete for 202 cSLE patients from the APPLE trial, of which 14 have DNA from both parents for analysis.

Conclusions : CLUE has successfully enrolled and collected biospecimens from 220 participants in 2 years. The synergy of the CARRA Lupus registry and CLUE, as well as APPLE samples, will enable comprehensive genetic studies will improve our understanding of cSLE and may lead to developing effective, targeted therapies for SLE.

IRB Statement : Ethics approval has been granted by CARRA and the relevant collaborating sites involved in the study.

Acknowledgements : This project was funded by a CARRA-Arthritis Foundation Large Grant. This work could not have been accomplished without the aid of the following organizations: The NIH’s National Institute of Arthritis and Musculoskeletal and Skin Diseases (NIAMS), the Arthritis Foundation, and the Centers for Disease Control and Prevention (CDC). We would also like to thank all participants and hospital sites that recruited patients for the CARRA Registry. APPLE supported by the NIH (National Institute of Arthritis and Musculoskeletal and Skin Diseases contract N01-AR-2-2265), the Edna and Fred L. Mandel Jr. Center for Hypertension and Atherosclerosis, and Pfizer, which provided atorvastatin and matching placebo. The authors thank the following CARRA Registry site principal investigators: K. Abulaban, C. Aguiar Lapsia, S. Ardoin, L. Barillas-Arias, M. Basiaga, K. Baszis, H. Brunner, H. Bukulmez, E. Chalom, J. Chang, D. Co, K. Cook, A. Cooper, C. Correll, T. Davis, F. Dedeoglu, M. DeGuzman, A. Dhanrajani, K. Ede, B. Edelheit, B. Feldman, I. Ferguson, D. Glaser, D. Goldsmith, B. Gottlieb, T. Graham, T. Griffin, T. Hahn, L. Harel, O. Harry, M. Hollander, S. Hong, M. Horwitz, J. Hsu, A. Huber, L. Imundo, C. Inman, P. Kahn, S. Kim, D. Kingsbury, M. Klein-Gitelman, L. Lim, M. Mannion, D. McCurdy, D. Milojevic, S. Mohan, T. Moore, K. Moore, L. Moorthy, S. Nativ, M. Natter, K. Onel, J. Patel, S. Prahalad, C. Rabinovich, A. Robinson, T. Ronis, M. Rosenkranz, N. Ruth, S. Sabbagh, K. Schikler, C. Schutt, E. Sloan, J. Spitznagle, Y. Sterba Rakovchik, K. Stewart, G. Syverson, S. Tarvin, M. Tesher, D. Toib, M. Toth, M. Twilt, H. Van Mater, D. Wahezi, P. Weiss, J. Weiss, L. Woolnough, E. Wu, A. Yalcindag, Y. Zhao APPLE was supported by the following investigators: S. Ardoin, E.M. Dewitt, C.E. Rabinovich, J. Ellis, K. Mieszkalski, J. Wootton, P. Chira, J. Hsu, T. Lee, C. Sandborg, J. Perea, B. Gottlieb, P. Irigoyen, J. Luftig, S. Siddiqi, Z. Ni, M. Orlando, E. Pagano, A. Eichenfield, L. Imundo, D. Levy, P. Kahn, C. Batres, D. Cabral, K.A. Haines, Y. Kimura, S.C. Li, J. Weiss, M. Riordan, B. Vaidya, E. von Scheven, M. Mietus-Snyder, E. Silverman, L. Ng, S. Bowyer, S. Ballinger, T. Klausmeier, D. Hinchman, A. Hudgins, M. Punaro, S. Henry, S. Zhang, N.G. Singer, E.B. Brooks, S. Miner, N. Szabo, L. Scalzi, D. Sherry, L. Dorfeld, S. Wilson, J. Tress, D. McCurdy, T. Hernandez, J. Vitale, M. Klein-Gitelman, A. Kress, N. Lowe, F. Patel, C. Wallace, S. Hamilton, R. Silver, K. Caldwell, D. Kamen, L. Wagner-Weiner, B. Puplava, A. Lonchev, G. Higgins, M. Bacani, H. Brunner, C. Rutherford, J. Meyers-Eaton, S. Nelson, A. Grom, L. Jung, T. Conway, L. Frank, L. Kuss, J. Soep, H. Senz, A. Reed, T. Mason, J. Jaquith, D.E. Paepke-Tollefsrud.

## A55 The relationship between resilience and executive functioning in a cross-sectional sample of youth with childhood-onset systemic lupus erythematosus

### Ashley Danguecan^1^, Isabella Zaffino^1^, Joanna Law^1^, Kiah Reid^1^, Angela Cortes^1^, Eugene Cortes^1^, Sandra Williams-Reid^1^, Adrienne Davis^1^, Asha Jeyanathan^1^, Sona Sandhu^1^, Lawrence Ng^1^, Paris Moaf^1^, Deborah Levy^1^, Linda Hiraki^1^, Andrea Knight^2^

#### ^1^The Hospital for Sick Children, Toronto, Ontario, Canada; ^2^The Hospital for Sick Children and SickKids Research Institute, Toronto, Ontario, Canada

##### **Correspondence:** Ashley Danguecan

*Pediatric Rheumatology 2024*, **22(S1):**A55

Background : Executive functions (EFs) are cognitive skills that facilitate goal-directed behaviour. EFs are associated with mental health and disease outcomes, medication adherence, and successful transition from pediatric to adult healthcare; thus, EFs are important for disease self-management. EF difficulties are common in childhood onset systemic lupus erythematosus (cSLE), and are associated with depression symptoms, disease damage, pain, sleep disturbance and fatigue. In addition to these adverse outcomes, it is also important to identify factors that promote EF to develop effective interventions. Resilience is increasingly recognized as a protective factor for health outcomes, and is defined as one’s capacity to withstand or recover quickly from adversity. The current study examined the associations between EF and resilience in a pediatric cSLE cohort.

Methods : A cross-sectional sample of youth with cSLE aged 10-17 years from a major tertiary hospital SLE outpatient clinic participated in the study between January 2020 and September 2023. All patients met ACR and/or SLICC classification criteria for SLE. Participants completed the Behaviour Rating Inventory of Executive Function, Second Edition (BRIEF-2), a standardized questionnaire assessing three domains of EF: emotion regulation (e.g., controlling strong emotions), cognitive regulation (e.g., planning and organization), and behavior regulation (e.g., impulse control). The Connor-Davidson Resilience Scale 10 Item (CD-RISC 10) and the Child and Youth Resilience Measure Revised (CYRM-R) were also administered to measure individual psychological resilience (i.e., emotionally “bouncing back” from adversity) and socio-ecological resilience (e.g., feeling connected to family and community), respectively. We examined the associations between CD-RISC 10 or CYRM-R scores and each domain of EF using separate multivariable regression models, adjusting for disease duration. *P*-values <.05 were considered statistically significant.

Results : Twenty-five youth with cSLE (80% female) with mean age of 15.2 years (SD=1.8) completed the BRIEF-2, CYRM-R and CD-RISC 10; mean disease duration was 33.0 months (SD=31.2) and 48% had active disease (SLEDAI-2K >4) (see Table 1). We found a higher psychological resilience was significantly associated with fewer cognitive regulation difficulties (β = -.443, 95% CI -2.23, -.10, *p*=0.03). There was a trend between higher social-ecological resilience and fewer cognitive regulation difficulties (β = -.419, 95% CI -2.14, .05, *p*=0.06). Disease duration did not show any significant associations with EF. See Table 2.

Conclusions : In this small cohort of youth with cSLE, higher self-reported psychological resilience was associated with fewer EF difficulties in the domain of cognitive regulation, with a similar trending association between social-ecological resilience and cognitive regulation. Further examination of how to promote optimal EF and increase resilience in youth with cSLE is needed in larger samples, with the goal of promoting quality of life and disease self-management.

IRB Statement : This project has been approved by the Research Ethics Board at The Hospital for Sick Children (REB #1000078857).

**Table 1 (Abstract A55):** Demographic and clinical characteristics of the total sample (*n*=25)

**Demographic Characteristic**	**Descriptive Statistics**
Age in years, mean (SD)	15.2 (1.8)
Female sex, n (%)	22 (88%)
Race, n (%)	
Asian	16
Black	3
White	4
Other	2
Household Income, # above the poverty line (%)	20 (80%)
**Clinical Characteristics**	
Disease Duration in months, mean (SD)	33.0 (31.2)
Active Disease (SLEDAI-2K >4), n (%)	12 (48%)
Presence of Disease Damage (SDI >0), n (%)	0

**Table 2 (Abstract A55):** Multivariable regression analyses for associations between resilience and executive function domains

	Behavior Regulation^a^	Emotion Regulation^**a**^	Cognitive Regulation^**a**^
**Adjusted β, 95% CI,** ***p*-value**			
**Predictor:** Individual Psychological Resilience (CD-RISC total score)	-.120 (-1.64, .94), *p*=.580	.248 (-1.46, .39), *p*=.242	**-.443 (-2.23, -.10),** *p*=.033*
Disease Duration	-.156 (-.35, .17), *p*=.468	-.272 (-.30, .07), *p*=.201	-.193 (-.32, .11), *p*=.332
**Adjusted β, 95% CI,** ***p*-value**			
**Predictor:** Social-Ecological Resilience (CYRM-R total score)	-.334, (-1.96, .28), *p*=.136	-.163 (-1.28, .61), *p*=.474	-.419 (-2.14, .05), *p*=.061
Disease Duration (months)	-.263 (-.42, .11), *p*=.235	-.303 (-.33, .07), *p*=.190	-.293 (-.39, .08), *p*=.181

^*^denotes statistically significance at *p*<.05

^a^BRIEF-2 index T score were with mean = 50, SD=10; Higher scores indicate more difficulty

## A56 Implementation assessment of a treat to target strategy for lupus in the pediatric rheumatology clinic

### Emily Smitherman^1^, Julia Harris^2^, Aimee Hersh^3^, Jennifer Huggins^4^, Livie Huie^1^, Jon Burnham^5^

#### ^1^University of Alabama at Birmingham, Birmingham, AL, USA; ^2^Children’s Mercy Kansas City, Kansas City, MO, USA; ^3^University of Utah, Salt Lake City, UT, USA; ^4^Cincinnati Children’s Medical Center, Cincinnati, OH, USA; ^5^Children’s Hospital of Philadelphia, Philadelphia, PA, USA

##### **Correspondence:** Emily Smitherman

*Pediatric Rheumatology 2024*, **22(S1):**A56

Background : Achievement of a lupus low disease activity state (LLDAS) has been associated with less organ damage, fewer disease flares, and improved health-related quality of life in children with systemic lupus erythematosus (cSLE). No prior studies have evaluated the implementation of lupus disease activity measure collection in the real-world. Our objective was to evaluate the acceptability, appropriateness, and feasibility of implementing a Treat to Target strategy for lupus in the pediatric rheumatology clinic.

Methods : The Pediatric Lupus Understanding Systems (PLUS) collaborative was formed consisting of 5 pediatric rheumatology sites located in children’s hospitals affiliated with academic medical centers in the United States. We operationalized the 5 cLLDAS criteria to collect at the point of care (Table 1) with plans for implementation phase 1 to track collection of each criteria at cSLE visits on a monthly basis. We completed a baseline implementation assessment using the Acceptability of Intervention Measure, Intervention Appropriateness Measure, and Feasibility of Intervention Measure. Each measure consists of 4 domains assessed on a Likert scale of 1-5 (1=completely disagree to 5=completely agree). Mean response was calculated for each domain. Barriers to cLLDAS criteria collection were also identified.

Results : We collected a baseline implementation assessment from each PLUS collaborative site leader (*n*=5). Sites reported a range of number of pediatric rheumatology providers (5-18), most had trainees in clinic, and 60% have mid-level providers (Table 2). The approximate number of patients with cSLE seen in 2022 varied from 73-150 with a broad mix of insurance. All major electronic health record (EHR) vendors are represented. Implementing collection of cSLE disease activity measures and a Treat to Target strategy in the pediatric rheumatology clinic was largely found to be acceptable and appropriate (Figure 1). Scores for feasibility of the intervention were less positive. Themes of identified barriers to collection of cLLDAS criteria included: need for EHR adjustments to collect discrete data, availability of laboratory results to calculate disease activity during clinic visit, preexisting systems to collect physician global assessment on 0-10 scale, and physician burnout to change.

Conclusions : Implementation of a treat to target approach to care of patients with cSLE is acceptable and appropriate although will require a dedicated effort to be feasible. A key determinant to monitoring real-world performance is ability to customize EHR with discrete data fields. Next steps are to use implementation facilitation via monthly meetings to improve performance from baseline.

IRB Statement : This study was approved by the University of Alabama at Birmingham Institutional Review Board (IRB-300009852).

Acknowledgements : The authors wish to acknowledge CARRA and the ongoing Arthritis Foundation financial support of CARRA. This project was funded by a CARRA-Arthritis Foundation Implementation Science Testing Grant.

**Table 1 (Abstract A56):** Criteria required to calculate cLLDAS operationalized for collection at the point of care

Implementation Phase 1	SLEDAI-2K completion
	New disease activity attestation
	Physician global assessment (0-3) completion
	Prednisone (or equivalent) dose documented
	Standard medication dose attestation

**Table 2 (Abstract A56):** Baseline characteristics of the pediatric rheumatology sites participating in PLUS collaborative

Site	Providers	Trainees	Mid Level Providers	Patients with cSLE seen in 2022	EHR Vendor
1	18	Yes	Yes	110	Epic
2	11	Yes	No	150	Epic
3	8	No	No	91	Cerner
4	5	Yes	Yes	73	Allscripts
5	7	Yes	Yes	75	Cerner

**Fig. 1 (Abstract A56):**
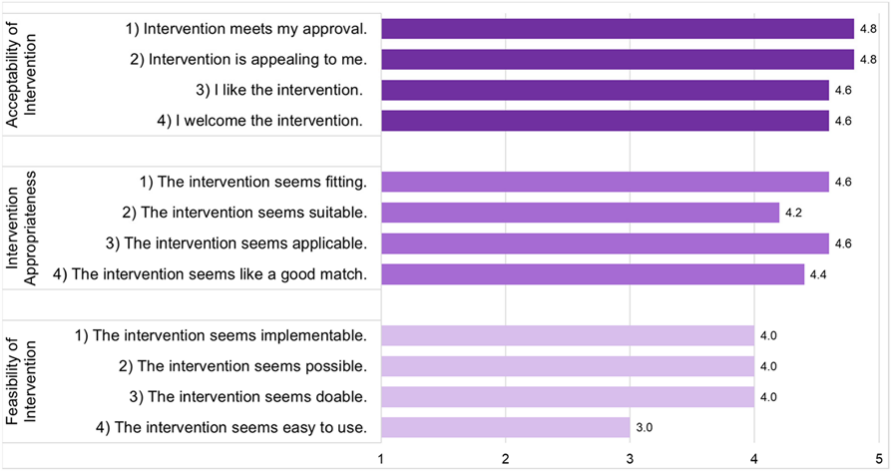
Mean response across 5 PLUS collaborative sites to each implementation measure domain using 5-point Likert scale from strongly disagree to strongly agree

## A57 Adalimumab use, deprescribing strategies, and quality of life within the CARRA registry among those with well-controlled JIA

### Anna Sutton^1^, Abner Nyandege^2^, Melissa Mannion^3^, Timothy Beukelman^4^, Brian Feldman^5^, Liangyuan Hu^2^, Marinka Twilt^6^, Ruud Verstegen^5^, Daniel Horton^2^, for the CARRA Registry Investigators^7^

#### ^1^University of Washington School of Public Health; ^2^Rutgers University, New Brunswick, NJ, USA; ^3^University of Alabama at Birmingham, Birmingham, AL, USA; ^4^University of Alabama at Birmingham, Birmingham, AL, USA; Childhood Arthritis and Rheumatology Research Alliance (CARRA), Washington, DC, USA; ^5^The Hospital for Sick Children, Toronto, Ontario, Canada; ^6^University of Calgary, Alberta Children’s Hospital, Calgary, AB, Canada; ^7^Childhood Arthritis and Rheumatology Research Alliance (CARRA)

##### **Correspondence:** Anna Sutton

*Pediatric Rheumatology 2024*, **22(S1):**A57

Background : Adalimumab is the most widely used biologic disease-modifying antirheumatic drug (DMARD) for juvenile idiopathic arthritis (JIA). While effective, chronic biologic use places burdens on patients and families that motivate some with well-controlled disease to taper or stop treatment. This study examined adalimumab deprescribing strategies (stopping or tapering), flares of disease, and patient/parent-reported outcomes (PROs) among CARRA Registry participants with well-controlled JIA.

Methods : We included participants in the CARRA Registry (2016-2022) with non-systemic JIA in clinical inactive disease (CID) on a stable dose of adalimumab. CID was defined by joint count=0, physician global assessment ≤1, and no active uveitis at two consecutive registry visits spaced ≥5 months apart (index date at the second qualifying visit). We excluded those with other rheumatic diseases, IBD, or who took glucocorticoids or new DMARDs at or between qualifying visits. Patient characteristics are reported from registry enrollment. We characterized patterns of adalimumab use and deprescribing during follow-up. We compared quality of life (QoL) and other PROs from the PROMIS Global Health Assessment taken at 6 ± 3 months after the index visit for patient groups of adalimumab continuation, tapering, or stopping without taper (groups not mutually exclusive) using Wilcoxon rank sum testing. Flares were defined as increasing ADA frequency after continuation or taper; restarting ADA after stopping; starting a new DMARD; new receipt of oral, intraarticular, or ocular glucocorticoids; joint count ≥1; physician global assessment >1; or new uveitis activity.

Results : Of 862 study participants, mean age was 7.6 years at JIA diagnosis and 11.8 years at study entry; most were white and from the US (Table 1). Two-thirds had either oligoarthritis (34%) or RF- polyarthritis (31%). Over follow-up time of 1.5 (IQR 0.9, 3.2) years, 808 (94%) continued with adalimumab, at least initially, while 336 (39%) had recorded deprescribing: 213 (25%) stopped without tapering after median 0.88 years of follow-up, and 123 (14%) tapered after median 0.96 years of follow-up (Table 2). Tapering occurred over a median of 6 months; most participants had only one observed tapering step (Table 2). While baseline PROs did not significantly differ among groups, QoL and several other PROs (general, physical health, overall instrument score) 6-months after the index visit were modestly higher in deprescribing groups (especially tapering) versus the continuation group (Table 3) despite higher rates of flares within 6 months after stopping (continue: 0%, stop without taper: 22%, taper: 1%).

Conclusions : Most patients with non-systemic JIA in the CARRA Registry continued on the effective dose of adalimumab at least 1 year after CID on medication. Among those with adalimumab deprescribing, most stopped without tapering, and tapers occurred over about 6 months. Patients stopping or tapering adalimumab had modestly higher quality of life than those continuing adalimumab, even with increased frequency of disease flare.

IRB Statement : This study was approved by the Rutgers IRB (Pro2023001269).

Acknowledgements : This project was supported with funding from CARRA and the Arthritis Foundation. We thank Alicia Iizuka and Matthew Iozzio (Rutgers University) for administrative assistance and Min-Lee Chang (Boston Children’s Hospital) for logistical support. This work could not have been accomplished without the aid of the following organizations: The NIH’s National Institute of Arthritis and Musculoskeletal and Skin Diseases (NIAMS) & the Arthritis Foundation. We would also like to thank all participants and hospital sites that recruited patients for the CARRA Registry. The authors thank the following CARRA Registry site principal investigators: K. Abulaban, C. Aguiar Lapsia, S. Ardoin, L. Barillas-Arias, M. Basiaga, K. Baszis, H. Brunner, H. Bukulmez, E. Chalom, J. Chang, D. Co, K. Cook, A. Cooper, C. Correll, T. Davis, F. Dedeoglu, M. DeGuzman, A. Dhanrajani, K. Ede, B. Edelheit, B. Feldman, I. Ferguson, D. Glaser, D. Goldsmith, B. Gottlieb, T. Graham, T. Griffin, T. Hahn, L. Harel, O. Harry, M. Hollander, S. Hong, M. Horwitz, J. Hsu, A. Huber, L. Imundo, C. Inman, P. Kahn, S. Kim, D. Kingsbury, M. Klein-Gitelman, L. Lim, M. Mannion, D. McCurdy, D. Milojevic, S. Mohan, T. Moore, K. Moore, L. Moorthy, S. Nativ, M. Natter, K. Onel, J. Patel, S. Prahalad, C. Rabinovich, A. Robinson, T. Ronis, M. Rosenkranz, N. Ruth, S. Sabbagh, K. Schikler, C. Schutt, E. Sloan, J. Spitznagle, Y. Sterba Rakovchik, K. Stewart, G. Syverson, S. Tarvin, M. Tesher, D. Toib, M. Toth, M. Twilt, H. Van Mater, D. Wahezi, P. Weiss, J. Weiss, L. Woolnough, E. Wu, A. Yalcindag, Y. Zhao.

**Table 1 (Abstract A57):** Demographic characteristics of children with well-controlled JIA on adalimumab within the CARRA Registry (*N*=862)

Characteristics	N (unless noted otherwise)	% (unless noted otherwise)
Demographics		
Age at JIA diagnosis	7.6 (mean)	5.0 (SD)
Age at index visit	11.8 (mean)	4.7 (SD)
Female sex	579	67.2
Race/Ethnicity		
White	666	77.3
Hispanic/Latino	59	6.8
Asian	24	2.8
Black	22	2.6
Other	89	10.5
Country		
US	811	94.1
Canada	37	4.3
Other/unknown	14	1.6
Index year		
2016-2018	219	25.5
2019-2020	395	45.8
2021-2022	248	28.8
Disease		
JIA category		
Oligoarthritis	293	34.0
RF- polyarthritis	271	31.4
Enthesitis-related arthritis	136	15.8
Psoriatic arthritis	86	10.0
RF+ polyarthritis	48	5.6
Undifferentiated arthritis	28	3.2
ANA+	406	47.1
Uveitis	226	26.2
Psoriasis	73	8.5
Sacroiliac joint arthritis	64	7.4
TMJ arthritis	44	5.1
Medications		
Medications at index date		
Methotrexate	428	49.7
Other cDMARD	44	5.1
Prior non-adalimumab bDMARD use	224	26.0
Adalimumab regimen at index		
Every other week	747	86.7
Weekly	115	13.3
Humira (vs. biosimilar)	849	99.8
Prior adalimumab use (days)	616 (median)	443, 957 (IQR)

*cDMARD* conventional disease-modifying anti-rheumatic drug, *bDMARD* biologic disease-modifying anti-rheumatic drug, *IQR* interquartile range

**Table 2 (Abstract A57):** Adalimumab use and deprescribing patterns among children with well-controlled JIA within the CARRA Registry

	***N***=862 (%)	Years follow-up time, median (IQR)
Treatment groups^a^		
Continued adalimumab use	808 (93.7%)	1.05 (0.46, 2.00) while continuing
Stop without taper	213 (24.7%)	0.88 (0.44, 1.56) until stopping
Taper	123 (14.3%)	0.96 (0.52, 1.56) until tapering
		0.49 (0.26, 0.94) while tapering
Tapering characteristics	***N*** **=123 (%)**	
Baseline adalimumab regimen		
Every other week	98 (79.7%)	
Weekly	25 (20.3%)	
Number of observed taper steps before stopping or end of follow-up		
One	103 (83.7%)	
Two	15 (12.2%)	
Three	1 (0.8%)	
Four	3 (2.4%)	

^a^Groups not mutually exclusive; 12 participants stopped without taper on the index date; 24 participants started tapering on the index date

**Table 3 (Abstract A57):** Quality of life and other patient/parent-reported outcomes (PROs) 6 months after adalimumab continuation, stop without taper, or taper

PRO^1^	Continue (***N***=363)		Stop without taper (***N***=122)		Taper (***N***=75)		
	Mean	Median (IQR)	Mean	Median (IQR)	Mean	Median (IQR)	***P***-value^**2**^
Quality of life	4.30	4 (4, 5)	4.39	5 (4, 5)	4.57	5 (4, 5)	0.02
General health	3.99	4 (3, 5)	4.05	4 (3, 5)	4.28	4 (4, 5)	0.02
Physical health	3.91	4 (3, 5)	4.10	4 (3, 5)	4.25	4 (4, 5)	0.001
Mental health	4.05	4 (3, 5)	4.00	4 (3, 5)	4.24	5 (4, 5)	0.51
Summary T-score^3^	41.0	41.7 (37.9, 45.4)	42.3	42. 9 (38.8, 45.7)	43.2	43.9 (38.8, 47.5)	0.004

^1^PROs based on the PROMIS Global Health Assessment taken at 6 ± 3 months after the start of adalimumab continuation, tapering, or stopping without taper (groups not mutually exclusive)

^2^ *P*-value from Wilcoxon rank sum testing comparing deprescribing groups (stop without taper or taper) vs. continue group

^3^Standardized summary instrument scores with general population mean of 50 and standard deviation of 10

## A58 Diagnosis, treatment, and functional outcomes for two adolescent female patients with lupus myelitis

### Cristina Saez^1^, Deanna Claus^1^, Andrew McCoy^2^, Gabriel Tarshish^1^, Cristina Sarmiento^1^

#### ^1^University of Colorado, Aurora, CO, USA; ^2^Children’s Hospital of Philadelphia, Philadelphia, PA, USA

##### **Correspondence:** Cristina Saez

*Pediatric Rheumatology 2024*, **22(S1):**A58

Background : Transverse myelitis is a rare neurologic complication associated with systemic lupus erythematosus (SLE) known as lupus myelitis. Little is known about the optimal treatment regimen for the disease or the functional outcomes after diagnosis, especially for pediatric patients.

Methods : This retrospective case series aimed to describe the clinical presentation, diagnostic approach, early treatment, and functional outcomes in two adolescent female patients diagnosed with lupus myelitis as a presenting sign of new-onset SLE seen at an academic pediatric tertiary care center.

Results : Both patients presented with features (Table 1) that were initially mistaken for other neurologic conditions. The combination of longitudinally extensive lesions of the spinal cord on neuroimaging and laboratory findings suggestive of an autoimmune process ultimately led to the diagnoses of lupus myelitis and new-onset SLE. Both patients received intravenous and oral corticosteroids, plasmapheresis, rituximab, cyclophosphamide, intravenous immunoglobulin, and acute intensive rehabilitation including physical therapy, occupational therapy, and speech therapy. Both patients demonstrated marked functional improvement in domains of self-care and mobility in the setting of acute inpatient rehabilitation after they began treatment for lupus myelitis.

Conclusions : Early diagnosis, treatment, and acute inpatient rehabilitation led to significant improvement in functional outcomes for the two pediatric patients in this study, and interdisciplinary collaboration allowed for a coordinated approach to achieve medical and functional goals.

IRB Statement : Project was reviewed by Colorado Multiple Institutional Review Board, part of the University of Colorado, and deemed to be exempt from IRB review. Date effective was March 10 2023 and no expiration date was given. Submission ID APP001-1

**Table 1 (Abstract A58):** Characteristics of patients (*N*=2) and acute care management. Presenting symptoms, laboratory abnormalities, imaging findings and initial treatment of both patients presented in the series

	Patient 1	Patient 2
**Demographic Characteristics**		
Age (years)	12	15
Sex (assigned at birth)	Female	Female
Past medical history	None	Aseptic meningitis at age 9, Depression
Prodromal signs and symptoms	Preceding upper respiratory illness, fatigue, musculoskeletal pain	Headache, neck stiffness, fever, rash
**Acute Care Management**		
Total length of acute care admission	24 days	17 days
Clinical findings on presentation	Urinary retention, lower extremity weakness, areflexia	Lower extremity weakness, areflexia, bowel and bladder incontinence
MRI findings	T2 hyperintensity nearly the entire length of spinal cord, greatest in central gray matter, including non- enhancing T2 hyperintensity in the upper cervical spinal cord extending into the cervicomedullary junction	Scattered T2 signal abnormalities throughout, extending from T1 to conus medullaris; faint cranial nerve (V, VI, VII) and increased sulcal enhancement in brain
Cerebrospinal fluid findings	WBC count: 93 cells/mm^3^ Glucose: 22 mg/dL Protein: >200 mg/dL	WBC count: 119 cells/mm^3^ Glucose: 39 mg/ dL Protein: 49 mg/ dL
Notable serologic findings	+ ANA + Anti-dsDNA Ab + Anti-RNP Ab + Anti-Smith Ab + Beta 2 glycoprotein immunoglobulin G Ab Low C3, low C4 Elevated ESR Pancytopenia	+ ANA + Anti-dsDNA Ab + Anti-RNP Ab + SSA Ab + Ribosomal P Ab + Coombs Low C3, low C4
Acute immunomodulatory treatment	IV methylprednisolone 1000 mg (HD # 8, 9, 10) IVIG 0.765 g/kg (HD # 4, 5, 6) Cyclophosphamide 500 mg (HD # 10, 23, 37, 50, 71) Rituximab induction 1000 mg (HD # 9, 24) Plasmapheresis (HD # 13, 15, 17, 22)	Corticosteroids IV 1000 mg (HD # 3, 4, 5, 6, 7) IVIG 1 g/kg (HD # 64,65) Cyclophosphamide 1,200 mg (750/m2) (HD # 12, 41, 66, 82, 123, 151) Rituximab induction 1000 mg (HD # 14, 27) Plasmapheresis (HD # 4, 5, 7, 9, 11)

## A59 Exploring depressive symptoms as a mediator of change in fatigue in individuals with childhood-onset lupus

### Beyan Sannah^1^, Michelle Adler^1^, Khalid Abulaban^2^, Elizabeth Kessler^2^, Andrea Knight^3^, Natoshia Cunningham^1^

#### ^1^Michigan State University, Grand Rapids, Michigan, USA; ^2^Helen DeVos Children’s Hospital; ^3^The Hospital for Sick Children and SickKids Research Institute, Toronto, Ontario, Canada

##### **Correspondence:** Michelle Adler

*Pediatric Rheumatology 2024*, **22(S1):**A59

Background : Fatigue and depressive symptoms are common in individuals with childhood-onset lupus are may adversely impact health-related quality of life. These symptoms are potentially modifiable over time. In our primary RCT study, we found both depression and fatigue may be responsive to cognitive behavioral therapy called TEACH for individuals with childhood-onset lupus. It is therefore important to understand potential mechanisms of change. Although it has not yet been studied in lupus, research in other disease populations such as multiple sclerosis, juvenile fibromyalgia, and cancer, has found that depression mediates the changes in fatigue following cognitive behavioral therapy. The current study examines whether depressive symptoms mediate changes in fatigue in adolescents and young adults with childhood-onset lupus.

Methods : A total of 59 individuals, 2 males and 57 females, between the ages of 12 and 22 years who enrolled in the primary trial (comparing TEACH to standard care) were included in the current study. Measures of fatigue and depressive symptoms were collected at three time points: Baseline (Time 1; T1), Mid-treatment/Waitlist (4 weeks after baseline; Time 2; T2), and Post Assessment (8 weeks after baseline, Time 3; T3). The measure used for assessing fatigue was the PROMIS-Fatigue Measure and for depressive symptoms was the Children’s Depressive Inventory, second edition (CDI-2) for those under age 18, and the Beck Depression Inventory (BDI), for those 18 years of age or older. All measures were scored using a T score. Means, ranges, and standard deviations of each measure were calculated and bivariate correlations were conducted. Next, we used general linear models and Sobel testing to conduct the mediation analysis. We examined the association between T1 fatigue and T2 depressive symptoms, the association between T1 and T3 fatigue, and the association between T2 depressive symptoms and T3 fatigue. Sobel testing was used to examine the significance of the mediation model and the variance of indirect effects.

Results : Table 1 reports study descriptives and correlations. Age was not correlated with any of the variables. In summary, the sample was categorized by moderate levels of fatigue and depressive symptoms. Visual inspection suggests most symptom changes occurred between Time 1 and Time 2. T1 fatigue and T2 depressive symptoms were correlated with T3 fatigue. However, T1 fatigue was not correlated with T2 depressive symptoms. General linear models revealed similar results (See Table 2). Overall, T2 depressive symptoms did not mediate the association between fatigue at baseline and post-assessment (Sobel = 1.27, *p* = .203), with T2 depressive symptoms accounting for only 7.5% of the variance of indirect effects

Conclusions : Depressive symptoms did not mediate the association between fatigue at baseline and post-assessment. Future analysis to look at differences in CBT completers versus controls will be important in addition to looking at other factors (e.g., sociodemographic variables) in relationship to changes in these symptoms.

IRB Statement : This submission has been approved by the Michigan State University (MSU) Biomedical and Health Inst. Review Board (BIRB). The submission was reviewed by the Institutional Review Board (IRB) through the Committee Review procedure. The IRB has found that this study protects the rights and welfare of human subjects and meets the requirements of MSU’s Federal Wide Assurance (FWA00004556) and the federal regulations for the protection of human subjects in research (e.g., 2018 45 CFR 46, 21 CFR 50, 56, other applicable regulations).

Acknowledgements : The authors wish to acknowledge CARRA and the ongoing Arthritis Foundation financial support of CARRA. This project was funded by a CARRA-Arthritis Foundation Transdisciplinary research grant.

**Table 1 (Abstract A59):** Correlations between study variables

	Min	Max	Mean	SD	Fatigue-T1	Fatigue-T2	Fatigue-T3	Mood-T1	Mood-T2	Mood-T3
Age	12	20	16.71	2.04	0.019	0.163	0.017	0.144	0.161	0.055
Fatigue-T1	40.90	78.50	61.09	6.99	--	0.588*	0.387*	0.380*	0.188	0.61
Fatigue-T2	34.30	77.80	55.63	7.72	0.588*	--	0.556*	0.331*	0.428*	0.159
Fatigue-T3	38.50	73.20	55.51	7.399	0.387*	0.556*	--	0.291*	0.441*	0.537*
Mood-T1	38.70	85.00	64.31	10.88	0.380*	0.331*	0.291*	--	0.637*	0.510*
Mood-T2	37.00	83.00	85.06	11.29	0.188	0.428*	0.441*	0.637*	--	0.761*
Mood-T3	37.00	89.60	56.88	11.62	0.61	0.159	0.537*	0.510*	0.761*	--

*Significant *p*-value <0.05

**Table 2 (Abstract A59):** Mediation Testing for Total (*N*=59) Sample

Model	Predictor	Variable	***R*** ^**2**^	***F***	***β***	***p***
1	Fatigue-T1	Fatigue-T3	0.150	10.039	0.387	0.002
2	Fatigue-T1	Mood-T2	0.035	1.871	0.188	0.177
3	Fatigue-T1	Fatigue-T3	0.315	11.427	0.353	0.005
	Mood-T2				0.374	0.003

## A60 Metabolomic profiling of plasma from children with juvenile idiopathic arthritis

### Colleen Correll, Kevin Murray, Rick Jansen

#### University of Minnesota

##### **Correspondence:** Colleen Correll

*Pediatric Rheumatology 2024*, **22(S1):**A60

Background : The etiology of juvenile idiopathic arthritis (JIA) remains unclear. Metabolomics research allows for the identification and quantification of thousands of metabolites and can lead to new insights into disease pathogenesis. We are presenting preliminary data from a discovery metabolomics study to assess unique metabolic profiles in children with JIA compared to unaffected controls and are presenting preliminary data here.

Methods : We compared plasma samples from 69 children with oligoarticular and rheumatoid factor (RF)-negative polyarticular JIA who were enrolled in the Juvenile Arthritis in Minnesota (JAMinn) study. Metabolic profiling was performed using the commercially available Biocrates’ MxP ® Quant 500 kit. All metabolite assays were performed on an Agilent 6495C triple quadrupole platform coupled with an Agilent Infinity II liquid chromatography system (Agilent, Santa Clara, CA, USA). Small molecule metabolites were measured by liquid chromatography-tandem mass spectrometry (LC-MS/MS). Lipids and hexoses were measured by flow injection analysis-tandem mass spectrometry (FIA- MS/MS). Analytical measurement was performed using multiple reaction monitoring approach. The raw data files were processed using Biocrates’ WebIDQ™ software to estimate metabolite concentrations. Concentration between plates were normalized using the pooled quality control sample analyzed on each plate in replicate. We used random forest to classify the most important metabolites by class and present class abundance across samples. Statistical significance cutoff is set at 0.05 with adjustment for multiple testing.

Results : Figure 1 shows the most important metabolite in the data differentiating cases from controls. Only one metabolite, phosphatidylcholine (PC) ae C30:2, was statistically significantly different in cases compared to controls (Figure 2). It was measured at a lower average among cases compared with controls. Figure 3 is a heat map demonstrating metabolite class patterns between cases and controls. No clear pattern differences were observed.

Conclusions : In this preliminary data comparing the metabolomic profiles in plasma from children with oligoarticular and RF-negative polyarticular JIA compared to controls, the only metabolite found to be statistically significantly different between cases and controls was PC ae C30:2, which was found to be lower in cases compared to controls, however of note, the log2 fold change is very small. PC is a phospholipid that has previously been shown to be lower in other autoimmune diseases including inflammatory bowel disease. The clinical significance of relatively low PC in patients with JIA in this study is unclear and further analysis is in progress.

IRB Statement : This study was approved by the University of Minnesota IRB. STUDY00001169

Acknowledgements : Thank you to the Rheumatology Research Foundation for supporting this work through the Investigator Award.

**Fig. 1 (Abstract A60):**
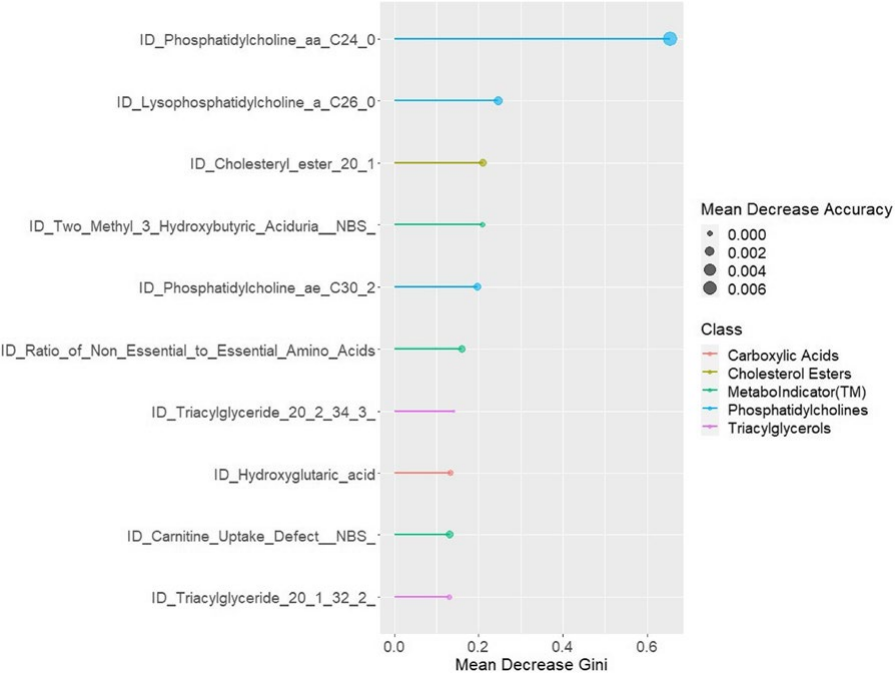
Random forest plot of metabolites classification between cases and controls. The larger the point at the end of the line and the longer the line the more important the variable is to classification. We can see which classes of metabolites appear in the top 10 most often by color key

**Fig. 2 (Abstract A60):**
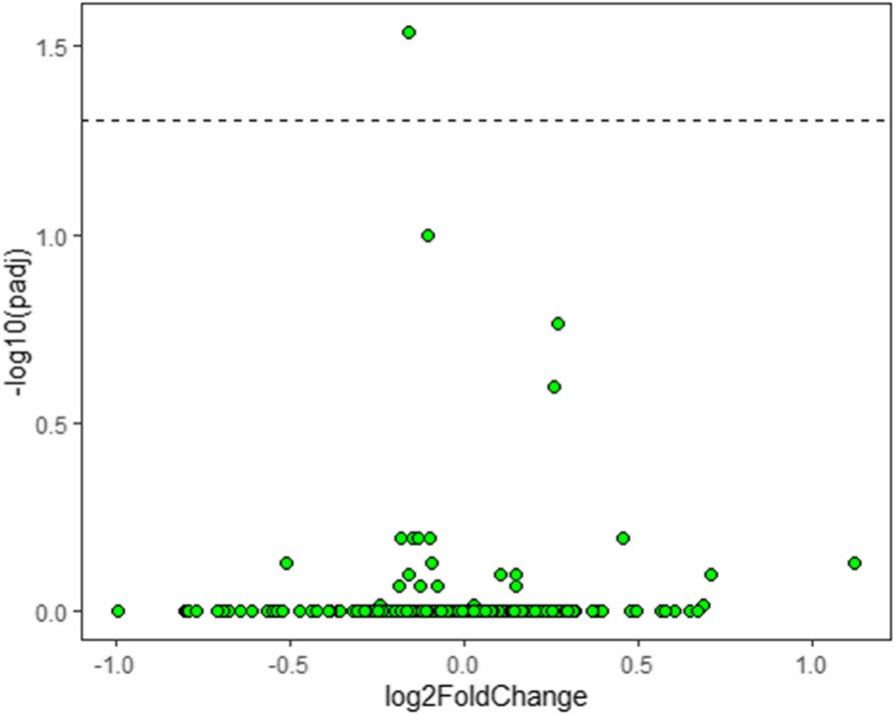
Volcano plot showing statistically significant results above the dashed line. The only metabolite seen above the dashed line is phosphatidylcholine ae C30:2. The log fold change is small and further evaluation is needed to determine the clinical significance

**Fig. 3 (Abstract A60):**
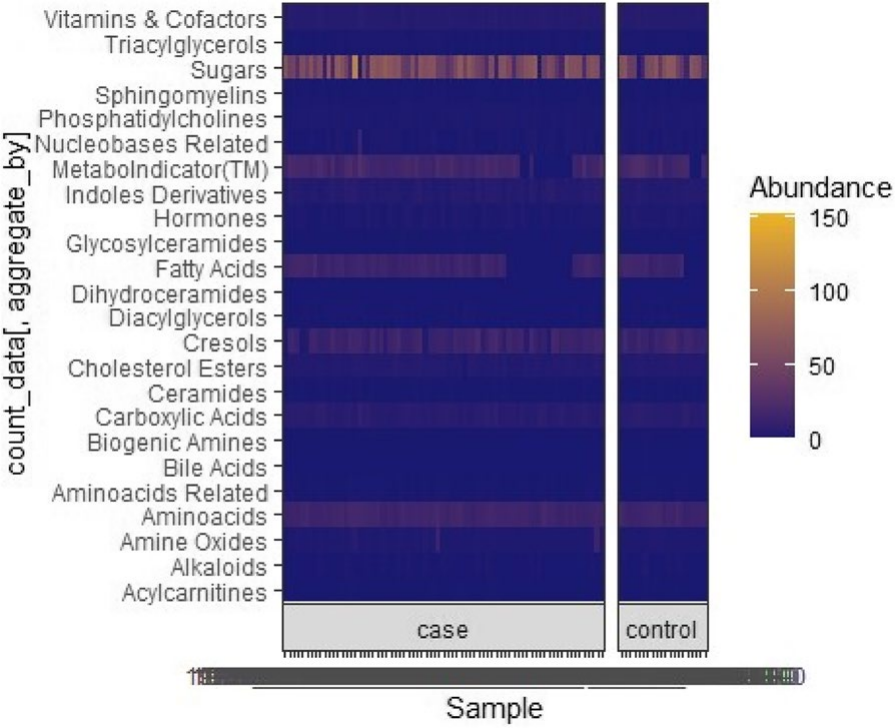
Heatmap demonstrating differences in metabolites groups between cases and controls. There was no clear pattern differences observed between metabolite classes across the two groups

## A61 CD14+ monocytes and CD8+ T cells demonstrate unique transcriptional signatures in macrophage activation syndrome, highlighting roles for interferons in monocytes and activation or exhaustion of cytotoxic T cells

### Susan Canny^1^, Hannah DeBerg^2^, Griffin Gessay^2^, Ailing Lu^3^, Mary Eckert^4^, Andrea La Bella^5^, Susan Shenoi^4^, Joyce Hui-Yuen^3^, Betsy Barnes^3^, Jessica Hamerman^2^

#### ^1^University of Washington, Seattle, WA, USA; ^2^Benaroya Research Institute; ^3^Feinstein Institutes for Medical Research; ^4^Seattle Children’s Hospital, Seattle, WA, USA; ^5^Northwell Health

##### **Correspondence:** Susan Canny

*Pediatric Rheumatology 2024*, **22(S1):**A61

Background : Macrophage activation syndrome (MAS) is a potentially fatal complication of rheumatic diseases. MAS is characterized by a dysfunctional hyperinflammatory response in which there is abnormal activation of lymphocytes and phagocytes, leading to an overproduction of inflammatory cytokines and damage to host tissues. Circulating monocytes are highly responsive to their surrounding environment, are known to exhibit phenotypic and functional changes during inflammation, and can give rise to macrophages that phagocytose immune cells. However, monocytes and macrophages have not been well-studied in MAS. CD8+ T cells play a critical role during MAS by driving myeloid cell activation through secretion of the cytokine, interferon gamma (IFNg). MAS is most commonly associated with systemic juvenile idiopathic arthritis (sJIA). At least 10% of sJIA patients will experience an overt episode of MAS with up to 50% exhibiting signs of subclinical inflammation.

Methods : We analyzed classical CD14+ monocytes from children with active MAS (6 subjects) compared to individuals with sJIA without MAS (4 subjects) and age/sex/race-matched healthy children (8 subjects) by flow cytometry and RNA sequencing (RNA-Seq). We analyzed monocytes and lymphocytes by single cell RNA sequencing (scRNA-Seq). Monocytes from eight MAS subjects and four matched healthy subjects were analyzed by scRNA-Seq; lymphocytes from six MAS subjects and two healthy subjects were analyzed by scRNA-Seq. Subjects with MAS were defined based on the 2016 classification criteria by Ravelli and colleagues as well as the ratio of ferritin to ESR.

Results : We found significant upregulation of CD16 surface expression during active MAS using flow cytometry (*n*=4-8 per group, *p* < 0.01). Our bulk RNA-Seq data show broad transcriptional changes in CD14+ monocytes from children with active MAS, including upregulation of RNase 2 (involved in processing RNAs for the innate immune sensor TLR8) and SLAMF7 (associated with monocyte/macrophage hyperinflammation in response to interferon gamma). scRNA-Seq analyses of myeloid cells from subjects with active MAS revealed a strong interferon signature in MAS monocytes with enrichment of interferon stimulated genes and alarmins. The MAS transcriptional signature identified by bulk RNA sequencing was enriched in all monocyte subpopulations. scRNA-Seq analyses of lymphocytes from subjects with active MAS revealed expansion of a CD8+ T cell subpopulation expressing markers of activation and exhaustion including LAG3, CTLA-4, and PD-1 as well as CD38 and HLA-DR. These T cells were also enriched for expression of IFNg, perforin, and granzyme B.

Conclusions : These data confirm an important role for cytokines, specifically interferons, in driving gene expression in monocytes during MAS and demonstrate expansion of a specific subpopulation of CD8+ T cells. Together, our data show that CD14+ monocytes and CD8+ T cells have unique transcriptional signatures during active MAS.

IRB Statement : The study was approved by the Institutional Review Boards of Seattle Children’s Hospital, Benaroya Research Institute, and Cohen Children’s Medical Center.

Acknowledgements : The authors gratefully acknowledge the participation of the research subjects and their families. This work was funded by: NIH (R01 AR076242 to JH and BB; T32 AR007108; T32 HD007233; and F32 HL156516 to SPC), the Arthritis National Research Foundation (to SPC), and a CARRA/Arthritis Foundation Career Development Award (to SPC). The authors wish to acknowledge CARRA and the ongoing Arthritis Foundation financial support of CARRA.

## A62 Adolescents’ perception of the transition from pediatric to adult rheumatology

### Julia Witowska, Brett Curtis, Melanie Donahue, Sara Platte, Rebecca Northway, Jacqueline Madison

#### University of Michigan, Ann Arbor, MI, USA

##### **Correspondence:** Julia Witowska

*Pediatric Rheumatology 2024*, **22(S1):**A62

Background : The majority of adolescents with rheumatic conditions now survive into adulthood with continued disease activity. These adolescents require adequate preparation as they move from pediatric to adult rheumatology to facilitate medical independence and maintain control of their condition. The aim of this study was to evaluate the current transition of care practices and experiences of adolescents with pediatric onset rheumatologic conditions.

Methods : A survey was sent to adolescents aged 18-27 who have transitioned from pediatric to adult rheumatology between 2020-2023 at a single academic medical center. The 32-question survey evaluated adolescents’ experiences and perception of various aspects of the transition process.

Results : The survey was completed by 25 patients with a response rate of 10%. The majority indicated that the transition to an adult model of care was not discussed until the age of 18-20 (56%), the same age that transition was completed (60%). Only 44% felt that they had enough time to discuss the transition with 64% feeling nervous or worried about transitioning to adult care (Figure 1). Most adolescents did not participate in a formalized transition program (88%) but reported that their disease was under good control at time of transition (68%). Less than half of patients had conversations with their providers regarding the differences between pediatric and adult care (32%) or long-term effects of their condition into adulthood (44%). Factors that were reported to have the greatest effect on the success of transition were stability and knowledge of disease, age at transition, and ability to manage medications (Figure 2). Adolescents were most concerned about transition preparedness, relationship with their new provider, and systemic factors affecting their transition (Table 1).

Conclusions : Transitioning adolescents with rheumatic disease report adequate disease control at the time of transition, but multiple deficiencies persist in preparing to transition to an adult model of care. Our study uniquely highlights the need to discuss different expectations between adult and pediatric care. We also found an opportunity to improve counseling by educating patients about their prognosis prior to transition. Efforts should be made to develop structured transition programs with earlier engagement regarding transfer from pediatric to adult rheumatology.

IRB Statement : This study was reviewed and determined to be exempt from ongoing review by the University of Michigan Medical School Institutional Review Board (IRBMED), study number HUM00235097.

**Fig. 1 (Abstract A62):**
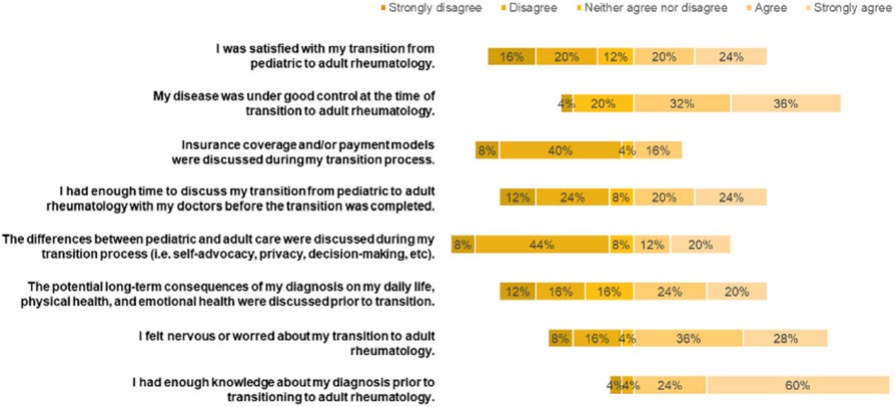
Adolescents’ experiences with the transition from pediatric to adult rheumatology

**Fig. 2 (Abstract A62):**
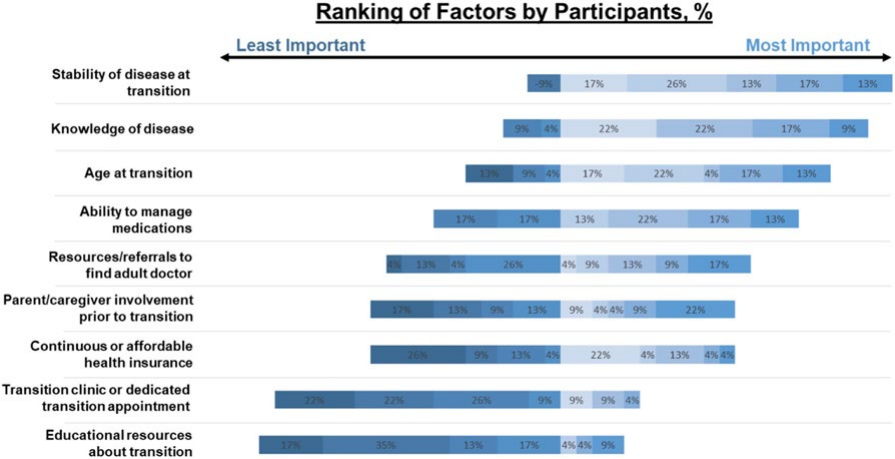
Factors affecting the transition from pediatric to adult rheumatology

**Table 1 (Abstract A62):** Adolescents’ concerns regarding current transition practices

Theme	Representative Quotes
Transition preparedness	“I think the transition should be slowed down, with the adult rheumatologist being assigned to the patient before their last appointment with pediatrics”
	“Letting patients know from the beginning when they will be expected to transition…before their last appointment with the pediatric specialist”
	“My transition was discussed during the last day of seeing my pediatric rheumatologist. I had no time to process my transition”
Relationship with providers	“The process itself should include going from the pediatric doctor to the same adult doctor if possible. It would make it easier on the young adult because they are comfortable with that doctor”
	“I feel like I knew my pediatric doctor really well and felt comfortable with her. It seemed like adult rheumatology felt less comfortable and inviting as well as hard to communicate with and connect with the new doctors”
	“The number one thing that my pediatric doctor told me was that adult rheumatology was not going to care as much as pediatric doctors will. He just wanted me to be prepared and to take control of my disease and be vocal about how I am feeling and what I need”
	“From personal experience, it is difficult to be the youngest patient your adult rheumatologist treats… I have JIA, a primary pediatric illness. Does my illness have a place in adult rheumatology?”
System factors	“Offer all patients transitioning to adult rheumatology the opportunity to have a transition clinic”
	“My experience was smooth, but had I not been referred and kept in system, it would have been different”
	“Pediatric facilities are bright and inviting. Adult facilities are dull colored and not as welcoming. If adult facilities were to change that, it would help with the transition process”
	“I had to facilitate the transfer myself, decide who I wanted to see as a provider, and request a referral to be sent for the physician. I think this should be a more structured process”

## A63 Increased levels of psychological distress, depression and anxiety symptoms in children with pediatric rheumatologic diseases

### Erin Treemarcki^1^, Natalie Rosenwasser^2^, Tamar Rubinstein^3^, Natoshia Cunningham^4^, Aimee Hersh^1^, Andrea Knight^5^

#### ^1^University of Utah, Salt Lake City, UT, USA; ^2^Seattle Children’s Hospital, Seattle, WA, USA; ^3^Albert Einstein College of Medicine, Bronx, NY; Children’s Hospital at Montefiore, Bronx, NY, USA; ^4^Michigan State University, Grand Rapids, Michigan, USA; ^5^The Hospital for Sick Children and SickKids Research Institute, Toronto, Ontario, Canada

##### **Correspondence:** Erin Treemarcki

*Pediatric Rheumatology 2024*, **22(S1):**A63

Background : Mental health problems are common in children with pediatric rheumatologic diseases (PRDs) and are associated with worsened quality of life and poorer disease-related outcomes. Psychological distress results from exposure to stress, may be exacerbated in response to traumatic events (e.g., COVID-19 pandemic), and can lead to significant mental health problems. We aimed to determine the rates of psychological stress in PRDs and compare to other measures of psychosocial functioning including anxiety, depressive symptoms, and COVID-related distress.

Methods : Eligible patients in this ongoing 3 center cross-sectional study are between 8-17 years and enrolled in the CARRA Registry with a diagnosis of JIA, jSLE, or JDM. Consented participants completed a one-time survey during a scheduled rheumatology visit, including Patient-Reported Outcomes Measurement Information System® (PROMIS) measures for psychological stress, physical stress, and depressive symptoms, in addition to the NIH-Toolbox Perceived Stress survey, clinically validated measures of depression (PHQ-9) and anxiety (SCARED), a visual analog scale for COVID-related distress, and a questionnaire to assess acceptability of mental health screening. Descriptive statistics were used for patient characteristics. The proportion of patients with a positive screen based on clinical cutoffs was determined for each measure. For the PROMIS and NIH-Toolbox measures, 1 standard deviation above the mean of the reference population (T-score 50) indicated high levels of that measure. The relationship between psychological distress and other measures was determined by Pearson Correlation Coefficient.

Results : The 150 patients who completed the survey had a mean age of 13.5 years (SD=2.7) and 91% had a diagnosis of JIA (Table 1). Psychological stress experiences were elevated in 34% and physical stress experiences in 41% (Table 2). High levels of perceived stress were reported in 20% of patients aged 13-17 years and 16% of those aged 8-12 years. While increased depressive symptoms were seen in only 24% on the PROMIS measure, 51% of patients had a positive PHQ-9 screen. Almost half the cohort (45%) had SCARED scores concerning for anxiety disorder. Most patients endorsed mild distress from the COVID-19 pandemic (median 2, IQR 0,5); only 5 (3.5%) endorsed severe distress. Psychological stress was highly correlated with physical stress, perceived stress, depressive symptoms (PROMIS and PHQ-9), and anxiety (Table 3); however, correlation with physical stress was not statistically significant. Half of those surveyed felt ready to discuss mental health with their rheumatology provider (49.7%) and moderately confident in discussing mental health concerns (51.7%).

Conclusions : Children with PRDs experience greater psychological distress that is related to physical stress, perceived stress, depressive symptoms, and anxiety. Patients want to and feel confident discussing mental health concerns with their rheumatology providers. Next steps include expanding this cohort to include increased patients with JDM and jSLE and determining the relationship between mental health symptoms and health-related outcomes including disease activity measures.

IRB Statement : This study was approved by the Institutional Review Board of Primary Children’s Hospital and the University of Utah.

Acknowledgements : This study was funded by a CARRA-Arthritis Foundation large grant. This work could not have been accomplished without the aid of the following organizations: The NIH’s National Institute of Arthritis and Musculoskeletal and Skin Diseases (NIAMS) & the Arthritis Foundation. We would also like to thank all participants and hospital sites that recruited patients for the CARRA Registry. The authors thank the following CARRA Registry site principal investigators: K. Abulaban, C. Aguiar Lapsia, S. Ardoin, L. Barillas-Arias, M. Basiaga, K. Baszis, H. Brunner, H. Bukulmez, E. Chalom, J. Chang, D. Co, K. Cook, A. Cooper, C. Correll, T. Davis, F. Dedeoglu, M. DeGuzman, A. Dhanrajani, K. Ede, B. Edelheit, B. Feldman, I. Ferguson, D. Glaser, D. Goldsmith, B. Gottlieb, T. Graham, T. Griffin, T. Hahn, L. Harel, O. Harry, M. Hollander, S. Hong, M. Horwitz, J. Hsu, A. Huber, L. Imundo, C. Inman, P. Kahn, S. Kim, D. Kingsbury, M. Klein-Gitelman, L. Lim, M. Mannion, D. McCurdy, D. Milojevic, S. Mohan, T. Moore, K. Moore, L. Moorthy, S. Nativ, M. Natter, K. Onel, J. Patel, S. Prahalad, C. Rabinovich, A. Robinson, T. Ronis, M. Rosenkranz, N. Ruth, S. Sabbagh, K. Schikler, C. Schutt, E. Sloan, J. Spitznagle, Y. Sterba Rakovchik, K. Stewart, G. Syverson, S. Tarvin, M. Tesher, D. Toib, M. Toth, M. Twilt, H. Van Mater, D. Wahezi, P. Weiss, J. Weiss, L. Woolnough, E. Wu, A. Yalcindag, Y. Zhao

**Table 1 (Abstract A63):** Demographic information

Patient Characteristics	
Age, Years (Mean, SD)	13.5 (2.7)
Sex (N, %)	
Female	98 (68.1)
Male	46 (32.0)
Diagnosis (N, %)	
JDM	3 (2.7)
JIA	136 (90.7)
SLE	10 (6.7)

**Table 2 (Abstract A63):** Patient-reported outcomes

	Complete (N)	Mean T-Score (SD)	Score >60 (N, %)	Mean Score (IQR)	Positive Screen (N, %)
Psychological Stress Experiences	150	57.28 (9.21)	51 (34.0)		
Physical Stress	148	57.84 (10.01)	61 (41.2)		
Perceived Stress					
Age 13-17 (Self)	97	51.01 (10.77)	19 (19.6)		
Age 8-12 (Parent)	49	48.62 (12.22)	8 (16.3)		
Depressive Symptoms	146	52.99 (10.91)	35 (24.0)		
PHQ-9 (Age 11-17)	116			6.68 (2.00, 11.00)	Mild: 25 (21.6) Moderate: 17 (14.7) Moderately Severe: 11 (9.5) Severe: 6 (5.2)
SCARED	143			24.71 (12.00, 36.50)	Score 25+: 65 (45.5) Score 30+: 54 (37.8)

For the PROMIS/NIH measures, T-score for the reference population is 50 and the standard deviation is 10. One standard deviation above the reference population indicates more of the measure. For the measures above, this would indicate increased mental health symptoms

**Table 3 (Abstract A63):** Relationship between psychological stress experiences and other measures of psychosocial functioning

Measure	Pearson Correlation Coefficient (P)
Physical Stress	0.739 (0.14)
Perceived Stress	
Age 13-17 (Self)	0.872 (<0.05)
Age 8-12 (Parent)	0.682 (<0.05)
Depressive Symptoms	0.858 (<0.05)
PHQ-9 (Age 11-17)	0.801 (<0.05)
SCARED	0.702 (<0.05)
COVID VAS	0.437 (<0.05)

## A64 Effects of sex and site of origin on osteoclast formation and activity

### Michael Christof^1^, Kiana Chen^2^, Xi Lin^2^, Lianping Xing^2^, Homaira Rahimi^2^

#### ^1^University of Rochester, Rochester, NY, USA; ^2^University of Rochester Medical Center, Rochester, NY, USA

##### **Correspondence:** Michael Christof

*Pediatric Rheumatology 2024*, **22(S1):**A64

Background : Rheumatoid arthritis (RA) is a chronic inflammatory autoimmune disorder characterized by synovial inflammation and bone erosions. We study inflammatory erosive arthritis using the TNF-transgenic (TNF-Tg) murine model of RA. TNF-Tg mice have sexual dimorphism in their disease, with female mice having worse disease than males. We aimed to determine how sex and location of osteoclast precursors (OCPs) affect osteoclast (OC) growth and quantity, and if sex also affects osteoclast bone resorptive activity. We hypothesize that more osteoclasts will differentiate from bone marrow than peripheral blood and from female than male TNF-Tg mice, as well as greater bone resorption from female-derived, TNF-Tg OCs.

Methods : Bone marrow (BM) and peripheral blood mononuclear cells (PBMC) were harvested from 3-month-old female and male TNF-Tg mice (*n*=3 mice/group). Cells were cultured in OC differentiation media with 30ng/mL of Macrophage Colony-Stimulating Factor (M-CSF) and 30 ng/mL Receptor Activator of Nuclear Factor Kappa-Β Ligand (RANK-L). After OC formation, cells were fixed and stained for tartrate-resistant acid phosphatase (TRAP). OCs were defined as cells with >3 nuclei and TRAP+. BM cells were also harvested from 3-month-old TNF-Tg and WT male and female mice and grown on bovine bone slices with 100ng/mL of M-CSF and 100ng/mL RANK-L. Bone slices were fixed and stained with TRAP and toluidine blue after 10 days of culture. ImageJ was used to analyze the bone pit area. Unpaired t-test, two-way ANOVA, and one-way ANOVA with Tukey’s post-test for multiple comparisons were used for analysis. Values are reported as mean +/- standard deviation.

Results : BM cells formed significantly more OCs than PBMC (392.6 +/- 83.65 vs 200.5 +/-87.28) from the same TNF mice (Fig. 1E). OCs formed from PBMCs were larger than those derived from BMs, evidenced by significantly increased percentage of OCs with >10 nuclei compared with BM OCs (35.85 +/-10.73% in PBMC-OCs vs 22.5 +/- 7.83% in BM OCs) (Fig. 1F). No significant difference between sex in BM-derived OCs and PBMC-derived OCs was observed. There were more male-derived and female-derived BM OCs than female-derived PBMC OCs (399.9 +/- 47.24 and 385.3 +/- 122.9 vs 147.2 +/- 49.39) (Fig. 1G). There were also more male-derived PBMC-OCs than female-derived BM-OCs for the percentage of OCs with >10 nuclei (42.17 +/- 11.24% vs 17.27 +/- 7.48%) (Fig. 1H). Representative images of male and female TNF-Tg OCs are shown (Fig. 1A-D), as well as bone pits from TNF-Tg, male-derived BM-OCs (Fig. 2A). There was no significant difference in bone pit sizes between TNF and WT male and female mice (Fig. 2B).

Conclusions : BM-derived OCPs produce more OCs than PBMCs yet PBMC-derived OCs have >10 nucleated OCs, suggesting the origin of OCPs may affect OC activity. Bone pit sizes did not differ between sexes regardless of inflammatory environment, indicating OC activity is not affected. This finding suggests that once cells are committed to the bone resorption process, origin and sex have less effect early in the osteoclastogenesis pathway. Further studies are needed to determine whether specific sex hormones alter OC growth and activity.

IRB Statement : IACUC approval.

**Fig. 1 (Abstract A64):**
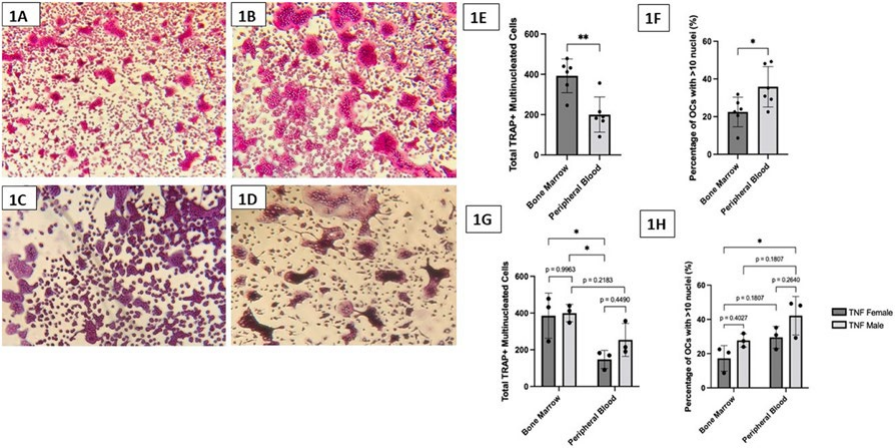
Images of PBMC and BM-derived OCPs from 3-month-old female and male TNF-Tg mice. Female PBMC (**A**) and male PBMC (**B**) and female BM (**C**) and male BM (**D**) at 10X magnification. TRAP+ cells and the percentage of cells with >10 nuclei were identified between BM and PBMC-derived OCs (**E**, **F**). BM and PBMC-derived cells from males and females were compared for total cells (**G**) and percentage of >10 nucleated OCs (H). * = *p*<0.05; ** = *p*<0.01

**Fig. 2 (Abstract A64):**
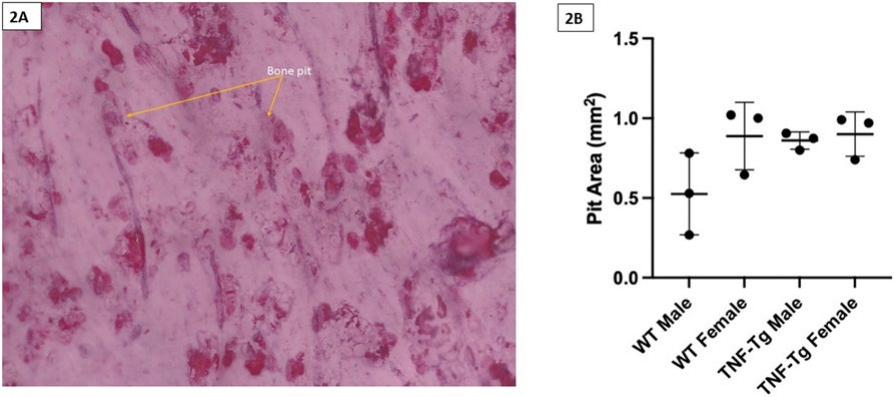
Image of stained bone pits on a bovine bone slice at 10X magnification. Arrows indicate two resorption pits on a bone slice (**A**). Pit areas on slices were compared between WT and TNF male and female-derived OCs (**B**). No difference was observed in bone resorption activity between OCs derived from male and female TNF-Tg mouse BM by resorption pit assay

## A65 Characterization of the youngest cohort with non-systemic juvenile idiopathic arthritis: demographics and medication use of patients ≤2 years of age in the childhood arthritis and rheumatology research alliance registry

### Christina Gulla^1^, Tara Lozy^2^, Daniel Choi^3^, Ginger Janow^4^, for the CARRA Registry Investigators^5^

#### ^1^Hackensack University Medical Center, Hackensack, NJ, USA; ^2^Center for Discovery and Innovation, Nutley, NJ, USA; ^3^Hackensack Meridian School of Medicine, Nutley, NJ, USA; ^4^Joseph M. Sanzari Children’s Hospital at Hackensack Meridian Health, Hackensack, NJ, USA; ^5^Childhood Arthritis and Rheumatology Research Alliance (CARRA)

##### **Correspondence:** Christina Gulla

*Pediatric Rheumatology 2024*, **22(S1):**A65

Background : Juvenile idiopathic arthritis (JIA) is the most common rheumatic disease in children. Tumor necrosis factor inhibitors (TNFi) have demonstrated efficacy and safety in older patients and are approved for use in children >age 2 years. However, use in children ≤2 is not well described despite frequent use in practice. Children ≤2 who would benefit from TNFi may have delays in treatment and increased morbidity due to the lack of FDA approval. Aim was to describe the demographics, medication use, outcomes, and adverse events in children diagnosed with non-SJIA ≤2 enrolled in the Childhood Arthritis and Rheumatology Research Alliance (CARRA) registry.

Methods : The CARRA registry was used. Demographics, clinical features, and medication use of those diagnosed with non-SJIA < 35 months (mo) were analyzed and who started a TNFi ≤35 mo, >35mo, and never used TNFi. The effectiveness cohort included those that started a TNFi < 35mo with enrollment within 6mo of diagnosis with data available for ≥2 follow ups. Descriptive statistics, ANOVA/ChiSquare tests, repeated measures ANOVA/generalized linear model and logistic regression models were used in this analysis to summarize and compare the subgroups with a significance threshold of 0.05.

Results : 1,458 patients diagnosed with non-SJIA ≤35mo were enrolled between 2015-2021. Those that started a TNFi < 35mo were younger, shorter disease duration at time of enrollment, and more likely to have poly-JIA with higher joint counts. Methotrexate was the most commonly used drug. There was an increased rate of glucocorticoid use in patients that started TNFi >35 mo. >90% that were on a TNFi were on methotrexate at some point (Table 1). TNFi use increased over time and there was a decline in all treatments 2019-2021 with corresponding decline in diagnoses (Figure 1). Those that started a TNFi < 35mo had less active joint count, less joints with decreased range of motion, less abnormal inflammatory markers, and improved disease activity scores over time (Table 2). There were few adverse events in those that started a TNFi. Odds of developing new onset uveitis was less if patients were on a TNFi at some point.

Conclusions : TNFi are an accepted treatment in early non-sJIA and are utilized often in patients < 2. TNFi use in the 1st year after diagnosis increased over time, with a drop in all treatment and number of patients enrolled during the COVID pandemic. Starting TNFi < 35 mo may result in decreased steroid use and help protect against steroid related comorbidity. TNFi are effective in this age group with improvement in joint counts, inflammatory markers, and disease activity scores. TNFi are safe with minimal adverse events and may have a protective effect for development of uveitis.

IRB Statement : Our research was done in accordance with approval from the Hackensack Meridian Health Institutional Review Board.

Acknowledgements : The authors wish to acknowledge CARRA and the ongoing Arthritis Foundation financial support of CARRA. This work could not have been accomplished without the aid of the following organizations: the NIH’s National institute of Arthritis and Musculoskeletal and Skin Diseases (NIAMS) and the Arthritis Foundation. We would also like to thank all the participants and hospital sites that recruited patients for the CARRA registry. The authors thank the following CARRA registry site PIs: K Abulaban, C Aguiar Lapsia, S Ardoin, L Barillas Arias, M Basagia, K Baszis, H Brunner, H Bukulmez, E Chalom, J Chang, D Co, K Cook, A Cooper, C Correll, T Davis, F Dedeoglu, M DeGuzman, A Dhanrajani, K Ede, B Edelheit, B Feldman, I Ferguson, D Glaser, D Goldsmith, B Gottlieb, T Graham, T Griffin, T Hahn, L Harel, O Harry, M Hollander, S Hong, M Horowitz, J Hsu, A Huber, L Imundo, C Inman, P Hahn, S Kim, D Kingsbury, M Kein Gitelman, L kin, M Prahalad, Mannion, D McCurdy, D McCurdy, D Milojevic, S Mohan, T Moore, K moore, L Moorthy, S Nativ, M Natter, K Onel, J patel, C Rabinovish, A Robinson, T Ronis, M Rosenkranz, N Ruth, S Sabbagh, K Schikler, C Schutt, E Sloan, J Spitznagle, Y Sterba Rakovchik, K Stewart, G Syverson, S Tarvin, M Tesher, D Toib, M Toth, M Twilt, H Van Mater, D Wahezi, P Weiss, J Weiss, L Woolnough, E Wu, A Yalcindag, Y Chao.

**Table 1 (Abstract A65):** Demographics, clinical features, and medication use of patients with non-systemic JIA diagnosed at ≤35 months old

	Total diagnosed with non-sJIA 0-35 months of age (***n*** = 1,458)	Started TNFi 0-35 months of age (***n*** = 259)	Did not ever start TNFi (***n*** = 569)	Started TNF after 35 months of age (***n*** = 630)	***p***-value
Age at diagnosis (month), median (IQR)	22 (19 - 26)	21 (18 - 24)	22 (19 - 26)	23 (19 - 26)	<0.01
Disease duration at time of enrollment (month), median (IQR)	43 (5 - 100)	13 (2 - 58)	19 (2 - 68)	75 (33 - 130)	<0.01
Duration of Follow Up Visits (months), median (IQR)	36 (24 - 48)	36 (18 - 48)	30 (12 - 42)	36 (24 - 48)	<0.01
Race / Ethnicity, white n (%)					
*White*	1,169 (80.2)	208 (80.3)	443 (78.0)	518 (82.2)	0.08
Gender, female n (%)					
*Female*	1,238 (84.9)	216 (83.4)	468 (82.3)	554 (87.9)	0.02
**JIA classification (n, %)**					<0.01
***Enthesitis related arthritis***	18 (1.2)	2 (0.8)	5 (0.9)	11 (1.8)	
***Oligoarthritis***	831 (57.0)	87 (33.6)	414 (72.8)	330 (52.4)	
***Polyarthritis (RF -)***	480 (32.9)	132 (51.0)	119 (20.9)	229 (36.4)	
***Polyarthritis (RF +)***	20 (1.4)	5 (1.9)	7 (1.2)	8 (1.3)	
***Psoriatic arthritis***	87 (6.0)	30 (11.6)	16 (2.8)	41 (6.5)	
***Undifferentiated arthritis***	22 (1.5)	3 (1.2)	8 (1.4)	11 (1.8)	
**Joint count, n (%)**					<0.01
***< 5***	708 (48.6)	91 (35.1)	382 (67.1)	235 (37.3)	
***≥ 5***	717 (50.6)	167 (64.5)	182 (32.0)	388 (61.6)	
**+ HLA B27, n (%)**	53 (3.6)	6 (2.3)	20 (3.5)	27 (4.3)	0.38
**+ANA, n (%)**	943 (65.2)	170 (66.2)	363 (64.3)	410 (65.6)	0.88
**+anti CCP, n (%)**	30 (2.1)	10 (3.9)	11 (2.0)	9 (1.4)	0.31
**+Rheumatoid factor, n (%)**	23 (2.1)	7 (3.3)	5 (1.2)	11 (2.3)	0.20
***Glucocorticoid***	987 (67.7)	166 (64.1)	354 (62.2)	467 (74.1)	<0.01
***Methotrexate***	1194 (81.9)	241 (93.1)	378 (66.4)	575 (91.3)	<0.01
***Other Biologic***	176 (12.1)	37 (14.3)	30 (5.3)	109 (17.3)	<0.01
***Non-biologic DMARD***	147 (10.1)	16 (6.2)	39 (6.9)	92 (14.6)	<0.01

**Fig. 1 (Abstract A65):**
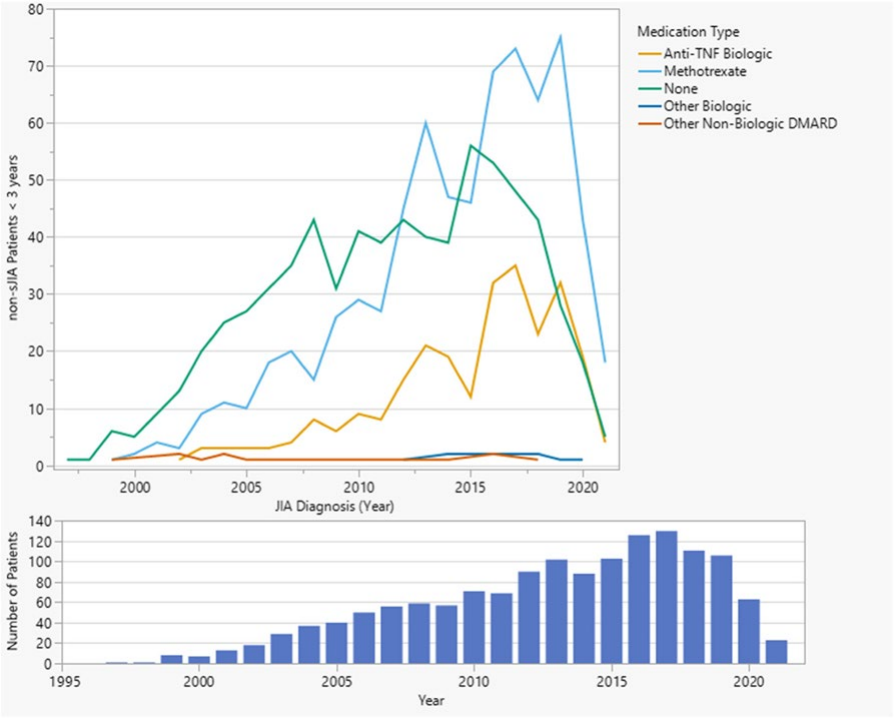
Medication Usage During 1st Year after Diagnosis of Non-sJIA Over Time

**Table 2 (Abstract A65):** Clinical outcomes for patients enrolled within 6 months of diagnosis

	TNFi started at 0-35 months			
	Within 3 mo diagnoses (***n***=82)	4-8 mo after diagnoses (***n***=89)	10-14 mo after diagnoses (***n***=70)	***p***-value
**Total number of active joints, median (IQR)**	5 (3 - 7)	1 (0 - 2)	0 (0 - 1)	<0.01
***Total***	79	87	67	
**Total # of joints with limited range of motion, median (IQR)**	3 (1 - 6)	0 (0 - 1.8)	0 (0 - 1)	<0.01
***Total***	74	84	66	
**Abnormal C-Reactive Protein value, n (%)**	28 (57.1)	4 (11.8)	2 (8.7)	0.02
***Total***	49	55	23	
**Abnormal ESR value, n (%)**	34 (66.7)	7 (18.4)	2 (8.0)	<0.01
***Total***	51	51	25	
**CHAQ disability index score, median (IQR)**	0.8 (0.3 - 1.5)	0.2 (0 - 0.8)	0.1 (0 - 0.6)	<0.01
***Total***	73	68	53	
**JADAS 10 (IQR)**	15.5 (10 - 20)	3 (1 - 7)	1 (0 - 3.4)	<0.01
***Total***	74	76	56	

## A66 Variability in vaccination practices in children with rheumatic diseases: results of a rheumatology provider childhood arthritis and rheumatology research alliance (CARRA)-wide survey

### Randal Desouza^1^, Merav Heshin Bekenstein^2^, Maria Schletzbaum^3^, Beth Rutstein^4^, Nora Singer^5^, Melanie Kohlheim^6^, Vincent Del Gaizo^6^, Kelly Wise^7^, Melica Nikahd^8^, Guy Brock^8^, Rebecca Sadun^9^, Monica Ardura^10^, Vidya Sivaraman^1^, For the CARRA Investigators^6^

#### ^1^Nationwide Children’s Hospital, Columbus, OH, USA; ^2^Tel Aviv Medical Center; ^3^Washington University School of Medicine; ^4^Children’s Hospital of Philadelphia, Philadelphia. PA, USA; ^5^Metro Health; ^6^CARRA; ^7^Nationwide Children’s Hospital, Columbus, OH, USA; ^8^The Ohio State University; ^9^Duke University School of Medicine, Durham, NC, USA; ^10^Nationwide Children’s Hospital/ The Ohio State University

##### **Correspondence:** Vidya Sivaraman

*Pediatric Rheumatology 2024*, **22(S1):**A66

Background : Immunocompromised children (ICC), including children with rheumatic diseases receiving immunosuppressive therapies (IST) are at increased risk of morbidity from vaccine-preventable infections. The 2022 American College of Rheumatology (ACR) Vaccination Guidelines emphasize the need to vaccinate ICC and minimize missed opportunities for immunizations. Children with rheumatic diseases receiving IST are eligible to receive inactivated vaccines, however vaccination rates remain unacceptably low in these children. The CARRA Vaccination Workgroup (WG) surveyed North American pediatric rheumatologists about their vaccination practices when caring for children receiving IST.

Methods : The CARRA Vaccination WG developed and electronically distributed a REDCap survey to CARRA member healthcare professionals from March-May 2022.

Results : The survey was completed by 219 pediatric rheumatology providers with a response rate of 60% (74% attendings, 21% fellows). More than 90% of rheumatology providers reviewed their patients’ vaccinations, with most reviewing live (28%) and non-live vaccines (31%) at the first visit only (Figure 1). Several providers used comments to clarify their vaccine review practices, most commonly reporting reviewing influenza and COVID vaccines alone, or in combination with other vaccines. Thirty-nine percent of providers reviewed conjugate (PCV-13) and polysaccharide (PPSV 23) pneumococcal vaccine status, especially in patients with childhood-onset SLE (c-SLE). Twenty-six percent reviewed vaccination status based on initiation or type of IST, and age, whereas half of respondents reported used a disease-specific review approach, focused primarily on c-SLE (89%), followed by systemic vasculitis (42%), juvenile dermatomyositis (39%) and juvenile idiopathic arthritis (28%). Forty-two percent of providers reported medication-specific review, including prior to Rituximab initiation (57%), initiation of any IST (20%), IVIG (10%), or cyclophosphamide (7%). There was also variability in communication about vaccines between rheumatology provider and primary care pediatricians (PCPs), with 41% of rheumatologists indicating they did not contact the PCP, 22% contacting the PCP regarding live vaccines only, 30% for live and non-live vaccines, 19% for pneumococcal vaccines, and 18% for ‘Other’, of which more than half of those who wrote narrative comments stated vaccine recommendations were included in the clinic note to the PCP.

Conclusions : This survey demonstrated significant variability in rheumatology provider approaches to vaccinations in ICC. While 90% of rheumatology providers reviewed vaccinations, less than half reviewed seasonal vaccines or pneumococcal vaccines, and those who did, mainly reviewed vaccinations in the setting of specific diseases, specific medications, or new patient visits. Our results highlight opportunities for improvement in the care of ICC by increasing awareness of the need for vaccination against vaccine-preventable infections. This study underscores the need for standardized vaccination practices in ICC and improved communication between providers to maximize immunization opportunities for ICC.

IRB Statement : The project was deemed IRB exempt.

Acknowledgements : The authors wish to acknowledge CARRA and the ongoing Arthritis Foundation financial support of CARRA

**Fig. 1 (Abstract A66):**
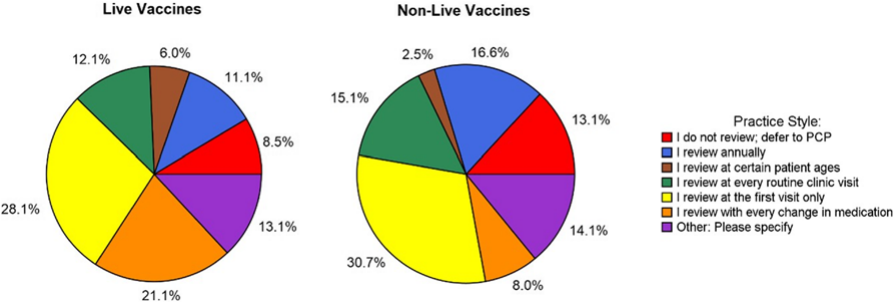
See text for description.

## A67 Preliminary findings of a community-based caregiver survey of pain, pain interference, and physical and emotional functioning in juvenile arthritis

### Daniella Schocken^1^, Kathleen Carluzzo^2^, Susmita Kashikar-Zuck^1^, Erin Knight^2^, Andrea Ring^3^, Jennifer Weiss^4^, Karen Schifferdecker^5^, for the CARRA Pain Committee^6^

#### ^1^Cincinnati Children’s Hospital Medical Center, Cincinnati, OH, USA; ^2^Center for Program Design and Evaluation, Geisel School of Medicine, Dartmouth College, Hanover, NH, USA; The Dartmouth Institute for Health Policy and Clinical Practice, Geisel School of Medicine, Dartmouth College, Hanover, NH, USA; ^3^Arthritis Foundation, Atlanta, GA, USA; ^4^Hackensack University Medical Center, Hackensack, NJ, USA; ^5^The Dartmouth Institute for Health Policy and Clinical Practice, Geisel School of Medicine, Dartmouth College, Hanover, NH, USA; ^6^Childhood Arthritis and Rheumatology Research Alliance (CARRA)

##### **Correspondence:** Daniella Schocken

*Pediatric Rheumatology 2024*, **22(S1):**A67

Background : The burden of pain and other symptoms in juvenile arthritis (JA) remains poorly understood. To better elucidate symptom severity and health-related quality of life (HRQOL) of patients with JA and their caregivers, patient-reported outcome measures (PROMs) are increasingly employed in research. This study aimed to understand pain and related aspects of health that were most important to children with arthritis through the administration of PROMs in a community-based caregiver survey.

Methods : Mixed-methods design and patient-centered approaches previously developed for evaluating HRQOL of adults with arthritis (Schifferdecker 2020) identified relevant validated PROMs, guiding their implementation and administration in the JA INSIGHTS survey. Caregivers of children with JA were invited to this non-incentivized online survey, which included the Patient Reported Outcomes Measurement Information System (PROMIS®) Pediatric Profile-25 v2.1 Parent Proxy as well as questions about their child’s arthritis diagnosis, care, and demographics. Respondents were invited to complete the survey multiple times, and between October 2020 and March 2023, 1024 surveys were completed by caregivers of 599 children with JA aged 5 to 17. This study analyzes cross-sectional data from those 599 unique participants’ most recent, most complete survey responses. Descriptive statistics were calculated for demographic data, and normalized t-scores for individual subscales were derived from responses to the PROMIS Pediatric Profile-25 for analysis.

Results : Overall, children for whom caregivers responded to the JA INSIGHTS survey were primarily female (443/599, 74%) and White (non-Hispanic) (466/599, 78%). Most respondents were under the care of a pediatric rheumatologist (567/599, 95%) and reported a diagnosis of juvenile idiopathic arthritis (JIA) (567/599, 95%). 316/599 (53%) of those surveyed had been diagnosed with arthritis in the past 5 years, with 152/599 (25%) of diagnoses occurring within the past 2 years. Average pain intensity rating (0-10, see Figure 1) reported by respondents was 3.67 (sd = 2.46). The average t-scores for pain interference and physical mobility were 57.69 (sd = 9.78) and 41.85 (sd = 9.03) respectively. Figures 2 and 3 illustrate the breakdown of respondent pain interference and physical functioning t-scores by cut-off values delineating mild, moderate, and severe levels as determined by measure validation data available from HealthMeasures.

Conclusions : Most respondents reported that their child had pain in the week preceding survey participation, with 66% of respondents indicating moderate or severe interference in daily activities due to pain and 51% of respondents indicating moderate or severe impairment in their physical functioning and mobility. Results of this survey point to a significant burden of pain and pain interference with reduced functioning in the setting of childhood arthritis. Given the demographic distribution of study participants, it will be important moving forward to look for ways to engage minority populations living with JA in future research as well as following up on these findings regarding pain and pain interference with further study.

IRB Statement : The research detailed in this abstract was subject to institutional review and approval which was obtained by the research team as appropriate.

Acknowledgements : The authors wish to acknowledge the contributions of Drs. Aimee Hersh and Laura Schanberg for their role in the Academic Advisory Committee which informed the data collection instruments, Arlene Vinci and her colleagues and volunteers at the Arthritis Foundation for their support in data collection, and the Arthritis Foundation for its generous support of this work. The authors wish to acknowledge CARRA and the ongoing Arthritis Foundation financial support of CARRA.

**Fig. 1 (Abstract A67):**
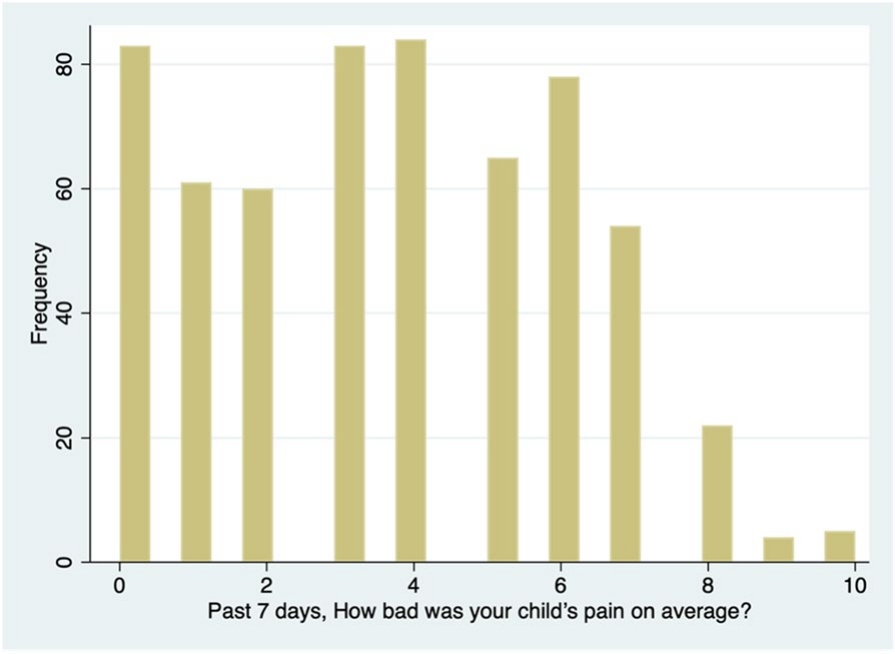
Pain Intensity Raw Score – PROMIS 25 Parent Proxy (*N*=599)

**Fig. 2 (Abstract A67):**
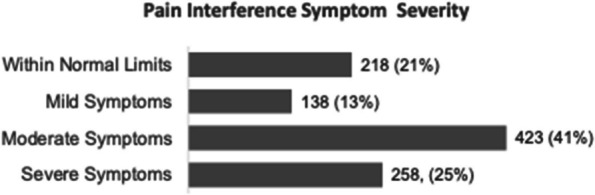
Pain Interference Symptom Severity by T-Score Cut Points – PROMIS 25 Parent Proxy (*N*=599)

**Fig. 3 (Abstract A67):**
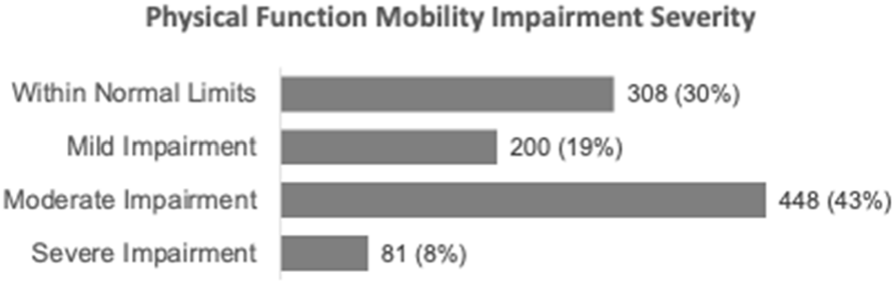
Physical Function Mobility Impairment Severity by T-Score Cut Points – PROMIS 25 Parent Proxy (*N*=599)

## A68 Sticking the landing: a 3-year qualitative longitudinal study on navigating transitions in pediatric rheumatology fellowship

### Sarah Bayefsky, Hannah Anderson, Dorene Balmer, Jay Mehta

#### Children’s Hospital of Philadelphia / University of Pennsylvania, Philadelphia, PA, USA

##### **Correspondence:** Sarah Bayefsky

*Pediatric Rheumatology 2024*, **22(S1):**A68

Background : Transitions present many challenges for medical trainees, and a lack of preparedness for these transitions is associated with negative outcomes, including higher rates of burnout (Westerman et al., 2013). Fellowship program directors are tasked with guiding trainees as they transition from residency to fellowship and from earlier to later years of fellowship. Yet little is known about what facilitates successful transitions. A longitudinal study is well-poised to explore transitions and to arm program directors with the knowledge to support fellows through these vulnerable periods.

Methods : We conducted a qualitative longitudinal study of 6 pediatric rheumatology fellows, each at a different institution in the United States that varied by size, geography, and hospital setting. Consistent with qualitative longitudinal research methodology (Balmer et al. 2021), we completed in-depth, recursive interviews of the fellows annually during their three years of training. We created inductive codes from patterns in the data and clustered codes into more abstract themes. We organized the findings into an explanatory model analogized to a Rubik’s cube, drawing from multiple and multidimensional transitions (MMT) theory (Jindal-Snape & Hannah, 2014), which posits that individuals inhabit multiple, non-static domains (for example, distinct and evolving roles at work and at home) and that a transition in one domain triggers transitions in other domains.

Results : The successful transitions of pediatric rheumatology fellows were facilitated by: (1) deconstructing old identities and constructing new ones, (2) navigating internal and external factors, and (3) acting with increasing autonomy. Fellows who were initially plagued by imposter syndrome later expressed excitement about their integration into the rheumatology community. Internal factors included personality traits; coping strategies; and self-reflection and feedback. External factors included influences from personal life, professional life, and the environment; close relationships (with patients, peers, staff, and superiors), clarity of program expectations, and quality of mentorship all play roles. For fellows, recognizing their emerging confidence, building and leading their own teams, and shifting from mentees to mentors allowed for the development of graded autonomy. Ultimately, the search for a faculty position (“job search”) in the final year of fellowship requires the assembling of new identities, navigation of internal and external factors, and autonomous actions (Figure 1).

Conclusions : Our qualitative longitudinal study closes a gap in the literature by elucidating successful transitions for pediatric rheumatology fellows. MMT theory provides a theoretical framework to explain the interplay of the many factors facilitating these transitions (Figure 2). Program directors can guide fellows through transitions and “stick the landing” in the job search by directly managing external factors related to professional life, aiding in the development of autonomy and of skills that mediate the impact of internal factors, and supporting fellows through challenges in their personal lives.

IRB Statement : This study was deemed exempt by The Children’s Hospital of Philadelphia (CHOP) IRB: “Re: IRB 18-015277: The study referenced above was reviewed by the IRB on 7/5/2018. The IRB has determined it meets the exemption criteria per 45 CFR 46.104(d) 2.”

Acknowledgements : We wish to acknowledge the Rheumatology Research Foundation, which provided funding via Dr. Jay Mehta’s Clinician Scholar Educator Award.

**Fig. 1 (Abstract A68):**
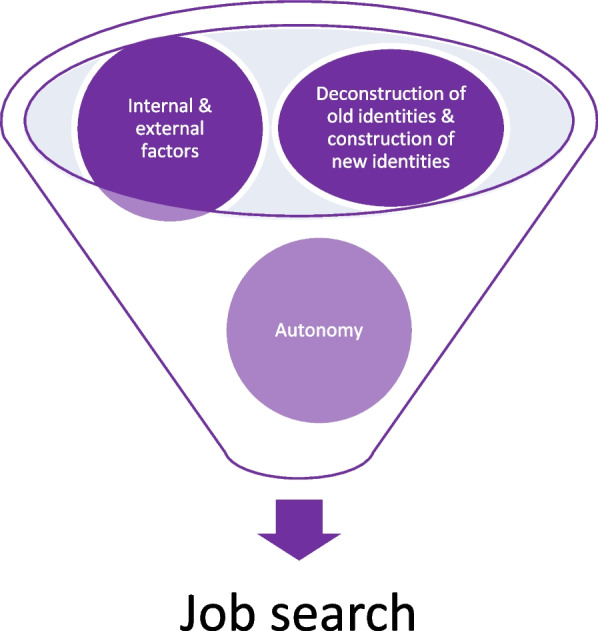
The search for a faculty position (“job search”) represents the culmination and merging of each theme that influenced the fellows’ transitions into and through fellowship

**Fig. 2 (Abstract A68):**
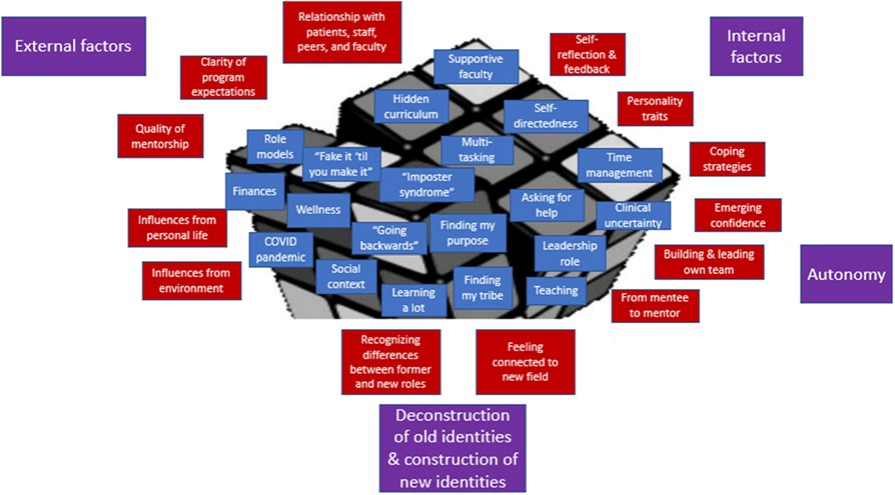
Explanatory model of the factors influencing the successful transitions of pediatric rheumatology fellows, based on multiple and multidimensional transitions (MMT) theory. The blue items are examples of codes; the red items are sub-themes; and the purple items are themes. A transition related to any one domain (code, sub-theme, or theme) prompts additional transitions in other domains

## A69 Characterizing JIA patients with persistent pain in the absence of active joint disease

### Daniella Schocken, Tracy Ting, Susmita Kashikar-Zuck

#### Cincinnati Children’s Hospital Medical Center, Cincinnati, OH, USA

##### **Correspondence:** Daniella Schocken

*Pediatric Rheumatology 2024*, **22(S1):**A69

Background : Understanding of pain in JIA remains incomplete, with varying estimates of the burden of chronic pain among children and adolescents with arthritis. Some children endorse pain even when disease activity is well-controlled, and comorbid juvenile fibromyalgia (JFM) may be present in as many as 1 in 6 children with JIA. The role of ongoing joint inflammation and other risk factors for chronic pain in JIA remain areas of active research. This study, part of a larger survey of pain and psychological resilience among children and adolescents with JIA, aims to examine characteristics of JIA patients reporting pain in the absence of active joint disease on physical exam.

Methods : 98 children aged 10 to 17 with an established diagnosis of JIA were recruited for study participation from the ambulatory clinic of a large academic medical center. Participants completed Numeric Rating Scales for pain intensity and well-being (0-10), the Patient Reported Outcomes Measurement Information System (PROMIS) Pediatric Pain Interference (PPI) Short Form v2.0, and the Pain and Symptom Assessment Tool (PSAT). Caregivers provided participants’ demographics and psychiatric health history. Exam findings at the time of study completion and arthritis history were obtained from chart review. A chi-squared test was applied to determine difference in frequency of findings between subgroups.

Results : Of 98 participants, 69 (70%) had no evidence of active arthritis or enthesitis on physical exam at the time of study participation. Of those 69 participants, 38 (55%) reported pain related to their arthritis during the previous 7 days. The average numeric pain rating reported among these participants was 3.89 (sd = 2.15), and their average reported overall well-being rating was 2.97 (sd = 2.35). The mean PPI t-score among these participants was 51.08 (sd = 10.52), and 10/38 (26%) demonstrated clinical characteristics consistent with comorbid JFM. A chi-square test of independence between active joint disease on physical exam and clinical features of JFM resulted in X^2 (3, *n* = 98) = 0.148 with a *p*-value of 0.99. 16/38 (42%) reported a history of anxiety and/or depression, and the chi-square between participants with and without pain in the absence of active joint disease and a history of mood disorders was X^2 (3, *n* = 69) = 1.999 (*p*-value = 0.57).

Conclusions : Over half of study participants with no evidence of arthritis or enthesitis on physical exam reported ongoing pain at the time their study visit, with many such participants demonstrating clinical features consistent with a diagnosis of comorbid JFM. However, there was no statistically significant relationship between the prevalence of active joint disease and JFM among study participants. Comorbid mood disorders had no relationship to the presence of pain in the absence of joint disease. These findings suggest that JFM and mood disorders should be considered independently from arthritis activity and managed proactively alongside joint disease. However, as no significant risk factors were identified in this cross-sectional study for persisting pain in the absence of active arthritis, further prospective, longitudinal research is warranted to better understand this clinical phenomenon.

IRB Statement : This research was approved by the Cincinnati Children’s Hospital Institutional Review Board (IRB) on May 2, 2022, under IRB ID#2022-0140.

Acknowledgements : The project described was supported by the National Institutes of Arthritis and Musculoskeletal Skin Diseases under Award Number P30AR076316. Additional support was provided by the National Center for Advancing Translational Sciences of the National Institutes of Health under Award Number UL1TR001425. The content is solely the responsibility of the authors and does not necessarily represent the official views of the NIH.

## A70 Factors driving disparities in glucocorticoid exposure among children with SLE in the CARRA registry

### William Soulsby^1^, Rebecca Olveda^1^, Jie He^2^, Laura Berbert^2^, Edie Weller^2^, Kamil Barbour^3^, Kurt Greenlund^3^, Laura Schanberg^4^, Emily von Scheven^1^, Aimee Hersh^5^, Mary Beth Son^2^, Joyce Chang^2^, Andrea Knight^6^, for the CARRA Registry Investigators^7^

#### ^1^University of California, San Francisco, CA, USA; ^2^Boston Children’s Hospital, Boston, MA, USA; ^3^Centers for Disease Control and Prevention (CDC), Atlanta, GA, USA; ^4^Duke University School of Medicine, Durham, NC USA; ^5^University of Utah, Salt Lake City, UT, USA; ^6^The Hospital for Sick Children and SickKids Research Institute, Toronto, Canada; ^7^ Childhood Arthritis and Rheumatology Research Alliance (CARRA)

##### **Correspondence:** William Soulsby

*Pediatric Rheumatology 2024*, **22(S1):**A70

Background : Differential glucocorticoid exposure and its toxicity may exacerbate racial disparities in lupus-related organ damage and mortality. Social determinants of health (SDOH) may drive differential medication use. We sought to determine how race and SDOH associate with cumulative glucocorticoid exposure over time in children with SLE. We hypothesized that minoritized race and living in more disadvantaged neighborhoods would be associated with greater average oral glucocorticoid exposure.

Methods : This was a cohort study of children with pSLE enrolled in the CARRA Registry between March 2017-December 2021 with a baseline enrollment visit, ≥1 follow up Registry visits, and a valid U.S. zip code. The primary exposures were self-identified race and/or ethnicity and area deprivation index (ADI). The primary outcomes were time-averaged mean prednisone dose (mg/day) and disease activity (SLEDAI-2K) over time. Associations between the primary exposures and time-averaged mean glucocorticoid dose were examined using univariate and multivariable linear regression models, adjusted for covariates (insurance status, age at enrollment, sex, major organ involvement, medication use at enrollment, disease duration at enrollment, and any secondary rheumatologic disease). Linear mixed effects models were used to model disease activity over time with a subject-level random intercept.

Results : A racially diverse cohort of 540 children with pSLE self-identified as 11% Asian, 27% Black, 23% Latino/a, 5% Other race, 25% White, and 9% selected more than one race. Black participants were more likely to be publicly insured, live in areas with higher social deprivation (ADI), and have renal disease (Table 1). In univariate analyses, Black race and living in areas in the highest ADI quartile were significantly associated with higher time-averaged mean prednisone dose (unadjustedβ=3.24, 95% CI: [0.88, 5.60] and β=4.68, 95% CI: [2.30, 7.06], respectively). Further adjustment for either insurance status or renal involvement attenuated this estimate, and neither race nor the highest quartile of ADI were significantly associated with time-averaged mean prednisone in the fully adjusted model (Table 2). Black race was independently associated with higher adjusted disease activity compared to White race (adjusted β=0.94, 95% CI: [0.11, 1.78] - Table 3).

Conclusions : Insurance status and renal disease were significant predictors of greater cumulative mean prednisone dose among children with pSLE in the CARRA Registry and attenuated the associations between Black race and neighborhood-level disadvantage with prednisone dose. In contrast, Black race was independently associated with higher disease activity over time, suggesting that there may be additional unmeasured SDOH driving this association. As Black participants were more likely to be publicly insured and live in areas of higher neighborhood disadvantage, structural confounding due to the unequal segregation of Black participants must be considered. Further work to understand the relationships between area-level segregation and individual SDOH is needed to identify points of intervention to improve pSLE outcomes.

IRB Statement : In this retrospective cohort study, we utilized data from the CARRA Registry, which is a prospective, multi-center registry developed to capture data about multiple rheumatic diseases, including systemic lupus erythematosus. We queried the Registry for all patients with cSLE and then analyzed clinical and demographic data. We used de-identified data from all active clinical sites between March 2017 and December 2021. Institutional Review Board approval was obtained at each enrolling site.

Acknowledgements : The authors wish to acknowledge CARRA and the ongoing Arthritis Foundation financial support of CARRA. This work could not have been accomplished without the aid of the following organizations: The NIH’s National Institute of Arthritis and Musculoskeletal and Skin Diseases (NIAMS) & the Arthritis Foundation. We would also like to thank all participants and hospital sites that recruited patients for the CARRA Registry. The authors thank the following CARRA Registry site principal investigators: K. Abulaban, C. Aguiar Lapsia, S. Ardoin, L. Barillas-Arias, M. Basiaga, K. Baszis, H. Brunner, H. Bukulmez, E. Chalom, J. Chang, D. Co, K. Cook, A. Cooper, C. Correll, T. Davis, F. Dedeoglu, M. DeGuzman, A. Dhanrajani, K. Ede, B. Edelheit, B. Feldman, I. Ferguson, D. Glaser, D. Goldsmith, B. Gottlieb, T. Graham, T. Griffin, T. Hahn, L. Harel, O. Harry, M. Hollander, S. Hong, M. Horwitz, J. Hsu, A. Huber, L. Imundo, C. Inman, P. Kahn, S. Kim, D. Kingsbury, M. Klein-Gitelman, L. Lim, M. Mannion, D. McCurdy, D. Milojevic, S. Mohan, T. Moore, K. Moore, L. Moorthy, S. Nativ, M. Natter, K. Onel, J. Patel, S. Prahalad, C. Rabinovich, A. Robinson, T. Ronis, M. Rosenkranz, N. Ruth, S. Sabbagh, K. Schikler, C. Schutt, E. Sloan, J. Spitznagle, Y. Sterba Rakovchik, K. Stewart, G. Syverson, S. Tarvin, M. Tesher, D. Toib, M. Toth, M. Twilt, H. Van Mater, D. Wahezi, P. Weiss, J. Weiss, L. Woolnough, E. Wu, A. Yalcindag, Y. Zhao.

**Table 1 (Abstract A70):** Demographic and clinical characteristics of children with pSLE in the CARRA Registry (March 2017 – December 2021)

	Total *N*=540	Asian *N*=58	Black *N*=146	Latino/a *N*=124	Other *N*=30	White *N*=135	More than one race *N*=47
Age at enrollment (years), median (IQR)	15 (12, 16)	15 (12,16)	15 (13, 17)	15 (13,16)	15 (12, 16)	15 (13, 16)	14 (11,16)
Female sex, N (%)	467 (87)	41 (81)	127 (87)	107 (86)	25(83)	119 (88)	42(89)
Insurance, N (%)							
Private	257 (48)	40 (69)	50 (35)	32 (26)	18 (60)	90 (67)	27 (58)
Public	248 (46)	16 (27)	92 (63)	77 (62)	12(40)	35 (26)	16 (34)
Other/Non-U.S.	19 (3)	1 (2)	2 (1)	6 (5)	0 (0)	8 (6)	2 (4)
None	16 (3)	1 (2)	2 (1)	9 (7)	0 (0)	2 (1)	2 (4)
Area Deprivation Index^a^ (ADI), N (%)							
0-25%ile	169 (31)	37 (64)	18 (12)	38 (31)	14 (47)	45 (33)	17 (36)
26-50%ile	144 (27)	17 (29)	29 (20)	38 (30)	6 (20)	39 (29)	15 (32)
51-75%ile	114 (21)	3 (5)	39 (27)	28 (23)	7 (23)	30 (22)	7 (15)
76-100%ile	113 (21)	1 (2)	60 (41)	20 (16)	3 (10)	21 (16)	8 (17)
Major Organ Involvement, N (%)							
CNS	78 (14)	6 (10)	26 (18)	13 (11)	9 (30)	11 (8)	13 (28)
Renal	277 (51)	24 (41)	83 (57)	61 (49)	16 (53)	68 (50)	25 (53)
CV/Pulm	152 (28)	12 (21)	56 (38)	35 (28)	5 (17)	32 (24)	12 (27)
Baseline Medication, N (%)							
Belimumab	43 (8)	3 (5)	20 (14)	5 (4)	2 (7)	9 (7)	4 (9)
Conventional DMARD^b^	462 (86)	53 (91)	125 (86)	102 (82)	28 (93)	115 (85)	39 (83)
Rituximab or Cyclophosphamide	163 (30)	13 (22)	59 (40)	34 (27)	10 (33)	27 (20)	20 (43)
Anti-malarial	523 (97)	54 (93)	144 (99)	120 (97)	30 (100)	129 (96)	46 (98)
Any secondary rheumatologic disease, N (%)	63 (12)	6 (10)	22 (15)	9 (7)	4 (13)	16 (12)	6 (13)
Time from diagnosis to enrollment (years), median (IQR)	0.4 (0.1, 1.2)	0.8 (0.1, 2.0)	0.4 (0.1, 1.4)	0.3 (0.1, 0.8)	0.3 (0.1, 1.0)	0.4 (0.1, 1.2)	0.3 (0.1, 1.2)
Duration of Registry follow-up time (years), median (IQR)	2.1 (1.3, 2.8)	2.4 (1.8, 2.9)	1.9 (1.0, 2.7)	2.1 (1.3, 2.7)	2.1 (1.5, 2.7)	2.2 (1.3, 2.9)	2.0 (1.1, 3.0)
Time adjusted mean prednisone dose (mg), median (IQR)	6.6 (1.7, 12.6)	4.1 (0, 7.4)	8.3 (3.5, 19.0)	6.5 (1.9, 12.5)	5.9 (2.6, 9.9)	6.4 (0.2, 11.4)	6.5 (1.4, 13.5)
Any prednisone restart or dose increase during study period, N (%)	199 (37)	19 (33)	66 (45)	42 (34)	13 (43)	43 (32)	16 (34)

^a^Area Deprivation Index (ADI) is a national measure of neighborhood social disadvantage at the level of census block group incorporating U.S. Census data (including American Community Survey results) that incorporates multiple social domains. Higher scores suggest more disadvantaged areas

^b^Conventional DMARDs include azathioprine, leflunomide, methotrexate, mycophenolate mofetil, mycophenolic acid, sirolimus, sulfasalazine, and tacrolimus

**Table 2 (Abstract A70):** Factors associated with time-adjusted mean prednisone dose among children with pSLE in the CARRA Registry

Value	Unadjusted β (95% CI)	Adjusted β (95% CI)
Race and Ethnicity		
Asian	-2.32 (-5.42, 0.79)	-1.16 (-4.18, 1.86)
Black	**3.24 (0.88, 5.60)** ^**^	1.43 (-0.97, 3.84)
Latino/a	0.25 (-2.21, 2.71)	-0.79 (-3.23, 1.66)
More than one	0.11 (-3.24, 3.46)	-0.14 (-3.36, 3.09)
Other	-1.33 (-5.32, 2.66)	-1.42 (-5.25, 2.41)
White	Reference	Reference
Area Deprivation Index (ADI)		
1 ≤ ADI Rank ≤ 25	Reference	Reference
26 ≤ ADI Rank ≤ 50	-1.23 (-3.46, 0.99)	-2.4 (-4.6, -0.2)^*^
51 ≤ ADI Rank ≤ 75	1.46 (-0.92, 3.83)	-0.45 (-2.89, 1.99)
V76 ≤ ADI Rank ≤ 100	**4.68 (2.3, 7.06)** ^***^	1.37 (-1.22, 3.96)
Insurance status		
Private	Reference	Reference
Public	**2.50 (0.74, 4.27)** ^**^	**1.99 (0.13, 3.84)** ^*^
Other or non-US insurance	-0.14 (-4.85, 4.58)	0.73 (-3.81, 5.27)
Uninsured	4.97 (-0.14, 10.09)	3.47 (-1.51, 8.45)
Age at enrollment	0.08 (-0.21, 0.38)	0.23 (-0.06, 0.53)
Male vs. Female sex	0.14 (-2.38, 2.66)	0.58 (-1.79, 2.95)
Central nervous system involvement	-1.22 (-3.67, 1.23)	-1.89 (-4.26, 0.47)
Renal involvement	**4.72 (3.04, 6.39)** ^***^	**4.62 (2.9, 6.33)** ^***^
Cardiopulmonary involvement	**2.7 (0.80, 4.60)** ^**^	1.38 (-0.48, 3.24)
Conventional DMARD use at enrollment	1.51 (-0.93, 3.96)	0.23 (-2.18, 2.63)
Rituximab or cyclophosphamide use at enrollment	2.19 (0.32, 4.05)*	0.48 (-1.4, 2.36)
Any secondary rheumatologic disease	-1.24 (-3.92, 1.44)	-1.16 (-3.7, 1.37)
Disease duration (yrs) at enrollment	**-1.26 (-1.81, -0.71)** ^***^	**-1.49 (-2.04, -0.94)** ^***^

^*^ *p*<0.05

^**^ *p*<0.01

^***^ *p*<0.001

**Table 3 (Abstract A70):** Factors associated with disease activity over time among children with SLE (CARRA Registry 2017-2021)

	Unadjusted^a^ β (95% CI)	Adjusted β (95% CI)
Race and Ethnicity		
Asian	-1.00 (-2.11, 0.10)	-0.40 (-1.44, 0.64)
Black	**1.49 (0.64, 2.34)** ^**^	**0.94 (0.11, 1.78)** ^*^
Latino/a	0.79 (-0.09, 1.67)	0.56 (-0.28, 1.41)
More than one	**1.36 (0.16, 2.56)** ^*^	1.03 (-0.09, 2.15)
Other	0.22 (-1.19, 1.64)	0.07 (-1.24, 1.39)
White	Reference	Reference
Area Deprivation Index (ADI)		
1 ≤ ADI Rank ≤ 25	Reference	Reference
26 ≤ ADI Rank ≤ 50	0.55 (-0.26, 1.36)	-0.01 (-0.77, 0.75)
51 ≤ ADI Rank ≤ 75	**1.11 (0.24, 1.97)** ^*^	0.39 (-0.45, 1.23)
76 ≤ ADI Rank ≤ 100	**1.66 (0.79, 2.54)** ^***^	0.44 (-0.47, 1.34)
Insurance status		
Private	Reference	Reference
Public	0.64 (0.00, 1.28)	0.22 (-0.43, 0.86)
Other or non-US insurance	0.78 (-0.96, 2.53)	0.75 (-0.86, 2.36)
Uninsured	**2.73 (0.87, 4.59)** ^**^	**1.87 (0.14, 3.60)** ^*^
Age at enrollment	0.01 (-0.09, 0.12)	0.07 (-0.03, 0.17)
Male vs. Female sex	-0.85 (-1.75, 0.06)	-0.66 (-1.47, 0.16)
CNS involvement	**1.18 (0.30, 2.06)** ^**^	0.78 (-0.04, 1.60)
Renal involvement	**2.40 (1.81, 3.00)** ^***^	**2.26 (1.67, 2.86)** ^***^
Cardiopulmonary involvement	**1.31 (0.62, 1.99)** ^***^	0.6 (-0.04, 1.24)
Conventional DMARD use at enrollment	0.80 (-0.14, 1.72)	0.14 (-0.74, 1.01)
Rituximab or cyclophosphamide use at enrollment	**1.32 (0.65, 1.99)** ^***^	0.29 (-0.35, 0.94)
Any secondary rheumatologic disease	-0.43 (-1.41, 0.54)	-0.43 (-1.31, 0.45)
Disease duration (yrs) at each visit	**-0.41 (-0.61, -0.21)** ^***^	**-0.44 (-0.63, -0.24)** ^***^

Linear mixed effects models of disease activity, as measured by the Systemic Lupus Disease Activity Index 2000 (SLEDAI-2K), with random intercept to account for within-subject correlation

^*^ *p*<0.05

^**^ *p*<0.01

^***^ *p*<0.001

^a^All models account for time from first Registry visit

## A71 Cumulative social disadvantage is associated with disease activity and functional disability in juvenile idiopathic arthritis: an analysis of the CARRA registry

### William Soulsby^1^, Erica Lawson^1^, John Boscardin^1^, Emily von Scheven^1^, for the CARRA Registry Investigators^2^

#### ^1^University of California, San Francisco, CA, USA; ^2^Childhood Arthritis and Rheumatology Research Alliance (CARRA)

##### **Correspondence:** William Soulsby

*Pediatric Rheumatology 2024*, **22(S1):**A71

Background : The impact of race and social determinants of health (SDoH) on health outcomes in juvenile idiopathic arthritis (JIA) remains poorly understood. Prior disparities research in JIA has largely analyzed these complex, intertwined social variables as independent risk factors and have reported inconsistent results of their impact on clinical outcomes, such as joint damage, pain, and disability. These inconsistencies may result from a failure to investigate interrelationships between social variables. A recent analysis demonstrated an association between cumulative social disadvantage and childhood arthritis using a combined score. In this analysis of the Childhood Arthritis and Rheumatology Research Alliance (CARRA) Registry, we used a similar method to investigate the effect of cumulative social disadvantage on disease activity and functional disability in JIA.

Methods : This is a cohort study of subjects with JIA enrolled in the CARRA Registry between July 2015-January 2022 with at least one Registry visit including all components of the clinical Juvenile Arthritis Disease Activity Score (cJADAS) and Child Health Assessment Questionnaire (CHAQ) score. A cumulative social disadvantage score was created, with a score of 1 given for each of the following: income (household income <$50,000/year), guardian education (high school or less), insurance (public insurance or none), and non-White race. Any missing components were given a score of 0. Univariate and multivariable logistic regression models, adjusted for age at enrollment, sex, JIA category, and any use of a conventional (cs-), biologic (bs-) DMARD or small molecule, were used to estimate the odds of persistent disease activity (oligoarticular JIA – cJADAS >1.0; all other categories >2.5) and functional disability (CHAQ score > 0). Random effect was included in the regression models to account for repeated measures.

Results : 9,672 subjects with JIA were identified (Table 1). Oligoarticular and RF- polyarticular JIA were the most common JIA subtypes reported. The majority of cohort required DMARD treatment (68.7%). 48.9% of the cohort had exposure to at least one variable associated with the combined score. 47.9% of patients were classified as having high cJADAS, and 56.8% had a high CHAQ score. In both unadjusted and adjusted analysis, cumulative social disadvantage with higher odds of active disease, highest for a score of 3 (Table 2 - adjusted odds ratio [aOR] 2.31, 95% CI: 1.97-2.71), and functional disability, also highest for a score of 3 (Table 3 - aOR 3.94, 95% CI: 3.15-4.93).

Conclusions : Cumulative social disadvantage was associated with higher odds of active disease and functional disability compared to those not experiencing these exposures among JIA subjects in the CARRA Registry. Exposure to multiple variables associated with social disadvantage confers higher risk of poor outcomes in JIA. To mitigate these disparities, targeted social risk screening adopted within pediatric rheumatology clinics should be studied as a potential intervention to identify at-risk patients who may benefit from social network support or other programming, such as patient navigation services.

IRB Statement : An IRB application was submitted for this study and determined by the University of California, San Francisco Institutional Review Board to be considered not human subjects research and therefore exempt from IRB review (study #22-36233; reference #338505).

Acknowledgements : The authors wish to acknowledge CARRA and the ongoing Arthritis Foundation financial support of CARRA. The authors also wish to acknowledge the Arthritis Foundation for their financial support of this research. Dr. Soulsby and this work were funded by an Arthritis Foundation Diversity, Equity, and Inclusion Research in Health Outcomes grant (#891767). This work could not have been accomplished without the aid of the following organizations: The NIH’s National Institute of Arthritis and Musculoskeletal and Skin Diseases (NIAMS) & the Arthritis Foundation. We would also like to thank all participants and hospital sites that recruited patients for the CARRA Registry. The authors thank the following CARRA Registry site principal investigators: K. Abulaban, C. Aguiar Lapsia, S. Ardoin, L. Barillas-Arias, M. Basiaga, K. Baszis, H. Brunner, H. Bukulmez, E. Chalom, J. Chang, D. Co, K. Cook, A. Cooper, C. Correll, T. Davis, F. Dedeoglu, M. DeGuzman, A. Dhanrajani, K. Ede, B. Edelheit, B. Feldman, I. Ferguson, D. Glaser, D. Goldsmith, B. Gottlieb, T. Graham, T. Griffin, T. Hahn, L. Harel, O. Harry, M. Hollander, S. Hong, M. Horwitz, J. Hsu, A. Huber, L. Imundo, C. Inman, P. Kahn, S. Kim, D. Kingsbury, M. Klein-Gitelman, L. Lim, M. Mannion, D. McCurdy, D. Milojevic, S. Mohan, T. Moore, K. Moore, L. Moorthy, S. Nativ, M. Natter, K. Onel, J. Patel, S. Prahalad, C. Rabinovich, A. Robinson, T. Ronis, M. Rosenkranz, N. Ruth, S. Sabbagh, K. Schikler, C. Schutt, E. Sloan, J. Spitznagle, Y. Sterba Rakovchik, K. Stewart, G. Syverson, S. Tarvin, M. Tesher, D. Toib, M. Toth, M. Twilt, H. Van Mater, D. Wahezi, P. Weiss, J. Weiss, L. Woolnough, E. Wu, A. Yalcindag, Y. Zhao.

**Table 1 (Abstract A71):** Demographic and clinical characteristics of pediatric patients with juvenile idiopathic arthritis (JIA) in the CARRA Registry

	N (%)
**Total N**	9672
**Median age at enrollment in years (IQR)**	11.7 (7.3, 15.1)
**Sex**	
Female	6720 (69.9)
Male	2892 (30.1)
**JIA Category**	
Oligoarticular JIA	3417 (35.7)
RF+ polyarticular JIA	611 (6.4)
RF- polyarticular JIA	2830 (29.6)
Systemic JIA	745 (7.8)
Enthesitis-related arthritis	1012 (10.6)
Psoriatic arthritis	703 (7.4)
Undifferentiated arthritis	244 (2.6)
**Median Area Deprivation Index (IQR)**	37.0 (20.0, 60.0)
**Ever use of a csDMARD, bsDMARD, or small molecule** ^**a**^	6643 (68.7)
**Components of cumulative social disadvantage score**	
Non-white race	2236 (23.1)
Household income <$50,000/year	1842 (19.0)
Guardian education of high school or less	1587 (16.4)
Publicly insured or no insurance	2546 (26.3)
**Cumulative social disadvantage score**	
0	4938 (51.1)
1	2458 (25.4)
2	1299 (13.4)
3	753 (7.8)
4	224 (2.3)
**Components of the cJADAS** ^**b**^	
Median Physical Global Assessment	1.0 (0.0, 3.0)
Median Parent/patient Global Assessment	2.0 (0.0, 4.0)
Active Joint Count	1.0 (0.0, 3.0)
**Median cJADAS (IQR)**	2.0 (0.0, 6.0)
**High cJADAS** ^**c**^	4633 (47.9)
**Median CHAQ** ^**d**^ **(IQR)**	0.1 (0.0, 0.6)
**High CHAQ** ^**e**^	5029 (56.8)

^a^Conventional synthetic DMARD, biologic DMARD

^b^Clinical Juvenile Arthritis Disease Activity Score

^c^Defined in subjects with oligoarticular JIA as cJADAS ≥1.1 and in all other JIA categories as >2.5

^d^Child Health Assessment Questionnaire

^e^Defined as a CHAQ score of >0

**Table 2 (Abstract A71):** Mixed effects model to estimate the effect of cumulative social disadvantage on persistent disease activity in children with JIA in the CARRA Registry

Cumulative Social Disadvantage Score	Unadjusted OR (95% CI), ***p***-value	Adjusted^**a**^ OR (95% CI), p-value
0	—	—
1	1.32 (1.19, 1.46), <0.001	1.34 (1.21, 1.48), <0.001
2	1.70 (1.50, 1.94), <0.001	1.82 (1.61, 2.07), <0.001
3	2.09 (1.77, 2.46), <0.001	2.31 (1.97, 2.71), <0.001
4	1.92 (1.45, 2.54), <0.001	2.18 (1.65, 2.86), <0.001

^a^Model adjusted for age at enrollment, sex, JIA category, and any use of a cs- or bDMARD or small molecule

**Table 3 (Abstract A71):** Mixed effects model to estimate the effect of cumulative social disadvantage on persistent functional disability in children with JIA in the CARRA Registry

Cumulative Social Disadvantage Score	Unadjusted OR (95% CI), ***p***-value	Adjusted^**a**^ OR (95% CI), p-value
0	—	—
1	1.62 (1.41, 1.87), <0.001	1.64 (1.42, 1.88), <0.001
2	3.61 (3.02, 4.31), <0.001	3.49 (2.92, 4.17), <0.001
3	3.89 (3.11, 4.89), <0.001	3.94, (3.15, 4.93), <0.001
4	3.81 (2.59, 5.59), <0.001	3.91 (2.67, 5.74), <0.001

^a^Model adjusted for age at enrollment, sex, JIA category, and any use of a cs- or bDMARD or small molecule

